# Genomic insights into the *Ixodes scapularis* tick vector of Lyme disease

**DOI:** 10.1038/ncomms10507

**Published:** 2016-02-09

**Authors:** Monika Gulia-Nuss, Andrew B. Nuss, Jason M. Meyer, Daniel E. Sonenshine, R. Michael Roe, Robert M. Waterhouse, David B. Sattelle, José de la Fuente, Jose M. Ribeiro, Karine Megy, Jyothi Thimmapuram, Jason R. Miller, Brian P. Walenz, Sergey Koren, Jessica B. Hostetler, Mathangi Thiagarajan, Vinita S. Joardar, Linda I. Hannick, Shelby Bidwell, Martin P. Hammond, Sarah Young, Qiandong Zeng, Jenica L. Abrudan, Francisca C. Almeida, Nieves Ayllón, Ketaki Bhide, Brooke W. Bissinger, Elena Bonzon-Kulichenko, Steven D. Buckingham, Daniel R. Caffrey, Melissa J. Caimano, Vincent Croset, Timothy Driscoll, Don Gilbert, Joseph J. Gillespie, Gloria I. Giraldo-Calderón, Jeffrey M. Grabowski, David Jiang, Sayed M. S. Khalil, Donghun Kim, Katherine M. Kocan, Juraj Koči, Richard J. Kuhn, Timothy J. Kurtti, Kristin Lees, Emma G. Lang, Ryan C. Kennedy, Hyeogsun Kwon, Rushika Perera, Yumin Qi, Justin D. Radolf, Joyce M. Sakamoto, Alejandro Sánchez-Gracia, Maiara S. Severo, Neal Silverman, Ladislav Šimo, Marta Tojo, Cristian Tornador, Janice P. Van Zee, Jesús Vázquez, Filipe G. Vieira, Margarita Villar, Adam R. Wespiser, Yunlong Yang, Jiwei Zhu, Peter Arensburger, Patricia V. Pietrantonio, Stephen C. Barker, Renfu Shao, Evgeny M. Zdobnov, Frank Hauser, Cornelis J. P. Grimmelikhuijzen, Yoonseong Park, Julio Rozas, Richard Benton, Joao H. F. Pedra, David R. Nelson, Maria F. Unger, Jose M. C. Tubio, Zhijian Tu, Hugh M. Robertson, Martin Shumway, Granger Sutton, Jennifer R. Wortman, Daniel Lawson, Stephen K. Wikel, Vishvanath M. Nene, Claire M. Fraser, Frank H. Collins, Bruce Birren, Karen E. Nelson, Elisabet Caler, Catherine A. Hill

**Affiliations:** 1 Department of Entomology, Purdue University, West Lafayette, Indiana 47907, USA; 2 Department of Biological Sciences, Old Dominion University, Norfolk, Virginina 23529, USA; 3 Department of Entomology, North Carolina State University, Raleigh, North Carolina 27695, USA; 4 Department of Genetic Medicine and Development, University of Geneva Medical School, Geneva 1211, Switzerland; 5 Swiss Institute of Bioinformatics, Geneva 1211, Switzerland; 6 Computer Science and Artificial Intelligence Laboratory, Massachusetts Institute of Technology, Cambridge, Massachusetts 02139, USA; 7 The Broad Institute of MIT and Harvard, Cambridge, Massachusetts 02142, USA; 8 Centre for Respiratory Biology, UCL Respiratory Department, Division of Medicine, University College London, Rayne Building, 5 University Street, London WC1E 6JF, UK; 9 SaBio, Instituto de Investigación en Recursos Cinegéticos, IREC-CSIC-UCLM-JCCM, Ronda de Toledo sn, Ciudad Real 13005, Spain; 10 Department of Veterinary Pathobiology, Center for Veterinary Health Sciences, Oklahoma State University, 250 McElroy Hall, Stillwater, Oklahama 74078, USA; 11 Laboratory of Malaria and Vector Research, NIAID, Rockville, Maryland 20852, USA; 12 VectorBase/EMBL-EBI, Wellcome Trust Genome Campus, Cambridge CB10 1SD, UK; 13 Bioinformatics Core, Purdue University, West Lafayette, Indiana 47907, USA; 14 J. Craig Venter Institute, Rockville, Maryland 20850, USA; 15 Genome Sequencing and Analysis Program, Broad Institute, Cambridge, Massachusetts 02142, USA; 16 Department of Biological Sciences, University of Notre Dame, Notre Dame, Indiana 46556, USA; 17 Departament de Genètica & Institut de Recerca de la Biodiversitat (IRBio), Universitat de Barcelona, Barcelona E-08028, Spain; 18 Vascular Physiopathology, Centro Nacional de Investigaciones Cardiovasculares, Madrid 28029, Spain; 19 Department of Medicine, Division of Infectious Diseases, University of Massachusetts Medical School, Worcester, Massachusetts 01605, USA; 20 Department of Medicine, University of Connecticut Health Center, Farmington, Connecticut 06030, USA; 21 Center for Integrative Genomics, Faculty of Biology and Medicine, University of Lausanne, Lausanne CH-1015, Switzerland; 22 Genetics, Bioinformatics, and Computational Biology Program, Virginia Bioinformatics Institute at Virginia Tech, Blacksburg, Virginia 24061, USA; 23 Department of Biology, Indiana University, Bloomington, Indiana 47405, USA; 24 Department Biological Sciences, Markey Center for Structural Biology, Purdue University, West Lafayette, Indiana 47907, USA; 25 Department of Biochemistry, Virginia Tech, Blacksburg, Virginia 24061, USA; 26 Department of Microbial Molecular Biology, Agricultural Genetic Engineering Research Institute, Giza 12619, Egypt; 27 Department of Entomology, Texas A&M University, College Station, Texas 77843, USA; 28 Department of Entomology, Kansas State University, Manhattan, Kansas 66506, USA; 29 Department of Entomology, University of Minnesota, St Paul, Minnesota 55108, USA; 30 Department of Neurosystems, Faculty of Life Sciences, University of Manchester, Manchester M13 9PT, UK; 31 Department of Bioengineering and Therapeutic Sciences, University of California, San Francisco, California 94143, USA; 32 Department of Entomology, The Pennsylvania State University, University Park, Pennsylvania 16802, USA; 33 Department of Entomology, Center for Disease Vector Research, University of California, Riverside, California 92506, USA; 34 Department of Pathology, Cambridge Genomic Services, University of Cambridge, Cambridge CB2 1QP, UK; 35 Department of Physiology, School of Medicine-CIMUS-Instituto de Investigaciones Sanitarias, University of Santiago de Compostela, Santiago de Compostela 15782, Spain; 36 Department of Experimental and Health Sciences, Universidad Pompeu Fabra, Barcelona 08003, Spain; 37 Department of Biological Sciences, California State Polytechnic University, Pomona, California 91768, USA; 38 Parasitology Section, School of Chemistry & Molecular Biosciences, University of Queensland, Brisbane, Queensland 4072, Australia; 39 GeneCology Research Centre, Faculty of Science, Health, Education and Engineering, University of the Sunshine Coast, Maroochydore, Queensland 4556, Australia; 40 Department of Biology, Center for Functional and Comparative Insect Genomics, University of Copenhagen, Copenhagen DK-2100, Denmark; 41 Department of Microbiology, Immunology & Biochemistry, University of Tennessee Health Science Center, Memphis, Tennessee 38163, USA; 42 Cancer Genome Project, Wellcome Trust Sanger Institute, Hinxton CB10 1SA, UK; 43 Department of Biochemistry, Genetics and Immunology, University of Vigo, Vigo 36310, Spain; 44 Department of Entomology, University of Illinois at Urbana-Champaign, Urbana, Illinois 61801, USA; 45 Department of Medical Sciences, Frank H. Netter MD School of Medicine at Quinnipiac University, Hamden, Connecticut 06518, USA; 46 Institute for Genome Sciences, University of Maryland, School of Medicine, Baltimore, Maryland 21201, USA

## Abstract

Ticks transmit more pathogens to humans and animals than any other arthropod. We describe the 2.1 Gbp nuclear genome of the tick, *Ixodes scapularis* (Say), which vectors pathogens that cause Lyme disease, human granulocytic anaplasmosis, babesiosis and other diseases. The large genome reflects accumulation of repetitive DNA, new lineages of retro-transposons, and gene architecture patterns resembling ancient metazoans rather than pancrustaceans. Annotation of scaffolds representing ∼57% of the genome, reveals 20,486 protein-coding genes and expansions of gene families associated with tick–host interactions. We report insights from genome analyses into parasitic processes unique to ticks, including host ‘questing', prolonged feeding, cuticle synthesis, blood meal concentration, novel methods of haemoglobin digestion, haem detoxification, vitellogenesis and prolonged off-host survival. We identify proteins associated with the agent of human granulocytic anaplasmosis, an emerging disease, and the encephalitis-causing Langat virus, and a population structure correlated to life-history traits and transmission of the Lyme disease agent.

## Abstract (web summary)

Ticks transmit a large number of pathogens that cause human diseases. Here, the authors sequence the genome of the tick *Ixodes scapularis* and uncover expansion of genes associated with parasitic processes unique to ticks and tick-host interactions.

## Graphical abstract

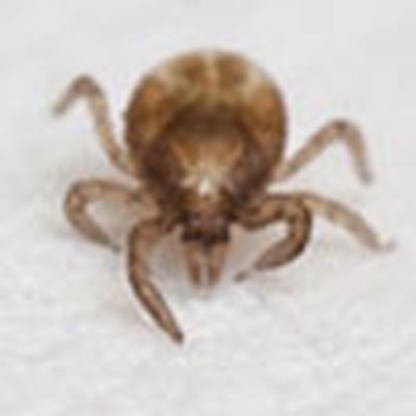

Ticks (subphylum Chelicerata: suborder Ixodida) are notorious ectoparasites and vectors of human and animal pathogens, transmitting a greater diversity of infectious agents than any other group of blood-feeding arthropods. Ticks are responsible for serious physical damage to the host, including blood loss and toxicosis. Tick-borne diseases result in significant morbidity and thousands of human and animal deaths annually. The genus *Ixodes* includes multiple species of medical and veterinary importance, most notably serving as vectors of Lyme borreliosis in North America, Europe and Asia. Lyme disease is the most prevalent vector-borne disease in the northern hemisphere^1^. In the USA, 22,014 confirmed human cases were reported in 2012 (ref. 2), with ∼10-fold more infections suspected^3^. In Europe, ∼65,500 Lyme borreliosis patients are documented annually^4^. In the USA, *Ixodes scapularis* also vectors the infectious agents that cause human babesiosis, human granulocytic anaplasmosis, tick-borne relapsing fever and Powassan encephalitis. The increased incidence and distribution of Lyme disease and other tick-borne diseases^5^ necessitates new approaches for vector control.

Subphyla Chelicerata (includes ticks and mites) and Mandibulata (includes insects) shared a common ancestor 543–526 million years ago (Myr ago)^6^. Tick life cycles differ in many aspects from those of insects (Fig. 1) and include long periods of host attachment and blood feeding, as well as months living off-host without feeding. ‘Three-host' ticks such as *I. scapularis* require a host blood meal at each life stage. Feeding occurs over several days and involves a period of slow feeding followed, after mating and insemination, by rapid consumption of a large blood meal. The synthesis of flexible new cuticle is a unique feature that permits the engorgement of ixodid ticks during feeding^7^. Moulting occurs off-host, and the subsequent developmental stage will ‘quest' for a new host from vegetation. *I. scapularis* exhibits a wide host range including small, ground-dwelling vertebrates, birds, white-tailed deer and humans.

The *I. scapularis* genome assembly is the first for a medically important acarine species. It affords opportunities for comparative evolutionary analyses between disease vectors from diverse arthropod lineages and serves as a resource for the exploration of how ticks parasitize and transmit pathogens to their vertebrate hosts.

## Results

### The first genome assembly for a tick vector of disease

The assembly, IscaW1, comprises 570,640 contigs in 369,495 scaffolds (N_50_=51,551 bp) representing 1.8 Gbp, including gaps (Table 1, Supplementary Table 2). The *ab initio* annotation of 18,385 scaffolds >10 Kbp in length and representing 1.2 Gbp (57% of the genome) predicted 20,486 protein-coding genes, and 4,439 non-coding RNA genes (Supplementary Figs 1–6 and Supplementary Table 3). Ixodid ticks typically have haploid genomes that exceed 1 Gbp (ref. 8). In contrast, the 90 Mbp genome of the two-spotted spider mite, *Tetranychus urticae*, a horticultural pest, is the smallest of any known arthropod, and contains <10% transposable elements^9^. Repetitive DNA is estimated to comprise ∼70% of the *I. scapularis* genome^10^, reflecting an extreme case of tandem repeat and transposable element accumulation.

The *I. scapularis* genome possesses 26 acrocentric autosomes and two sex chromosomes (XX:XY)^11^,^12^. Fluorescent *in situ* hybridization (FISH)-based physical mapping was used to develop a karyotype and physical map^12^ (Fig. 2; Supplementary Tables 12 and 15). Mapping revealed that tandem repeat accumulation in centromeric or peri-centromeric regions, also noted in some other arthropods^13^, is high in *I. scapularis* and comprises ∼40% of genomic DNA^10^. The low complexity tandem repeat families, ISR-1, ISR-2 and ISR-3, account for ∼8% of the genome^12^ (Supplementary Text). The most abundant ISR-2 (95–99 bp; ∼7% of the genome) is localized at the near-terminal heterochromatic regions of the chromosomes (Fig. 2).

The moderately repetitive fraction of the genome (∼30% of genomic DNA^10^) contains numerous copies of Class I and Class II transposable elements (Supplementary Tables 13 and 14 and Supplementary Text). For example, 41 well-represented elements (that is, comprising a full-length canonical and/or consensus sequence (Supplementary Figs 7 and 8)) of the long-terminal repeat (LTR) retro-transposon family, estimated to make up <1% of the genome, were identified. Thirty-seven members of the Ty3/gypsy group were identified, with the remainder being Pao/Bel-like. Two (Mag and CsRn1) of the six well-known insect Ty3/gypsy lineages were confirmed in the tick and two new clades, Squirrel and Toxo, are likely specific to the subphylum Chelicerata (Supplementary Fig. 8). Structural characterization of elements belonging to these lineages revealed shared features that include the CCHC gag and GPY/F integrase domains, and two ORFs matching gag and pol. The LTRs possess the TG..CA pattern^14^ and their integration generates a duplication of 4 bp.

Non-LTR retro-transposons comprise about 6.5% of the genome. Sequence conservation and transposable element copy number suggest recent activity in the *I. scapularis* CR1, I and L2 clades; these elements are also abundant in birds, mammals and lizards, and the possibility of horizontal transposable element transmission warrants further investigation. The R2, RTE and LOA non-LTR retro-transposon clades found in mosquitoes and *Drosophila* were not identified in the tick. Seemingly intact *mariner* and *piggyBac* transposable elements were identified, indicating possible recent or active transposition, and 234 miniature inverted-repeat transposable elements (MITEs) were annotated. These MITEs range in copy number from 50 to 14,500 and occupy ∼5% of the genome. Collectively, these findings suggest a genome permissive to high repeat accumulation.

Approximately 60% of tick genes have recognizable orthologs in other arthropods, about half of which are maintained across representative species of the major arthropod lineages (Supplementary Fig. 9). Approximately 50% of the remaining genes have homologs and ∼1/5th of tick genes appear unique (*T. urticae* has a similar proportion of unique genes); these provide an important resource to understand tick-specific processes and develop highly selective interventions. Analysis of gene models and 20,901 tentative consensus sequences (the Gene Index Project; compbio.dfci.harvard.edu/tgi) compiled from 192,461 expressed sequence tags (ESTs) identified ∼22% of *I. scapularis* genes as paralogs (Supplementary Note 1 and Supplementary Table 11). This is in line with estimates for *Homo sapiens* (15%)^15^ and the nematode, *Caenorhabditis elegans* (20%)^16^. Complementary analyses of paralogs^17^ suggest two duplication events in *I. scapularis*, involving hundreds of genes that took place within the last 40 million years, consistent with the radiation of ticks through Europe, America and Africa. The tick mitochondrial genome retains the inferred ancestral arthropod organization as predicted by its phylogenetic position^18^ (Supplementary Fig. 10).

The genome-scale quantitative molecular species phylogeny (Supplementary Text) inferred from single-copy orthologs from OrthoDB^19^, confirms the expected position of Chelicerata as basal to crustaceans and insects (Fig. 3a). The rate of molecular evolution of *I. scapularis* genes is slightly slower than that of other representative arthropods, and considerably slower than the rapidly evolving dipterans. Quantification of shared intron positions (Fig. 3b) and lengths (Fig. 3c) among orthologs reveals that *I. scapularis* shares greater than 10 times more intron positions exclusively with the non-arthropod species compared with the crustacean *Daphnia pulex* (Supplementary Figs 11–14 and Supplementary Tables 7–10). The species tree topology is reconstructed using only intron presence/absence data, but its branch lengths reveal that *I. scapularis* intron positions are more similar to those of the outgroup species, than to the other arthropods. This distinction is underscored by the contrasting length distributions of shared introns; *I. scapularis* lengths are most similar to those of mouse and other vertebrates, and an order of magnitude greater than in *D. pulex* and the representative insect species analysed. Ancestral eukaryotic genes likely possessed high intron densities similar to those of modern mammals^20^. The tick genome, therefore, supports an intron-rich gene architecture at the base of the arthropod radiation and more similar to that of ancestral metazoans than extant pancrustaceans.

### Ticks as parasites

**Figure 1:**
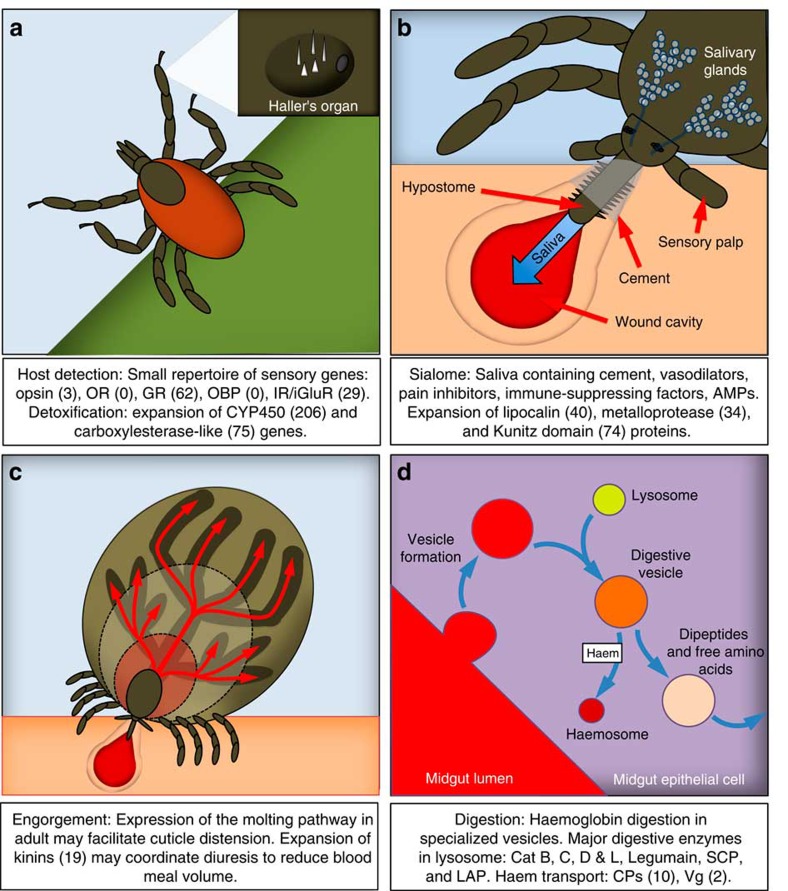
Genes associated with the unique parasitic lifestyle of *Ixodes scapularis*. (**a**) Host detection. Ticks spend long periods off-host and locate hosts by ‘questing' from vegetation. The Haller's organ, located on the first pair of tarsi, is the major sensory appendage. The tick has a relatively small repertoire of visual and chemosensory genes and an expansion of detoxification genes, presumably to counteract environmental toxicants. (**b**) Attachment and blood feeding. The tick creates a wound cavity and injects saliva containing cement, vasodilators, pain inhibitors, anticoagulants and immune-suppressing factors to facilitate long periods of attachment and blood feeding. (**c**) Engorgement. Blood engorgement takes place over days to weeks and includes slow and rapid phases (dotted lines indicate increase in body volume). New cuticle is putatively synthesized to accommodate ingestion of the large (∼100-fold increase in body weight) blood meal. The tick has an expansion of neuropeptide receptors to regulate diuresis and concentrate the blood meal. (**d**) Digestion. The processes of haemoglobin digestion in intracellular vesicles of midgut cells and haem sequestration involving specialized storage proteins are unique to ticks. Haemolyzed erythrocytes are absorbed by midgut epithelial cells by pinocytosis. Digestion is accomplished by fusion with lysosomes containing digestive enzymes (see text) and sequential breakdown of proteins (1) liberating haem and 8–11 kDa peptide fragments, (2) ∼5–7 kDa fragments, (3) 3–5 kDa peptides and finally (4) dipeptides and free amino acids. Amino acids are transcytosed from the digestive cells into haemolymph and haem is transported by haem-binding proteins to haemosomes for detoxification. Absorbed nutrients are converted to storage proteins (CP) throughout development or to vitellogenin in adult females for yolk provisioning of the egg just before oviposition. AMP, antimicrobial peptide; CAT, cathepsin; CP, haemlipoglyco-carrier protein; CYP450, cytochrome P450; GR, gustatory receptor; IR/iGluR, ionotropic receptor/ionotropic glutamate receptor; LAP, lysosomal aspartic protease; OBP, odorant binding protein; OR, odorant receptor; SCP, serine cysteine protease; Vg, vitellogenin.

**Figure 2:**
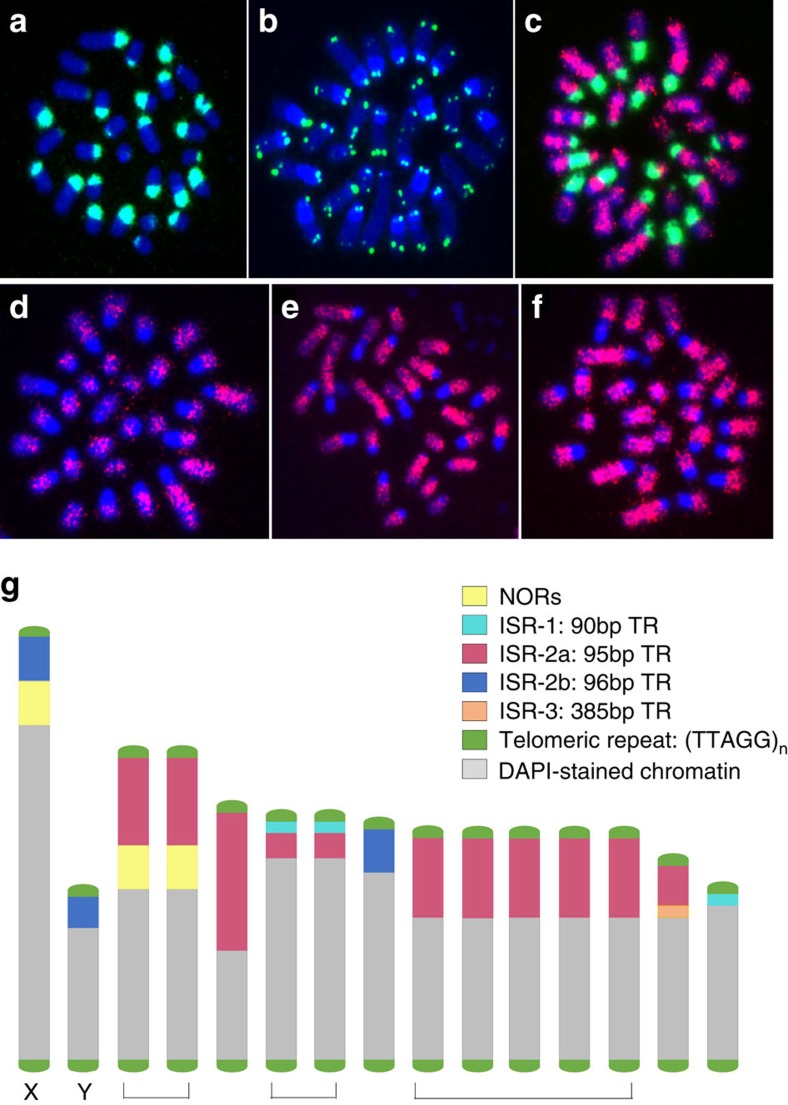
Organization of DNA on the *Ixodes scapularis* chromosomes. Families of tandem repeats (TRs) comprise approximately 40% of the genome and were localized by fluorescent *in situ* hybridization (FISH) to ISE18 cell line mitotic chromosome spreads. (**a**) Representative FISH image of C_o_*t*-1 DNA (green) at the heterochromatic terminal region of the DAPI-stained chromosomes (blue), presumed to represent the centromere. (**b**) Representative FISH of a telomeric repeat probe (TTAGG)_n_. Not all DAPI-stained chromosomes (blue) in this image show the ‘two-spot' telomeric hybridization signal (green) at both ends due to the limited depth of field possible during imaging. (**c**) Representative FISH of a BAC clone (BAC ID: 192414) in red and the ISR-2a 95 bp tandem repeat in green. BAC clone hybridization signals are dispersed throughout the presumed euchromatic regions of the DAPI-stained chromosomes (blue). Hybridization of the 95 bp tandem repeat is prevalent at one end of most of the chromosomes that is believed to represent the centromeres. (**d**–**f**) FISH using probes from clones in a small-insert gDNA library containing tandem repeats; Clone O-21 (**d**); Clone B-20 (**e**); Clone B-01 (**f**). Note that the hybridization signals (red) are dispersed among the presumed euchromatic regions of the DAPI-stained chromosomes (blue) and not at the heterochromatic termini thought to represent the centromeres. (**g**) Ideogram showing the relative arrangement of tandemly repetitive DNA based on FISH to the presumed acro- or telocentric chromosomes. The 13 autosomes and the X and Y sex determining chromosomes are shown. Brackets indicate groups of chromosomes sharing similar hybridization patterns. The individual chromosomes within these groups could not be distinguished based on relative size or distribution of tandemly repetitive DNA. Chromosomes are drawn to scale based on the representative example. Variability in the relative sizes of ISE18 chromosomes among different chromosome spreads prevented development of a standard karyotype where chromosomes are assigned numbers based on size and FISH marker distribution.

**Figure 3:**
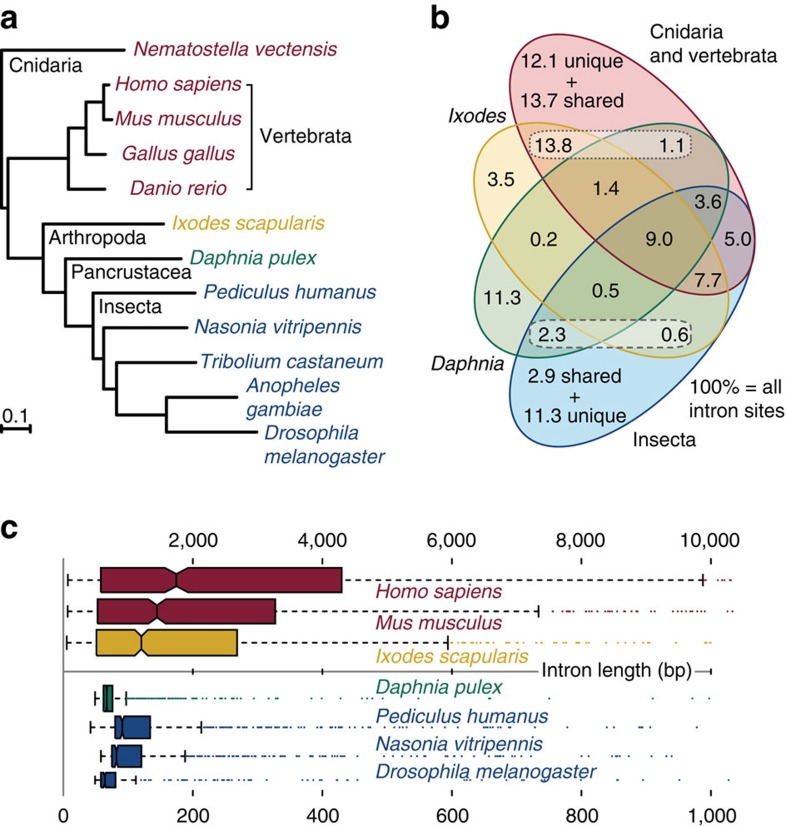
Molecular and intron evolution of *Ixodes scapularis* orthologs. (**a**) The species phylogeny computed from the concatenated alignment of single-copy orthologous protein-coding genes confirms the position of the Subphylum Chelicerata at the base of the arthropod radiation, an outgroup to the clade Pancrustacea that contains crustaceans and hexapods. The average rate of *I. scapularis* molecular evolution is slower than that in the fast-evolving dipterans (*Anopheles gambiae* and *Drosophila melanogaster)*, comparable to other representative arthropods for which genome sequences are available, and faster than that of vertebrates. (**b**) Quantification of the proportions of shared and unique intron positions from well-aligned regions of universal orthologs reveals that, compared with the crustacean, *Daphnia pulex*, *I. scapularis* shares more than 10 times as many introns exclusively with at least one of the five outgroup species (from Cnidaria and Vertebrata) (dotted box, 13.8% versus 1.1%). Conversely, *D. pulex* has more intron positions exclusively in common with the representative insects (dashed box, 2.3% versus 0.6%). (**c**) *I. scapularis* intron lengths are more similar to those of introns from orthologous genes in the vertebrates *Homo sapiens* and *Mus musculus*, and are an order of magnitude longer than introns from the pancrustacean species analysed. The intron length distributions are shown for ancient introns found in both *I. scapularis* and *D. pulex* and at least one of the five outgroup species and at least one insect; boxplots indicate medians, first and third quartiles, and whiskers.

Tick mouthparts (chelicerae and barbed hypostome) attach to and create a feeding lesion in the dermis of the host (Fig. 1b). Tick saliva consists of a complex mixture of peptides and other compounds that facilitate attachment and disarm host haemostasis, inflammation and immunity, thereby enabling prolonged blood feeding. Antimicrobials in the saliva^21^ presumably prevent bacterial overgrowth within the ingested blood and/or feeding lesion. Transcriptome analyses indicate that tick saliva is exceptionally diverse compared with that of haematophagous insects^22^. Also, genes encoding salivary gland products are evolving rapidly in comparison with other gene families, possibly due to the immune pressure imposed by the host. Notably, the genome reveals an expanded repertoire (74, 0.4% of the predicted proteome) of proteins containing a Kunitz domain (Supplementary Table 16), implicated in protease inhibition and channel-blocking activity, with roles in inhibiting coagulation, angiogenesis and vasodilation. The tick genome is the richest source of this gene family identified to date. In contrast, only 0.05% of human and 0.1% of bovine proteins have this signature domain^23^, while the mosquito vectors *Aedes aegypti, Culex quinquefasciatus* and *Anopheles gambiae* have only five, eight and four proteins with this domain, respectively. Other tick gene expansions of note include the lipocalins (40 genes), linked to anti-inflammatory activity in other systems^24^, and the metalloproteases (34 genes), which are involved in fibrin degradation and inhibition of angiogenesis^25^

Ticks have evolved a novel mechanism for haemoglobin digestion. Haemolysis of host erythrocytes occurs in the midgut but the digestion of blood meal proteins takes place within specialized vesicles of midgut epithelial cells following internalization by pinocytosis (Fig. 1d). Haemoglobin digestion occurs via a cascade of proteolytic enzymes resulting in dipeptides and free amino acids that are transcytosed into the haemolymph (Supplementary Text and Supplementary Table 21). Orthologs of *Ixodes ricinus* haemoglobinolytic enzymes^26^ were identified in the *I. scapularis* genome that contains multiple genes for *cathepsin D* (three genes), *cathepsin L* (three genes), and *serine carboxypeptidase* (four genes), suggesting the relative importance of these enzymes in haemoglobin digestion. Haemoglobinolytic enzymes have also been identified in other tick species^27^,^28^, suggesting that this mode of haemoglobin digestion is widespread throughout the Ixodida. Liberated haem is transported from the digestive vesicles by transport proteins to haemosomes, unique storage vesicles where haem is detoxified by formation of haematin-like aggregates^29^. Thus, haemoglobinolysis in ticks is similar to that in endoparasitic flatworms and nematodes. However, tick-specific intracellular digestion in midgut epithelial vesicles and haem detoxification in specialized haemosomes could offer novel acaricide targets (Supplementary Text and Supplementary Table 21).

**Figure 4:**
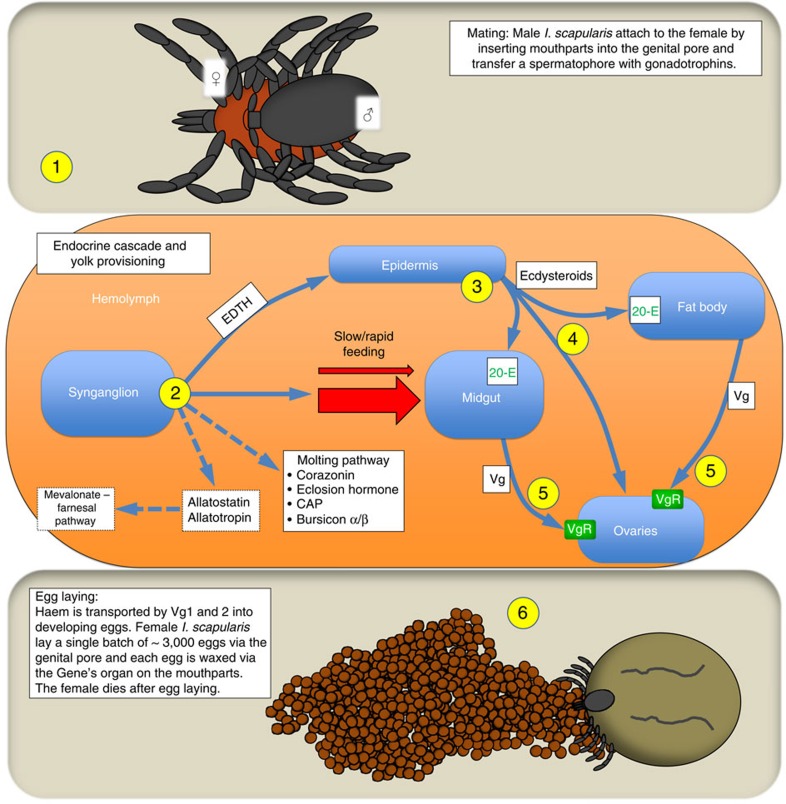
Model of neuroendocrine processes controlling mating and egg production in *Ixodes scapularis*. (1) Mating takes place off or on the host (before or during blood feeding), but is required for rapid blood feeding. The male attaches to the genital pore of the female via its mouthparts (evidence suggests the potential involvement of female specific cuticular lipids and a non-volatile mounting pheromone in *I. scapularis*), then transfers sperm and gonadotropins (unidentified at present), among other seminal components, including the spermatophore, (2) Gonadotropins initiate the synganglion to release EDTH, stimulate rapid engorgement, initiate synthesis of neuropeptides which in insects regulate moulting and synthesis of new cuticle (tick functions unknown), and release of allatostatins and allatotropins (which may stimulate or inhibit the mevalonate-farnesal pathway), (3) EDTH initiates production of ecdysteroids by the epidermis, (4) High ecdysteroid titres activate transcription factors for VgR in the ovaries, are stored in developing eggs and, as 20-E, activates transcription factors for Vg in the fat body and specialized cells of the midgut, (5) Vg is taken up via VgR-receptor mediated endocytosis by developing oocytes and incorporated into the yolk as vitellin, and (6) The female produces a single batch of ∼3,000 mature eggs from the genital pore that are passed forward to the mouthparts for coating with wax released from the Gene's organ. Biochemical and genomic evidence suggests that *I. scapularis* do not make JH III although the genes for the preceding mevalonate and parts of the farnesal pathway were identified. Dashed lines indicate proposed pathways and factors. 20-E, 20-hydroxyecdysone; CAP, cardioactive peptide; EDTH, hypothesized epidermal trophic hormone; Vg, vitellogenin (yolk protein in haemolymph before egg uptake); VgR, vitellogenin receptor.

Haem is associated with multiple essential functions as it complexes with proteins that perform oxygen transport and sensing, enzyme catalysis and electron transfer^30^. However, ticks are incapable of *de novo* haem synthesis, and it has been proposed that they rely on haem recovery from the diet^31^. The identification of orthologous genes in *I. scapularis* for the enzymes hemF, hemG and hemH associated with the production of protohaem (Supplementary Fig. 15 and Supplementary Table 20) suggests these may be remnants of a once functional haem synthesis pathway that became redundant following adaptation to a blood diet. In the absence of *de novo* synthesis, haem storage in ticks is likely essential, especially during the extended periods that occur between blood feeding and during egg development. In ticks, two families of storage proteins ensure haem availability and protect against the toxicity of a haem-rich diet: haemlipoglyco-carrier proteins (CPs) and the yolk proteins, vitellogenins (Vgs)^32^ (Fig. 1d). CPs are predominant in all tick developmental stages except the embryo. In contrast, Vg is produced in the fat body and midgut of adult females during vitellogenesis (Fig. 4), and is transported via the haemolymph to the developing oocyctes where it is stored as vitellin. Vitellin is the main protein in the egg and the likely source of haem for developing embryos^33^. Ten putative *CP* genes, the most described from a tick to date, and two *Vg* genes were identified in the *I. scapularis* genome (Supplementary Fig. 16 and Supplementary Table 22).

The genome contains orthologs for at least 39 invertebrate neuropeptide genes (Supplementary Tables 25–28), including peptides that regulate ecdysis, cuticle synthesis, hardening and tanning. Orthologs involved in insect moulting^34^, that is, corazonin, eclosion hormone, cardioactive peptide and buriscon α and β, were identified (Fig. 4). Additional novel putative neuropeptide genes were identified based on the presence of tandem repeats in conserved C-terminal sequences, including the canonical sequences for amidation and dibasic (or monobasic) cleavage signals (Supplementary Table 25). ESTs matching corazonin, eclosion hormone and bursicon α and β were found in the synganglion transcriptome of adult *Dermacentor variabilis*^35^, which do not moult, suggesting previously unrecognized roles for these neuropeptide hormones. Companion analyses^36^ identified major differences in gene expression between *I. scapularis* and the soft tick, *Ornithodoros turicata* (Argasidae) in response to feeding that may explain how synganglion neuropetides regulate different life styles of the two tick families. The identification of orthologs of neuropeptides known to regulate insect moulting provides a much needed starting point to understand the regulation of development in ticks and in the modification of cuticle to accommodate the approximately 100-fold increase in size that occurs during blood feeding (Fig. 4).

In ticks, over-hydration from large blood meals is counterbalanced by hormonally controlled salivary secretion into the host, presumably regulated by neuropeptides and their G-protein-coupled receptors (GPCRs) (Fig. 1c). The homologs of many insect neuropeptides, protein hormones, biogenic amines and associated GPCRs^37^ (Supplementary Tables 25–28) that steer processes such as diuresis, behaviour, reproduction and development^38^, were identified in *I. scapularis*. Some of the neuropeptide genes identified encode multiple neuropeptides. Of note is the extreme number of copies (19) of the kinin gene, which ranges from one to eight in other arthropods^38^ (Supplementary Table 28), suggesting that high peptide copy number is also needed for effective diuresis. In accordance, four kinin GPCRs are present (Supplementary Table 28). The tick has 20 GPCRs for five biogenic amines, a number similar to that for all other sequenced arthropods^37^, suggesting an early evolutionary origin of these molecules and a core set of highly conserved arthropod signalling molecules. Typically in insects, each neuropeptide interacts with one, or at most two, GPCRs^37^. Remarkably, the numbers of some neuropeptide GPCRs have expanded significantly (up to 10-fold) in *I. scapularis* (Supplementary Tables 26 and 28). This includes the GPCRs for AKH/corazonin-related peptide, allatostatin-A, diuretic hormones (calcitonin- and CRF-like), inotocin, kinin, pigment-dispersing-factor, sulfakinin, and tachykinin (Supplementary Table 28)^37^. In insects, these GPCRs are involved in regulating meal size (kinin), satiety (sulfakinin) and diuresis (kinin, tachykinin and calcitonin-like diuretic hormone)^38^. In ticks, the increased efficacy and fine regulation of diuresis may be accomplished through an increased repertoire of diuretic GPCRs rather than via corresponding neuropeptides, emphasizing their potential as targets for tick control.

Blood feeding is essential for reproduction in adult female ticks (Fig. 4). In lower insects, reproduction is largely regulated by juvenile hormone III. Biochemical evidence suggests that ticks do not synthesize juvenile hormone III and instead employ ecdysteroids to initiate vitellogenesis (Fig. 4, reviewed in^33^). In insects, the final hydroxylations for the synthesis of ecdysteroids are performed sequentially by cytochrome P450s (CYP450s) encoded by the *Halloween* genes (Supplementary Fig. 17 and Supplementary Table 19). Genes for all steroidogenic CYP450s except for *phantom* were identified in the *I. scapularis* genome and putative gene duplications were identified for *disembodied* and the *spook/spookier* clades, suggesting conservation of ecdysteroid regulated processes between ticks and insects. Genes for seven of the nine enzymes in the insect mevalonate pathway that produces the juvenile hormone precursor, farnesyl-pyrophosphate (farnesyl-PP), were identified in the tick genome (Supplementary Fig. 18 and Supplementary Table 18). There are five insect enzymes involved in the conversion of farnesyl-PP to juvenile hormone III. Only the gene for farnesol oxidase in the juvenile hormone branch was found in the *I. scapularis* genome (Supplementary Table 18) and is transcribed in the synganglion of *I. scapularis* and *D. variabilis*. The tick genome reveals a striking expansion of the methyl transferase family (44 genes) and EST data indicate that at least 26 of these are transcribed (Supplementary Fig. 19). However, the *I. scapularis* methyl transferases studied so far lack the juvenile hormone binding motif. An ortholog of the insect cytochrome P450 (CYP15A1) that adds the epoxide to methyl farnesoate to produce juvenile hormone III was not found in either the tick genome (Supplementary Table 18) or synganglion transcriptomes. The neuropeptides, allatostatin and allatotropin, which perform a variety of functions in insects, including the regulation of juvenile hormone biosynthesis, were also identified in the tick (Fig. 4). Important questions remain as to the role of the mevalonate-farnesal pathway in tick reproduction and development. In a complementary study, transcripts for genes in the mevalonate-farnesal pathway were identified from the synganglion of two hard and one soft tick species^39^.

The *I. scapularis* genome reflects a parasitic lifestyle requiring detoxification of multiple xenobiotic factors (Fig. 1a). We identified a record 206 CYP450 (Supplementary Table 23) and 75 carboxylesterase/cholinesterase-like genes, including five putative acetylcholinesterase genes (Supplementary Table 24). CYPs are haem-containing enzymes that catalyse biological oxidation reactions, many of which detoxify xenobiotics, including acaricides. In contrast, the body louse, *Pediculus humanus*, also an obligate blood-feeding ectoparasite, has 36 CYPs, the fewest known in an animal^40^, while the plant feeding mite, *T. urticae* has 81 (ref. 9). Carboxylesterases are also associated with metabolic detoxification in animals. While the function of these enzymes is not known, the abundance of these genes in *I. scapularis* may reflect the need to detoxify large blood meals from diverse hosts and toxicants encountered during off-host stages.

As a parasite that lives largely off-host, *I. scapularis* has developed unique mechanisms for host detection that are reflected in the genome (Fig. 1a). The sensory system in ticks includes setiform sensilla for chemo-, mechano-, thermo- and hygroreception, non-setal sensilla and dorsal light-sensing cells. Chemoreception occurs presumably through the unique Haller's organ located on the tarsi that are presented when ticks ‘quest' for a host. In insects, smell and taste are mediated by families of membrane receptors and extracellular ligand-binding proteins^41^. The chemoreceptor genes identified in the tick genome belong to the gustatory receptor and ionotropic glutamate receptor (iGluR)-related ionotropic receptor families. Sixty-two gustatory receptors were identified that fall into three major clades (Supplementary Fig. 20, Supplementary Table 29 and Supplementary Note 1). The largest of the clades (43 genes) is exclusive to *I. scapularis* and the relatively short branch lengths compared with those for other representative species, suggest a recent lineage-specific expansion. Although phylogenetically distant, this clade is related to the Dipteran sugar receptors and a set of three distinctive *D. pulex* gustatory receptors^42^. The second clade includes 16 tick gustatory receptors, also more closely related to the sugar receptors than to other representative gustatory receptors, with branch lengths suggesting an early diversification. The remaining clade (three genes) clusters with the largest *D. pulex* expansion. Of the 29 *IR/iGluR* genes identified, 15 are likely of the chemosensory type (ionotropic receptor) and 14 are canonical iGluRs (Supplementary Fig. 21 and Supplementary Tables 30 and 31). Members of the insect odorant receptor, odorant-binding protein (OBP) and chemosensory protein B families^43^ were not identified in the tick and only one member of the chemosensory protein (CSP) family was found. Our analysis supports the hypothesis that the origin of insect odorant receptors and OBPs occurred after the split of the lineages Hexapoda and Crustacea (∼470 Myr ago)^42^,^44^; the CSPs, however, are predicted to appear before the split of the Chelicerata and Pancrustacea lineages. Phylogenetic analyses indicate that odorant receptors belong to a divergent lineage originated from gustatory receptors, while OBPs could have derived from a CSP-like ancestor^44^. Both events may have occurred concomitantly as an adaptation of ancestral hexapods to the terrestrial environment (380–450 Myr ago). Chelicerate olfaction may, therefore, rely exclusively on ionotropic receptors, which are expressed in olfactory organs across Protostomia^45^, although it is also possible that some gustatory receptors have been recruited to this sensory function, as in *Drosophila melanogaster*^46^. Comparative transcriptomics has identified putative GPCRs, ionotropic receptors, odorant turnover enzymes and other transcripts specific to the Haller's organ in ticks^47^. Evidence suggests the potential involvement of female specific cuticular lipids and a non-volatile mounting pheromone in *I. scapularis* during mating^48^. These data and morphological studies provide an emerging model for research on tick chemical communication and new control methods.

The tick possesses a small repertoire of photon-sensitive receptors compared with most insects. Genes for three opsin GPCRs were identified (Fig. 1a, Supplementary Table 26) and include orthologs of the insect putative long-wavelength sensitive ‘visual' opsins, the honey bee ‘non-visual' pteropsin likely involved in extraocular light detection and regulation of circadian rhythm^49^, as well as the *D. melanogaster* Rh7 opsin^50^. Orthologs of the insect UV and short wavelength receptors were not identified. This indicates a reduced visual system as compared with other blood-feeding arthropods (Supplementary Text) that rely heavily on visual processes during flight for location of mates, hosts and oviposition sites. During host detection, olfactory, mechano- and thermoreception may offset limited visual acuity and wavelength detection in the tick.

### Ticks as vectors of pathogens and parasites

**Figure 5:**
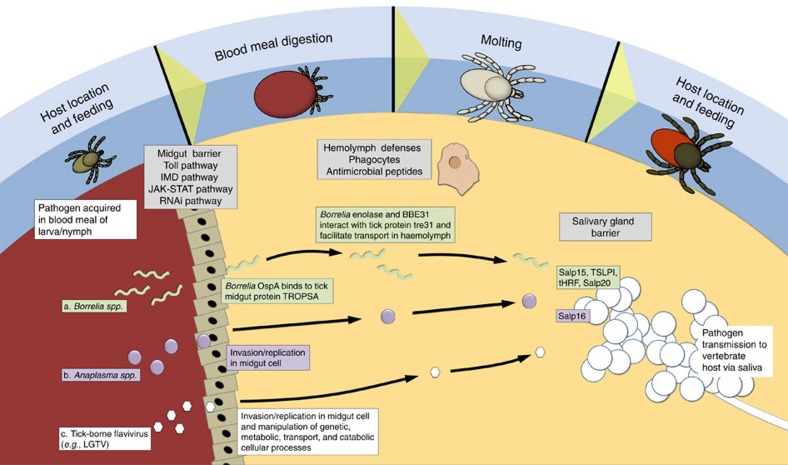
Key features of pathogen transmission by *Ixodes scapularis*. The tick life stages involved in the transmission of a typical pathogen (outer ring) and critical physical/physiological barriers to pathogen acquisition, replication and transmission (inner circle) are depicted. Representative pathogens: (a) *Borrelia spp.*; (b) *Anaplasma phagocytophilum;* (c) Tick-borne flavivirus (e.g., Langat virus, LGTV). The different strategies employed by a parasite to navigate from the midgut to the salivary glands, and tick and parasite derived factors known to facilitate these processes are shown. IMD, Immunodeficiency; JAK-STAT, Janus Kinase/Signal Transsducers and Activators of Transcription; OspA, *Borrelia* outer surface protein A; Salp15/16, salivary gland protein 15/16; tHRF, tick histamine-release factor; TROPSA, tick receptor for OspA; TSLP1, tick salivary lectin pathway inhibitor.

Ticks are biological vectors of viruses, bacteria and protozoa that are typically acquired via the blood meal and transmitted through saliva during feeding (Fig. 5). The tick immune system has several mechanisms to fend off pathogen invasion. Most components of the Toll, IMD (Immunodeficiency), JAK-STAT (Janus Kinase/Signal Transducers and Activators of Transcription) immune pathways and the RNA interference-antiviral signalling pathways were identified in the tick genome (Supplementary Figs 22 and 23 and Supplementary Table 17). The repertoire of immunity-related genes also includes akirins, antimicrobial peptides, caspases, defensins, oxidases, the fibrinogen-related protein family of ixoderins, lysozymes, thio-ester containing proteins and peptidoglycan-recognition proteins (Supplementary Table 17).

Multiple infection factors facilitate transmission of the Lyme disease pathogen, *Borrelia burgdorferi* (Fig. 5). These include the tick salivary gland proteins Salp15, Salp20, Salp25D, tick salivary lectin pathway inhibitor and tick histamine-release factor, as well as the tick receptor for OspA and tick protein tre31, and the *Borrelia* lipoprotein BBE31 (ref. 51). Increasingly, research is focused on interactions with *Anaplasma phagocytophilum* (Rickettsiales: Anaplasmataceae), the causative agent of human granulocytic anaplasmosis prevalent in the USA and Europe^52^. The *I. scapularis* proteins P11, SALP16, α1, 3-fucosyltransferases and the X-linked inhibitor of apoptosis E3 ubiquitin ligase are required for *A. phagocytophilum* infection and transmission, and modification of the tick cytoskeleton by *A. phagocytophilum* increases infection^53^,^54^,^55^. To establish infection, *A. phagocytophilum* inhibits apoptosis in midgut and salivary gland cells through the JAK/STAT and intrinsic pathways^56^. In response, the extrinsic apoptosis pathway is induced in tick salivary glands. All known components of these pathways were identified in the tick with the exception of the Perforin ortholog (Supplementary Table 17). Systems biology analyses^56^ revealed that the generalized responses of tick cells to *A. phagocytophilum* infection include changes in protein processing in the endoplasmic reticulum and glucose metabolism. Protein misfolding is increased in infected tick cells, a possible strategy by which *A. phagocytophilum* evades the cellular response to infection. The subsequent activation of protein targeting and degradation, reduces endoplasmic reticulum stress and prevents cell apoptosis, and may also benefit the pathogen through provision of raw materials critical for an obligatory intracellular parasite with reduced biosynthetic and metabolic capacity^57^. In addition, *A. phagocytophilum* can induce an increase in expression of antifreeze glycoproteins, enhancing *I. scapularis* survival in cold temperatures^58^, and downregulate Porin expression to inhibit apoptosis, increasing tick colonization^55^,^56^. Tick cells respond to pathogen infection by decreasing glucose metabolism and increasing Subolesin and Heat Shock Protein expression, and limiting rickettsial infection^59^,^60^.

We used quantitative proteomics to further characterize tick–*Anaplasma* interactions, and identify differential protein expression in an *I. scapularis* ISE6 cell line in response to infection; 735 unique peptides assigned to 424 different *I. scapularis* proteins, were identified (Supplementary Tables 32–35). In total, 83 proteins were differentially represented (50 under- and 33 over-represented; Supplementary Fig. 24 and Supplementary Table 32). Under-represented (13) and over-represented (8) proteins were identified during early infection (11–17% infected cells at 3 days post-inoculation). Most were also represented as infection advanced when the number of under- and over-represented proteins increased to 50 and 31, respectively (56–61% infected cells; 10 days post-inoculation). Analysis of protein ontology demonstrated differences between under- and over-represented proteins in both early and late infections for cell growth (adducin, spectrin and β-tubulin) and transport (Na^+^/K^+^ ATPase, voltage-dependent anion-selective channel or mitochondrial porin and fatty acid-binding protein; Supplementary Tables 32–34).

The genome of a *Rickettsia* (Alphaproteobacteria: Rickettsiales) species, *Rickettsia* endosymbiont of *Ixodes scapularis* (REIS), was assembled from both bacterial artificial chromosome clones and recruited whole-genome shotgun reads (available at GenBank, NZ_ACLC00000000). Phylogenomics analysis of the REIS genome, which comprises a single 1.82 Mbp chromosome and four plasmids, indicates a novel non-pathogenic species that is ancestral to all Spotted Fever Group *Rickettsia* species, providing a valuable resource for understanding the evolution of symbiosis versus pathogenicity^61^.

Much less is known about the molecular mechanisms involved with viral interactions in ticks. Research suggests the RNA interference pathway provides an important defense against virus infection in tick cells, with a significant expansion of *Ago* genes in comparison with insects^62^. In a companion proteomics study of the *I. scapularis* ISE6 cell line following infection with the Langat virus^63^, 266 differentially expressed tick proteins were identified. Functional analyses suggest perturbations in transcription, translation and protein processing, carbohydrate and amino acid metabolism, transport and catabolism responses. The majority of differentially expressed proteins were downregulated, similar to the proteomics profile described above. Interestingly, 121 differentially expressed proteins lacked homology to known orthologs, suggesting these may be unique to *I. scapularis*.

### Population structure of *Ixodes scapularis* in North America

The restriction-site-associated DNA sequencing (RADseq) technique was employed for genome-wide discovery of single-nucleotide polymorphisms (SNPs) and examination of genetic diversity within and among eight *I. scapularis* populations from the north-east, mid-west and south-east regions of the USA and the Wikel reference colony. F-statistics were used to assess genetic distance as evidence of selection. F_IS_ values (range 0.003–0.012; Supplementary Table 36) suggest random mating or low levels of inbreeding among members comprising each population. Further supporting this hypothesis, among all populations, the average observed heterozygosity (Ho) per variable SNP was comparable (range 0.013–0.016) to expected heterozygosity (He) (range 0.013–0.018) and the nucleotide diversity over all SNP loci (π) (range 0.015–0.019) was comparable among samples. F_ST_ values (range 0.03–0.16; Supplementary Table 37), support a single species classification for *I. scapularis* across North America as previously reported^64^. Low-moderate genetic variation (F_ST_=0.03–0.06) was observed among northern tick populations from Indiana, Maine, Massachusetts, New Hampshire and Wisconsin, and moderate variation (F_ST_=0.07–0.09) among southern populations from Florida, North Carolina and Virginia. F_ST_ analyses revealed signatures of north–south structure in *I. scapularis* populations. Moderate-to-high genetic variation was observed between northern versus southern populations (F_ST_=0.10–0.15). Interestingly, low genetic variation (F_ST_=0.03–0.06) was observed between populations from the mid-west (Indiana and Wisconsin) versus the north-east (Maine, Massachusetts and New Hampshire), two areas associated with a high prevalence of human Lyme disease cases. As expected, moderate-to-high genetic variation was observed between the reference Wikel colony and field populations (F_ST_=0.07–0.16).

**Figure 6:**
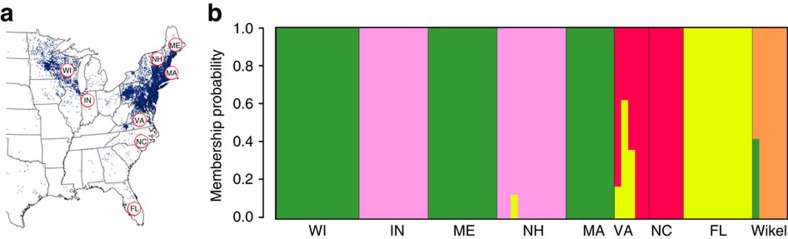
Population structure of *Ixodes scapularis* across North America. (**a**) Tick sampling sites in Indiana (IN), Massachusetts (MA), Maine (ME), North Carolina (NC), New Hampshire (NH), Wisconsin (WI), Florida (FL) and Virginia (VA) overlaid against reported Lyme disease cases in 2012 (modified from CDC: http://www.cdc.gov/lyme/stats/maps/map2012.html); (**b**) Membership probabilities in bar plots for individual *I. scapularis* comprising different clusters and showing separation of genetic groups based on 34,693 RADtag SNP markers. SNP, single-nucleotide polymorphism; WK, *I. scapularis* WIKEL reference strain.

The population structure of *I. scapularis* was separately analysed using a subset of representative SNPs. Membership probabilities, interpreted as proximities of individuals belonging to each cluster, revealed five clades (Fig. 6), with clear separation of the Wikel colony from field populations. Clustering of Indiana and New Hampshire, and Massachusetts, Maine and Wisconsin populations, indicates significant shared alleles, while the Virginia, Florida and North Carolina populations may share a small number of alleles. Interestingly, the population structure suggests a genetic component associated with differences in the natural history of northern and southern *I. scapularis* and a correlation to the prevalence of human Lyme disease cases. The incidence of Lyme disease is greatest in the upper mid-west and north-east where *I. scapularis* populations feed predominantly on deer as adults and complete the life cycle over 2 years. In contrast, southern populations exploit a wider range of vertebrate hosts and are not quiescent during winter^64^,^65^. These data provide important resources to determine the genetic basis of host preference and vector competence, and the correlation with Lyme disease transmission.

### Genome-based interventions to control tick-borne disease

**Figure 7:**
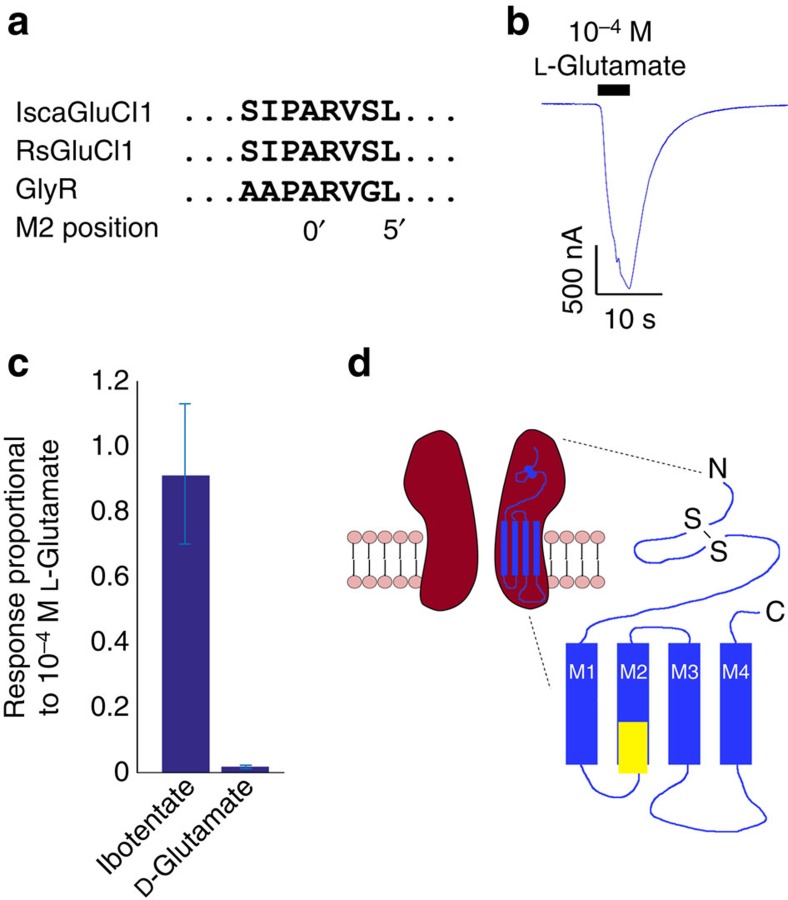
De-orphanizing *Ixodes scapularis* receptors as candidate targets for the development of new acaricides. The newly identified *I. scapularis* dicysteine-loop, ligand-gated anion channel subunit (IscaGluCl1, KR107244) contains the ‘PAR' motif centred on the 0' position in the second transmembrane region (TM2), characteristic of ligand-gated anion channels and is aligned with a brown dog tick *Rhipicephalus sanguineus* GluCl (RsGluCl1, ACX33155) and human glycine receptor α-subunit (P23415) (**a**). Using the *Xenopus laevis* oocyte receptor expression vehicle, IscaGluCl1 yielded robust chloride currents in response to 10^−4^ M L-glutamate (**b**) and ibotenate (**c**) but only weak currents in response to the same concentration of D-glutamate (**c**); ibotentate and D-glutamate responses are depicted relative to L-glutamate (*n*=6, 8; error bars represent±1 s.e.m.). No response was detected in the presence of 10^−4^ M acetylcholine (ACh), γ-amino butyric acid (GABA), dopamine, histamine, serotonin, tyramine or glycine. The subunit is therefore identified as an *Ixodes scapularis* homomer-forming GluCl subunit, (IscaGluCl1), illustrated by a schematic of a homomeric GluCl showing two of the five subunits and the position of the PAR motif in yellow (**d**).

Prevailing methods of tick control rely heavily on the use of repellents and acaricides. Resistance to currently applied pesticides that disrupt neural signalling and tick development has prompted the search for novel targets. GPCRs represent a source of candidate targets for development of novel interventions. High-throughput target-based approaches have been employed to discover new mode-of-action chemistries that selectively inhibit the *I. scapularis* dopamine receptors^66^. The ligand-gated ion channels (LGICs) offer another rich source of targets. iGluRs play a major role in neurotransmission and chemosensory signalling within arthropods^67^. Twenty-nine putative *iGluR* genes and 32 putative cys-loop receptors were identified in the *I. scapularis* genome (Fig. 7, Supplementary Table 31). Among the *iGluR* genes, 14 encode members of the three principal subclasses of synaptic iGluRs (AMPA, Kainate and NMDA; Supplementary Fig. 21 and Supplementary Tables 30 and 31), while the remaining 15 more divergent sequences likely belong to the chemosensory ionotropic receptor subfamily (see above). The cys-loop LGIC family also contains six candidate glutamate-gated Cl^−^ channels (GluCls), 12 nicotinic acetylcholine receptor subunits, and four GABA-gated chloride channels. One histamine-gated Cl^−^ channel and one pH-gated Cl^−^ channel gene were also identified. Both the iGluRs and cys-loop LGIC families contain tick-specific genes with no apparent insect ortholog. This striking divergence may contribute to the apparent ineffectiveness of some insecticides on acaricidal targets^67^. Classifying LGIC candidates by functional expression is underway and an example is shown for a GluCl (Fig. 7; Supplementary Fig. 25). Selective targeting of tick LGICs and GPCRs may offer routes to new, safe and effective acaricides.

## Discussion

The genome sequence of *I. scapularis*, the first for a medically important chelicerate, offers insights into the molecular processes that underpin the remarkable parasitic lifestyle of the tick and its success as a vector of multiple disease-causing organisms. Foundational studies of genome organization and population structure will advance research to determine the genetic basis of tick phenotypes, and efforts are ongoing to discover novel chemistries that selectively disrupt molecular targets mined from the genome. This study is a pioneering project for genome research on ticks and mites of public health and veterinary importance, with efforts proposed to expand genomic resources across this phyletic group. In 2011, the National Institutes of Health approved the sequencing of additional species of hard ticks, including European and Asian *Ixodes* species, the soft tick *Ornithodoros moubata* (Family Argasidae) and the *Leptotrombidium* mite vector of scrub typhus (Superorder Acariformes)^68^ (Supplementary Table 38). The *I. scapularis* genome offers a roadmap for research on tick–host–pathogen interactions to achieve the goals of the One Health Initiative^69^ and improve human, animal and ecosystem health on a global scale.

## Methods

### Genome sequencing, assembly and annotation

The genome of *I. scapularis* Wikel strain was sequenced in a joint effort by the Broad Institute and the JCVI and funded by the National Institute of Allergy and Infectious Diseases, National Institutes of Health. The *I. scapularis* Wikel strain (Quinnipiac University, Hamden, CT) genome was sequenced to approximately 3.8-fold coverage using Sanger sequencing and assembled using the Celera Assembler configured to accommodate high repeat content within the genome and heterozygosity in the donor population (Supplementary Table 1). The assembly and raw reads are available at GenBank under the project accession ABJB010000000, consisting of contig accessions ABJB010000001-ABJB011141594 and VectorBase as IscaW1, 3 May 2012. The annotation of the *I. scapularis* genome was performed via a joint effort between the JCVI and VectorBase. The genome annotation release (IscaW1.4) is available at VectorBase (https://www.vectorbase.org/) and GenBank (accession ID: ABJB010000000). Forty-five bacterial artificial chromosome clones, ∼183,834 ESTs and 45 microRNAs were also sequenced and annotated (Supplementary Figs 4–6 and Supplementary Tables 4–6).

### Proteomics of *Ixodes*-*Anaplasma* interactions

The *I. scapularis* ISE6 cells were inoculated with *A. phagocytophilum* (human NY18 isolate) or left uninfected. Uninfected and infected cultures (*n*=5 independent cultures each) were sampled at early infection (11–17% infected cells (Avg±s.d., 13±2)) and late infection (56–61% infected cells (Avg±s.d., 58±2)) and used for proteomics. Protein extracts from the four experimental conditions, control uninfected early, infected early, control uninfected late and infected late (100 μg each) were gel-concentrated, digested overnight at 37 °C with 60 ng μl^−1^ trypsin (Promega, Madison, WI, USA) and the resulting tryptic peptides from each proteome were extracted and iTRAQ labelled for the analysis. The samples were fractionated by isoelectric focusing and each fraction analysed by liquid chromatography-mass spectrometry/mass spectrometry (LC-MS/MS) using a Surveyor LC system coupled to a linear ion trap mass spectrometer model LTQ (Thermo Finnigan, San Jose, CA, USA) and protein identification was carried out using SEQUEST algorithm (Bioworks 3.2 package, Thermo Finnigan), allowing optional (Methionine oxidation) and fixed modifications (Cysteine carboxamidomethylation, Lysine and N-terminal modification of +144.1020 Da). The MS/MS raw files were searched against the alphaproteobacteria combined with the arachnida Swissprot database (Uniprot release 15.5, 7 July 2009) supplemented with porcine trypsin and human keratins. This joint database contains 638,408 protein sequences. False discovery rate of identification was controlled by searching the same collections of MS/MS spectra against inverted databases constructed from the same target databases. The alphaproteobacteria Swissprot database was used to identify *Anaplasma* and discard possible symbiotic bacterial sequences from further analyses.

### *Ixodes scapularis* genetic diversity and population structure

74 RADseq libraries were produced from female *I. scapularis* representing nine ‘populations' from the states of Florida, Indiana, Maine, Massachusetts, North Carolina, New Hampshire, Virginia and Wisconsin and the Wikel reference colony. RADseq libraries were constructed using 1 μg genomic DNA from individual ticks, separately digested with the *Sbf*I restriction enzyme. Adaptor ligated libraries were pooled and sequenced at the Purdue Genomics Core Facility on the Illumina HiSeq 2500 in Rapid run mode. Further analysis was performed by the Bioinformatics Core at Purdue University. Illumina reads were corrected for restriction site, clustered and de-multiplexed (sorted by barcode) using the ‘process_radtags.pl' script of STACKS. For SNP identification, reads from each sample were separately aligned to the IscaW1 assembly using the end-to-end mode and default parameters of Bowtie2 v 2.1.0. Genetic diversity within and between *I. scapularis* populations was calculated using 745,760 SNPs across 35,460 polymorphic loci. F-statistics were used to assess genetic distance or differentiation as evidence of selection where F_IS_ is the inbreeding coefficient of an individual (I) relative to the subpopulation (S) and F_ST_ is the difference in allele frequency between subpopulations (S) compared with the total population (T). The population structure of *I. scapularis* across North America was separately analysed using a subset of 34,693 representative SNPs (1 SNP per polymorphic locus). The ‘population' step from STACKS was used to analyse genetic diversity and fastStructure (beta release) was used to analyse population structure. Detailed methods are available in Supplementary Text. All variation data are available at NCBI SRA (SRP065406), VectorBase and via BioMart: http://biomart.vectorbase.org.

### Functional expression of tick LGICs

Expression studies were performed on mature oocytes extracted from anaesthetised female *Xenopus laevis*. Briefly, complementary RNA encoding IscaGluCl1 was injected at 1 mg ml^−1^ using a Drummond Nanoject injector into oocytes that had been treated for 20–40 min in a 2 mg ml^−1^ solution of collagenase type 1A (Sigma UK) in calcium-free saline. Following 3–5 days incubation at 18 °C in saline supplemented with penicillin (100 units per ml), streptomycin (100 μg ml^−1^), gentamycin (50 μg ml^−1^) and 2.5 mM sodium pyruvate, oocytes were secured individually in a Perspex chamber (∼90 μl) and perfused continually in saline at 5 ml min^−1^. They were impaled by two glass microelectrodes filled with 3 M KCl (resistance 1–5 MOhm in saline), with which the oocytes were voltage clamped at −100 mV using an Axoclamp 2A amplifier. Solutions were applied in the perfusing saline. The saline consisted of (in mM): NaCl 100, KCl 2, CaCl_2_ 1.8, MgCl_2_ 1, HEPES 5, adjusted to pH 7.6 with 10 M NaOH.

## Additional information

**Accession codes:** The data reported in this paper are archived at GenBank under the project accession ABJB010000000, consisting of contig accessions ABJB010000001-ABJB011141594, and at VectorBase (IscaW1, 3 May 2012). The genome annotation release (IscaW1.4) is available at GenBank (accession ID: ABJB010000000) and VectorBase (https://www.vectorbase.org/) and RADseq data have been deposited in the NCBI Sequence Read Archive (SRA) under accession code SRP065406.

**How to cite this article:** Gulia-Nuss, M. *et al*. Genomic insights into the *Ixodes scapularis* tick vector of Lyme disease. *Nat. Commun.* 7:10507 doi: 10.1038/ncomms10507 (2016).

## Supplementary Material

Supplementary Information Supplementary Figures 1-25, Supplementary Tables 1-38, Supplementary Note 1, Supplementary Methods and Supplementary References

## Figures and Tables

**Table 1:** Summary of the *Ixodes scapularis* genome assembly and annotation statistics.

*IscaW1 assembly statistics*	
Total number of sequence reads	17.4 M
Estimated fold coverage of the assembly	3.8-fold
Number of scaffolds	369,495
N_50_ scaffold length	51,551 bp
Number of contigs used in assembly	570,637
N_50_ contig length	2,942 bp
Total length of combined contigs	1.4 Gb
Total length of combined scaffolds (including gaps)	1.8 Gb
Estimated genome size	2.1 Gb
	
*Annotation release 1.2 statistics*	
Total number of genes	20,486
Mean gene length	10,589 bp
Mean coding DNA sequence (CDS) length	855 bp
