# Supplementary material for: Genomic insights into the Ixodes scapularis tick vector of Lyme disease
Source: Nat Commun. 2016 Feb 9;7:10507. doi: 10.1038/ncomms10507 (PMC4748124; doi:10.1038/ncomms10507)
Supplement: Supplementary Information — Supplementary Figures 1-25, Supplementary Tables 1-38, Supplementary Note 1, Supplementary Methods and Supplementary References [file ncomms10507-s1.pdf]

Supplementary Figures

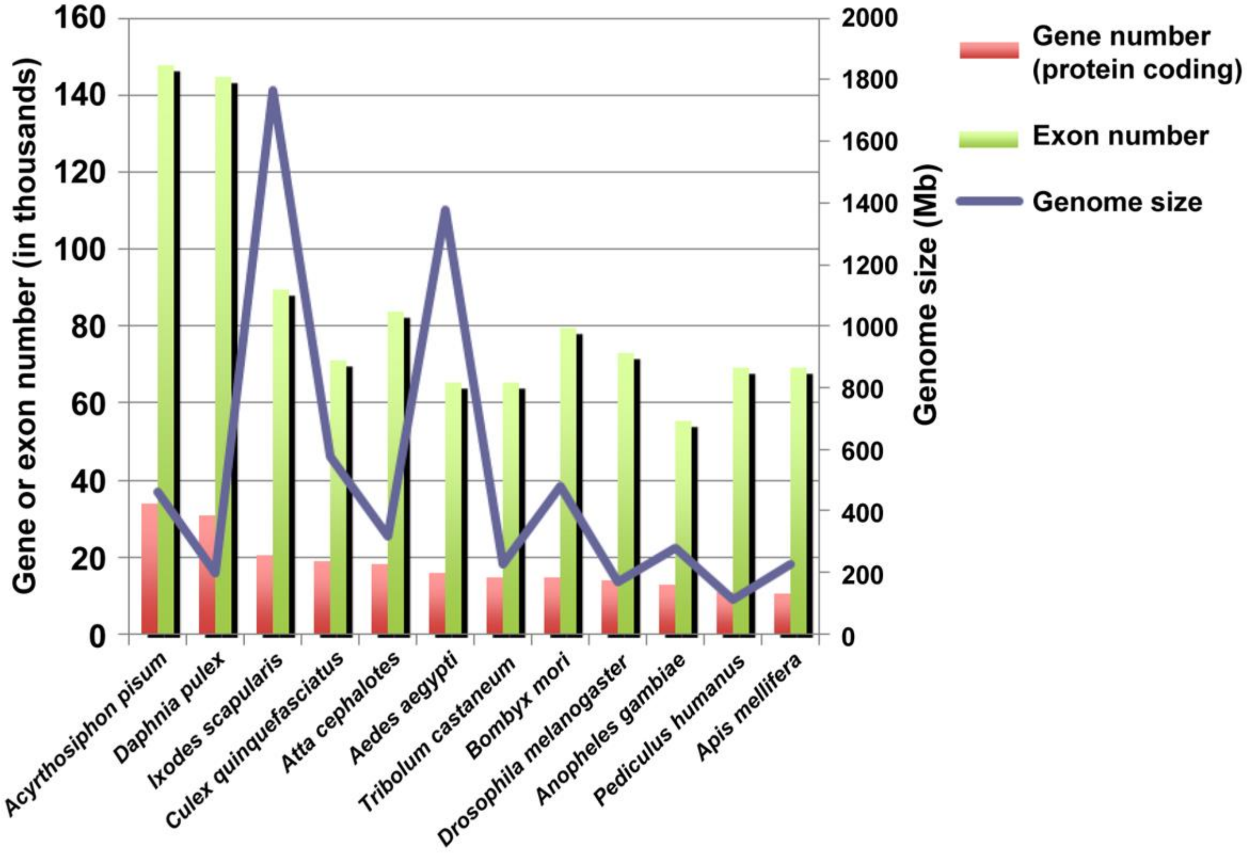

Supplementary Fig. 1. Comparison of the number of genes, exons and genome size (in Mb) in 12 arthropod genomes (based on EnsemblGenomes release 12).

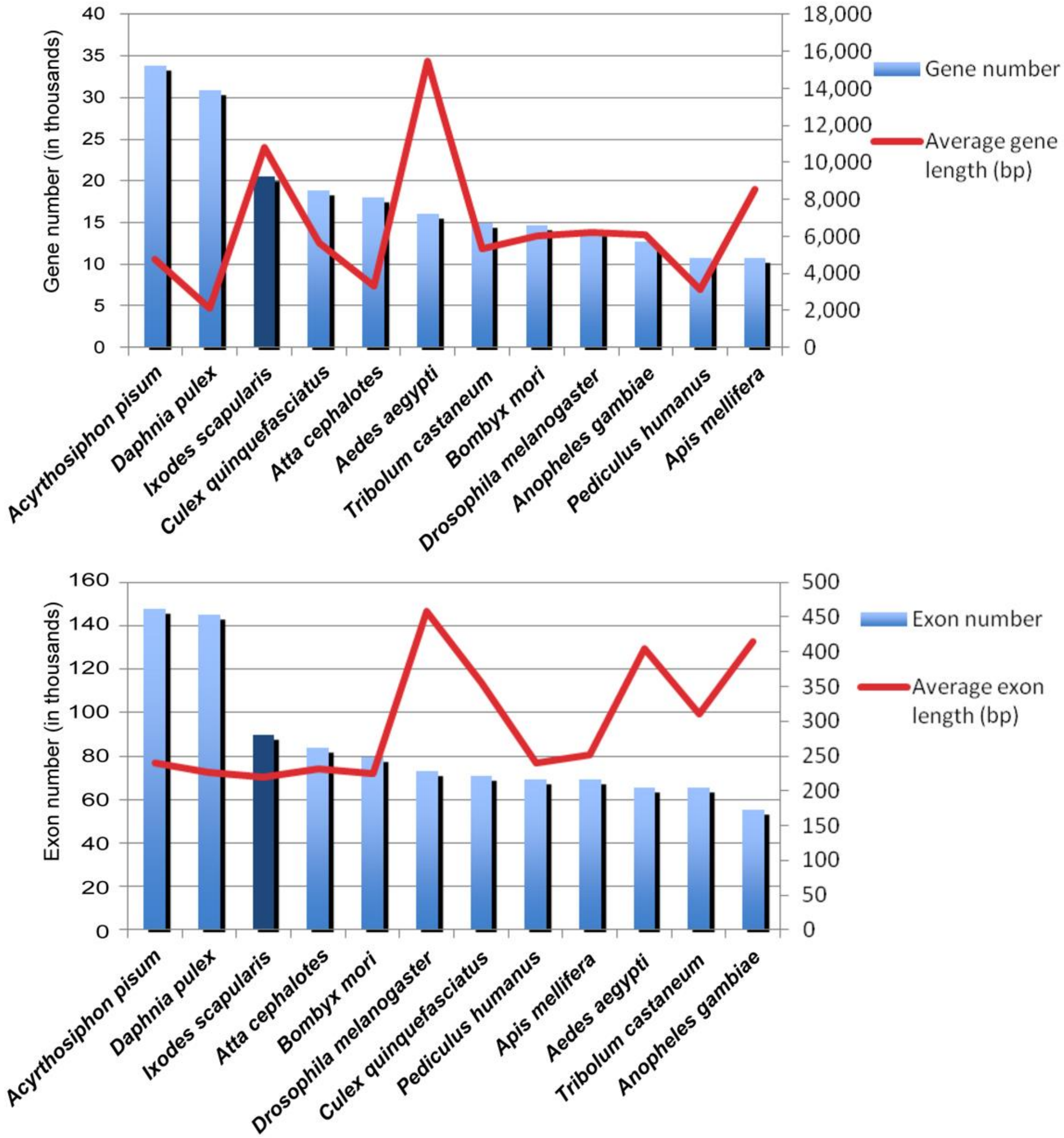

Supplementary Fig. 2. (a) Comparison of the number of genes and their average length in 12 arthropod genomes (based on EnsemblGenomes release 12). (b) Comparison of the number of exons and their average length in 12 arthropod genomes (based on EnsemblGenomes release 12).

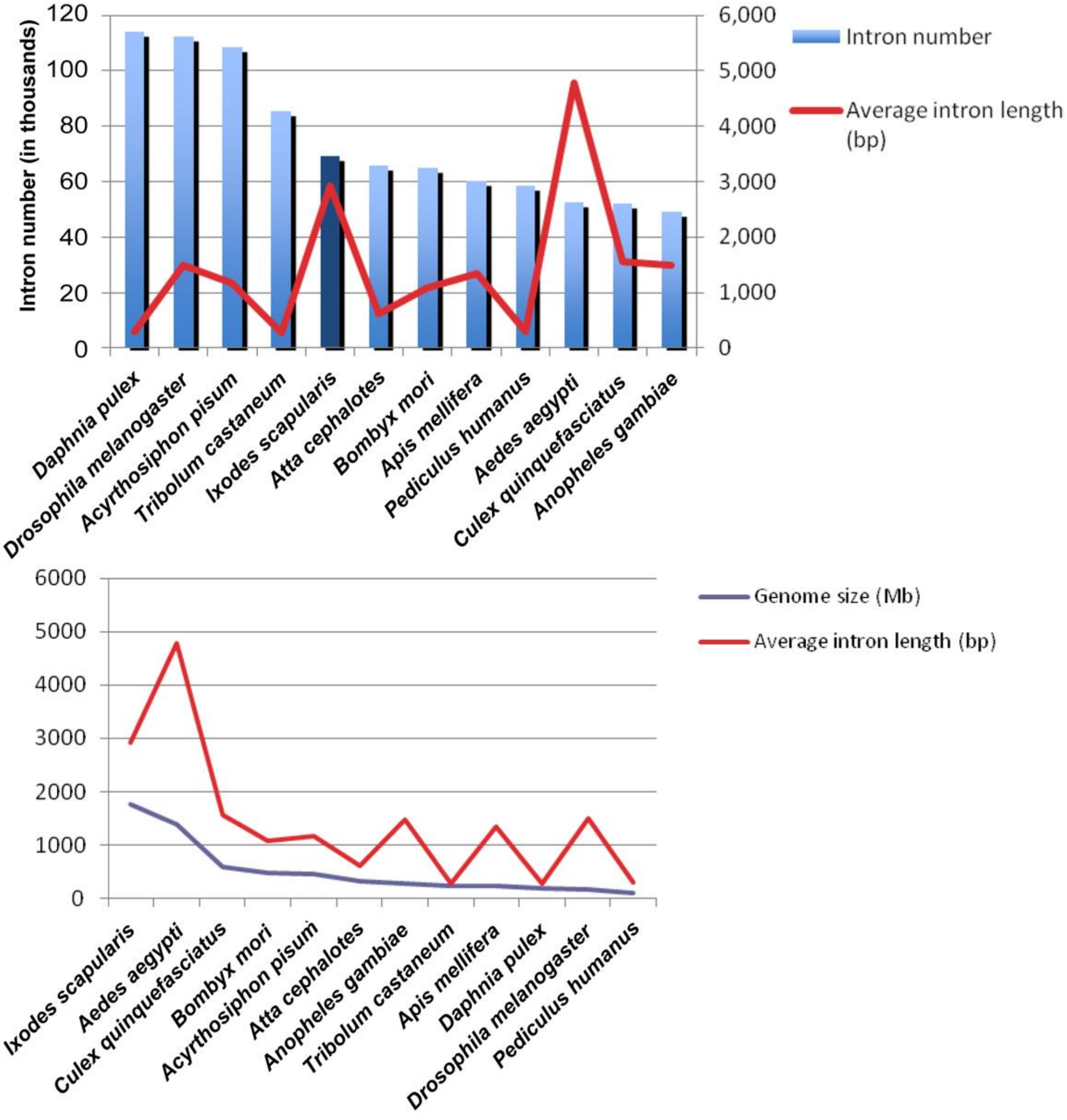

Supplementary Fig. 3. (a) Comparison of the number of introns and their average length in 12 arthropod genomes (based on EnsemblGenomes release 12). (b) Comparison of the genome size and and average intron length in 12 arthropods genomes (based on EnsemblGenomes release 12).

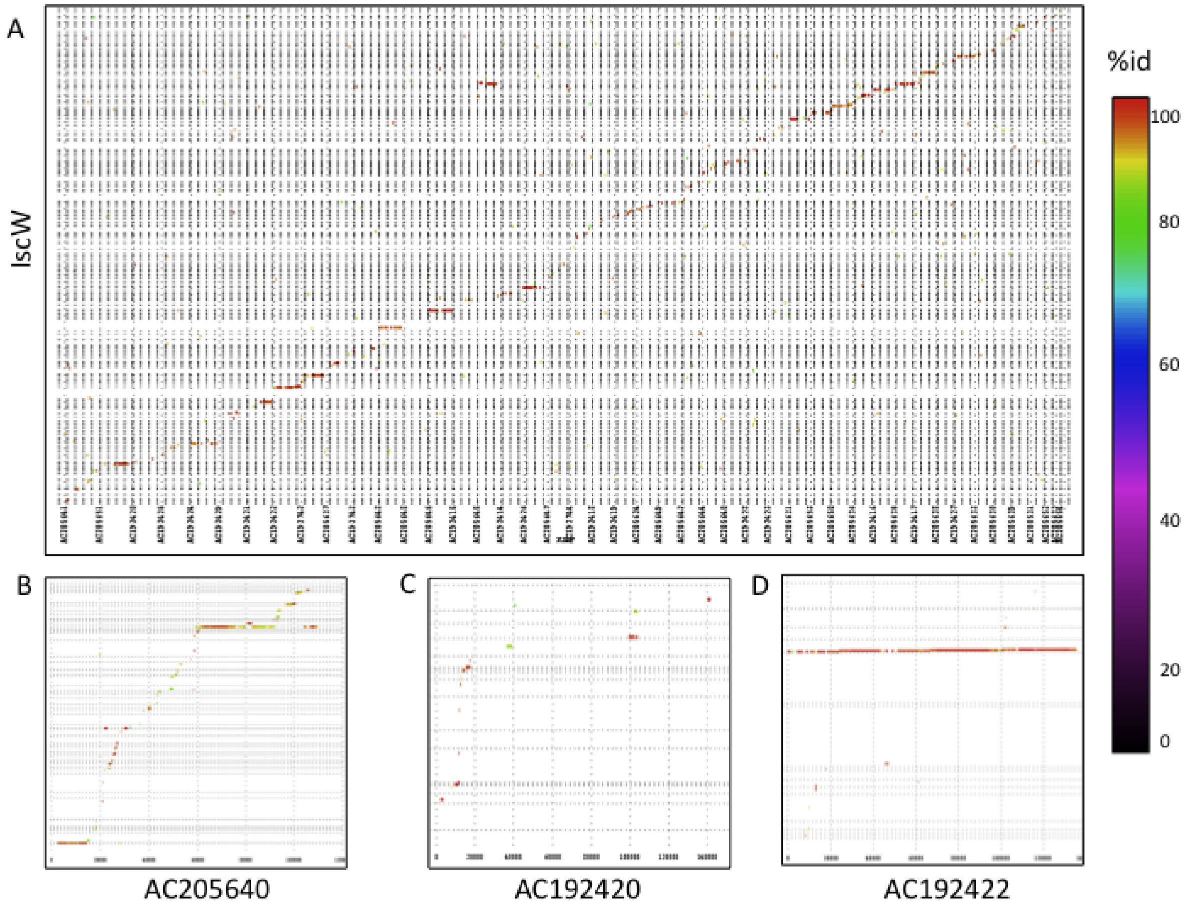

Supplementary Fig. 4. BAC mapping to *I. scapularis* genome scaffolds. Annotated scaffolds were mapped to 45 sequenced BACs to assess the level of representation in the current annotated assembly. **(a):** Nucmer alignments of all BACs (x axis) to IscW annotated scaffolds (y axis). (b, c and d): Individual BAC sequences represented in two or more IscW scaffolds **(b)**, BAC sequence does not align significantly to any scaffold **(b)** and BAC sequence is represented by a single IscW scaffold **(c).** All mappings are shown in Supplementary Table 4.

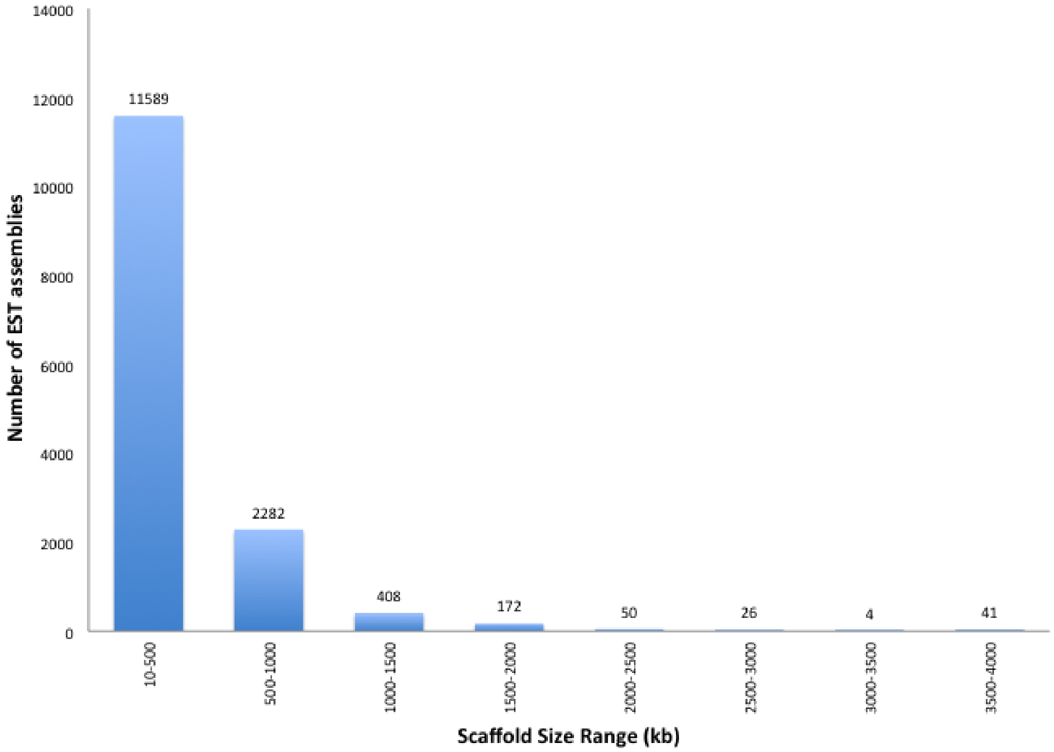

Supplementary Fig. 5. Alignment of *Ixodes scapularis* Expressed Sequence Tag (EST) and cDNA sequences to IscaW1 scaffolds. The *I. scapularis* EST set, comprising 193,151 EST and cDNA sequences, was aligned to the IscaW1 scaffold sequences and assembled. EST sequences were utilized to generate high quality training sets and improve gene structures. ESTs assembled using PASA, were aligned to the core scaffolds representing the annotated genome. ESTs were also used to evaluate and capture potential genes in small contigs that were not initially included in the annotated scaffolds. EST hits to small contigs that are not part of the annotated scaffolds typically represent transcripts derived from transposable elements such as non-LTR type elements, and do not contain an open reading frame.

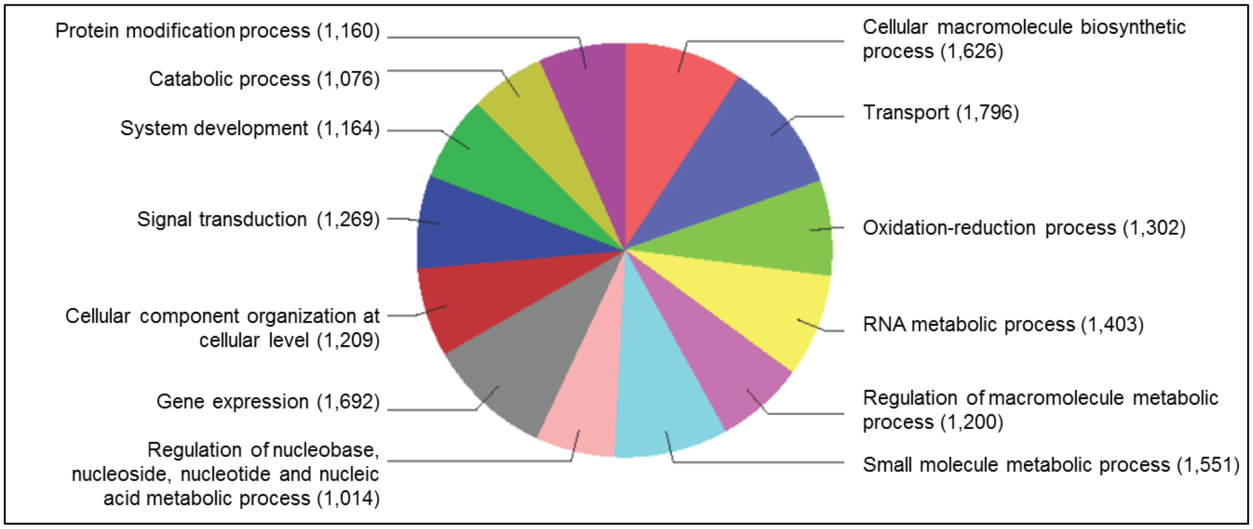

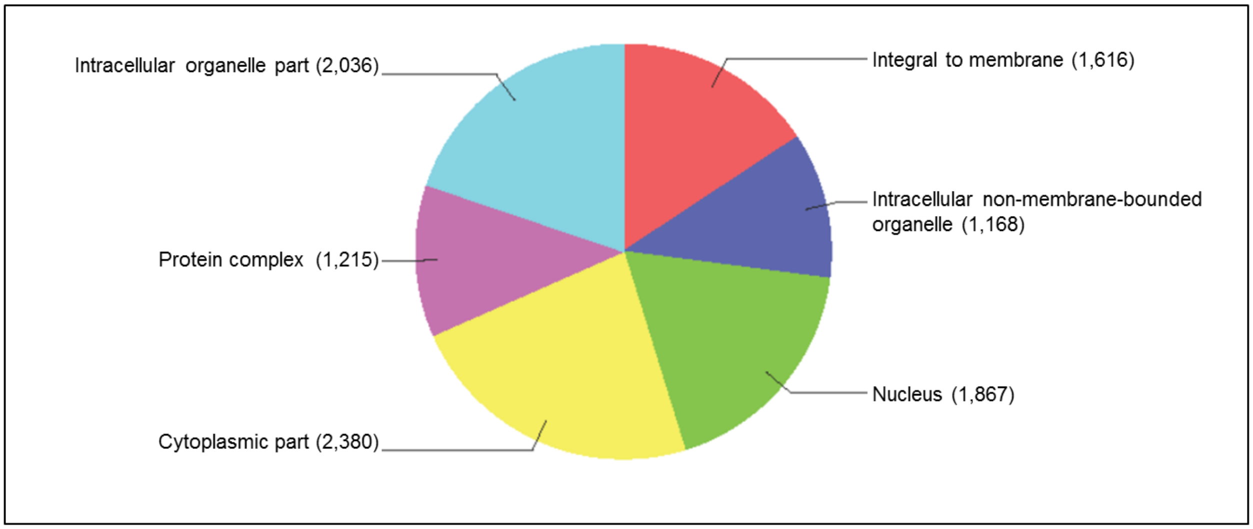

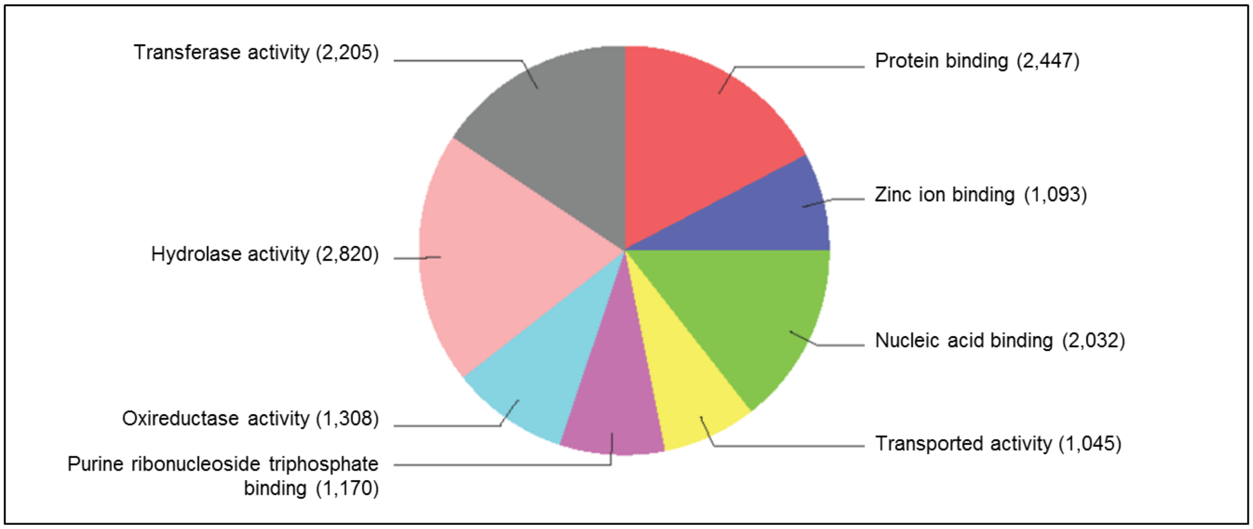

Supplementary Fig. 6. Functional analysis of the *Ixodes scapularis* IscaW1 gene models showing the gene ontology results for: (a) Biological Process (b) Cellular

Component and (c) Molecular Function categories. Multi-level pie charts show all GO terms that exceed the cut-off value of 1,000 sequences. Numbers in parentheses indicate the total number of sequences assigned to a specific GO term.

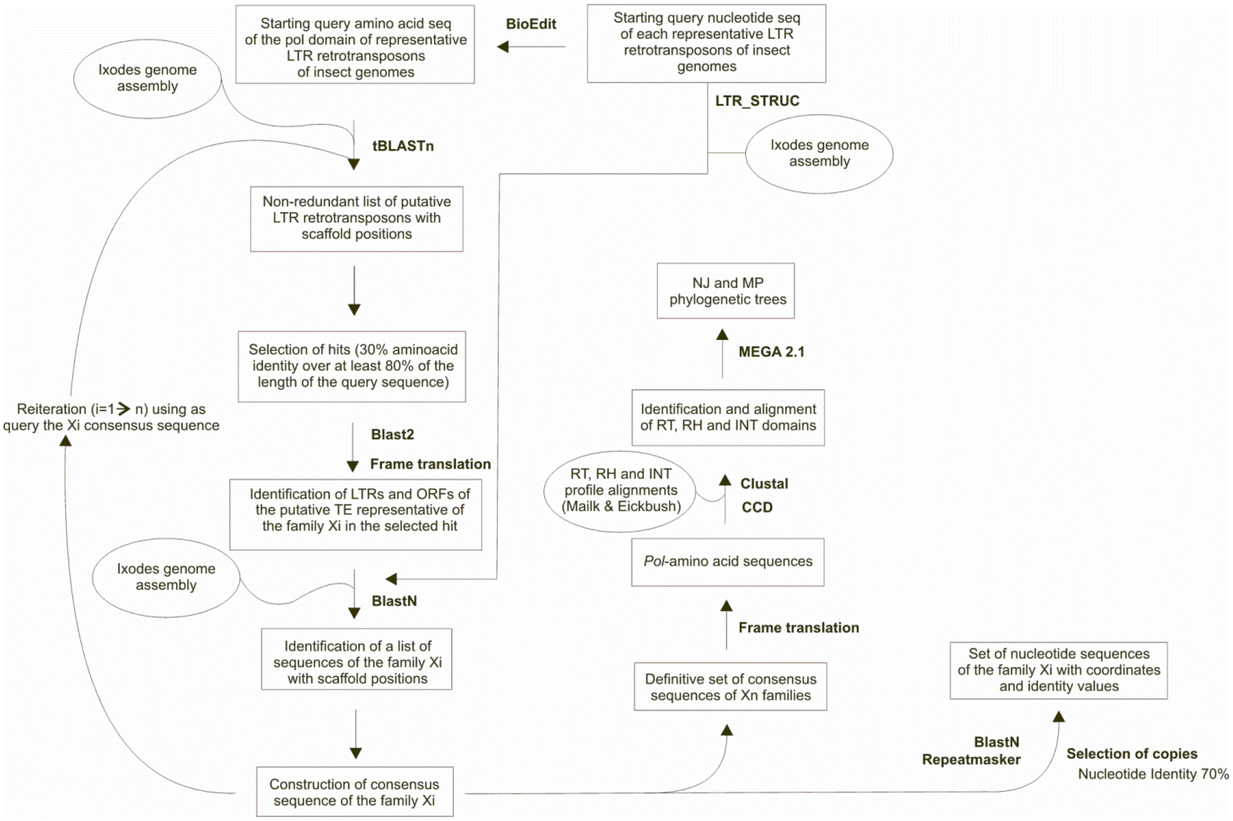

Supplementary Fig. 7. Schematic showing the strategy employed for the identification of all LTR retrotransposons in the genome of *Ixodes scapularis*. b. Identification of LTR elements. b. Phylogenetic analysis. C. Identification of the number of copies of each LTR retrotransposon. Circles indicate databases used for searches. Rectangles indicate input/output files and. Programs used are written in bold beside arrows. See Supplementary text for details. CCD = Conserved Domain Database, RT = Retrotranscriptase; RH = Ribonuclease; INT = Integrase.

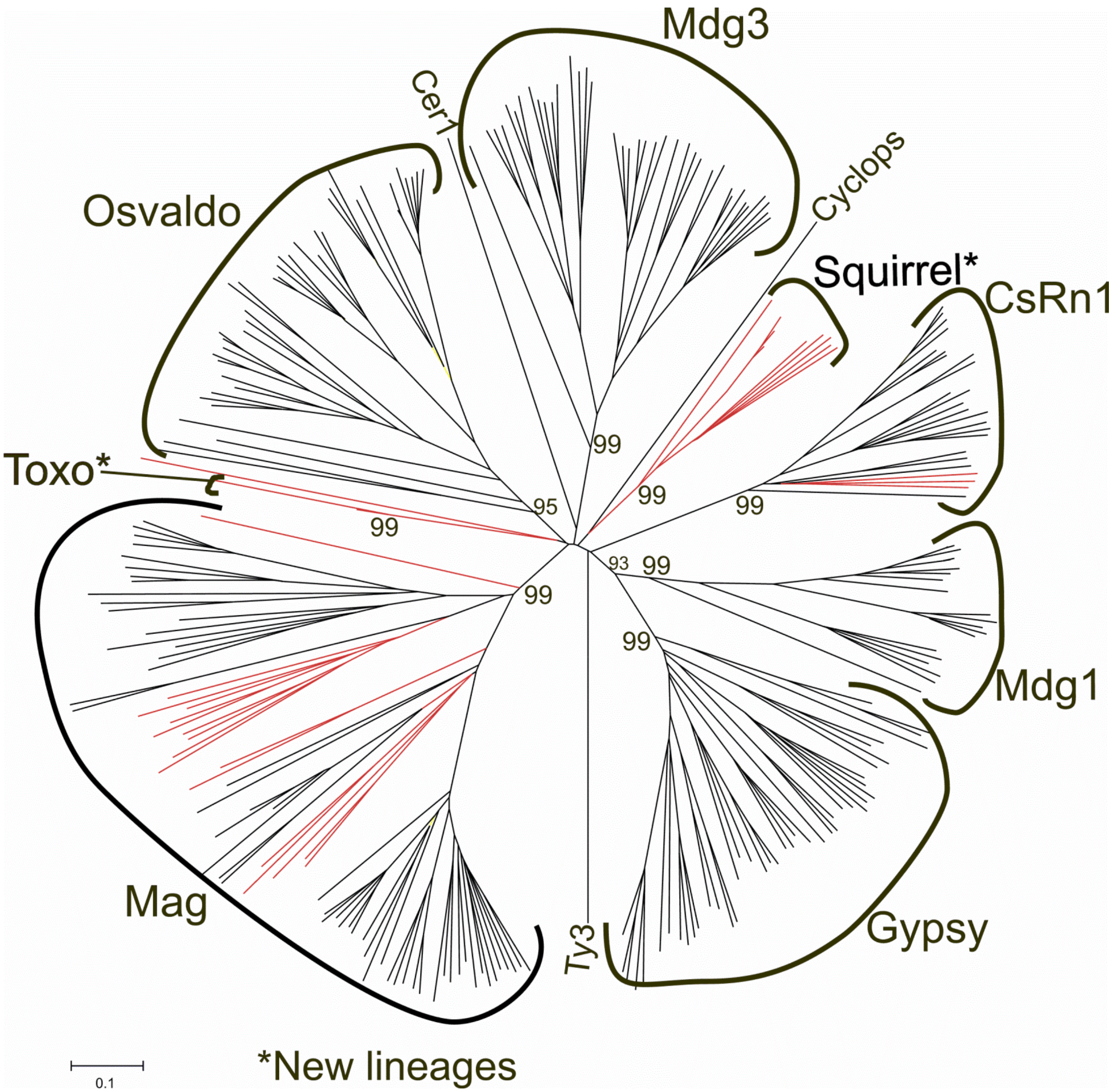

Supplementary Fig. 8. New Ty3/gypsy lineages in the genome of *Ixodes scapularis*. Phylogenetic relationships between the Ty3/gypsy retrotransposons of *Ixodes scapularis* and insect genomes inferred by the NJ method and based on the conserved domains of RT, RnaseH, and INT[51]. Bootstrap values (1,000 replications) supporting the clusters of each lineage of the Ty3/gypsy family are shown. Names of Ty3/gypsy lineages are shown in capitals. Two new lineages (named Toxo and Squirrel; indicated by asterisk) are supported by bootstrap values of 99%. The phylogeny contains elements from *Ixodes scapularis* (red branches), *Drosophila melanogaster, Anopheles gambiae, Aedes aegypti*, and *Culex pipiens*. Non-insect representative elements in the phylogeny are the retrotransposons *Ty3* from the yeast *Saccharomyces cerevisiae*, *Cyclops* from the plant *Vicia faba*, and *Cer1* from the Nematoda *Caenorhabditis elegans*.

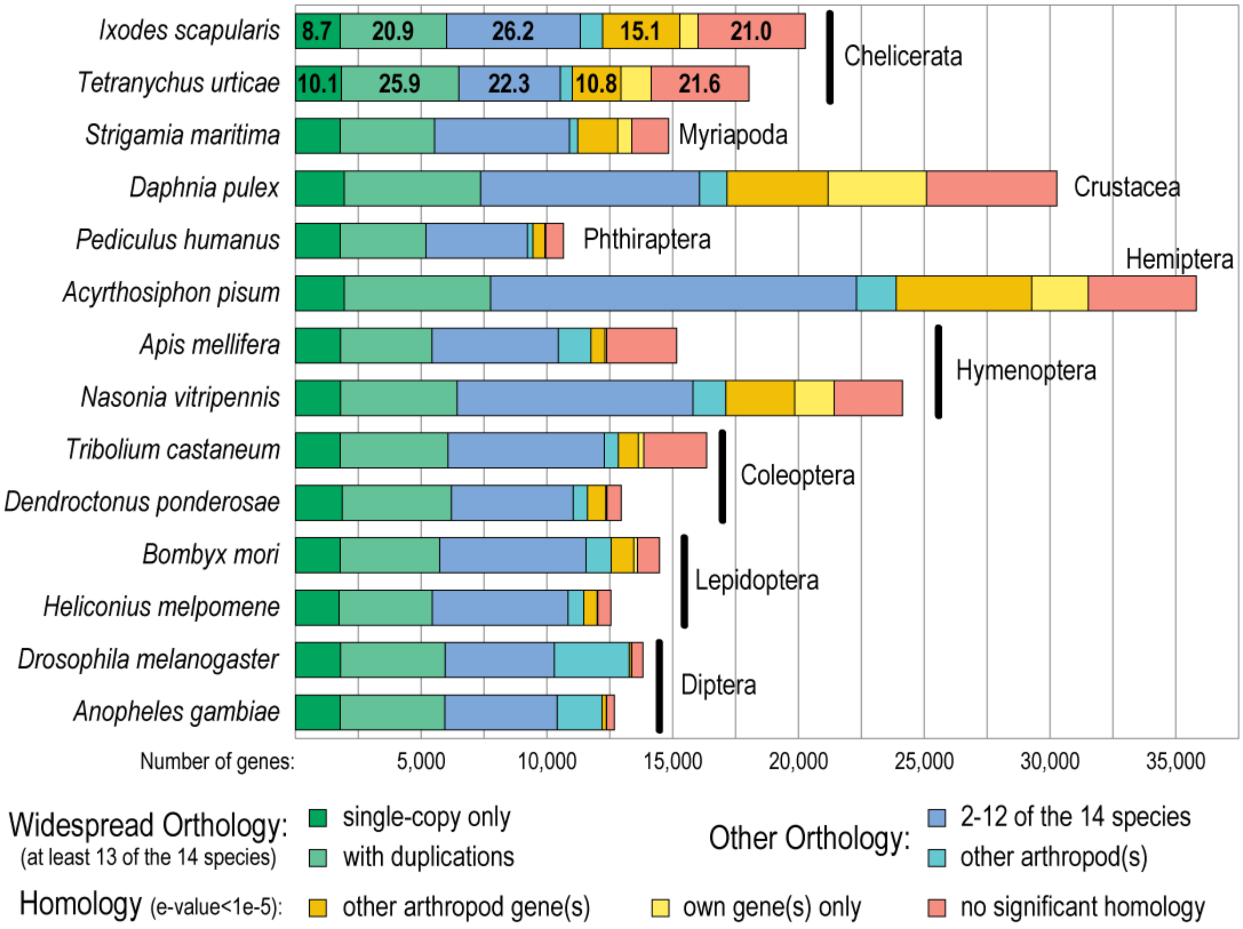

Supplementary Fig. 9. *Ixodes scapularis* gene orthology and homology across Arthropoda. Orthologous and homologous relations between *I. scapularis* genes and those from other sequenced arthropods were examined using orthologous groups delineated across 87 arthropod species from www.OrthoDB.org[25] (release 8). About 30% of *I. scapularis* genes have recognizable orthologs in all or almost all of the representative species selected from nine different arthropod lineages (green fractions, at least 13 of the 14 species - single-copy or with duplications). A further ~30% of *I. scapularis* genes are less widely conserved across Arthropoda (blue fractions, present in 2-12 of the 14 species, or present in at least one of the 73 other arthropods). Of the remaining *I. scapularis* genes with no identifiable orthology, about half exhibit homology (e-value <1e-05) to genes from the other 86 arthropods or to other *I. scapularis* genes (yellow fractions, homology to other arthropod genes or homology only to other genes in the same genome). The two chelicerates show very similar fractions of genes that currently have no significant homologs in other arthropod genomes, so-called “orphan” genes. The major fractions of the two chelicerate species’ gene sets are labeled with the corresponding percentages of their total gene counts.

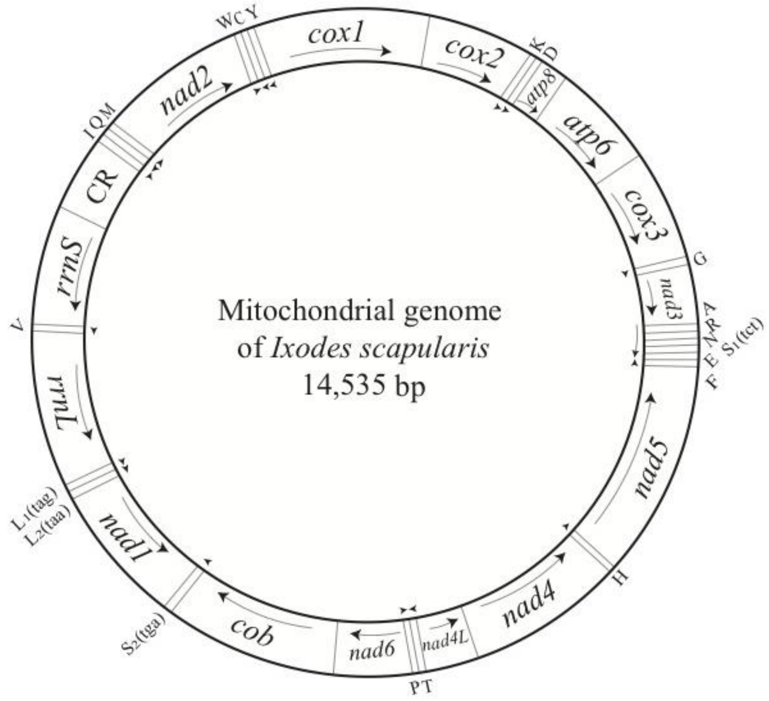

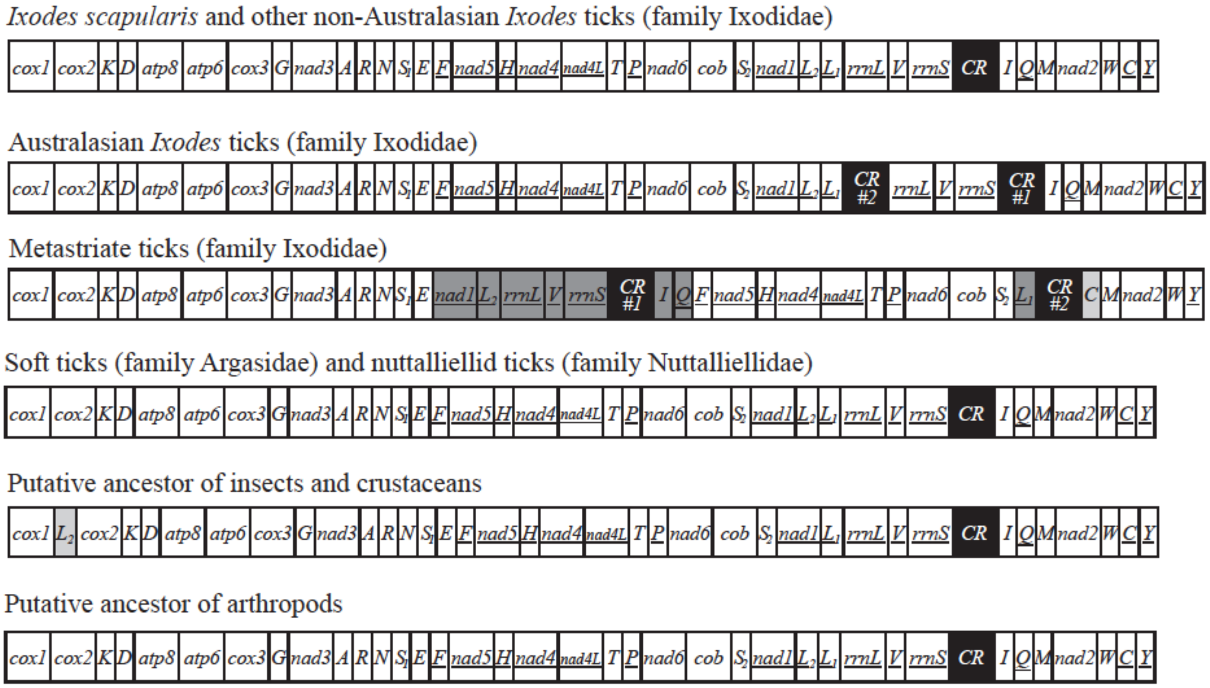

Supplementary Fig. 10. The organization of the mitochondrial genome of *Ixodes scapularis* (a), and comparison of mitochondrial gene arrangement between *I. scapularis* and other ticks and arthropods (b). (a) Genes are shown as boxes and were drawn approximately to scale. Arrows indicate the orientation of transcription.

Protein-coding and rRNA genes are abbreviated as *atp6* and *atp8* (for ATP synthase subunits 6 and 8), *cox1-3* (for cytochrome c oxidase subunits 1-3), *cob* (for cytochrome b), *nad1-6* and *4L* (for NADH dehydrogenase subunits 1-6 and 4L), and *rrnL* and *rrnS* (for large and small rRNA subunits). tRNA genes are shown with the single-letter abbreviations of their corresponding amino acids. The two tRNA genes for leucine are L_1 (anti-codon sequence UAG) and L_2(UAA), and those for serine are S_1(UCU) and S_2(UGA). *CR* is the abbreviation for the putative control region. **(b)** The circular mitochondrial genomes are linearized at the 5' end of *cox1* (for the purpose of illustration). Genes and putative control regions (*CR*) are shown as boxes but were not drawn to scale. Genes are transcribed from left to right except those underlined, which are transcribed from right to left. Putative control regions are highlighted in black. Dark, grey, shaded-boxes indicate genes that changed position relative to the putative ancestor of arthropods. Pale, grey, shaded-boxes indicate genes that changed both position and the orientation-of-transcription, relative to the putative ancestor of arthropods.

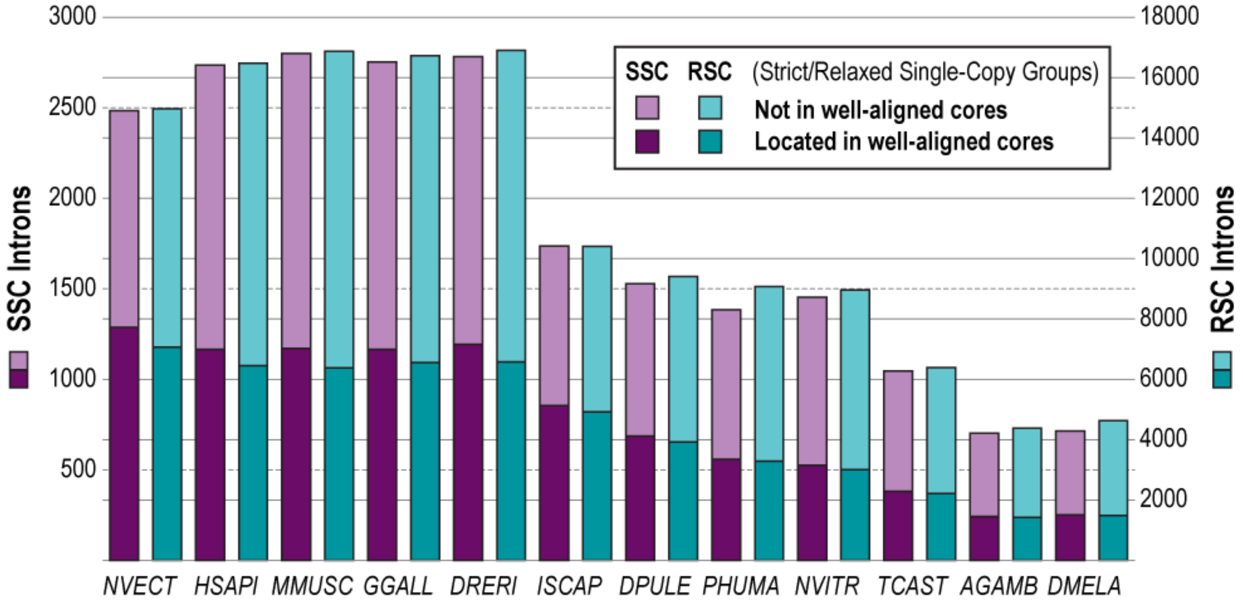

Supplementary Fig. 11. Introns in single-copy orthologs across 12 species. Introns were mapped on to the protein sequence alignments of 524 Strict Single-Copy (SSC) orthologs and 1,529 Relaxed Single-Copy (RSC) orthologs, allowing for small splice site changes, and conserved regions with an intron in at least one species were identified by requiring >30% amino acid identity in the aligned blocks flanking the intron position. Between 32% and 52% of introns in each species are located in well-aligned core regions of the ortholog alignments and therefore may be compared across the 12 species, and examining SSC or RSC sets does not affect the proportions of informative introns. Abbreviations: *NVECT*, *Nematostella vectensis*; *HSAPI*, *Homo sapiens*; *MMUSC*, *Mus musculus*; *GGALL*, *Gallus gallus*; *DRERI*, *Danio rerio*; *ISCAP*, *Ixodes scapularis*; *DPULE*, *Daphnia pulex*; *PHUMA*, *Pediculus humanus*; *NVITR*, *Nasonia vitripennis*; *TCAST*, *Tribolium castaneum*; *AGAMB*, *Anopheles gambiae*; *DMELA*, *Drosophila melanogaster*.

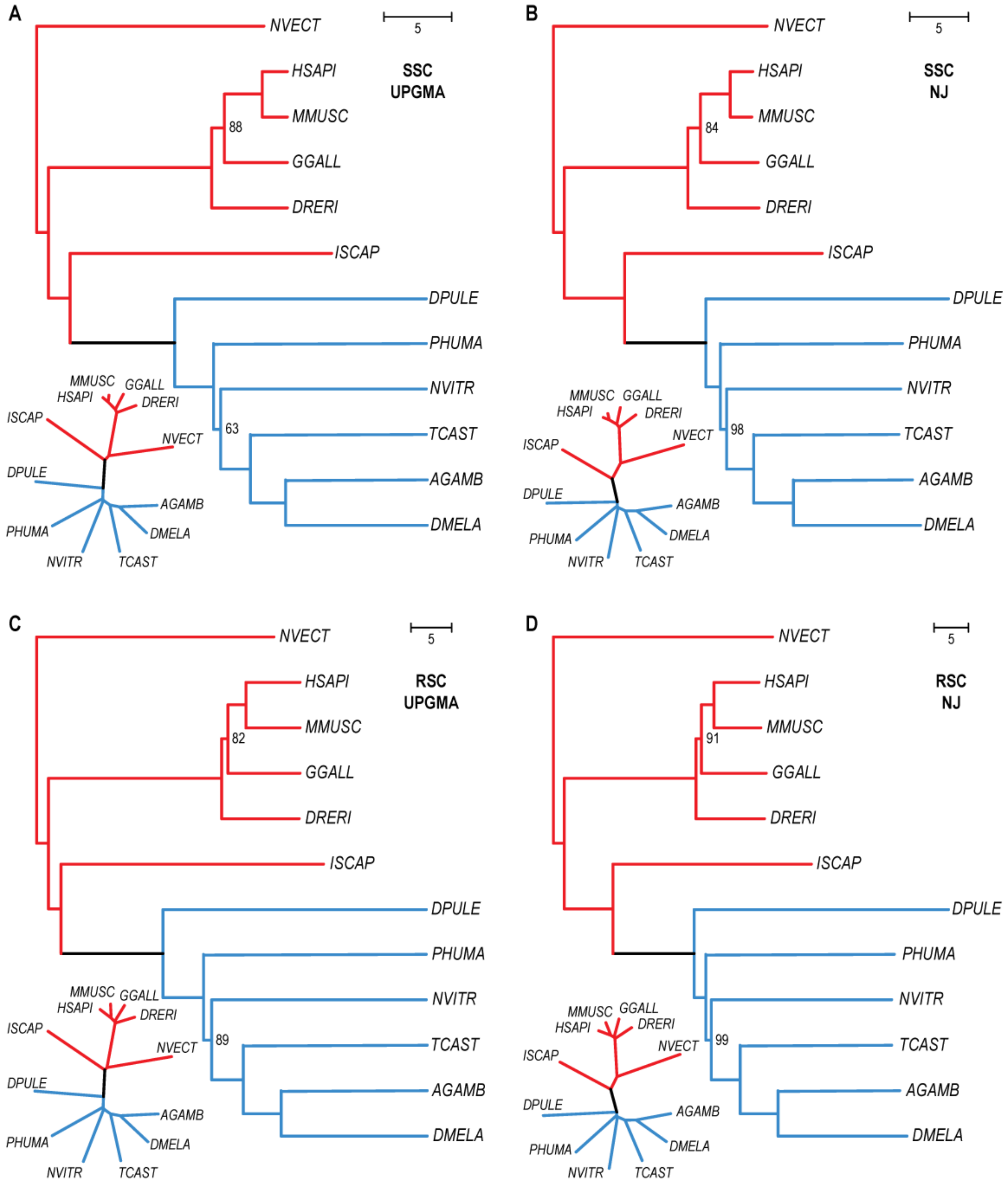

Supplementary Fig. 12. 12-species phylogeny based on the conservation of intron positions. Euclidean distance matrices from presence/absence matrices for 4,621 Strict Single-Copy (SSC, a & b) and 13,459 Relaxed Single-Copy (RSC, c & d) ortholog intron positions were employed to construct phylogenetic trees using Unweighted Pair Group Method with Arithmetic Mean (UPGMA, a & c) and Neighbor Joining (NJ, b & d) algorithms. *I. scapularis* (*ISCAP*) consistently shows greater similarity to the outgroup

species (red), human, mouse, chicken, zebrafish and sea anemone, than to the pancrustaceans (blue). Bootstrap values are indicated for the two nodes on each tree with less than 100% support: the alternative topologies cluster *PHUMA* and *NVITR* together and/or swap the positions of *DRERI* and *GGALL*. Unrooted radial trees are presented at the lower left of each panel. Abbreviations: *NVECT*, *Nematostella vectensis*; *HSAPI*, *Homo sapiens*; *MMUSC*, *Mus musculus*; *GGALL*, *Gallus gallus*; *DRERI*, *Danio rerio*; *ISCAP*, *Ixodes scapularis*; *DPULE*, *Daphnia pulex*; *PHUMA*, *Pediculus humanus*; *NVITR*, *Nasonia vitripennis*; *TCAST*, *Tribolium castaneum*; *AGAMB*, *Anopheles gambiae*; *DMELA*, *Drosophila melanogaster*.

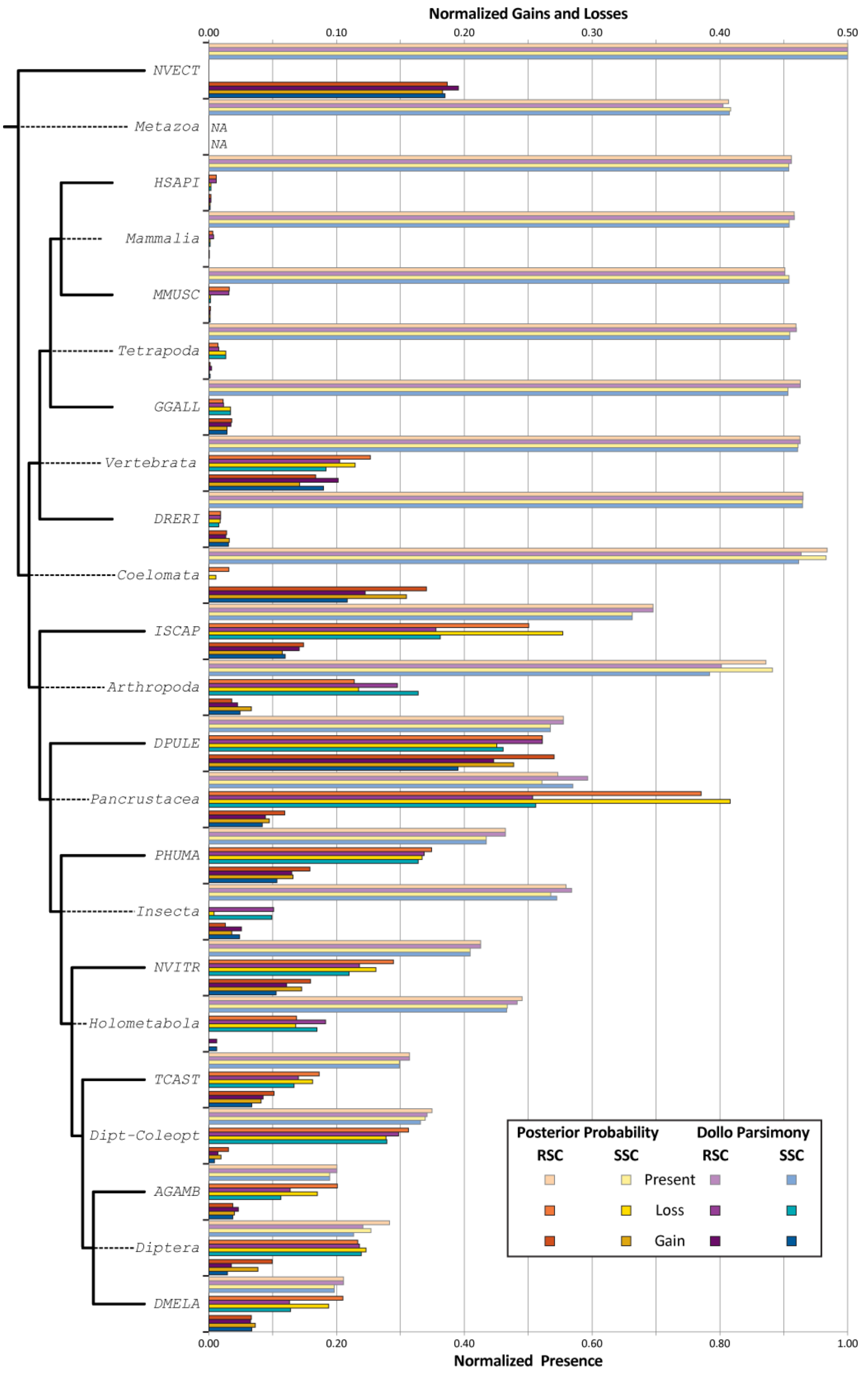

Supplementary Fig. 13. Intron gain/loss estimates across the 12-species phylogeny. Analysis of intron gain and loss across the 12-species phylogeny for the Strict Single-Copy (SSC) and Relaxed Single-Copy (RSC) sets of orthologs using Dollo Parsimony (DP) and Posterior Probability (PP) methods implemented in the MALIN

suite for maximum likelihood analysis of intron evolution in eukaryotes[33]. Data are normalized by the maximum number of introns (always *NVECT*) in order to compare the estimates from different sets using different methods. Normalization: Gained, Lost, or Present Introns / Maximum number of Introns. NB: the scale for the normalized gain and loss estimates (0.0-0.5) is double that of the normalized presence data (0.0-1.0). Corresponding numbers are presented in Supplementary Table 9. Abbreviations: *NVECT*, *Nematostella vectensis*; *HSAPI*, *Homo sapiens*; *MMUSC*, *Mus musculus*; *GGALL*, *Gallus gallus*; *DRERI*, *Danio rerio*; *ISCAP*, *Ixodes scapularis*; *DPULE*, *Daphnia pulex*; *PHUMA*, *Pediculus humanus*; *NVITR*, *Nasonia vitripennis*; *TCAST*, *Tribolium castaneum*; *AGAMB*, *Anopheles gambiae*; *DMELA*, *Drosophila melanogaster*.

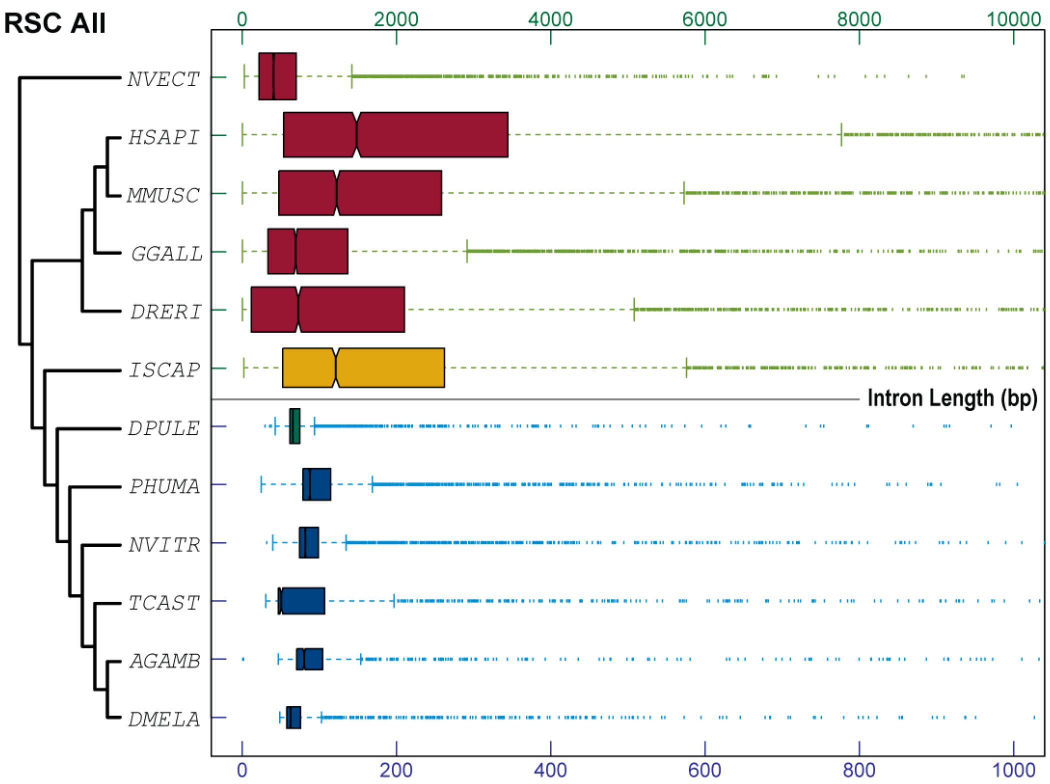

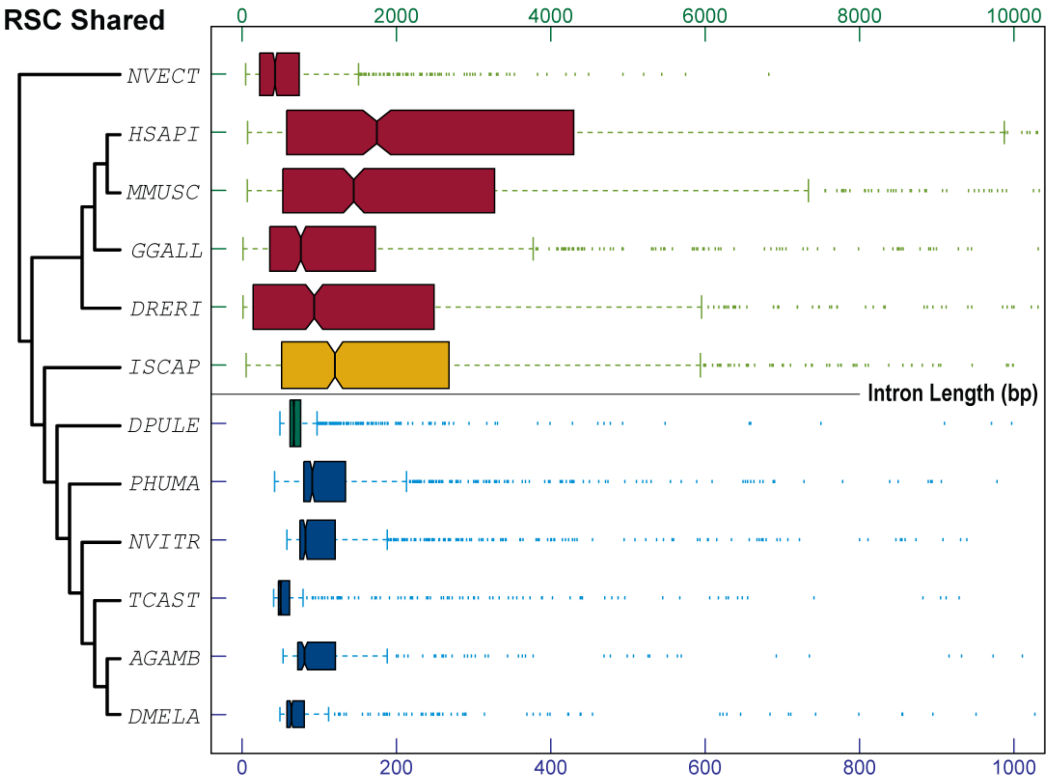

Supplementary Fig. 14. Intron length distributions across 12 species. The length distributions of informative introns for the Relaxed Single-Copy (RSC) orthology sets are

plotted: RSC All, all informative introns; RSC Shared, informative introns found in *ISCAP* and *DPULE* and at least one non-arthropod and at least one insect. Boxes indicate the median, 1st and 3rd quartiles, and whiskers show up to 1.5 times the interquartile range, box heights are proportional to the number of introns. *I. scapularis* (*ISCAP*) introns are most similar to those of *MMUSC* and other vertebrates, and more than an order of magnitude longer than pancrustacean introns. *NVECT*, *HSAPI*, *MMUSC*, *GGALL*, *DRERI*, and *ISCAP* scale to 10,000 bp (green axis) while the pancrustaceans scale to 1,000 bp (blue axis). The numbers, along with Wilcoxon test results, are presented in Supplementary Table 10. Abbreviations: *NVECT*, *Nematostella vectensis*; *HSAPI*, *Homo sapiens*; *MMUSC*, *Mus musculus*; *GGALL*, *Gallus gallus*; *DRERI*, *Danio rerio*; *ISCAP*, *Ixodes scapularis*; *DPULE*, *Daphnia pulex*; *PHUMA*, *Pediculus humanus*; *NVITR*, *Nasonia vitripennis*; *TCAST*, *Tribolium castaneum*; *AGAMB*, *Anopheles gambiae*; *DMELA*, *Drosophila melanogaster*.

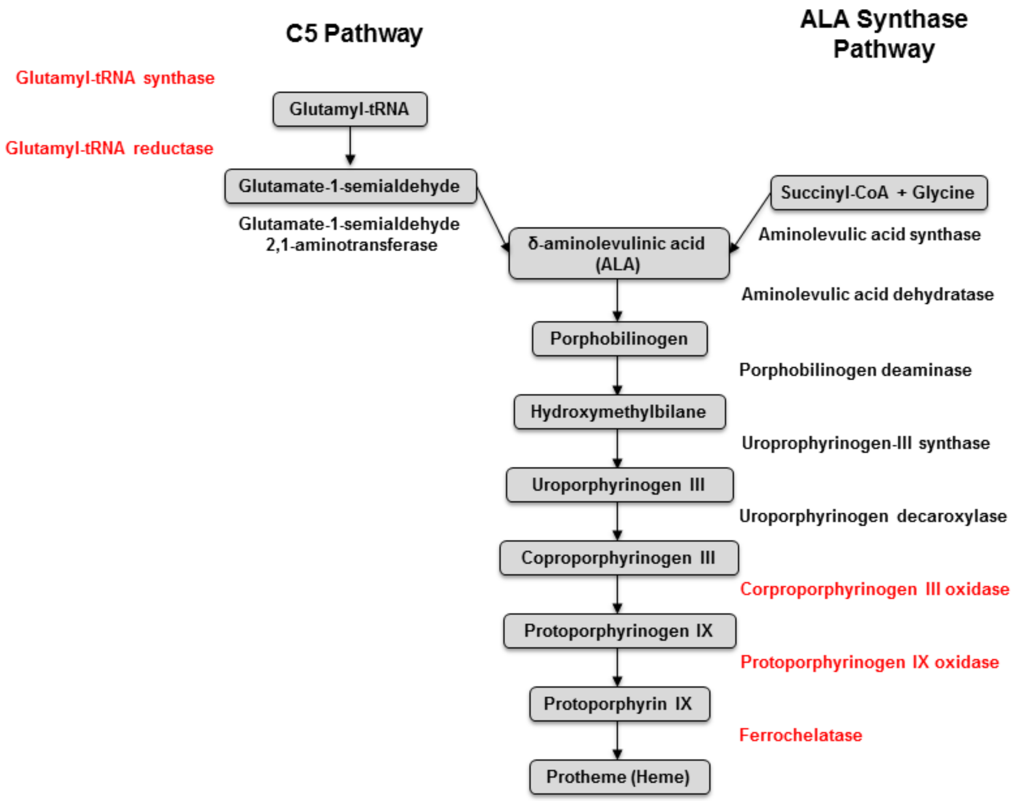

Supplementary Fig. 15. Heme synthesis pathways and heme synthesis genes identified in the *Ixodes scapularis* genome. Candidate heme synthesis genes identified in the *I. scapularis* genome are shown in red. The VectorBase accession numbers for each of the putative *I. scapularis* heme synthesis genes are listed in Supplementary Table 20.

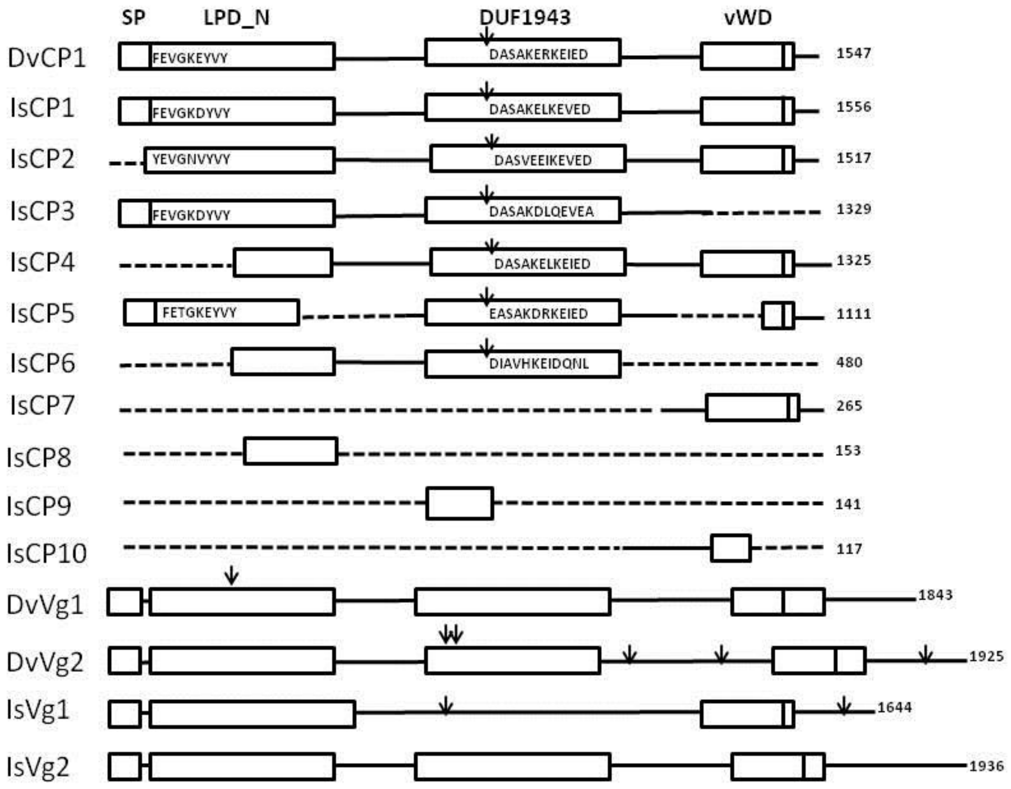

Supplementary Fig. 16. Schematic representation of putative CPs and Vgs detected in *Ixodes scapularis* genome compared to the confirmed counterparts from *Dermacentor variabilis*. DUF1943: a domain of unknown function, LPD_N: lipid binding domain, SP: signal peptide, vWD: von Willebrand type D domain. Arrows represent the RXXR location while vertical solid lines represent the GL/ICG domain location. Amino acid sequence shown represents the N-terminal sequence of the 2 CP subunits starting directly downstream of the signal peptide and the RXXR cleavage sites. Dash lines represent missing sequences. DvCP1 (ABD83654), DvVg1 (AAW78577), DvVg2 (ABW82681), IsCP1 (ISCW021709), IsCP2 (ISCW014675), IsCP3 (ISCW021710), IsCP4 (ISCW012424), IsCP5 (ISCW012423), IsCP6 (ISCW021704), IsCP7 (ISCW021707), IsCP8 (ISCW021706), IsCP9 (ISCW021705), IsCP10 (ISCW024299), IsVg1 (ISCW013727), IsVg2 (ISCW021228).

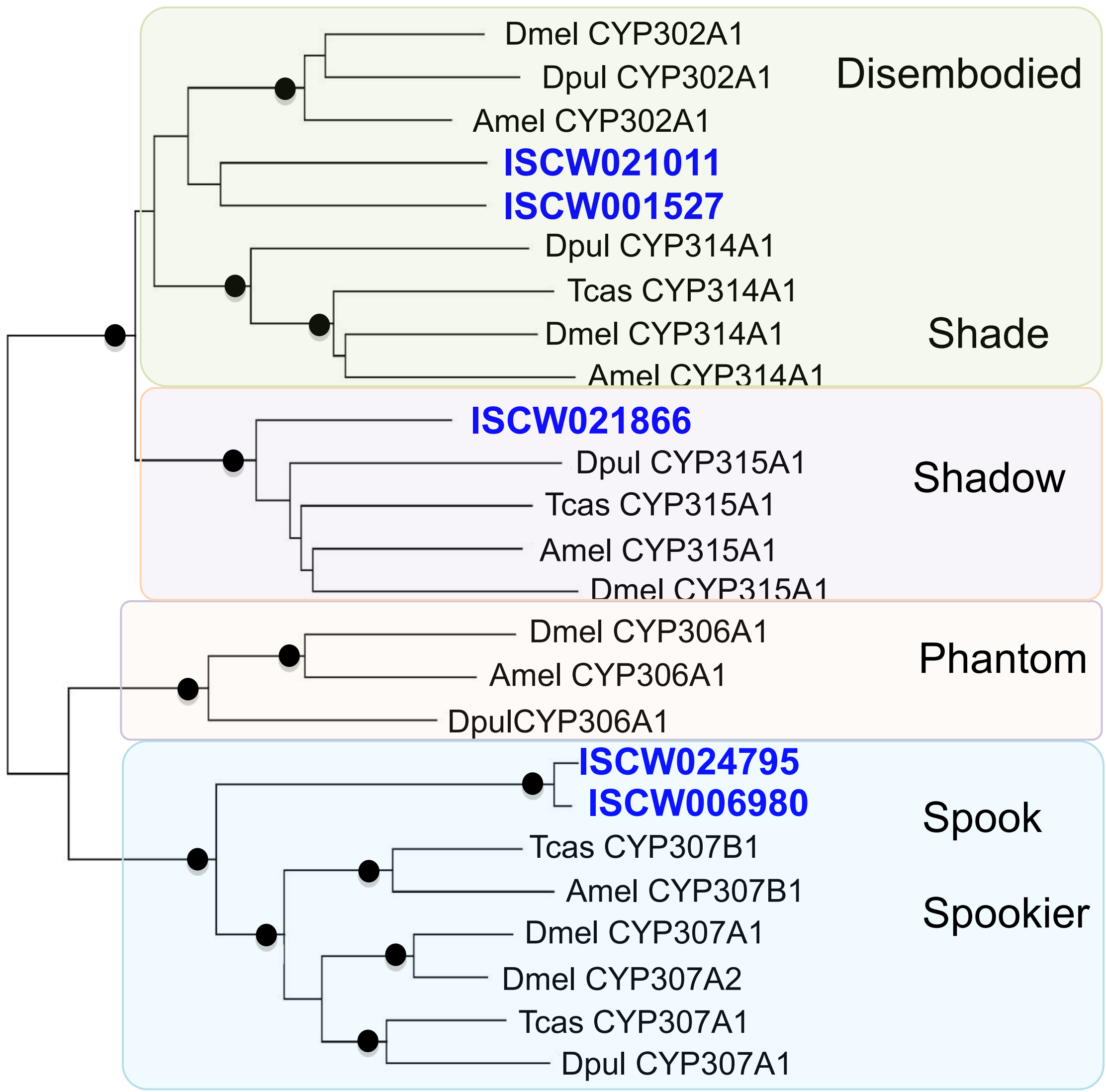

Supplementary Fig. 17. Cytochrome P450 genes orthologous to the Halloween genes that encode steroidogenic cytochrome P450s for hydroxylations of 20-hydroxylecdysone. Blue font indicates the VectorBase accession number for the corresponding predicted protein sequences identified in the *I. scapularis* genome. Solid circles at branch points indicate bootstrapping support with higher than 70% in 1000 replication of the neighbor-joining tree. Insect genes in the orthologous group are from *Tribolium castaneum* (Tcas), *Drosophila melanogaster* (Dmel), *Apis mellifera* (Amel), and *Daphnia pulex* (Dpul).

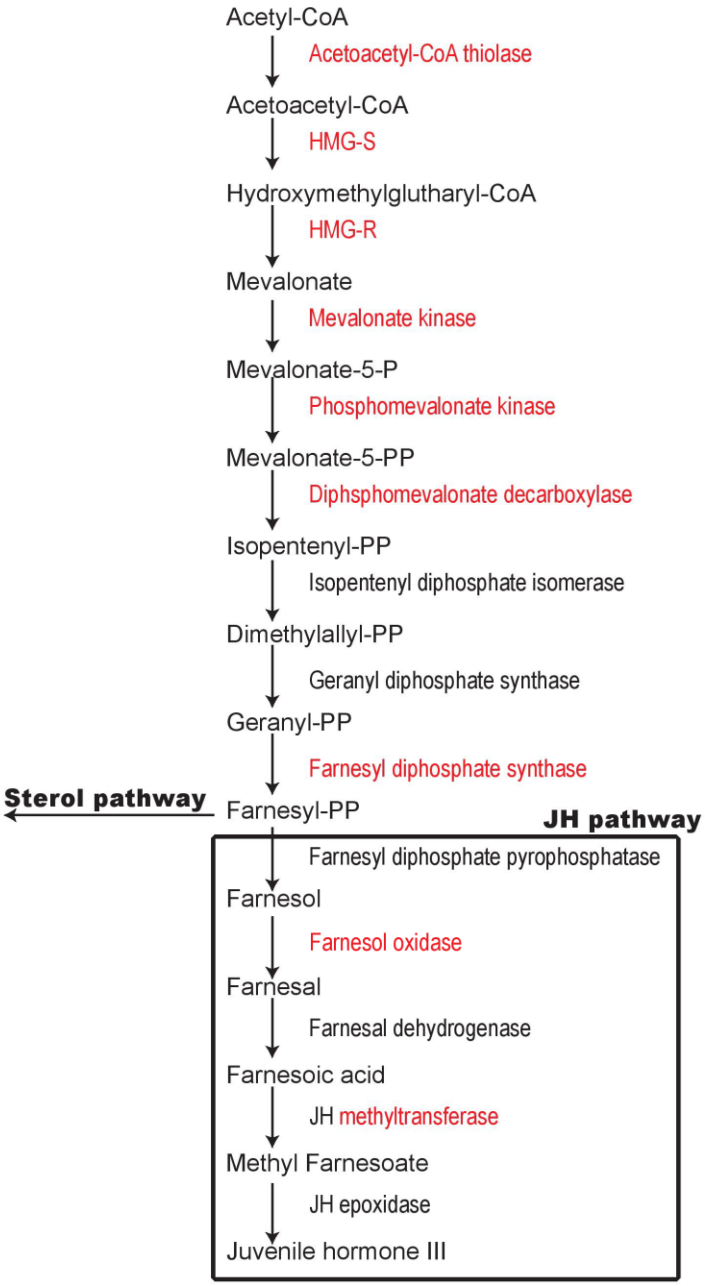

HMG-S, Hydroxymethylglutaryl-CoA synthase
HMG-R, Hydroxymethylglutaryl-CoA reductase
JH, Juvenile Hormone

Supplementary Fig. 18. The mevalonate/farnesoic acid pathway in *Ixodes scapularis.* Genes encoding enzymes highlighted in red were identified in the *I. scapularis* genome. There is no evidence that putative *I. scapularis* methyl transferases are involved in the synthesis of methyl farnesoate. There is no direct evidence for the production of methyl farnesoate or juvenile hormone (JH) in ticks, and no evidence that these compounds, when introduced exogenously, affect tick development and reproduction.

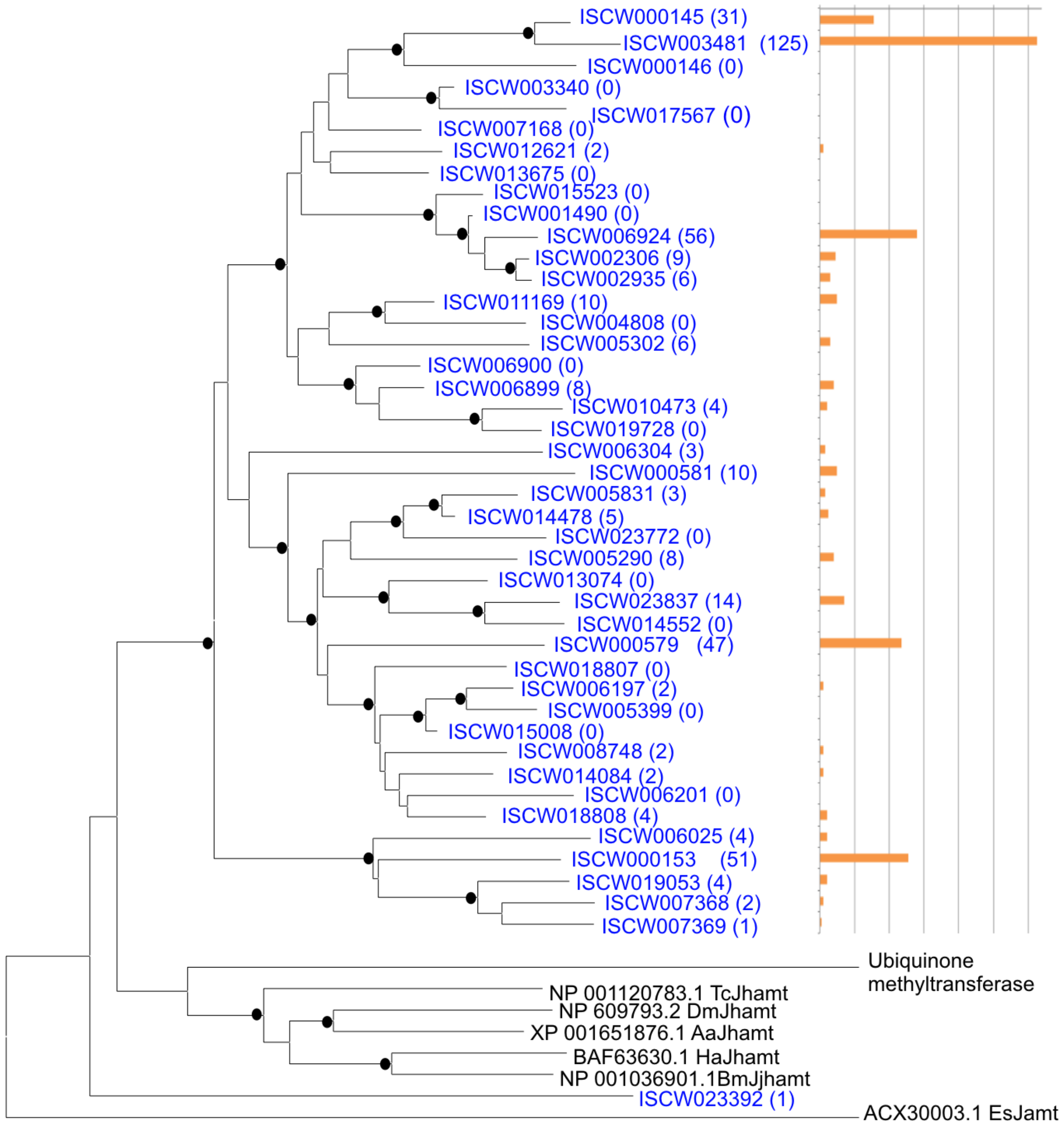

Supplementary Fig. 19. Recent gene expansion for farnesoic acid methyltransferase/methyl transferase in the *Ixodes scapularis* genome showing 44 copies. Blue fonts are for the sequences found in the *I. scapularis* with the frequency of the EST for each predicted gene in the parenthesis and bar graph on the right column. Solid circles at the branching points are for bootstrapping supports with higher than 70% in 1000 replications of the neighbor-joining tree. Insect genes in the orthologous group are from *Tribolium castaneum* (Tc), *Drosophila melanogaster* (Dm), *Aedes aegypti* (Aa), *Helicoverpa armigera* (Ha), *Bombyx mori* (Bm), and *Eriocheir*

sinensis (Es). The VectorBase accession for the predicted protein and corresponding base-pair range of each gene on the *I. scapularis* scaffolds are; ISCW000145, DS624614 (1302..5354); ISCW000146, DS624614 (9631..10170); ISCW000153, DS763941 (182505..186413); ISCW000579, DS947122, (6316..11994); ISCW000581, DS629339, (179176..194485), ISCW001490, DS706167, (10281..31698); ISCW002306, DS932067, (897..5521); ISCW002935, DS768854, (735905..747194); ISCW003340, DS779352, (21734..27575); ISCW003481, DS793841, (18669..25357); ISCW004808, DS674354, (11645..16314); ISCW005290, DS970697, (84452..85469); ISCW005302, DS629339, (117659..129013); ISCW005399, DS777710, (6901..10266); ISCW005831, DS887498, (197112..207263); ISCW006025, DS954326, (27895..30828); ISCW006197, DS748781, (26926..29875); ISCW006201, DS851612, (37761..41988); ISCW006304, DS930042, (10717..12534); ISCW006899, DS690206, (9184..9657); ISCW006900, DS741077, (829..13451); ISCW006924, DS872849, (1313..1855); ISCW007168, DS789606, (16620..25257); ISCW007263, DS768854, (719374..727853); ISCW007368, DS779352, (129774..133039); ISCW007369, DS967436, (35953..41027); ISCW008032, DS652581, (3712..4362); ISCW008748, DS748497, (1567..25229); ISCW010473, DS615618, (20473..34490); ISCW011169, DS751725, (281571..300251); ISCW012621, DS638221, (12578..13060); ISCW013074, DS781271, (1344..5551); ISCW013675, DS781271, (13943..14422); ISCW014084, DS751647, (5328..26009); ISCW014478, DS880071, (28950..39043); ISCW014552, DS977870, (16944..20120); ISCW015008, DS644550, (414041..414787); ISCW015523, DS928935, (5815..7014); ISCW016046, DS972004, (8558..19224); ISCW017567, DS746255, (64199..77852); ISCW018807, DS661924, (43567..48537); ISCW018808, DS735014, (408..7677); ISCW019053, DS710865, (6303..9071); ISCW019728, DS970447, (16214..18413); ISCW023392, DS802122, (55507..58567); ISCW023772, DS938188, (37911..40070); ISCW023837, DS770764, (14572..19070).

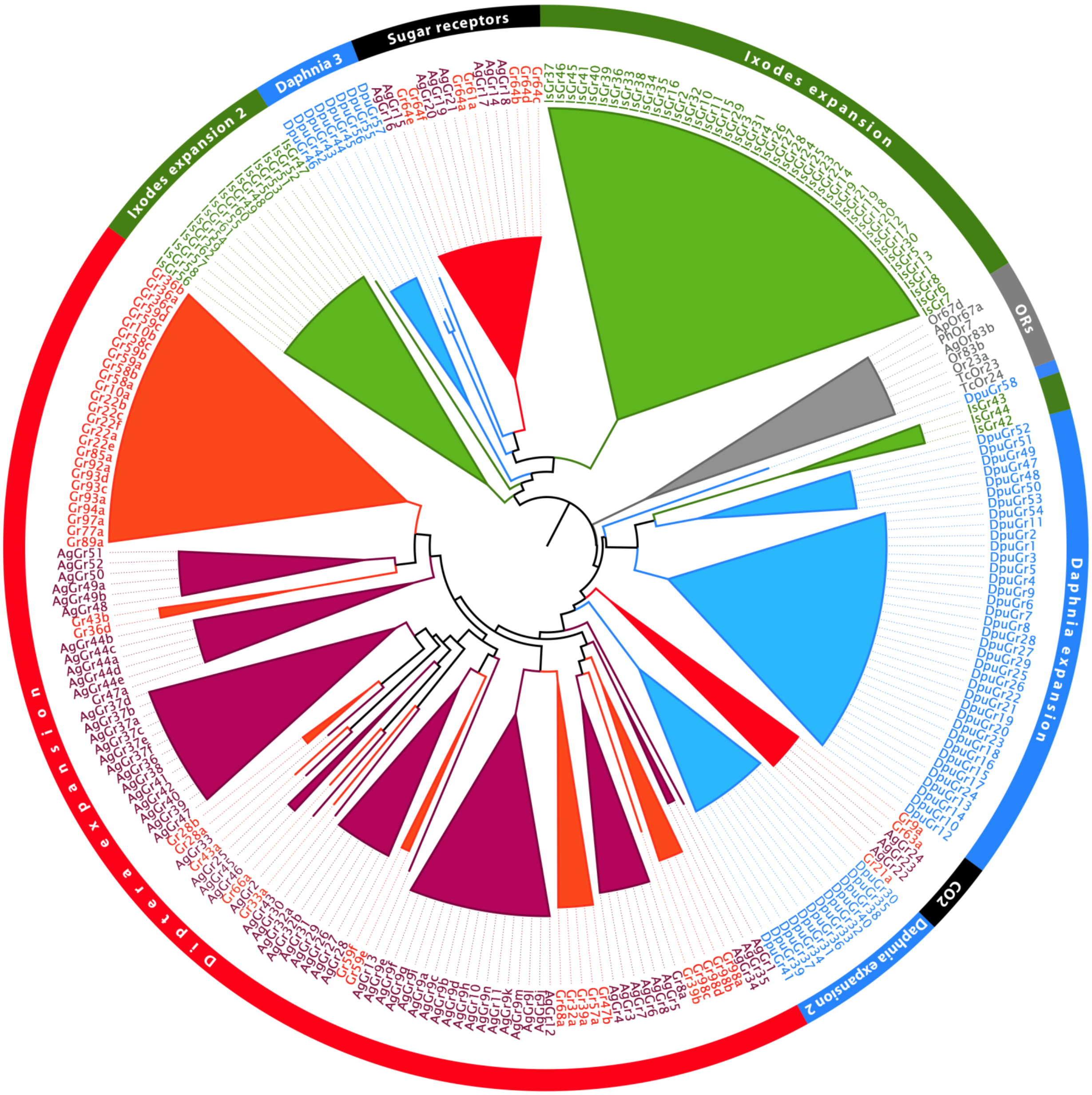

Supplementary Fig. 20. Phylogenetic relationships among gustatory (GRs) and olfactory (ORs) receptors. Protein sequences from *Ixodes scapularis* (green), *Daphnia pulex* (blue), *Drosophila melanogaster* (orange) and *Anopheles gambiae* (maroon). Sugar and CO_2 receptors are highlighted in black. The insect olfactory receptors (grey) include protein sequences of several species: *D. melanogaster* (Or), *Tribolium castaneum* (Tc), *Anopheles gambiae* (Ag), *Pediculus humanus* (Ph), and *Acyrthosiphon pisum* (Ap).

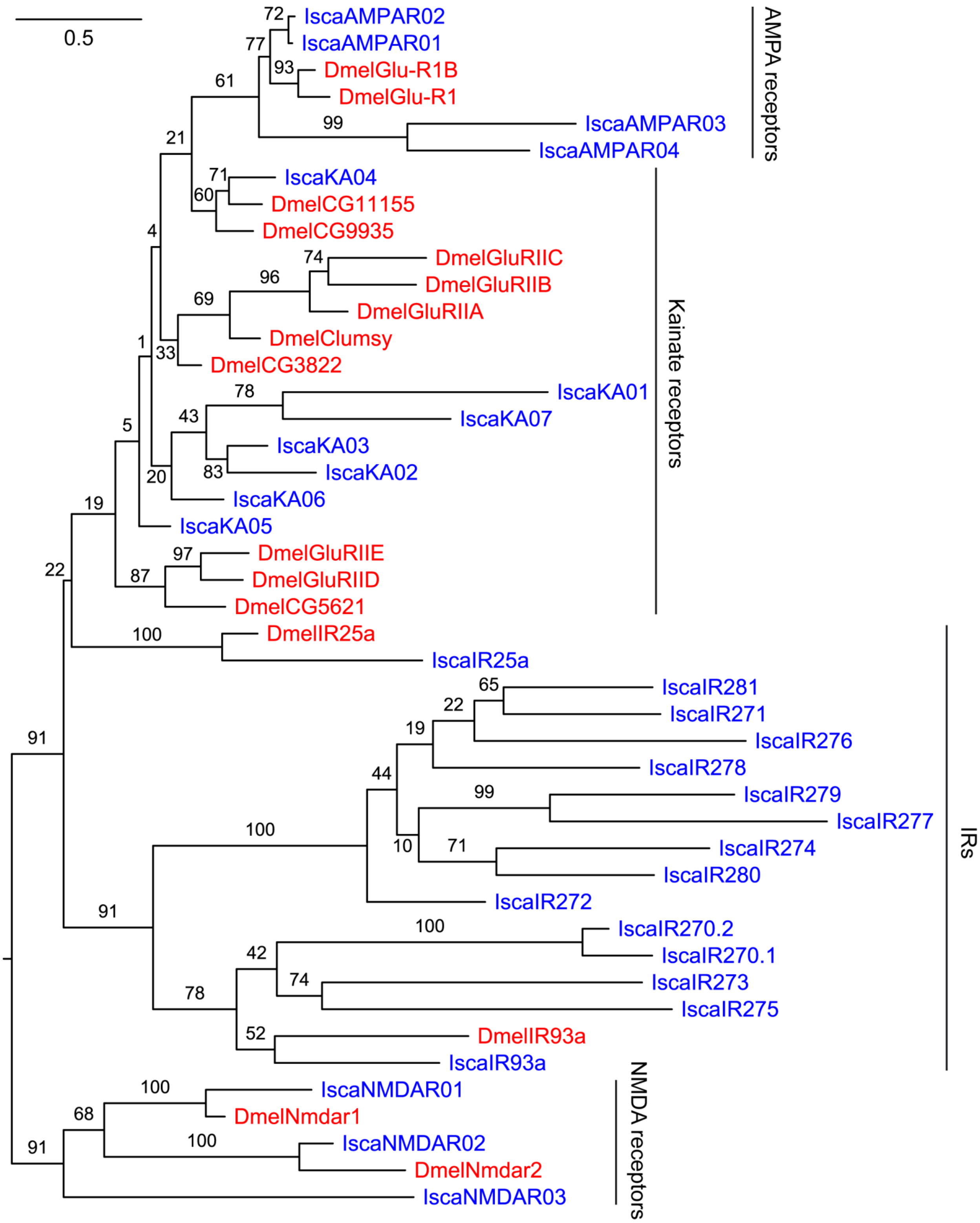

Supplementary Fig. 21. Phylogenetic tree of the *Ixodes scapularis* Ionotropic Receptor (IR) and ionotropic glutamate receptor protein sequences (blue), alongside their *Drosophila melanogaster* (red) orthologs. Different receptor subfamilies of receptors are highlighted with black vertical lines. Protein sequences

were aligned with MUSCLE, and the tree was built with RAxML under the WAG model of substitution with 1000 bootstrap replicates. Bootstrap values for each branch are indicated on the tree. The scale bar represents the number of substitutions per site**.**

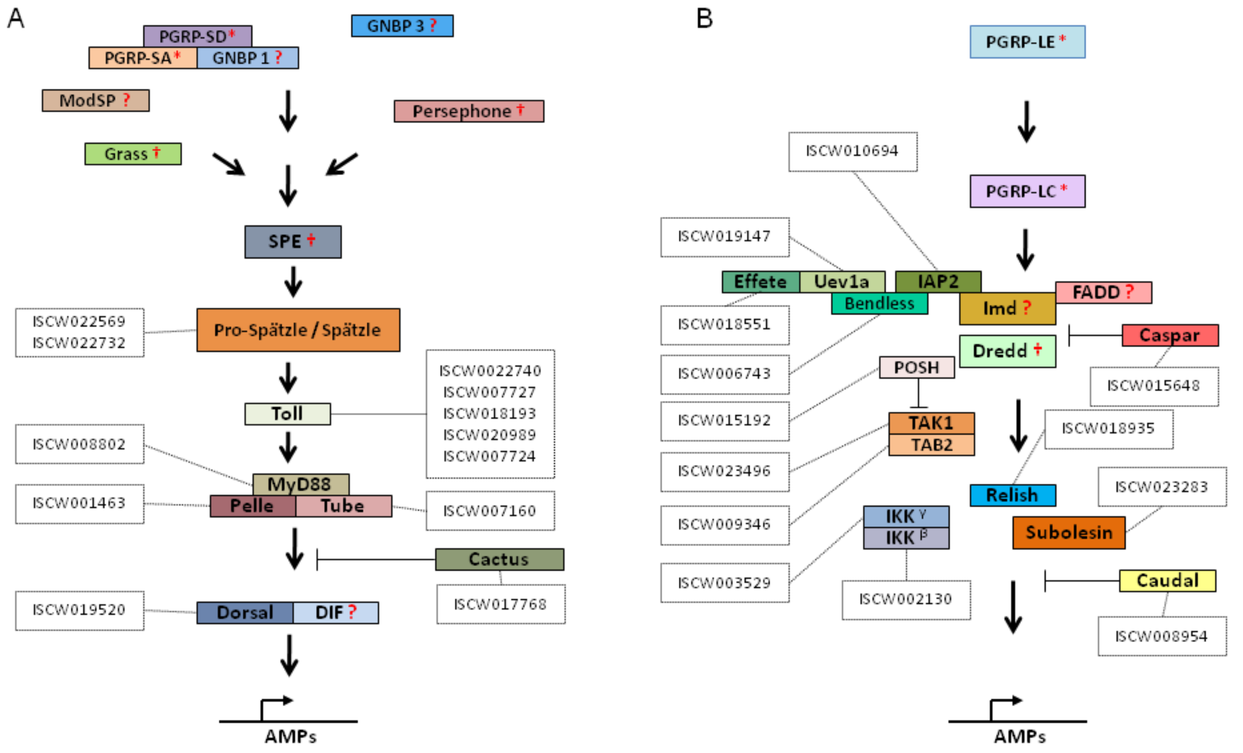

Supplementary Fig. 22. *In silico* analysis of the (a) Toll and (b) IMD pathways in the *Ixodes scapularis* genome. Gene identifiers were obtained from VectorBase (www.vectorbase.org) and compared to the Toll and IMD pathways in *Drosophila melanogaster*, *Anopheles gambiae* and *Aedes aegypti* mosquitoes. Gene identifiers from *I. scapularis* are boxed. Red question marks indicate genes that were not identified in the *I. scapularis* genome. Dagger marks represent sequences for which putative *I. scapularis* homologues were uncovered but cannot be categorized as precise orthologs. Asterisks indicate sequences for which putative *I. scapularis* homologues were uncovered but cannot be categorized at the isoform level.

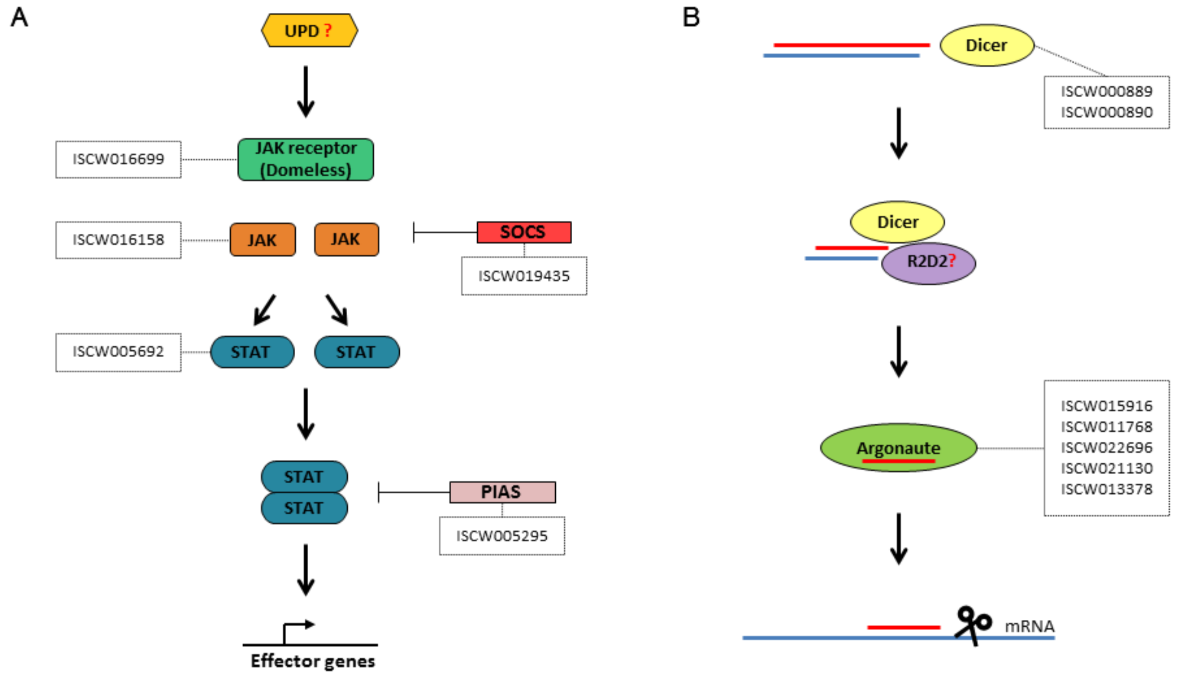

Supplementary Fig. 23. ***In silico*** **analysis of the (a) JAK/STAT and (b) anti-viral RNAi pathways in the** ***I. scapularis*** **genome.** Gene identifiers were obtained from VectorBase (www.vectorbase.org) and compared to the JAK/STAT and RNAi pathways in *Drosophila melanogaster*, *Anopheles gambiae* and *Aedes aegypti* mosquitoes. Gene identifiers from *I. scapularis* are boxed. Red question marks indicate genes that were not identified in the *I. scapularis* genome.

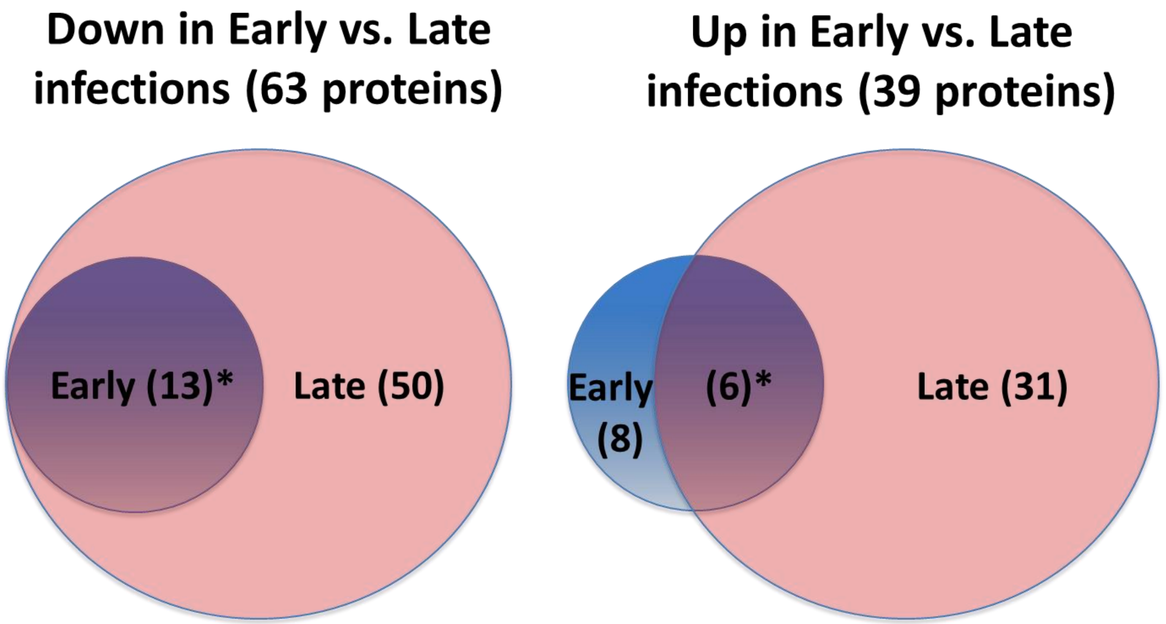

Supplementary Fig. 24. Protein expression in early and late *Anaplasma phagocytophilum* infection of *Ixodes scapularis* ISE6 cells. The Venn diagram shows the number of proteins (in parenthesis) that are over- or under- represented in early versus late infection (*indicates significant overlaps; $p<10^6$).

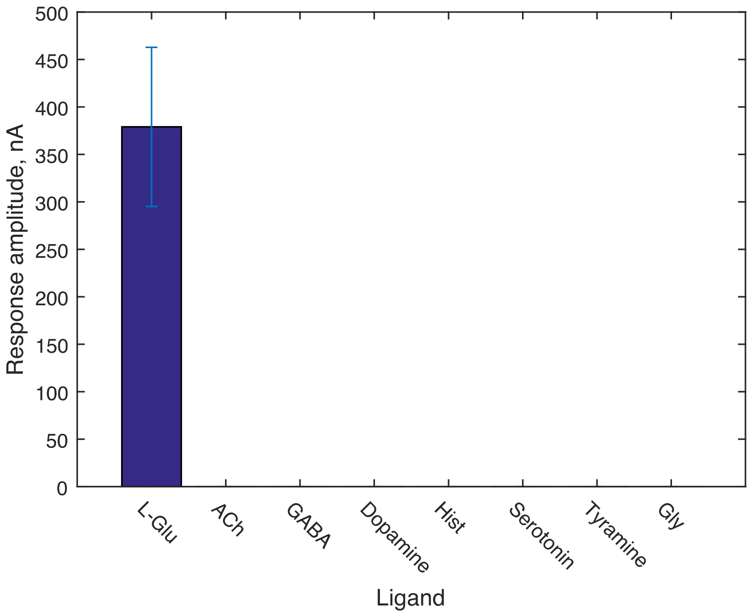

Supplemental Fig. 25. The *Ixodes scapularis* ligand-gated anion channel (KR107244) expressed in *Xenopus laevis* oocytes was exposed in turn to a series of neurotransmitter molecules that have been shown to activate invertebrate ligand-gated anion channels. The transmitters were tested separately at 10^{-4} M on oocytes (n=29, 7, 7, 6, 7, 6, 6 respectively). Only L-glutamate yielded a current. All others tested (acetylcholine (ACh), γ-amino butyric acid (GABA), dopamine, histamine, serotonin, tyramine) were without effect. Glycine, which like GABA activates ligand gated anion channels in mammalian brain was also without effect (n=7). This selectivity for L-glutamate led to the nomenclature IscaGluCl1 for subunit KR107244.

Supplementary Tables

Supplementary Table 1. Cumulative effect of *Ixodes scapularis* IscaW1 assembly intervention.

**Assembly Settings**	**A**	**B**	**C**		**D**	
Input reads	~12,000,000	16,632,252	16,875,697		16,875,697	
Software version	CA 3.1	CA 4.0	CA 4.0		CA 4.0	
Partial overlaps for trim						
+ K-mer seed length	22	16	16	28	16	28
+ seed frequency threshold	default	50	50	1000	50	1000
+ error threshold	6%	6%	6%	6%	6%	6%
+ overlap length threshold	40	40	40	300	40	300
+ detect and trim chimer	yes	yes	no		no	
Full overlaps for unitigs						
+ K-mer seed length	22	22	14		14	
+ seed frequency threshold	default	default	8000		8000	
+ initial error threshold	6%	6%	20%		20%	
+ basecall correction	yes	yes	no		no	
+ final error threshold	3%	3%	13%		13%	
Contig building						
+ assumed genome size	default	default	default		1 Gbp	
+ max error, unitig join	6%	6%	20%		20%	
+ max error, gap close	6%	6%	12%		20%	
**Assembly Results**	**A**	**B**	**C**		**D**	
Contigs						
+ number of contigs	600,000	843,837	474,816		570,640	
+ max bases per contig	30,000	76,172	83,974		117,687	
+ contig N_{50} bases	1,900	1,943	3,116		2,942	
+ mean bases per contig	n/a	1,997	2,554		2,433	
+ mean coverage	2.0	2.3	3.4		3.5	
+ reads incorporated	30%	35%	37%		44%	
+ total contig bases	n/a	1,684,909,012	1,212,614,075		1,388,475,690	
Scaffolds						
+ number of scaffolds	500,000	680,216	327, 135		369, 495	
+ max span per scaffold	250,000	645,492	3,360,897		3,699,225	
+ scaffold N_{50} span	2,200	2,535	22,441		51,551	
+ mean span per scaffold	n/a	3,209	4,606		4,774	
+ total scaffold span	2,150,000,000	2,182, 541,146	1,506,734,076		1,763,920,678	

Four assemblies of *Ixodes scapularis.* Columns A through D summarize four runs of the Celera Assembler software. **Assembly Settings**. Partial overlaps are local alignments between read pairs. Celera Assembler trimmed terminal basecalls of reads based on drop off patterns in the partial overlap collection. Parameter changes during assemblies B and C were designed to enlarge the collection. Assemblies C and D used the union of two collections. Full overlaps are pair-wise alignments that fully cover at least two of the four read ends; they capture dovetail and containment relations. Parameter changes during assemblies C and D were designed to enlarge the collection. Parameter changes during assemblies C and D were designed to reduce sensitivity to high-coverage unitigs (genome size), consensus differences in multiple sequence alignments (unitig join), and basecall differences between trimmed sequences at contig ends (gap close). **Assembly Results.** In the Celera Assembler output, contigs and scaffolds are redundant organizations of the same consensus sequence. Every contig belongs to one scaffold and every scaffold spans one or more contigs. Contigs have positive read coverage at every base. Scaffolds span gaps between contigs where gap size is derived from spanning

mate constraints. Scaffolds also span repetitive regions where a unitig consensus is placed as surrogate for read coverage. Contig N50 is the number of bases of the smallest contig in the minimal set that covers 50% of the assembly's total contig bases. Scaffold N50 is the span smallest scaffold in the minimal set that covers 50% of the assembly's total scaffold span. Mean coverage is the sum of bases after trimming in reads incorporated into contigs divided by total contig bases.

Supplementary Table 2. Size and distribution of DNA on *Ixodes scapularis* IscaW1 scaffolds.

Scaffold Length	No. Scaffolds	Total No. Bases[a]	% Genome in Scaffolds
1Mb – 4 Mb	51	84,551,570	3.6%
100 kb – 999 kb	2,914	743,132,618	32.1%
10 kb – 99 kb	14,397	390,238,447	16.8%
< 10 kb	352,130	545,998,043	23.6%
Total	369,492	1,763,920,678	76%

Calculations are based on the genome size estimate of 2.31 Gb
[a]Based on total scaffold span for column “D” in Supplementary Table 1.

Supplementary Table 3. *Ixodes scapularis* genome annotation IscaW1 statistics

**Transcription units - genes**	*Ixodes scapularis*	*Aedes aegypti* (AaegL1.3)	*Anopheles gambiae* (AgamP3.7)	*Drosophila melanogaster* (FB2012_04)	*Daphnia pulex (Ensembl Genome 71)*	*Tetranychus urticae (Ensembl Genomes 72)*
Total number of protein coding genes	20,486	15,998	12,810	13,937	30,894	18,224
Mean gene length (bp)	10,589	15,456	6,383	6,492	2,116	2,733
Median gene length (bp)	4,259	5,895	2,076	2,088	1,279	1,549
Shortest gene (bp)	95	105	111	117	150	99
Longest gene (bp)	242,297	428,674	365,622	395,988	114,502	104,962
**Exons**						
Total number of exons	89,663	64,752	56,398	74,024	144,872	69,647
Number of mono-exonic genes	5,707	1,874	1,187	2,170	5,149	2,213
Max. no. exons/gene	81	41	67	82	83	55
Median exon length (bp)	219	231	232	246	154	179
**Introns**						
Total number of introns	69,163	52,370	51,024	133,158	113,998	16,041
Percentage of genes with introns	72.10%	88.3%	90.8%	84.4%	83.3%	88%
Mean intron length (bp)	2,284	4,789	1,566	1,568	285	456
Median intron length (bp)	1, 599	145	96	103	76	96
Shortest intron (bp)	15	1	1	1	0 (!)	1
Longest intron (bp)	54, 366	329,294	249,417	141,627	48,487	59,291
**Coding sequences (CDS)**						
Mean CDS length (bp)	855	1,363	1,616	1,977	976	1,074
Median CDS length (bp)	594	1,053	11,191	1,404	702	807
Shortest CDS (bp)	95	81	78	20	150	63
Longest CDS (bp)	15,248	33,987	47,535	68,850	23,331	54,762
**RNAs**						
Non coding RNAs	4,439	1,279	612	474	3,567	n/a
tRNAs	4,402	962	450	314	3,559	n/a
miRNAs	51	84	121	n/a	7	n/a
rRNAs	8	233	41	160	1	n/a
**Miscellaneous Statistics**						
Gene frequency (genes/kb)	1/70	1/82	1/22	1/12	1/6	1/5

Percentage of coding region in genome	6%	-	-	-	-	-
Av. Intergenic region (bp)[a]	80, 410	-	-	-	-	-
Av. Intergenic region (bp)[b]	57, 141	-	-	-	-	-
Intergenic regions GC content	32%	-	-	-	-	-
Coding regions GC content	56%	-	-	-	-	-
Total GC content	NA	*38.2*	*40.9*	*42.5*		-

NA, not available
[a]ten longest scaffolds
[b]global

Supplementary Table 4. Analysis of *Ixodes scapularis* Bacterial Artifical Chromosomes (BACs) showing assembly completeness and mapping to IscaW1 scaffolds.

BAC Name	GenBank Accession	BAC Length (bp)	Sequencing Center	Assembly Status	IscaW1 Scaffold Hits[a]	GenBank IscaW1 Scaffold ID
ISG1-05A01	AC192414	117,688	Broad	1 C	2	DS776359<br>DS810098
ISG1-33A01	AC192415	122,081	Broad	2 OP	3	DS818409<br>DS825617<br>DS768807
ISG1-36A01	AC192416	106,082	Broad	2 OP	1	DS694416
ISG1-40A01	AC192417	102,815	Broad	3 UP	4	DS840034<br>DS872355<br>DS892014<br>DS682990
ISG1-41A01	AC192418	114,701	Broad	3 UP	1	DS907939
ISG1-43A01	AC192419	113,880	Broad	2 OP	multiple	N/A
ISG1-45A01	AC192420	146,997	Broad	19 UP	multiple	N/A
ISG1-49A01	AC192421	137,442	Broad	7 UP	2	DS858616<br>DS746366
ISG1-51A01	AC192422	135,954	Broad	2 OP	1	DS752087
ISG1-53A01	AC192423	109,798	Broad	2 OP	multiple	N/A
ISG1-55A01	AC192424	145,462	Broad	5 UP	1	DS732299
ISG1-60A01	AC192425	145,957	Broad	6 OP	multiple	N/A
ISG1-64A01	AC192426	117,608	Broad	4 UP	1	DS868627
ISG1-66A01	AC192427	98,661	Broad	1 C	1	DS911446
ISG1-67A01	AC192428	109,864	Broad	3 OP	multiple	N/A
ISG1-68A01	AC192429	142,474	Broad	3 UP	multiple	N/A
ISG1-48A01	AC192742	133,074	Broad	1 C	1	DS780529
ISG1-54A01	AC192743	130,937	Broad	8 UP	multiple	N/A
ISG1-61A01	AC192744	115,169	Broad	2 OP	multiple	N/A
ISG1-02A01	AC200531	77,162	Broad	1 C	multiple	N/A
ISG1-01F14	AC205630	95,257	JCVI	1 C	multiple	N/A
ISG1-01P02	AC205631	108,728	JCVI	2 OP	multiple	N/A
ISG1-03K02	AC205632	26,509	JCVI	1 C	multiple	N/A
ISG1-03P02	AC205633	97,928	JCVI	4 OP	multiple	N/A
ISG1-06P02	AC205634	112,417	JCVI	1 C	multiple	N/A
ISG1-11P02	AC205635	104,824	JCVI	1 C	1	DS800715
ISG1-12P02	AC205636	106,974	JCVI	6 OP	4	DS688082<br>DS708137<br>DS966567<br>DS826915
ISG1-14C07	AC205637	132,125	JCVI	3 OP	multiple	N/A
ISG1-15P02	AC205638	100,378	JCVI	1 C	multiple	N/A
ISG1-16P02	AC205639	92,783	JCVI	4 OP	multiple	N/A
ISG1-22P02	AC205640	109,965	JCVI	4 OP	multiple	N/A
ISG1-24P02	AC205641	179,341	JCVI	1 C	multiple	N/A
ISG1-27P02	AC205642	110,473	JCVI	1 C	3	DS859588<br>DS928213<br>DS859588
ISG1-31P02	AC205643	128,247	JCVI	1 C	1	DS891538
ISG1-37P02	AC205644	110,110	JCVI	2 OP	multiple	N/A
ISG1-41M08	AC205645	120,605	JCVI	2 OP	2	DS712833

						DS712833
ISG1-42P02	AC205646	122,242	JCVI	1 C	1	DS840967
ISG1-43E15	AC205647	115,710	JCVI	1 C	multiple	N/A
ISG1-44P02	AC205648	126,904	JCVI	3 OP	multiple	N/A
ISG1-47P02	AC205649	112,049	JCVI	2 OP	multiple	N/A
ISG1-56P02	AC205650	107,316	JCVI	1 C	1	DS636787
ISG1-58P02	AC205651	172,210	JCVI	2 OP	1	DS879425
ISG1-62P02	AC205652	50,437	JCVI	1 C	multiple	N/A
ISG1-63P02	AC205653	108,041	JCVI	1 C	3	DS976271
						DS897480
						DS981194
ISG1-69P02	AC205654	14,567	JCVI	1 C	multiple	N/A

Broad, the Broad Institute of MIT/Harvard; JCVI, J. Craig Venter Institute; C, complete assembly of BAC clone: BAC assembly sequence is complete and ungapped; OP, ordered pieces: the BAC assembly is incomplete but the order of contigs comprising the BAC is known; UP, unordered pieces: the BAC assembly is incomplete and the order of the pieces cannot be deduced basded on read mate pair information; [a]numeric value indicating number of IscaW1 scaffolds that align to the assembled BAC clone; multiple, 10 or more IscaW1 scaffolds align to the sequence of the assembled BAC clone.

Supplementary Table 5. Analysis of gene content of *Ixodes scapularis* BAC clones. The IscaW1 predicted protein sequences were queried against the sequence of assembled BAC clones using BLASTX.

BAC Clone GenBank Accession	BAC length (bp)	Genbank Protein Locus Tag	IscaW1 Gene length (bp)	Hit coordinates on BAC		% ID to annotated IscaW1 protein	Gene coverage (%BAC/IscaW1)	Protein name
				5' end	3' end			
AC205647	115,710	ISCW001627	999	71,697	72,691	96.2	100	hypothetical protein
AC205650	107,316	ISCW001662	1,595	40,314	41,908	100	100	voltage-gated potassium channel
AC205637	132,125	ISCW005308	1,139	1,261	2,400	95.18	99.82	conserved hypothetical protein
AC192428	109,864	ISCW005551	813	51,858	52,672	97.06	100	hypothetical protein
AC205642	110,473	ISCW007049	903	11,042	11,941	95.57	100	hypothetical protein
AC205646	122,242	ISCW007900	912	12,862	13,716	97.19	93.53	conserved hypothetical protein
AC192414	117,688	ISCW008378	1,017	104,265	105,120	96.85	84.17	hypothetical protein
AC205636	106,974	ISCW009194	1,776	104,329	106,149	95.28	100	leucine-rich transmembrane protein, putative
AC205645	120,605	ISCW009445	1,086	36,434	37,293	98.84	79.01	hypothetical protein
AC205637	132,125	ISCW011925	1,182	99,621	100,799	97.88	99.83	hypothetical protein
AC205637	132,125	ISCW011924	909	101,454	102,362	97.14	100	hypothetical protein
AC192418	114,701	ISCW012420	1,188	101,002	102,058	95.84	87.96	polyprotein of retroviral origin
AC205636	106,974	ISCW013209	1,974	51,710	53,682	97.63	100	hypothetical protein
AC205648	126,904	ISCW014746	1,401	75,055	76,459	96.59	99.86	hypothetical protein
AC205653	108,041	ISCW015369	753	94,678	95,428	98.93	99.73	hypothetical protein
AC192428	109,864	ISCW017315	1,113	48,937	49,821	97.3	79.78	hypothetical protein
AC192418	114,701	ISCW018410	1,799	76,283	78,081	98.78	100	conserved hypothetical protein
AC192422	135,954	ISCW019007	1,464	88,949	90,400	96.6	99.8	transmembrane protein C9orf46
AC192422	135,954	ISCW019010	1,083	16,384	17,471	99.17	100	conserved hypothetical protein
AC205645	120,605	ISCW019863	2,507	86,223	88,736	96.07	100	zinc finger protein, putative
AC205645	120,605	ISCW019864	4,307	89,682	93,981	95.79	99.72	zinc finger protein, putative
AC205635	104,824	ISCW019867	6,487	80,684	87,155	98.68	100	zinc finger protein, putative
AC205635	104,824	ISCW019869	1,182	52,935	54,101	97.29	100	zinc finger protein, putative
AC205635	104,824	ISCW019871	1,353	4,616	5,971	99.41	100	carbon-nitrogen hydrolase
AC205646	122,242	ISCW020015	945	111,615	112,559	100	100	hypothetical protein
AC192421	137,442	ISCW021499	843	18,199	19,056	96.5	99.88	hypothetical protein
AC192421	137,442	ISCW021498	897	46,540	47,435	98.11	99.55	hypothetical protein
AC205640	109,965	ISCW024821	1,090	6,451	7,539	99.45	100	hypothetical protein

Supplementary Table 6. Putative microRNA genes identified in the *Ixodes scapularis* genome. MicroRNA gene predictions were consolidated from miRBase[160], miROrtho[161], and VectorBase[162], resulting in a conservative set of 45 miRNAs. Family: assigned based on similarity to miRBase miRNAs. miRBase-ID, miROrtho-ID, VectorBase-ID: resource specific gene identifiers. miRBase-Family: family identifier, if predicted. Chromosome, Start, End, Strand: location in the *I. scapularis* genome or trace reads.

Family	miRBase-ID	miROrtho-ID	VectorBase-ID	miRBase-Family	Scaffold	Start (bp)	End (bp)	Strand
**bantam**	MI0012259	616211	NA	MIPF0000153	DS612599	38772	38872	-
**mir-133**	MI0012266	NA	NA	MIPF0000029	DS613658	228744	228844	-
**mir-7**	MI0012282	615892	NA	MIPF0000022	DS629750	8358	8437	-
**mir-263**	MI0012272	NA	ISCW000811	MIPF0000122	DS633978	93542	93641	+
**mir-263**	NA	616209	ISCW000812	NA	DS633978	112822	112905	+
**mir-96**	MI0012288	NA	ISCW000813	MIPF0000072	DS633978	113211	113315	+
**mir-279**	MI0012274	615761	ISCW000516	MIPF0000184	DS634011	38506	38597	+
**mir-153**	MI0012268	617851	NA	MIPF0000050	DS642248	1296642	1296742	-
**mir-219**	MI0012270	NA	ISCW002511	MIPF0000044	DS658596	21107	21207	-
**mir-315**	MI0012278	NA	NA	MIPF0000141	DS711462	57333	57433	+
**mir-8**	MI0012286	615572	ISCW005313	MIPF0000019	DS755496	12152	12245	-
**mir-2001**	MI0010250	616271	NA	none	DS758004	38911	38988	-
**mir-2**	MI0012276	NA	NA	MIPF0000049	DS799611	45157	45257	-
**mir-2**	MI0012277	616921	NA	MIPF0000049	DS799611	45547	45647	-
**mir-71**	MI0012283	616923	NA	MIPF0000278	DS799611	45689	45789	-
**mir-184**	MI0012269	NA	NA	MIPF0000059	DS803854	570	670	-
**mir-1**	MI0012261	NA	ISCW019387	MIPF0000038	DS811420	416289	416380	+
**mir-1905**	NA	616494	NA	NA	DS833022	58235	58298	-
**mir-124**	MI0012265	NA	ISCW009604	MIPF0000021	DS840700	85144	85244	+
**none**	MI0015941	NA	NA	none	DS841188	7191	7293	+
**mir-137**	MI0012267	617785	NA	MIPF0000106	DS847994	158277	158377	-
**mir-276**	MI0016443	616110	NA	none	DS848078	34717	34803	-
**mir-335**	NA	618522	NA	NA	DS850031	1991	2088	+
**mir-1993**	MI0015940	NA	NA	none	DS862055	102	182	+
**mir-1175**	NA	616890	NA	NA	DS874548	51760	51844	-
**mir-750**	MI0012284	616924	NA	MIPF0000796	DS874548	52964	53064	-
**mir-9**	MI0012285	NA	NA	MIPF0000014	DS885551	436490	436590	+
**mir-317**	MI0012279	617989	NA	MIPF0000144	DS891538	57423	57523	-
**mir-iab-4**	MI0016445	617926	NA	MIPF0000151	DS891538	738905	739003	-
**mir-iab-4**	NA	617910	NA	NA	DS891538	738924	738992	+
**mir-10**	MI0012262	NA	NA	MIPF0000033	DS891538	2780812	2780883	+

**mir-993**	MI0012289	617990	NA	MIPF0000698	DS891538	3285820	3285911	-
**mir-67**	MI0012281	617852	NA	MIPF0000293	DS911299	1700291	1700391	-
**mir-87**	MI0012287	NA	NA	MIPF0000152	DS929532	41471	41571	-
**mir-375**	MI0012280	NA	NA	MIPF0000114	DS929532	41471	41571	-
**mir-87**	NA	617584	NA	NA	DS929532	41890	41991	-
**mir-12**	MI0012264	NA	NA	MIPF0000181	DS942119	45739	45839	-
**mir-305**	MI0016444	616960	NA	MIPF0000158	DS945001	228258	228352	-
**mir-275**	MI0012273	617124	NA	MIPF0000187	DS945001	243239	243339	-
**mir-190**	NA	616305	NA	NA	DS969850	171528	171624	+
**mir-125**	NA	NA	ISCW023847	NA	DS978597	217027	217099	-
**mir-99**	MI0012263	NA	ISCW023848	MIPF0000025	DS978597	252465	252565	-
**let-7**	MI0012260	NA	NA	MIPF0000002	gnl|ti|1145246679	601	700	+
**mir-29**	MI0012275	NA	NA	MIPF0000009	gnl|ti|1308393763	736	831	+
**mir-252**	MI0012271	NA	NA	MIPF0000285	gnl|ti|1711070620	757	857	+

Supplementary Table 7. Proportions of shared intron positions across 12 animal species. Examining conservation of intron positions between *ISCAP*, *DPULE* and either the five insects (*PHUMA*, *NVITR*, *TCAST*, *AGAMB*, *DMELA*) or the five non-arthropods (*NVECT*, *HSAPI*, *MMUSC*, *GGALL*, *DRERI*) reveals that greater than 10 times more intron positions are shared exclusively between at least one of the outgroup species (Cnidaria or Vertebrata) and *ISCAP*, compared to *DPULE* (13.80% compared to 1.08%). Conversely, *DPULE* shares about 4 times more intron positions exclusively with insects (2.34% compared to 0.58%). The percentages shown in Fig. B are the mean values from the numbers of shared or unique positions out of the total number of intron positions (4,621 SSC and 13,459 RSC) as detailed in the table. Abbreviations: *NVECT*, *Nematostella vectensis*; *HSAPI*, *Homo sapiens*; *MMUSC*, *Mus musculus*; *GGALL*, *Gallus gallus*; *DRERI*, *Danio rerio*; *ISCAP*, *Ixodes scapularis*; *DPULE*, *Daphnia pulex*; *PHUMA*, *Pediculus humanus*; *NVITR*, *Nasonia vitripennis*; *TCAST*, *Tribolium castaneum*; *AGAMB*, *Anopheles gambiae*; *DMELA*, *Drosophila melanogaster*.

Intron Positions	SSC	RSC	SSC%	RSC%	Mean%
**OUT ONLY**	656	1776	14.20	13.20	13.70
**INS ONLY**	128	403	2.77	2.99	2.88
**OUT-*ISCAP* ONLY**	659	1795	14.26	13.34	13.80
**OUT-*DPULE* ONLY**	47	155	1.02	1.15	1.08
**INS-*ISCAP* ONLY**	24	85	0.52	0.63	0.58
**INS-*DPULE* ONLY**	108	314	2.34	2.33	2.34
**OUT-INS-*ISCAP* ONLY**	330	1116	7.14	8.29	7.72
**OUT-INS-*DPULE* ONLY**	180	456	3.90	3.39	3.64
**OUT-*ISCAP-DPULE* ONLY**	68	180	1.47	1.34	1.40
**INS-*ISCAP-DPULE* ONLY**	27	52	0.58	0.39	0.49
***ISCAP-DPULE* ONLY**	12	23	0.26	0.17	0.22
**OUT-INS ONLY**	239	646	5.17	4.80	4.99
**OUT-INS-*ISCAP-DPULE***	432	1169	9.35	8.69	9.02
***NVECT* ONLY**	476	1382	10.30	10.27	10.28
***HSAPI* ONLY**	3	12	0.06	0.09	0.08
***MMUSC* ONLY**	3	8	0.06	0.06	0.06
***GGALL* ONLY**	37	124	0.80	0.92	0.86
***DRERI* ONLY**	40	95	0.87	0.71	0.79
***ISCAP* ONLY**	154	501	3.33	3.72	3.53
***DPULE* ONLY**	502	1579	10.86	11.73	11.30
***PHUMA* ONLY**	138	459	2.99	3.41	3.20
***NVITR* ONLY**	136	431	2.94	3.20	3.07
***TCAST* ONLY**	87	302	1.88	2.24	2.06
***AGAMB* ONLY**	48	164	1.04	1.22	1.13
***DMELA* ONLY**	87	232	1.88	1.72	1.80
**Totals**	4621	13459	100	100	100

OUT: at least one of 5 outgroup species, *NVECT*, *HSAPI*, *MMUSC*, *GGALL*, *DRERI.*
INS: at least one of 5 insect species, *PHUMA*, *NVITR*, *TCAST*, *AGAMB*, *DMELA.*
SSC: strict single-copy; RSC: relaxed single-copy**.**

Supplementary Table 8. Proportions of shared *Ixodes scapularis* intron positions. Examining pairwise conservation of intron positions between *ISCAP* and each of the other eleven species shows the greatest sharing with the non-arthropods (*NVECT*, *HSAPI*, *MMUSC*, *GGALL*, *DRERI*): about 3 times more than with *AGAMB* and *DMELA*, and about 1.5-1.8 times more than with *DPULE*, *PHUMA, NVITR, and TCAST*. Abbreviations: *NVECT*, *Nematostella vectensis*; *HSAPI*, *Homo sapiens*; *MMUSC*, *Mus musculus*; *GGALL*, *Gallus gallus*; *DRERI*, *Danio rerio*; *ISCAP*, *Ixodes scapularis*; *DPULE*, *Daphnia pulex*; *PHUMA*, *Pediculus humanus*; *NVITR*, *Nasonia vitripennis*; *TCAST*, *Tribolium castaneum*; *AGAMB*, *Anopheles gambiae*; *DMELA*, *Drosophila melanogaster*.

ISCAP	**ALL SSC %**	**ALL RSC %**	**SHARED SSC %**	**SHARED RSC %**
NVECT	29.86	30.07	35.01	35.67
HSAPI	31.84	32.3	33.13	33.82
MMUSC	31.83	32.03	33.12	33.54
GGALL	31.71	32.08	33.28	33.92
DRERI	31.84	32.18	33.43	33.94
DPULE	17.49	16.09	22.22	21.04
PHUMA	20.89	21.12	23.3	23.92
NVITR	19.5	19.92	21.79	22.57
TCAST	17.02	16.62	18.85	18.72
AGAMB	10.76	11.09	11.85	12.39
DMELA	10.31	10.85	11.57	12.25

SSC: strict single-copy, RSC: relaxed single-copy.
ALL: *ISCAP*-OTHER Shared / *ISCAP*-OTHER Total Intron Positions.
SHARED: *ISCAP*-OTHER Shared / *ISCAP*-OTHER Total Non-Unique Intron Positions.

Supplementary Table 9. Intron presence, gain, and loss estimates across the 12 animal species phylogeny. Intron presence, gain, and loss estimates across the phylogeny for the strict (SSC) and relaxed (RSC) sets of orthologs using Dollo Parsimony (DP) and Posterior Probability (PP) methods of the MALIN suite for maximum likelihood analysis of intron evolution in eukaryotes[33]. The normalized numbers and the species phylogeny with all named nodes are presented in Supplementary Fig. 9. Abbreviations: *NVECT*, *Nematostella vectensis*; *HSAPI*, *Homo sapiens*; *MMUSC*, *Mus musculus*; *GGALL*, *Gallus gallus*; *DRERI*, *Danio rerio*; *ISCAP*, *Ixodes scapularis*; *DPULE*, *Daphnia pulex*; *PHUMA*, *Pediculus humanus*; *NVITR*, *Nasonia vitripennis*; *TCAST*, *Tribolium castaneum*; *AGAMB*, *Anopheles gambiae*; *DMELA*, *Drosophila melanogaster*.

	**SSC30: 4621 sites**						**RSC30: 13459 sites**					
	**DP**			**PP**			**DP**			**PP**		
**Branch/Leaf**	present	gain	loss	present	gain	loss	present	gain	loss	present	gain	loss
DMELA	506	87	165	506	94	242	1492	232	447	1492	236	743
**Diptera**	584	38	307	654	99	317	1707	125	834	2000	352	826
AGAMB	487	48	145	487	52	219	1420	164	451	1420	133	713
**Diptera-Coleoptera**	853	12	359	872	25	357	2416	52	1052	2473	110	1107
TCAST	768	87	172	768	105	209	2223	302	495	2223	362	612
**Holometabola**	1200	16	218	1203	0	175	3416	45	648	3470	0	486
NVITR	1053	136	283	1053	187	337	3011	431	836	3011	564	1023
**Insecta**	1402	62	127	1378	47	11	4019	181	358	3957	93	0
PHUMA	1118	138	422	1118	170	430	3284	459	1194	3284	562	1235
**Pancrustacea**	1467	108	659	1342	122	1051	4196	314	1795	3864	421	2727
DPULE	1376	502	593	1376	614	580	3928	1579	1847	3928	1911	1847
**Arthropoda**	2018	63	422	2271	86	302	5677	160	1043	6170	128	806
ISCAP	1706	154	466	1706	148	713	4921	501	1257	4921	524	1773
**Coelomata**	2377	279	0	2487	398	14	6560	866	0	6848	1205	112
DRERI	2392	40	20	2392	41	23	6581	95	65	6581	100	66
**Vertebrata**	2372	231	236	2374	183	295	6551	716	725	6547	593	894
GGALL	2334	37	44	2334	37	44	6552	124	82	6552	129	80
**Tetrapoda**	2341	3	34	2341	1	34	6510	15	56	6504	9	52
MMUSC	2337	3	4	2337	3	4	6380	8	112	6380	10	113
**Mammalia**	2338	0	3	2338	0	3	6484	2	28	6483	2	23
HSAPI	2336	3	5	2336	3	5	6454	12	42	6454	12	41
**Metazoa**	2098	NA	NA	2103	NA	NA	5694	NA	NA	5756	NA	NA
NVECT	2574	476	0	2574	471	0	7076	1382	0	7076	1320	0

Supplementary Table 10. Comparisons of intron length distributions across 12 animal species. Comparison of intron lengths among the 12 species for the Strict Single-Copy (SSC) and Relaxed Single-Copy (RSC) sets of orthologs. Intron counts, their median and mean lengths, and the p-values from Wilcoxon tests that compare the length distributions are presented for 1. all informative introns, 2. informative introns found in *ISCAP* and *DPULE* and at least one non-arthropod and at least one insect, 3. informative introns shared between *ISCAP* and each of the other species. The length distributions are presented as boxplots in Supplementary Fig. 7 and the species for which the shared site data are presented in Fig. 3C (main text) are indicated with an asterisk (*). Abbreviations: P-Wilcox, paired Wilcoxon test; *NVECT*, *Nematostella vectensis*; *HSAPI*, *Homo sapiens*; *MMUSC*, *Mus musculus*; *GGALL*, *Gallus gallus*; *DRERI*, *Danio rerio*; *ISCAP*, *Ixodes scapularis*; *DPULE*, *Daphnia pulex*; *PHUMA*, *Pediculus humanus*; *NVITR*, *Nasonia vitripennis*; *TCAST*, *Tribolium castaneum*; *AGAMB*, *Anopheles gambiae*; *DMELA*, *Drosophila melanogaster*.

SSC	**1. All: 4621 sites**				**2. Shared: 432 sites**				**3. *ISCAP*-shared**			
	**Count**	**Median**	**Mean**	**Wilcox**	**Count**	**Median**	**Mean**	**Wilcox**	**Count**	**Median**	**Mean**	**P-Wilcox**
NVECT	2574	404.0	635.0	<2.2e-16	371	421.0	684.8	<2.2e-16	1278	401.0	628.7	<2.20e-16
HSAPI	2336	1386.0	2998.1	6.60e-05	399	1474.0	3575.0	8.02e-03	1287	1385.0	3169.0	1.35e-05
MMUSC	**2337**	**1128.0**	**2111.0**	**9.22e-02**	**401**	**1288.0**	**2550.0**	**6.14e-01**	**1287**	**1191.0**	**2257.0**	**6.23e-01**
GGALL	2334	665.5	1474.7	<2.2e-16	398	703.5	1473.0	1.43e-07	1281	685.0	1364.0	<2.20e-16
DRERI	2392	666.5	1548.1	<2.2e-16	407	861.0	1819.6	3.88e-05	1305	713.0	1589.0	1.98e-13
ISCAP	**1706**	**1223.5**	**2053.3**	**NA**	**432**	**1194.5**	**2187.7**	**NA**	***1552***	***1205.5***	***2043.6***	**NA**
DPULE	1376	66.0	91.5	<2.2e-16	432	66.0	99.7	<2.2e-16	539	66.0	94.9	<2.20e-16
PHUMA	1118	87.0	143.3	<2.2e-16	313	90.0	160.3	<2.2e-16	590	88.0	141.3	<2.20e-16
NVITR	1053	81.0	357.8	<2.2e-16	304	81.0	348.8	<2.2e-16	538	81.0	268.3	<2.20e-16
TCAST	768	51.0	435.6	<2.2e-16	246	50.0	436.2	<2.2e-16	421	50.0	357.1	<2.20e-16
AGAMB	487	78.0	539.7	<2.2e-16	146	77.0	557.4	<2.2e-16	236	78.0	534.7	<2.20e-16
DMELA	506	62.0	351.3	<2.2e-16	143	62.0	183.4	<2.2e-16	228	62.0	408.2	<2.20e-16

RSC	**1. All: 13459 sites**				**2. Shared: 1169 sites**				**3. *ISCAP*-shared**			
	**Count**	**Median**	**Mean**	**Wilcox**	**Count**	**Median**	**Mean**	**Wilcox**	**Count**	**Median**	**Mean**	**P-Wilcox**
NVECT	7076	407.0	645.9	<2.2e-16	976	426.5	662.6	<2.2e-16	3608	408.0	648.8	<2.20e-16
HSAPI*	6454	1483.0	3687.2	<2.2e-16	1060	1745.5	4723.6	7.93e-12	3674	1552.0	4042.0	<2.20e-16
MMUSC*	**6380**	**1224.5**	**2743.9**	**3.26e-01**	**1050**	**1447.0**	**3751.0**	**3.68e-04**	**3620**	**1288.0**	**3052.5**	**1.98e-02**
GGALL	6552	693.5	1700.0	<2.2e-16	1070	761.0	2217.1	2.10e-11	3680	727.0	1815.0	<2.20e-16
DRERI	6581	728.0	1787.0	<2.2e-16	1084	935.0	2204.0	1.81e-07	3701	785.0	1917.0	<2.20e-16
ISCAP*	**4921**	**1213.0**	**2034.0**	**NA**	**1169**	**1204.0**	**2125.0**	**NA**	***4420***	***1188.0***	***1999.0***	**NA**
DPULE*	3928	66.0	86.9	<2.2e-16	1169	67.0	89.2	<2.2e-16	1424	67.0	86.9	<2.20e-16
PHUMA*	3284	88.0	155.8	<2.2e-16	861	91.0	188.4	<2.2e-16	1733	89.0	163.3	<2.20e-16
NVITR*	3011	82.0	565.1	<2.2e-16	816	82.0	436.0	<2.2e-16	1580	82.0	419.2	<2.20e-16
TCAST	2223	51.0	459.9	<2.2e-16	628	50.0	374.4	<2.2e-16	1187	50.0	368.4	<2.20e-16
AGAMB	1420	80.5	473.8	<2.2e-16	408	81.0	628.9	<2.2e-16	703	80.0	574.6	<2.20e-16
DMELA*	1492	63.0	256.9	<2.2e-16	383	64.0	305.3	<2.2e-16	696	63.0	328.5	<2.20e-16

Supplementary Table 11. Summary of gene group counts using OrthoMCL clustering of reciprocal best hit BLASTP.

Species	oGene	nGroup	UDup	Orth1	OrDup	OrGrp	OrMis1
**No. in Chelicerata**							
Ixodes scapularis	11817	8594	2079	7239	2499	7945	110
Tetranychus urticae	11194	6685	4000	5227	1967	5937	147
Dermacentor variabilis	41142	11157	31914	4076	5152	4865	1189
**No. in Crustacea**							
Daphnia magna	38049	20334	20313	9092	8644	12354	5
Daphnia pulex	27825	14456	10784	10410	6631	11866	10
Pandalus latirostris	28999	13397	19164	5330	4505	7017	122
**No. in Insecta**							
Acrythosiphon pisum	24954	9724	8848	6778	9328	8068	43
Drosophila melanogaster	11523	8449	2519	6925	2079	7627	38
Schistocerca gregaria	26797	14280	14667	6826	5304	8705	15
Tribolium castaneum	12523	8919	2119	7584	2820	8429	29
Nasonia vitripennis	18662	9605	7544	7480	3638	8259	23
**No. in Vertebrate Outgroup Species**							
Homo sapiens	18820	11829	2310	8282	8228	11089	61
Danio rerio	19916	11777	3130	8139	8647	11171	84

oGene = number of genes with reciprocal best hits used by orthomcl.
nGroup = number of gene family groups (2+genes), orthology + species-unique.
OrGrp = count of ortho groups (nGroup = OrGrp + unique paralog groups).
UDup = species-unique duplicated paralog genes.
Orth1 = count of single ortho gene.
OrDup = count of duplicated ortho gene.
OrMis1 = groups missing gene all others have (ignoring human)

Data sources:
Chelicerata: *Ixodes scapularis* 2011, https://www.vectorbase.org/; *Tetranychus urticae*, http://www.nature.com/nature/journal/v479/n7374/pdf/nature10640.pdf; *Dermacentor variabilis*, http://www.ncbi.nlm.nih.gov/pubmed/20060044;
Crustacea: *Daphnia pulex* 2010, http://arthropods.eugenes.org/EvidentialGene/daphnia/daphnia_genes2010/; *Daphnia magna* 2011, pre-release gene set; *Pandalus latirostris*, http://www.ncbi.nlm.nih.gov/pubmed/22016807;
Insecta: *Acrythosiphon pisum* 2011, http://arthropods.eugenes.org/EvidentialGene/pea_aphid2/genes-bestof3/;
Drosophila melanogaster, NCBI RefSeq 2011; *Locusta migratoria* http://www.ncbi.nlm.nih.gov/pubmed/21209894
Tribolium casteneum, UniProt 2011; *Nasonia vitripennis* 2012, http://arthropods.eugenes.org/EvidentialGene/nasonia/;
Vertebrates: *Homo sapiens*, NCBI RefSeq 2011; *Danio rerio*, NCBI RefSeq 2011

Supplementary Table 12. Summary of tandem repeats identified from an *Ixodes scapularis* small insert genomic DNA library and FISH-based physical mapping to ISE18 cell line chromosomes.

Clone ID	Repeat Family	Repeat Length(s) (bp)	Copy Number(s) in End-sequence	Hybridization Intensity	Hybridization Description
A-02	ISR-2	95	12.8	NC	NC
A-03	-	21, 26, 49	5.7, 13.8, 1.9	NS	NS
A-07	ISR-3	376	2.5	S	S
A-12	-	14, 48	2.1, 2	NS	NS
A-17	-	118	1.9	M	S/D
A-22	-	None (control)	NA	NS	NS
B-01	-	35, 70	3.2,2.8	S	D
B-08	ISR-2a	95	17.8	S	S
B-11	-	4, 4, 12	40.3, 22, 12	W	D
B-13	ISR-2c	97	15.9	S	S
B-20	-	11, 25, 25, 39	2.4, 3.2, 9.1, 4.2	S	D
B-22	-	None (control)	NA	NS	NS
B-24	ISR-2b	96	10.5	S	S
C-02	-	41, 83	7.4, 3.7	NS	NS
C-07	ISR-2c	97	13.3	S	S
C-12	-	63	12	NS	NS
C-13	-	26,31	2,2.6	M	D
C-20	ISR-2a	95	13.1	NC	NC
D-02	ISR-2c	97	13.8	NC	NC
D-03	-	2, 32	58.5, 2.3	W	D
D-12	-	98	2	W	D
D-19	ISR-3	386	2.1	NC	NC
D-23	ISR-2b	96	17.4	S	S
E-01	ISR-2a	95	14.4	NC	NC
E-09	ISR-2a	95	12.7	NC	NC
E-18	ISR-2d	99	12.9	S	S
E-19	ISR-2a	95	3.7	NC	NC
E-20	-	2	54.5	W	D
E-21	-	196	9.1	M	S/D
E-23	-	36	5.1	W	D
E-24	ISR-2d	99	12.3	S	S
F-11	-	16, 33	2.1, 2.1	NS	NS
F-12	-	2, 46	30, 5.3	W	D
G-04	ISR-2d	99	13.8	S	S
G-14	ISR-2a	95	12.8	NC	NC
G-17	-	37, 14	2.6, 3	W	D
G-20	ISR-3	385	2.9	S	S
H-16	ISR-1	90, 179, 446	11.6, 5.8, 2	M	S
H-18	-	44	3.4	NS	NS
H-19	-	16, 243	2.1, 2.7	M	D
H-21	ISR-2d	99	13.6	S	S
H-22	ISR-2a	95	15.8	NC	NC
H-24	-	15, 17, 17, 44, 59	9.9, 2.6, 2.4, 3.2, 2.5	W	D
I-01	-	40, 41, 41, 81	2.5, 4.5, 3.6 1.9	M	D
I-06	ISR-2a	95	12.1	NC	NC
I-22	ISR-2a	95	10.6	NC	NC
I-24	ISR-2a	95	17	S	S

J-02	-	14, 40	7.9, 2	NS	NS
J-08	-	22, 42	2.7,2	NS	NS
J-15	-	77	2	W	D
K-01	ISR-2a	95	11	NC	NC
K-02	ISR-2a	95, 284	6.1, 2	NC	NC
K-05	ISR-2b	96	18.5	S	S
K-13	-	2, 40, 32, 2	31.5, 1.9, 2.5	M	D
L-01	-	18	10.4	NS	NS
L-10	ISR-2a	95	17.1	S	S
L-23	-	13, 17, 29	1.9, 9.5, 2	NS	NS
M-04	ISR-2a	95	16.7	S	S
M-10	-	135	8.2	W	D
M-16	ISR-2c	97	12.9	NC	NC
M-17	ISR-2c	97	12.8	NC	NC
M-19	ISR-2a	95	10.5	NC	NC
M-21	ISR-2a	95	4.7	NC	NC
M-23	-	2, 21	49.5, 2.4	M	D
N-01	-	71	4.5	M	D
N-07	ISR-2a	95	13.5	NC	NC
N-11	ISR-2c	97	14.9	NC	NC
N-17	-	6	16.7	M	D
N-19	ISR-2a	95	15.3	NC	NC
O-03	-	207, 413	8.4, 4.2	S	S/D
O-10	ISR-2a	95	15.2	NC	NC
O-14	ISR-2d	99	10.9	S	S
O-15	-	11, 32, 32	2.8, 3.8, 3.6	NS	NS
O-21	-	6, 17, 18, 78	8, 3.4, 13.8, 2	S	D
O-24	-	155	3.7	W	D
P-03	-	27, 53, 83	12.4, 5.9 2.5	W	D
P-07	-	21	5	NS	NS
P-14	ISR-2c	97	16.1	NC	NC

Hybridization intensity: S=strong; M=moderate; W=weak; NS=no signal, NC=not conducted. Hybridization descriptions: S=specific; D=dispersed; NS=no signal; NC=not conducted. The “Repeat Family” column indicates which of the tandem-repeat containing clones that were classified into ISR-1-3[41] or that contained different tandem repeats that remain unclassified (-).

Supplementary Table 13. Summary of transposable elements identified in the *Ixodes scapularis* genome.

TE name	Elements per family	Copy Number	Base pairs	% Genome
**Class I**				
**LTR retrotransposons**	**41**	**29462**	**11383395**	**0.64**
Gypsy	37	28997	11189309	0.63
Pao-Bel	4	465	194086	0.01
**Non-LTR retrotransposons**	**530**	**606602**	**118212063**	**6.70**
CR1	128	133579	26561455	1.50
I	43	49040	9402964	0.53
Jockey	2	6	3896	0.00
L1	171	201621	36843465	2.09
L2	65	57882	11639922	0.66
R1	7	430	61781	0.00
R4	2	1924	582360	0.03
Other Non-LTR	112	162120	33116220	1.88
**Penelope**				
Penelope	132	94326	19113444	1.08
**Class II**				
**DNA transposons**	**254**	**293281**	**54005181**	**3.06**
hAT	52	32713	7362901	0.42
Merlin	3	652	160598	0.01
Mutator	10	397	99572	0.01
P	35	28807	4859952	0.28
PIF	30	38609	7054230	0.40
piggyBac	76	108314	21178514	1.20
Tc1mariner	48	83789	13289414	0.75
**MITEs**	**234**	**343838**	**87535895**	**4.96**
m2bp	17	24668	6492577	0.37
m3bp	7	7033	1537791	0.09
m4bp	88	127330	31503909	1.78
m5bp	2	1225	414964	0.02
m6bp	3	7523	1738704	0.10
m7bp	3	7746	1198574	0.07
m8bp	22	56732	18130019	1.03
m9bp	2	4802	892191	0.05
mTA	90	106779	25627166	1.45
**Unclassified**	**98**	**20073**	**5849509**	**0.33**
**Total**	**1289**	**1379140**	**294717617**	**16.69**

This table represents a conservative estimation of the repeat content because we focused on manually annotated TEs. Annotation of long TEs is especially difficult given the fragmented nature of the genome assembly. Tandem repeats and satellite sequences are not included. TE copy numbers and base pairs were obtained by running RepeatMasker version 3.2.9 with the *Ixodes scapularis* TE library (available for

download from the TEfam database at: http://tefam.biochem.vt.edu) and VectorBase (https://www.vectorbase.org/).

Supplementary Table 14. Summary of transposable elements identified in the *Ixodes scapularis* coding sequence.

Class	Total Families	Total Sequences	Bases Occupied	Percent Genome
**Class I**				
L1	1,773	1,980	137,648,067	6.55
Ty3_gypsy	1,644	1,867	67,124,477	3.20
Penelope	290	328	18,329,691	0.87
Pao Bel	81	97	3,988,612	0.19
Rnase_H	24	26	164,423	0.01
**Class II**				
piggyBac	80	90	8,723,874	0.42
PIF	40	43	7,637,650	0.36
hAT	102	129	7,538,310	0.36
Mariner	74	91	6,280,194	0.30
P	54	58	6,054,528	0.29
Mutator	19	56	188,089	0.01
Merlin	3	3	160,327	0.01
Unclassified (mostly fragments)	1,338	2,693	40,366,119	1.92
**Total**	5,522	7,461	304,204,361	14.49

A transposable element genomic search was devised by (1) doing Psiblast of the coding regions of representatives of the diverse families of transposable elements against the non-redundant database from NCBI; (2) constructing matrices from the alignments to be used by the tool rpsBLAST; (3) by retrieving genomic matches by rpsBLAST against this database that are larger than 500 nt and *e* value < $1e^{-15}$, with additional 500 nt of flanking regions; (4) finding terminal repeats (direct and inverted) and trimming the sequences accordingly (sequence without repeats are trimmed on their coding sequences); (5) by clusterizing the data set of 7,461 elements that have 90% identity over 90% of its length to obtain 5,522 clusters of elements, then (6) comparing the consensus sequences by BLAST to several databases and (7) finally running a program to classify these elements. The obtainied sequequences were compared to the genome to identify the number of bases occupied by this representative set.

Supplementary Table 15. Summary of fluorescent *in situ* hybridization (FISH) to *Ixodes scapularis* ISE18 cell line chromosome spreads using BAC clone probes. Probes included only fully sequenced and assembled BAC clones from the 10X BAC clone library. D=Dispersed signal; S=Specific signal; I=Inconsistent result.

Genbank Accession	BAC Size(bp)	10X BAC Library Plate/Well	FISH Result
AC192414	117688	5/A1	D
AC192415	122081	33/A1	D
AC192416	107486	36/A1	D
AC192417	102815	40/A1	D
AC192418	114701	41/A1	D
AC192419	113880	43/A1	D
AC192420	146997	45/A1	D
AC192421	137443	49/A1	D
AC192422	135954	51/A1	D
AC192423	109798	53/A1	D
AC192424	144952	55/A1	D
AC192425	145957	60/A1	D
AC192426	117608	64/A1	S
AC192427	98661	66/A1	D
AC192428	106828	67/A1	D
AC192429	136633	68/A1	D
AC192742	133074	48/A1	D
AC192743	130937	54/A1	D
AC192744	115169	61/A1	D
AC200531	77162	2/A1	D
AC205630	95257	1/F14	D
AC205631	108628	1/P2	D
AC205632	26509	3/K2	I
AC205633	97628	3/P2	I
AC205634	112417	6/P2	S
AC205635	104824	11/P2	D
AC205636	106471	12/P2	D
AC205637	131925	14/C7	D
AC205638	100378	15/P2	D
AC205639	92483	16/P2	D
AC205640	109665	22/P2	I
AC205641	179341	24/P2	D
AC205642	110473	27/P2	S
AC205643	128247	31/P2	D
AC205644	110010	37/P2	D
AC205645	120505	41/M8	D
AC205646	122242	42/P2	D
AC205647	115710	43/E15	D
AC205648	126704	44/P2	D
AC205649	111949	47/P2	D
AC205650	107316	56/P2	D

AC205651	172110	58/P2	D
AC205652	50437	62/P2	D
AC205653	108041	63/P2	D
AC205654	14567	69/P2	I

Supplementary Table 16. Summary of protein domains identified in *Ixodes scapularis* sialome sequences.

Group No.[a]	Group Name	Mol. Wt. (kDa)	Proposed Gene No.[b]	*Is*	*Hs*	*Bt*	*Gg*	*Aa*	*Cq*	*Ag*	*Dm*	*Ce*	*At*	Function[c]
3	Kunitz domain-containing peptides	Multiples of 8	30	74	26	28	7	5	5	4	30	57	0	Anti-clotting
13b	Selenoproteins	15	3	2	4	2	1	1	1	1	1	1	1	Presumed antioxidant
13b	Alkyl hydroperoxide reductase	28	3	6	23	8	5	6	6	6	10	4	11	Detoxification
8	Metalloproteases	55	3	34	108	71	38	9	14	10	19	8	0	Fibrinolytic
25b	Dipeptidyl peptidase	60	2	7	7	8	3	9	8	10	7	1	0	Kininase
12b	Defensin	6	2	8	13	29	12	4	1	2	1	0	0	Immunity
17b	Cystatin	14	2	13	30	23	9	2	0	0	4	3	11	antiinflammatory, immunosuppressor
17c	Serpin	24	2	44	115	80	31	32	48	25	44	11	12	Serine protease inhibitor
25a	Serine proteases	Various	2	133	293	154	77	392	448	321	283	12	0	Specificity unknown
17a	TIL domain peptide	11	2	23	16	7	15	23	13	23	11	29	0	Unknown
25c	Phospholipase A2	Truncated	1	2	2	2	2	5	1	1	1	0	0	Specificity unknown
13a	Glutathione peroxidase	25	1	8	15	7	4	3	4	5	4	10	8	Presumed antioxidant
26b	Antigen 5	35	1	12	38	13	11	35	30	19	22	34	22	Unknown
26a	Ixoderin	30	1	27	61	26	34	35	92	49	22	7	0	Immunity
12a	GGY repeat family[d]	4.7-13	Various families											Unknown, possibly antimicrobial
18	Mucins[e]	Various	Various families											Unknown
10	Ixostatin	9-11	25	11										unknown
15	WC-10 family	9-11	21	4										Unknown
11	Lipocalins	18-24	20	40										Kratagonist
16	LPTS family	12-16	11	0										Unknown
4	Proline/Glycine rich peptides	6-8	10	24										Unknown
7	9 and 7 kDa family	7-9	10	12										Unknown
6	5.3 kDa family	5.3	9	6										Antimicrobial
1	Basic tail polypeptides	13-14	8	16										Anti-clotting
9	Ixodegrin (RGD containing peptides)	<4	5	10										Probable platelet aggregation inhibitor

14	Anticlomplement Isac	16	4	1	Anticomplement
19	IS6 family	9-12	4	2	Unknown
2	Basic tailless polypeptides	10-11	3	included in group 1	Unknown
20	12 kDa family	12	3	2	Unknown
5	18.7 kDa family	19	2	4	Unknown
12c	Microplusin	13	2	15	Antimicrobial
21	26 kDa family	26	2	2	Unknown
23	Toxin like, may be related to IS6	8-9	2	1	Unknown
24	SRAEL family	16-22	2	2	Unknown
25d	Small ribonuclease	6	1	1	Unknown
22	30 kDa family	30	1	20	Unknown

[a]Based on[64]. The supplemental table can be obtained from http://exon.niaid.nih.gov/transcriptome/Ix_scapularis_sialome_2005/Sup-tables/Sup-table-2.xls.
[b]Proteins that are >90% divergent in amino acid sequence.
[c]Based on at least one member of a protein family that has been functionally analyzed.
[d]Heterogeneous family,with poor primary sequence conservation, but having GGY repeats.
[e]Heterogeneous family having in common solely over 10 N-acetyl-galactosylation sites.
Aa, Aedes aegypti; Ag, Anopheles gambiae; At, Arabidopsis thaliana; Bt, Bos taurus; Ce, Caenorhabditis elegans; Cq, Culex quinquefasciatus; Dm, Drosophila melanogaster; Gg, Gallus gallus; Hs, Homo sapiens; Is, Ixodes scapularis.

Supplementary Table 17. List of putative immune-related genes identified in the *Ixodes scapularis* genome.

Immune pathway and gene	Gene description	*I. scapularis* supercontig #	Base pair range on supercontig	Genbank accession #
**Toll Pathway**				
Dorsal	Embryonic polarity dorsal	DS612897	344,672-368,433	ISCW000140
Cactus	NF-kappaB inhibitor	DS807313	89,019-110,525	ISCW019520
	IkappaB	DS789268	14,144-34,799	ISCW007030
Pelle	serine-threonine protein kinase	DS633730	60,689-76,958	ISCW001463
Tube	cyclin T-dependent kinase CDK9	DS787602	500,517-518,860	ISCW007160
MyD88	myd88	DS831454	19,812-43,217	ISCW008802
Toll	toll	DS894332	370,864-378,206	ISCW022740
	toll	DS795254	164,145-173,413	ISCW007727
	toll	DS692880	318,809-322,642	ISCW018193
	toll	DS863226	213,685-217,374	ISCW020989
	toll	DS795254	116,582-121,636	ISCW007724
	toll	DS725696	4,704-5,021	ISCW017724*
	toll	DS795254	130,237-135,616	ISCW007726**
	toll	DS695149	145,084-147,147	ISCW004495**
	toll	DS794567	257,906-260,571	ISCW008289**
	toll	DS851201	446,328-461,671	ISCW020221**
Spätzle	spatzle alternatively spliced isoform 11.27	DS924847	58,310-78,232	ISCW022569
	Sptzle 1B	DS915052	406,105-422,910	ISCW022732
**Imd pathway**				
Caudal	homeobox protein cdx	DS839652	4,771-4,947	ISCW008954
Relish	nuclear factor nf-kappa-B P105 subunit	DS737890	107,162-147,186	ISCW018935
IKK gamma	protein kinase	DS711115	74,892-92,974	ISCW003529
IKK beta	inhibitor of nuclear factor kappa-B kinase alpha	DS684865	34,555-75,124	ISCW002130
TAK1	tak1	DS956364	46,654-69,194	ISCW023496
TAB2	conserved hypothetical protein	DS831661	125,422-146,802	ISCW009346
POSH	conserved hypothetical protein	DS980186	71,040-94,158	ISCW015192
Caspar	regulator of the ubiquitin pathway	DS635599	608,153-631,045	ISCW015648

Effete	ubiquitin protein ligase	DS734834	71,859-74,198	ISCW018551
Bendless	ubiquitin protein ligase	DS734517	102,190-115,886	ISCW006743
Uev1a	ubiquitin-conjugating enzyme	DS755574	226,269-228,921	ISCW019147
IAP2	inhibitor of apoptosis protein 1 and 2	DS874571	17,481-47,594	ISCW010694
**RNAi pathway**				
Dicer	dicer-1<br>dicer-1	DS643033<br>DS643033	158,526-187,719<br>191,838-226,069	ISCW000889<br>ISCW000890
Argonaute	translation initiation factor 2C	DS620030<br>DS879840<br>DS903494<br>DS887784<br>DS906490	375,533-385,847<br>1,146-17,131<br>161,089-164,329<br>612,740-615,223<br>52,601-76,044	ISCW015916<br>ISCW011768<br>ISCW022696<br>ISCW021130<br>ISCW013378
FMRP	HyFMR	DS662130	57,130-96,383	ISCW002912
VIG	vasa intronic gene	DS630348	7,046-19,031	ISCW000538
Tudor-SN	4SNc-Tudor domain protein	DS947409	53,618-100,428	ISCW014289
Armitage	Conserved hypothetical protein	DS771975	54,521-70,842	ISCW019555
Aubergine	Cniwi protein<br>Cniwi protein	DS692353<br>DS861388	8,438-10,987<br>229,689-253,854	ISCW004464<br>ISCW011373
Rm62	ATP-dependent RNA helicase	DS668332<br>DS668332<br>DS819551	17-18,383<br>27,837-64,710<br>52,974-68,087	ISCW002701<br>ISCW002703<br>ISCW009472
**JAK/STAT pathway**				
JAK (Hopscotch)	Tyrosine protein kinase	DS636921	613,020-649,592	ISCW016158
STAT	Stat3	DS736534	85,785-110,372	ISCW005692
JAK receptor (Domeless)	Receptor protein tyrosine phosphatase	DS672509	132,429-173,396	ISCW016699
PIAS	Sumo ligase	DS741077	191,455-212,512	ISCW005295
SOCS	SOCS box SH2 domain-containing protein	DS788896	252,388-253,269	ISCW019435

**Other immune-related genes**				
Akirin	Protective antigen D48/subolesin	DS936446	66,643-89,471	ISCW023283
Antimicrobial peptides (AMPs)***	AMP	DS766801	7,539-10,842	ISCW005927
	AMP	DS858447	10,615-12,036	ISCW011162
	scapularisin	DS766801	805-4,569	ISCW005926
	secreted salivary gland peptide	DS766801	37,932-40,936	ISCW005928
	microplusin preprotein	DS700881	37,354-41,025	ISCW004019
	microplusin preprotein	DS683675	11,851-16,401	ISCW002113
Caspases****	caspase	DS689930	4,952-18,616	ISCW003039
	caspase	DS923722	42,757-57,807	ISCW013172
	caspase	DS980848	29,397-36,907	ISCW015329
	caspase	DS896168	55,609-67,445	ISCW022545
Defensins	preprodefensin	DS759251	1,299-2,130	ISCW024381
	preprodefensin	DS664851	77,757-80,159	ISCW016747
	preprodefensin	DS929532	1,258-2,470	ISCW022594
	preprodefensin	DS633368	480-1,253	ISCW024015
	defensin	DS930883	134,844-141,980	ISCW022102
Duox	dual oxidase 1	DS798980	38,754-135,685	ISCW007865
Fibrinogen-related proteins	ixoderin precursor	DS662660	9,473-15,818	ISCW002664
	ixoderin precursor	DS929502	1,407-13,639	ISCW012248
	ixoderin precursor	DS959741	7,679-17,815	ISCW013746
	ixoderin precursor	DS860650	948-6,195	ISCW024686
	ixoderin precursor	DS899572	85,691-93,595	ISCW022063
Lysozymes	lysozyme	DS613145	77,141-77,891	ISCW001646
	lysozyme	DS613145	47,267-56,398	ISCW001645
	lysozyme	DS844216	51,691-65,539	ISCW020680
	C-type lysozyme	DS670557	67,058-72,249	ISCW017129
NADPH oxidase	NADPH oxidase	DS690902	246,606-271,833	ISCW002630
Peptidoglycan Recognition Receptors (PGRPs)	PGRP	DS686855	766-1,809	ISCW024175
	Ammonium transporter	DS861599	1-805	ISCW024689
	PGRP	DS697694	122,963-124,835	ISCW004389
	PGRP	DS904186	380,517-387,075	ISCW022212
Thio-ester containing proteins (TEPs)	TEP	DS837598	3,653-100,934	ISCW020822
	alpha-2 macroglobulin	DS790028	75,581-99,146	ISCW019887
	alpha-2 macroglobulin	DS970697	293,496-343,165	ISCW023777
	alpha-2 macroglobulin	DS716413	7,286-62,557	ISCW003923
		DS687147	35,629-71,042	ISCW003089
	conserved	DS779097	53,866-97,786	ISCW007141

	hypothetical protein alpha-2 macroglobulin	

[*]Sequence only shows the Toll/Interleukin-1 receptor domain (TIR) but no leucine-rich repeats (LRRs).
[**]Sequence only shows LRRs but no TIR domain.
[***]AMPs include all the sequences uncovered as AMPs but that were not annotated as “defensins”.
[****]These sequences represent caspases that share similarity with death related ced-3/Nedd2-like protein (Dredd caspase).

Supplementary Table 18. Genes in the *Ixodes scapularis* genome with similarity to the enzymes involved in the mevalonate/farnesyl PP and JH pathways in insects.

			Top BLAST result	
**Enzyme**	**Scaffold (bp range)**	**VectorBase Accession**	**Organism (GenBank Accession)**	***e*-value Amino Acid Identity**
**Farnesyl-PP pathway**				
Acetoacetyl-CoA thiolase	DS624476 (15968-41821)	ISCW016117	*Dendroctonus ponderosae* (AFI45001)	4e-170 (61%)
HMG-S[a]	DS690902 (31998-46779)	ISCW002615	*Nasonia vitripennis* (XP_003426942)	9e-169 (68%)
HMG-R[b]	DS842351 (4797-71147)	ISCW009466	*Pediculus humanus corporis* (XP_002428525)	0.0 (55%)
Mevalonate kinase	DS735207 (298570-308399)	ISCW018716	*Camponotus floridanus* (EFN64406)	4e-60 (35%)
Phosphomevalonate kinase	DS881578 (239893-241993)	ISCW021370	*Acromyrmex echinatior* (EGI169273)	1e-44 (42%)
Diphosphomevalonate decarboxylase	DS921134 (457048-474172)	ISCW022273	*Apis mellifera* (XP_001121619)	7e-122 (51%)
Isopentenyl diphosphate isomerase	not found	not found	not found	not found
Geranyl diphosphate synthase	not found	not found	not found	not found
Farnesyl diphosphate synthase	DS834911 (53770-59211)	ISCW009264	*Dendroctonus jeffreyi* (AAX78435)	2e-96 (47%)
**JH Pathway**				
Farnesyl diphosphate pyrophosphatase	not found	not found	not found	not found
Farnesol oxidase	DS838300 (597086-612073)	ISCW020246	*Ceratosolen solmsi marchali* (XP_011505480)	1e-99 (60%)
Farnesal dehydrogenase	not found	not found	not found	not found
Methyltransferase	DS624614 (1302-5354)	ISCW000145	*Schistocerca gregaria* (ADV17350)	4e-18 (29%)
JH[c] epoxidase	not found	not found	not found	not found

[a] Hydroxymethylglutaryl-CoA synthase.
[b] Hydroxymethylglutaryl-CoA reductase.

[c] Juvenile hormone.

Supplementary Table 19. Putative *Ixodes scapularis* genes associated with ecdysone synthesis and the ecdysone receptor.

Gene name	VectorBase Accession	Scaffold	Coordinates on Scaffold (bp)
**CYP307A1 (*Spook*)**	ISCW024795	DS931697	106..1604
**CYP307B1 (*SPOT*)**<br>**CYP307A2 (*Spookier*)**	ISCW006980	DS782423	5612..12969
**CYP306A1 (*Phantom*)**	ND		
**CYP302A1 (*Disembodied*)**	ND		
**CYP315A1 (*Shadow*)**	ISCW021866	DS864024	44582..49687
**CYP314A1 (*Shade*)**	ISCW021011	DS857608	115434..126481
	ISCW001527	DS638370	30627..62
**Ecdysone receptor**	ISCW003147	DS667471	104353..111170

Supplementary Table 20. Summary of *Ixodes scapularis* aminolevulinic acid (ALA) synthesis, proto-heme synthesis and heme degradation pathways.

Enzyme	Gene	VectorBase Accession	Scaffold	Transcript Evidence GenBank Accession	Gene Identified in REIS
ALA synthase Aminolevulic acid synthase	*hemA*	-	-	-	+
Glutamyl-tRNA synthase†	*gltX*	ISCW018719	DS735207	Dv syn YP_001857605.1	+
Glutamyl-tRNA reductase	*gtrA/hemA*	-	-	Dv ov YP_002306262.1	-
Glutamate-1-semialdehyde 2,1-aminotransferase	*hemL*	-	-	-	+
Aminolevulic acid dehydratase	*hemB*	-	-	-	+
Porphobilinogen deaminase	*hemC*	-	-	-	+
Uroprophyrinogen-III synthase	*hemD*	-	-	-	+
Uroporphyrinogen decaroxylase	*hemE*	-	-	-	+
Corpproporphyrinogen III oxidase	*hemF*	ISCW010977<br>ISCW006377	DS891848<br>DS752864	-<br>-	+
	hemN	-	-	-	+
Protoporphyrinogen IX oxidase	*hemG*	ISCW023396	DS626813	Is syn NP_001167359	+
	hemY	-	-	-	-
Ferrochelatase	*hemH*	ISCW016187	DS626813	Dv syn ZP_03286863.1	+
Heme oxygenase	*hemO*	-	-	Is syn XP_002711461	-
Biliverdin reductase	-	-	--	-	-
Protoheme IX farnesyl transferase	*cyoE*	ISCW008907	DS846584	Ot syn XP_002411071.1	-

ALA, δ-aminolevulinic acid; REIS, *Rickettsia* endosymbiont of *Ixodes scaplaris*[10]; syn, syngalnglion transcriptome; ov, ovary transcriptome, Dv, *Dermacentor variabilis*; Is, *I. scapularis*; Ot, *Ornithodorus turicata*; †peptide evidence (see supplemental text).

Supplementary Table 21. List of *Ixodes scapularis* hemoglobin digesting genes and gene annotations[1]

Function	Gene name	Vector base accession no.	Scaffold	Scaffold coordinates (bp)	Gene length (bp)	Transcript Length (bp)	Length (AA)	No. Exons
Primary hemoglobin cleavage	Cathepsin D (Aspartic protease)	IscW_ISCW023880	DS949737	152,273-190,227	37954	1179	392	1
		IscW_ISCW013185	DS900056:	10,899-23,895	12,996	963	320	8
		IscW_ISCW003823	DS722875:	17,354-32,869	15,515	1100	345	6
	Cathepsin L (Cysteine protease endopeptidase)	IscW_ISCW024899	DS970886	4,733-5,116	5313	383	127	1
	Cathepsin L (Cysteine protease endopeptidase)	IscW_ISCW000076	DS629804	3,735-4,818	1,083	300	99	2
	Legumain (Aspartic endopeptidase)	IscW_ISCW015983	DS621767	1,225-3,192	14,974	1968	446	1
Secondary hemoglobin cleavage	Cathepsin B (Endopeptidase)	IscW_ISCW005981	DS754946	9,428-18,454	9,026	672	223	5
	Cathepsin L (Cysteine protease endopeptidase)	IscW_ISCW024899	DS970886	4,733-5,116	5313	383	127	1
		IscW_ISCW024213	DS704563	4,637-6,606	9464	255	84	1
Tertiary hemoglobin cleavage	Cathepsin C (Aminodipeptidase)	IscW_ISCW003494	DS694733	169,868-186,056	16,188	1,080	352	7
	Cathepsin B (Endopeptidase)	IscW_ISCW005981	DS754946	9,428-18,454	9,026	672	223	5
Final hemoglobin cleavage	SCP (Serine carboxypeptidase)	IscW_ISCW021184	DS88627	64,978-83,896	18,918	1705	473	4
		IscW_ISCW006427	DS752045	233-3,375	3,142	1125	374	2
		IscW_ISCW010371	DS886430	77,615-100,122	22,507	1431	476	6
		IscW_ISCW007492	DS725233	346-7,634	7,288	1416	471	4
	LAP (Leucine aminopeptidase)	IscW_ISCW0023735	DS967246	117,498-141,675	24,177	1590	529	11

[1]Legend: Hemoglobin is digested intracellularly in specialized lysosome (hemosomes, see Fig. 1D). The digestive pathway comprises four major cleavage processes. 1) Primary digestion of the globin moieties into large fragments by the aspartic proteases Cathepsin D and legumain, supported by the cysteine endopeptidase Cathepsin L; 2) digestion of the resulting large peptide fragments (8 -11 kDa) by the endopeptidases Cathepsin B and Cathepsin L, resulting in intermediate size fragments (~ 5 – 7 kDa); 3) digestion of the intermediate size fragments by Cathepsin C and B resulting in small fragments (~ 3 – 5 kDa); 4) digestion of the small peptide fragments by SCP and LAP, liberating free amino acids and dipeptides. Free heme resulting from hemoglobinase activity is inactivated by forming large hematin-like aggregates that accumulate inside the hemosomes[123].

Supplementary Table 22. Summary of *Ixodes scapularis* hemelipoglyco-carrier protein (CP) and vitellogenin (Vg) gene annotations.

I. scapularis Gene	VectorBase Accession	Scaffold	Scaffold Coordinates (bp)	Length (bp)	Length (AA)	No. Exons
**Hemelipoglyco-carrier Protein Genes**						
CP 1	ISCW021709	DS853155	640,093-674,357	4,934	1,556	25
CP 2†	ISCW014675	DS946795	23,797-86,142	4,554	1,517	24
CP 3†	ISCW021710	DS853155	687,061-748,951	3,990	1,329	22
CP 4†	ISCW012424	DS930868	97,596-131,023	3,978	1,325	20
CP 5†	ISCW012423	DS930868	45,085-91,058	3,336	1,111	19
CP 6†	ISCW021704	DS853155	567,881-571,678	1,440	480	8
CP 7†	ISCW021707	DS853155	617,028-622,418	845	265	4
CP 8†	ISCW021706	DS853155	567,881-571,678	460	153	3
CP 9†	ISCW021705	DS853155	605,937-607,255	425	141	2
CP 10†	ISCW024299	DS725419	389-1,716	354	117	2
**Vitellogenin Protein Genes**						
Vg 1	ISCW013727	DS950603	16,225-44,518	4,935	1,644	26
Vg 2	ISCW021228	DS874548	255,397-286,108	5,811	1,936	22

†Incomplete gene model.

Supplementary Table 23. Putative cytchrome P450 genes in *the Ixodes scapularis* genome.

CYP2 Clan[a]	VB Accession[b]	CYP3 Clan	VB Accession	CYP4 Clan	VB Accession
CYP18C1	ISCW009830	CYP41A2	ISCW022948	CYP4W2	ISCW024589, ISCW024427
CYP307A1	ISCW006980, ISCW024795	CYP41B1	ISCW022672	CYP4W2-de10b11b	ABJB010319121
CYP3001A1	ISCW002226	CYP41C1	ISCW022945		
CYP3001A2	ISCW008379	CYP41C2	ISCW022947	CYP4W3	ISCW003279
CYP3001A3	ISCW003457	CYP41C3	ISCW019880	CYP4W4	ISCW022701, ISCW022702
CYP3001A4	ISCW004522	CYP41C4	EW786235.1, EW899917.1	CYP4W5	ABJB010639789.1
CYP3001A5	ABJB010183741.1	CYP41C5	ISCW019198	CYP4W6	ISCW013762
CYP3001A6	ABJB010948131.1	CYP41C6v1	ISCW024627	CYP4W7	ISCW017084
CYP3001A7	ABJB010161347.1	CYP41C6v2	ISCW011029	CYP4DL1	ISCW022695
CYP3001B1	ISCW016425	CYP41C7	ISCW022987	CYP4DL2	ISCW022697
CYP3001B2	EW827166.1, EW827167.1	CYP41C8	ISCW024413	CYP4DL3P	ABJB010866300.1
CYP3001B3	ISCW006203	CYP41C9	ISCW000510	CYP4DL4	ISCW007225
CYP3001B4	ISCW006204	CYP41C10	ISCW010134, ISCW024611	CYP4DL4-de3b	ABJB011028851
CYP3001B5	ISCW004521	CYP41C11	ISCW013318	CYP4DL5	EW922199.1
CYP3001B6	ISCW002182	CYP41C12	ISCW007389	CYP4DM1	ISCW022693
CYP3001B7	ISCW006219	CYP41C13	ISCW008554	CYP4DN1	ISCW024545
CYP3001B8	ISCW016424	CYP41C14	ISCW002138	CYP4DN2	ISCW022708
CYP3001B9	ABJB010987053.1	CYP41C15	ISCW003215	CYP4DP1	ISCW022706
CYP3001C1	ISCW022449	CYP41D1	ISCW008571	CYP4DP2	ABJB010524713.1
CYP3001C2	ISCW022451	CYP3004A1	ISCW018384	CYP4DQ1	DS865979, DS895862, DS714189
CYP3001D1	ISCW008112	CYP3004A2	ISCW018383	CYP4DR1	ISCW016620
CYP3001D2	ISCW013045	CYP3004A3	EW879600.1	CYP4DS1	ISCW005615
CYP3001D3	EW958870.1	CYP3004A4	EW959618.1	CYP4DS2	ISCW005544
CYP3001D4	ISCW024917	CYP3001B1	ISCW016424	CYP4DS3	ABJB010393473.1
CYP3001D5	ABJB010249968.1	CYP3004C1v1	ISCW022652	CYP4DS4	ISCW002003
CYP3001E1	ISCW007349	CYP3004C1v2	EW883126.1	CYP4DS5	ISCW010787
CYP3001F1	ISCW009779	CYP3004C2	ISCW022649	CYP4DS6	ISCW010786
CYP3001F2	ISCW006867	CYP3004C3P	ISCW020210	CYP4DS7	ISCW010788
CYP3001G1	ISCW006936	CYP3004D1	ISCW001306, ISCW001307	CYP4DS8	ISCW019906
CYP3001G2	ISCW022148			CYP4DS9	ABJB010117553.1
CYP3001H1	ISCW005196	CYP3004D2	ISCW002936	CYP4DS10	ISCW002005
CYP3001H2	ABJB010355812.1	CYP3005A1	ISCW003160	CYP4DT1	ISCW016623
CYP3001J1	ISCW002317	CYP3005A2	DS799461.1	CYP319A3	ISCW022705
CYP3001K1	ISCW003266	CYP3005A3	ISCW004132	CYP319A4	DS764743
CYP3001L1	ISCW023771, ISCW006380	CYP3005A4	ISCW007953	CYP319A5	ISCW022704
CYP3001L2	ISCW012594	CYP3005A5	ISCW007954	CYP319A6v1	EW883950.1
CYP3001L3	ISCW018982	CYP3005A6	ISCW012000	CYP319A6v2	ISCW024808
CYP3001L4	ISCW015950	CYP3005A7	ISCW011997	CYP319A7	ISCW022703
CYP3001M1	ISCW017305	CYP3005A8	ISCW011996		
CYP3001M2	ISCW017964	CYP3005A9	ISCW024674		
CYP3001M3	ISCW017306	CYP3005A10	ISCW020282	**Mito Clan**	

CYP3001M4	DS798591			CYP302A1	ISCW010580
CYP3001N1	ISCW007262	CYP3005A11	ISCW011028	CYP3012A1v1	ISCW001527
CYP3001N2	ISCW002062	CYP3005A12	ISCW024197, ISCW008823	CYP3012A1v2	EW909080.1
CYP3001N3	EW887890.1, EW855831.1	CYP3005A13	ISCW024701	CYP3012A1v3	ABJB010790473.1
CYP3001N4	ABJB010068877.1	CYP3005A14	ISCW001241	CYP3012A1v4	EW834608.1
CYP3001P1	ISCW015055	CYP3005A15v1	ISCW024274	CYP3012A2	ABJB010381557
CYP3001P2	ISCW015054	CYP3005A15v2	ISCW024583	CYP3012A3	EW961943.1
CYP3001P3	ISCW006806	CYP3005A16	ISCW014319	CYP3012A4	ISCW008267
CYP3001Q1	ISCW012310	CYP3005A17	ISCW007367	CYP314A1	ISCW021011
CYP3001Q2	ISCW009178	CYP3005A18	ISCW004905	CYP315A1	ISCW021866, ISCW021867
CYP3001Q3v1	ISCW014782	CYP3005A19	ISCW008019		
CYP3001Q3v2	ABJB010212140.1	CYP3005A20	ISCW001104, ISCW001105	**CYP20 clan**	
CYP3001R1	ISCW016133	CYP3005A21	ISCW001103	CYP20	ISCW015973, ISCW015974
CYP3001R2	ISCW005591	CYP3006A1	ISCW005198		
CYP3001S1	ISCW014295	CYP3006B1	ISCW009640		
CYP3002A1	ABJB010539647.1	CYP3006C1	ISCW012785		
CYP3002A2	ISCW024473	CYP3006D1	ISCW014588		
CYP3003A1	ISCW019785	CYP3006E1	ISCW001476		
CYP3003A2	ISCW019784	CYP3006F1	ISCW001473		
CYP3003A3	ISCW022069	CYP3006G1	ISCW007133		
CYP3003A4	ISCW022070	CYP3006G2	ISCW007134		
CYP3003A5	ISCW022071	CYP3006G3	EW786984.1, EL516481		
CYP3003A5-de1b	ABJB010494977.1	CYP3006G4	ISCW001622		
CYP3003A6	ISCW022073	CYP3006G5	ISCW016204, ISCW016205		
CYP3003A7	ISCW022075				
CYP3003A8P	ISCW022076	CYP3006G6	ISCW000235		
CYP3003A9	ISCW022077	CYP3006G7P	DS949456.1		
		CYP3006H1	ISCW001075		
		CYP3007A1	ISCW017673		
		CYP3007A2	EW793223.1, EW875640.1		
		CYP3007A3	ISCW014407		
		CYP3007A4	ISCW000434		
		CYP3007A5	EW886425.1		
		CYP3008A1v1	DS711134.1		
		CYP3008A1v2	ABJB010083687.1		
		CYP3008A1v3	ABJB010429442.1		
		CYP3008A2	ISCW003335		
		CYP3008A3	ISCW010505		
		CYP3008B1	DS884020		
		CYP3009A1	ISCW016385		
		CYP3009A2	DS641118		
		CYP3009A3	ISCW016388		
		CYP3009A4	DS641118		
		CYP3009A5	ISCW016389		
		CYP3009A6	DS641118		

CYP3009A7	DS641118
CYP3009A8	ISCW016390
CYP3009A9	ISCW016392
CYP3009A9-de11b12b	DS641118
CYP3009A10	ISCW016393
CYP3009A10-de6b	DS641118
CYP3009A11	DS641118
CYP3009A12	ISCW015064
CYP3009A13	ISCW013158
CYP3009A14	ISCW007317
CYP3009B1	ISCW007380
CYP3009B2	ISCW011357
CYP3009B3	ISCW023575
CYP3009C1	ISCW016395
CYP3009D1	ISCW015040
CYP3009D2v1	ISCW015041
CYP3009D2v2	ISCW009986
CYP3009D3	ISCW003934
CYP3009D4	DS641118
CYP3009D5P	DS641118
CYP3009D6	ISCW016397
CYP3009D7	ISCW005328
CYP3009D8	EW899738.1, EW899739.1
CYP3010A1	ISCW001418
CYP3010B1	ISCW010002, ISCW010003
CYP3011A1	ISCW006560
CYP3011A2v1	EW797008.1, EW883539.1
CYP3011A2v2	ISCW012810
CYP3011A3	ISCW009136

[a]The clans are higher level clades of genes. Ticks have five clans (including CYP20). The 2 clan has 68 entries with one possible allele (v2). P or –dexxx on the end of a name indicates a pseudogene. (de- indicates detritus exon adjacent to a parent gene, the numbers 10b11b etc indicate the exons that are present). The 2 clan has 2 pseudogenes, 1 variant and 65 genes. The 3 clan has 5 pseudogenes, 7 variants and 100 genes. The 4 clan has 3 pseudogenes, 1 variant and 33 genes. The mito clan has 3 variants and 7 genes. The 20 clan has only 1 gene. There are a total of 206 P450 genes. Halloween genes are (CYP302A1 [disembodied gene (dib)], CYP307A1 [spook (spo)], CYP314A1 [shade (shd)], CYP315A1 [shadow (sad)]). CYP18A1 in *Drosophila melanogaster* has 26 hydroxylase activity and is essential for metamorphosis[185]. [b]VectorBase accession numbers include ISCW gene model numbers if available, if there is no gene model, contig accessions ABJB01XXXXXXX.1 or scaffold accessions DSXXXXXX or ESTs EWXXXXXX.1 are given.

Supplementary Table 24. Putative carboxylesterase genes Identified in the *Ixodes scapularis* genome

Classification	VectorBase Accession Number	Protein Length[†]	*Ixodes scapularis* Scaffold	Base Pair Range on Scaffold
Carboxylesterase/ AChE-like	ISCW012483	654	DS901690	148,125..169,517
	ISCW007849[a]	651	DS807640	41,806..55,016
	ISCW020835[a, b]	647	DS812474	15,574..21,604
	ISCW020833[a]	640	DS818569	257,240..263,338
	ISCW011400[a]	634	DS859680	251,048..253,591
	ISCW011399[a]	632	DS859680	183,448..185,998
	ISCW012339[b, c]	623	DS907147	57,074..98,844
	ISCW020830[a, b, c]	620	DS818569	213,005..222,326
	ISCW022870[b]	617	DS903315	33,354..35,307
	ISCW001079	592	DS638237	347,645..356,732
	ISCW003637[b]	586	DS727378	78,715..80,741
	ISCW005431[b]	564	DS758735	7,074..13,075
	ISCW022246[b]	558	DS921995	692,289..694,852
	ISCW017638[b]	557	DS717196	297,489..301,318
	ISCW006617[b]	556	DS737125	63,964..67,823
	ISCW020819[a]	555	DS839663	26,717..48,021
	ISCW022251[a]	547	DS921995	896,844..900,843
	ISCW021541[a, c]	542	DS889213	16,369..23,464
	ISCW022244[a]	538	DS921995	665,765..669,060
	ISCW013353	534	DS904610	25,924..28,794
	ISCW020832[a]	524	DS818569	238,942..245,039
	ISCW020825[a, c]	518	DS818569	60,968..62,524
	ISCW003278[c]	517	DS685100	11,465..21,610
	ISCW001132[b]	504	DS639501	286,801..288,315
	ISCW001748[b]	504	DS631740	5,492..7,006
	ISCW010310[b]	500	DS819927	6,796..8,298
	ISCW020821[a, b]	499	DS839663	94,462..97,065
	ISCW009205[b]	499	DS828756	4,652..6,151
	ISCW006206[a, b]	499	DS770580	30,210..38,997
	ISCW006896	494	DS789758	5,982..7,560
	ISCW015340[b]	493	DS976673	38,037..39,518
	ISCW004315[b]	483	DS694420	58,222..59,673
	ISCW022252[a]	481	DS921995	912,311..927,215
	ISCW019926	480	DS798293	1,550,582..1,572,844
	ISCW024484	471	DS773540	719..2,131
	ISCW024669	467	DS867945	499..1,899
	ISCW007848[a]	464	DS807640	28,185..29,576
	ISCW022036	461	DS926387	907,297..921,121
	ISCW024395	460	DS750145	5,506..6,889
	ISCW002384[b]	452	DS663061	396,472..397,830
	ISCW010323	432	DS833783	42,994..59,925
	ISCW006205[a]	425	DS770580	425..4,231
	ISCW014784	421	DS945247	9,272..12,166
	ISCW020826[a]	413	DS818569	102,860..112,799
	ISCW021542[a, c]	388	DS889213	47,182..54,677
	ISCW007846[a]	358	DS807640	11,151..12,224
	ISCW003776	356	DS731177	27,172..46,710
	ISCW022253[a]	354	DS921995	933,635..943,599
	ISCW020829[a]	348	DS818569	133,597..151,342

	ISCW015477	330	DS980614	7,699..11,969
	ISCW001875	325	DS671188	563,450..582,923
	ISCW020818[a]	311	DS839663	18,224..21,291
	ISCW020837[a]	287	DS812474	41,993..42,853
	ISCW009994	281	DS822211	2,541..12,634
	ISCW022255[a]	279	DS921995	1,025,904..1,026,740
	ISCW020827[a]	279	DS818569	123,253..124,089
	ISCW011398[a]	279	DS859680	180,717..181,553
	ISCW001837[a]	272	DS683640	413,937..414,752
	ISCW023613[a]	270	DS963588	441,327..442,136
	ISCW020828[a]	259	DS818569	128,594..129,370
	ISCW022256[a]	250	DS921995	1,059,602..1,060,351
	ISCW021543[a]	250	DS889213	54,792..55,544
	ISCW022245[a]	245	DS921995	682,492..684,860
	ISCW000833	228	DS629490	41,992..45,753
	ISCW015220	227	DS974313	721..11,930
	ISCW009289	227	DS841411	1,826..9,546
	ISCW020834[a]	217	DS818569	273,050..276,739
	ISCW006376	207	DS768980	5,026..13,061
	ISCW022249[a]	202	DS921995	846,363..846,968
	ISCW024894	188	DS932732	484..1,047
	ISCW020831[a]	186	DS818569	228,504..229,808
	ISCW004947	141	DS728330	428..4,745
	ISCW022250[a]	128	DS921995	870,943..875,528
	ISCW014233	118	DS937587	97..453
	ISCW007945[a]	113	DS788282	16,722..17,682
Carboxylesterase	ISCW019824	391	DS796655	26,651..39,339
Juvenile Hormone Esterase	ISCW016978	412	DS654645	97,242..121,428
	ISCW022078	276	DS892946	257,260..279,544
Pyrethroid-Metabolizing Carboxylesterase	ISCW014411	530	DS971257	10,154..12,726
	ISCW014780	503	DS967860	56,802..58,313
	ISCW022961[b]	491	DS939604	711,415..712,890
	ISCW023610[a]	489	DS963588	305,840..327,886
	ISCW023611[a]	431	DS963588	334,284..355,108
	ISCW023612[a]	359	DS963588	380,390..381,538
	ISCW007946[a]	255	DS788282	22,248..23,305
	ISCW024448	246	DS793560	222..962
	ISCW001836[a]	208	DS683640	406,591..413,763
	ISCW022937	183	DS971562	177,536..178,574
	ISCW023615[a]	155	DS963588	451,386..452,909

[†]Gene models ranked in order of descending amino acid length of conceptual protein.
[a]Denotes scaffold containing two or more carboxylesterase gene models.
[b]Denotes potentially complete gene model.
[c]Denotes putative acetylcholinesterase; AChE, acetylcholinesterase.

Supplementary Table 25. Putative neuropeptide genes in *Ixodes scapularis.*

Neuropeptide Genes	Scaffold	Scaffold Coordinates (bp)	VectorBase Accession
Achatin-like (GFGE)	DS940350	23019..23117	NA
AKH/corazonin-related peptide	DS968442	24720..24893	NA
Allatostatin A	DS971562	340315..339812	ISCW022939
Allatostatin B (myoinhibitory peptide)	DS704057	214860..217973	ISCW017595
Allatostatin C	DS617680	26756..26517	ISCW001803
Allatosattin CC	DS614450	94160..93978	ISCW001408
Allatotropin	DS723986	69897..74019	ISCW017791
Vasopressin/Oxytocin-like (inotocin)[a]	DS955335	731..757	NA
	DS655913	50489..50686	NA
Bursicon alpha	DS725348	327154..329168	ISCW004617
Bursicon beta	DS725348	334706..336760	ISCW004618
CAPA (Pyrokinin / periviscerokinin)	DS798279	53447..57119	ISCW019582[c]
CCAP	DS863512	155818..156096	ISCW010619
CCHamide-1[a]	DS920188	4270..1925	ISCW013057
	DS721341	944..1070	
Corazonin	DS968442	4830..8114	ISCW014429
Calcitonin-like diuretic hormone 1	DS849364	213812..229510	ISCW020490
Calcitonin-like diuretic hormone 2	DS833812	290964..308120	ISCW009341
Corticotropin-releasing factor-related	DS951787	1112..1534	ISCW007845
diuretic hormone[b]	DS793410	18053..18115	
Eclosion hormone	DS652454	187087..184932	ISCW001941
EFLamide	DS945230	55463-66354	ISCW014582
Glycoprotein A2[b]	DS850534	41653..41751	NCBI prediction
	DS669550	1333..1046	
	DS957846	12481..12573	
Glycoprotein B5	DS860962	49721..66736	ISCW010926
Insulin like peptide (ILP4)	DS687889	56659..72302	ISCW002549
Ion transport peptide	DS934076	108540..97467	ISCW023228
Kinin	DS680282	583..1410	ISCW024200[1]
Neuroparsin	DS781496	23994..25192	NA
Orcokinin	DS860349	8710..8450	ISCW010518[1]
Proctolin	DS752645	23044..97988	ISCW005701[1]
PTTH-like	DS624571	79215..96811	ISCW001809
RYamide	DS762742	10487...40630	ISCW005825
SIFamide	DS939604	10166..18491	ISCW022950
Short neuropeptide F[a]	DS682464	1..104	ISCW007409
	DS800964	20299..20852	
Sulfakinin	DS674693	50707..50498	NA
Tachykinin[a]	DS805407	18901..16589	ISCW008383
	DS714254	229..80	
Trissin	DS706258	980-1054	
**Novel Putative Neuropeptide Genes[c]**			
FLVamide	DS925401	117227..115863	NA
GTVamide-1[a]	DS641015	1037..1	NA
	DS726073	1037..1	NA
	DS871441	73..5	
GTVamide-2	DS873396	117488..117156	NA
IRLamide	DS963481	1316..567	NA
LHFamide	DS918990	378863..381517	ISCW012656
LHFa/AVFamide[b]	DS918990	305627..305271	ISCW000205
	DS647107	913..1149	
LRFamide	DS810236	227458..231328	ISCW019773[d]
PWGamide	DS680282	715..1383	ISCW024200

QFTa/QFAa/QLTamide	DS810352	3..1431	NA
QFAa/ HFAa/QLTamide[a]	DS799148	921..19	NA
	DS699187	1175..1	NA
QFAa/QVKamide	DS658524	979..2	NA

[a]The gene likely spans multiple scaffolds (and multiple predictions)
[b]Possible allelic forms of two scaffolds.
[c]Predicted based on the repeated short peptides with C-terminal amidation canonical signals (GR or GK). These peptides do not have homology with other known, insect neuropeptides.
[d]Predictions that need to be corrected for the reading frame.
NA=Not found in computationalannotation.

Supplementary Table 26. List of G protein-coupled receptors (GPCRs) identified in *Ixodes scapularis.*

GPCR class	GPCR subclass	GPCR family	*I. scapularis* GPCR	*I. scapularis* scaffold	Coordinates on scaffold (bp)	VectorBase accession
**(1) Class A-Rhod(opsin) receptor family**						
	**Amine receptors**					
		**Dopamine**				
			GPRdop1[†]	DS648196	133,404-134,681	ISCW001496
			GPRdop2[†]	DS812273	247,251-248,624	ISCW008775
			GPRdop3_1	DS715310	294,120-294,563	ISCW005105
			GPRdop3_2	DS748057	13,854-13,963	ISCW006077
			GPRdop3_3	DS978565	1,946-10,664	ISCW015254
			GPRdop3_4	DS834842	11,072-11,233	ISCW008917
		**Muscarinic acetylcholine**				
			mAChR1	DS660344	46,915-48,657	ISCW001961
			mAChR2	DS968008	135,220-137,700	ISCW014424
		**Octopamine/Tyramine**				
			GPRoa1[†]	DS729026	32,100-33,527	ISCW003835
			GPRoa2[†]	DS847958	146,790-147,929	ISCW008552
			GPRtyr1[†]	DS964012	103,184-104461	ISCW013655
			GPRtyr2[†]	DS728699	4690-5811	ISCW005195
		**Serotonin**				
			GPR5ht1	DS756593	240,283-253,629	ISCW019072
			GPR5ht2	DS883764	16,244-17,440	ISCW020906
			GPR5ht3	DS666028	398,996-400,363	ISCW017050
			GPR5ht4	DS756593	157,071-160,431	ISCW019070
	**Peptide receptors**					
		ACP				
			ACP-R1[†]	DS874502	36,974-42,229	ISCW011612+NEW
			ACP-R2[†]	DS635143;<br>DS675617;<br>DS797600	68,533-69,052;<br>21664-21,867;<br>569-1124	ISCW001755+<br>ISCW003272+<br>ISCW008018
			ACP-R3[†]	DS786800	296,857-297,402;<br>441,895-445,805	ISCW019339+<br>ISCW019342
			ACP-R4[†]	DS679693;<br>DS621023	139,339-139,860;<br>55,588-58,021	ISCW017422+<br>ISCW000658
			ACP-R5	DS641985	30,478-31,447	ISCW000195
			ACP-R6	DS908815	64,376-64,909	ISCW013251
		Allatotropin				

	AT-R	DS978161	73,824-74,264	ISCW015323+ ISCW015322
Allatostatin (A)				
	Ast-A-R1	DS627425	21,088-22,374	ISCW001334
	Ast-A-R2	DS616747	171,212-172,466	ISCW016381
	Ast-A-R3	DS616747	216,452-217,633	ISCW016382
	Ast-A-R4	DS946344	139,992-141,146	ISCW014938
Allatostatin (B)				
	Ast-B-R1	DS814451	498,437-516,130	ISCW008779-A
	Ast-B-R2	DS814451	498,437-516,130	ISCW008779-B
Allatostatin (C)				
	Ast-C-R	DS789528	6,027-12,848	ISCW007666
Bursicon				
	Burs-R	DS641526	219,850-250,933	ISCW015788
Capa/CAP_{2b}/Periviscerokinin				
	Capa-R1†	DS640702; DS713265; DS713265; DS674949; DS980147	9,628-9,977; 1,470-1,694; 18-304; 1,541-1,674; 14,584-15,105	ISCW000633+NEW+ ISCW015219
	Capa-R2	DS902408	45-15,095	ISCW012018
	Capa-R3	DS967049	68,951-69,238	ISCW014181
CCAP				
	CCAP-R1	DS642648	32,147-35,927	ISCW000563
	CCAP-R2	DS902282	1,720-11,529	ISCW013454
	CCAP-R3	DS713552	1,900-18,499	ISCW004135
	CCAP-R4	DS881571	21,179-21,656	ISCW011686
CCHamide-1				
	CCHa1-R	DS955040	343,318-344,424	ISCW015075
Corazonin				
	CRZ-R1†	DS862522; DS753205	337,666-338,286; 99,573-100,052	ISCW010571+ ISCW006212
	CRZ-R2	DS743283	73,952-74,512	ISCW005601
GPA2/GPB5				
	LGR1-A	DS776412	12,445-14,686	ISCW007539
	LGR1-B	DS670197	19,910-20,201	ISCW001983
Inotocin				
	IT-R1†	DS658583	333,292-352,021	ISCW016651
	IT-R2†	DS811967	34,737-35,711	ISCW008700

	IT-R3†	DS802003	16,558-17,508	ISCW007179
Kinin				
	Kin-R1†	DS915052	240,714-241,681;<br>53,234-53,339;<br>49,154-49,247;<br>45,556-45,826;	ISCW022730+<br>ISCW022728
	Kin-R2	DS915052	769,801-769,880;<br>803,104-803,316	ISCW022739
	Kin-R3	DS915406	62,791-63,588	ISCW022222
	Kin-R4	DS972284	15,019-15,848	ISCW015326
Myosuppressin				
	MS-R	DS710828	131-1,127	ISCW004636
Proctolin				
	Proct-R	DS711613	287,447-289,213;<br>289,343-291,511	ISCW017865+<br>ISCW017866
Pyrokinin				
	PK-R*	DS929178	1,062,985-1,063,966	ISCW022759+<br>ABJB011125851
RYamide				
	RYa-R1	DS816975	121,888-122,667;<br>34,779-35,090	ISCW020603+<br>ISCW020601
	RYa-R2	DS816975	9,435-9,878	ISCW020600
SIFamide				
	SIFa-R	DS721695	449,641-450,381;<br>563,784-566,715	ISCW017837+<br>ISCW017839
Short Neuropeptide F				
	sNPF-R1	DS646881	88,703-90,025	ISCW000923
	sNPF-R2	DS646881	90,611-91,642	ISCW000924
Sulfakinin				
	SK-R1	DS748459	41,702-49,507	ISCW005570
	SK-R2	DS822900	6,468-9,788	ISCW009627
	SK-R3	DS909250	840,577-842,643	ISCW022781
	SK-R4	DS747369	8,639-37,302	ISCW005948
	SK-R5	DS671072	3,595-6,522	ISCW001892
	SK-R6	DS784565	1,046-55,113	ISCW007293
	SK-R7	DS648932	8,474-11,106	ISCW001201
Tachykinin				
	TK-R1	DS848485	50,750-51,163;<br>102,898-133,431	ISCW010010+<br>ISCW010011
	TK-R2	DS969660	233,259-233,744;	ISCW013545+

				96,620-97,175	ISCW013543
		TK-R3	DS787613	187,848-188,293	ISCW007598
		TK-R4	DS966520	9,416-9,925	ISCW013598
		TK-R5	DS643864	392,493-428,777	ISCW015892
		TK-R6	DS765493	118,152-120,504	ISCW006511
		TK-R7	DS754151	181,199-209,245	ISCW005553
		TK-R8	DS641764	8,595-8,791	ISCW000039
		TK-R9	DS747089	177,811-178,007	ISCW006476
		TK-R10	DS649700	31,210-31,421	ISCW001766
	Trissin				
		Trissin-R1	DS746403	46,424-55,835	ISCW006418
		Trissin-R2	DS812310	49,622-55,240	ISCW009718
**Purine receptors**					
	**Adenosine**				
		GPRads1	DS751891	15-731	ISCW006710
		GPRads2	DS857834	851,959-852,927	ISCW021342
		GPRads3	DS688131	171,862-193,233	ISCW002246
**(Rhod)opsin receptors**					
	**Long**	GPRop1_1	DS655566	780-1051	NEW
		GPRop1_2	DS631721	363-641	NEW
		GPRop1_3	DS955589	190-451	NEW
		GPRop1_4	DS681879	565-876	NEW
	**Unknown**	GPRop2_1	DS727386	14108-17853	ISCW004568
		GPRop2_2	DS647038	58-387	NEW
	**Pteropsin**	GPRop3	DS748823	19,086-19,376	ISCW005498
**Orphan/Putative Class A GPCRs**					
		GPRorp1	DS834336	4,245-8,992	ISCW009595
		GPRorp2	DS928128	52-732	ISCW011905
		GPRorp3	DS885437	65,156-92,934	ISCW021283
		GPRorp4	DS854897	42,286-78,035	ISCW020998
		GPRorp5	DS895157	172,724-173,425	ISCW022377
		GPRorp6	DS810236	58,185-65,642	ISCW019770
		GPRorp7	DS961247	253,289-254,266	ISCW014455
		GPRorp8	DS694733	137,237-138,349	ISCW003493
		GPRorp9	DS758491	336,265-339,216	ISCW018984
		GPRorp10	DS799887	152,872-154,406	ISCW007873
		GPRorp11	DS622494	210,268-238,098	ISCW000432
		GPRorp12	DS794020	20,137-20,862	ISCW007619
		GPRorp13	DS957018	374,567-374,881	ISCW023266
		GPRorp14	DS819573	1,022-9,833	ISCW008691

GPRorp15	DS895157	130,408-130,773	ISCW022376
GPRorp16	DS727732	6,810-7,274	ISCW018171
GPRorp17	DS718929	48,953-50,059	ISCW004650
GPRorp18	DS695281	139,310-140,419	ISCW018273
GPRorp19	DS651746	89,066-90,402	ISCW015953
GPRorp20	DS849590	246,992-248,101	ISCW010126
GPRorp21[†]	DS978744	75,826-92,419	ISCW015218+NEW
GPRorp22	DS664726	6,762-7,730	ISCW002641
GPRorp23	DS909250	807,932-809,260	ISCW022779
GPRorp24	DS951856	59,174-59,443	ISCW013584
GPRorp25	DS933420	12,704-13,765	ISCW014824
GPRorp26	DS626219	6,965-8,056	ISCW000606
GPRorp27	DS822291	20,914-22,134	ISCW010179
GPRorp28	DS847080	82-553	NEW
GPRorp29	DS794029	116-365	NEW
GPRorp30	DS957063	1,197-1,356	NEW
GPRorp31	DS622115	720-804	NEW
GPRorp31	DS734036	1,535,022-1,543,165	ISCW018990
GPRorp32	DS848412	15,099-15,401	ISCW009648
GPRorp33	DS673067	5,821-6,303	ISCW002847
GPRorp34	DS923672	7,604-8,503	ISCW013090
GPRorp35	DS825031	28,068-29,836	ISCW009568
GPRorp36	DS915257	2,800-28,037	ISCW013383
GPRorp37	DS708537	221,842-297,739	ISCW018360
GPRorp38	DS930058	108,838-109,239	ISCW013211
GPRorp39	DS755450	72,170-87,450	ISCW006089
GPRorp40	DS848412	15099-15401	ISCW009648
GPRorp41	DS673067	5,821-6,303	ISCW002847
GPRorp42	DS923672	7,604-8,503	ISCW013090
GPRorp43	DS825031	28,068-29,836	ISCW009568

(2) Class B – Secretin receptor family

Diuretic hormone receptors

Calcitonin-like

CT/DH-R1[*]	DS922272	17,085-30,991	ISCW012970
CT/DH-R2	DS687147	107,053-141,306	ISCW003092
CT/DH-R3	DS769661	26,884-146,275	ISCW018841
CT/DH-R4	DS711942	10,647-69,312	ISCW004902
CT/DH-R5	DS677381	25,745-78,149	ISCW017538

Corticotropin-releasing hormone-like (CRF-like)				
	CRF/DH-R1*	DS783174	89,137-131,136	ISCW007036
	CRF/DH-R2a†‡	DS784114;	14,660-139,631;	ISCW007612;
		DS789666	100,614-100,769	ISCW007615
	CRF/DH-R2b	DS704079	5,933-78,153	ISCW017942
	CRF/DH-R3	DS758074	212,330-212,392	NEW
			214,835-214,983	NEW
			221,884-221,950	ISCW019068
	CRF/DH-R4	DS793456	1,543-38,369	ISCW019312
	CRF/DH-R5	DS810171	300-480	NEW
**Pigment dispersing factor receptor**				
	PDF-R1	DS668046	445,965-498,626	ISCW017309
	PDF-R2	DS668046	721,274-777,173	ISCW017314
**Orphan/ Putative Class B GPCRs**				
	GPRorp1	DS906776	30,788-78,611	ISCW012057
	GPRorp2	DS909780	606,602-613,020	ISCW022534
	GPRorp3	DS650442	28,460-36,150	ISCW016343
	GPRorp4	DS650414	56,145-57,149	ISCW000074
	GPRorp5	DS757053	175,008-192,421	ISCW005937
	GPRorp7	DS929178	814,031-814,471	ISCW022757
	GPRorp8	DS921316	47,452-59,327	ISCW012038
	GPRorp9	DS714433	547,697-550,000	ISCW018246
	GPRorp10	DS968865	1,339,830-1,340,321	ISCW023674
	GPRorp11	DS968865	1,320,857-1,321,348	ISCW023671
	GPRorp12	DS968865	1,338,700-1,339,191	ISCW023673
	GPRorp13	DS646990	289,490-339,295	ISCW000464
	GPRorp14	DS806217	91,135-151,617	ISCW019673
	GPRorp15	DS627544	239,378-248,563	ISCW001355
	GPRorp16	DS929508	189,207-239,610	ISCW012721
	GPRorp17	DS756825	20,407-111,042	ISCW006717
	GPRorp18	DS905169	68,799-70,697	ISCW022854
	GPRorp19	DS885034	25,512-27,359	ISCW010897
	GPRorp20	DS730006	701-1,822	ISCW004659
	GPRorp21	DS674693	369,558-371,486	ISCW016899
	GPRorp22	DS968865	1,272,426-1,274,804	ISCW023670
	GPRorp23	DS958380	104-9,636	ISCW014021
	GPRorp24	DS979492	46,128-46,284	ISCW015339
	GPRorp25	DS788275	144-266	NEW

(3) Class C – Metabotropic glutamate-like receptor family

**Metabotropic glutamate receptors**				
	GPRmgl1	DS827319	1,297-9,829	ISCW010068
	GPRmgl2	DS837710	392,177-408,030	ISCW020530
	GPRmgl3	DS727862	10,596-30,463	ISCW004657
	GPRmgl4	DS687238	5,152-11,333	ISCW016808
	GPRmgl5	DS908406	8,172-49,656	ISCW013154
	GPRmgl6_1	DS614359	383,268-425,389	ISCW016580
	GPRmgl6_2	DS614359	190,697-267,345	ISCW016579
	GPRmgl7	DS814554	1,703-14,641	ISCW009984
	GPRmgl8	DS686939	2,170-2,646	ISCW002379
**GABA(B) receptors**				
	GPRgbb1	DS792523	21,774-51,362	ISCW019833
	GPRgbb2_1	DS856995	432,245-455,920	ISCW021466
	GPRgbb2_2	DS856995	551,946-568,013	ISCW021468
	GPRgbb3	DS963588	123,993-201,680	ISCW023607
	GPRgbb4_1	DS842011	316,154-318,677	ISCW020868
	GPRgbb4_2	DS842011	205,862-222,694	ISCW020865
	GPRgbb4_3	DS842011	165,731-169,913	ISCW020864
	GPRgbb4_4	DS842011	92,375-140,091	ISCW020863
	GPRgbb4_5	DS842011	33,574-49,385	ISCW020862
**Orphan/Putative Class C GPCRs**				
	GPRorp1	DS959025	442,721-466,585	ISCW023311
	GPRorp2	DS877997	216-1,225	ISCW011406
	GPRorp3	DS696092	86,002-87,104	ISCW017670
	GPRorp4	DS963588	471,884-472,447	ISCW023616

**(4) Class D- Atypical 7TM proteins**				
**Frizzled**				
	GPRfz1	DS671553	112,320-113,984	ISCW003177
	GPRfz2	DS624476	452,659-454,854	ISCW016122
	GPRfz3	DS976137	33,694-34,746	ISCW015217
	GPRfz4	DS702749	85,729-87,155	ISCW003981
	GPRfz5	DS703155	48,275-50,017	ISCW004077
	GPRfz6	DS708614	201,139-213,311	ISCW004862
	GPRfz7	DS877147	771-1175	NEW
**Smoothened**				
	GPRsmo	DS857340	534,668-554,150	ISCW021763
**Starry night**				
	GPRstn	DS931589	4,009-100,015	ISCW022151

The *I. scapularis* G protein-coupled receptors (GPCRs) are categorized according to their predicted class, subclass, and family. The scaffold number, annotation coordinates and the GenBank accession number (ISCW identifier) corresponding to each GPCR are provided. Abbreviations for GPCR nomenclature: ACP, AKH/corazonin-related peptide; adr, adrenergic; ads, adenosine; Ast, allatostatin; AT, Allatropin; Burs, bursicon; CT, calcitonin; Capa, Capa peptide; CCHa1, CCHamide-1; CCAP, cardioacceleratory peptide; cir, cirl/latrophilin; CRF, Corticotropin-releasing factor-like; CRZ, corazonin; dop, dopamine; fz, frizzled; gbb, gamma amino butyric acid B receptor ($GABA_B$); GPA2, Glycoprotein hormone-alpha-2; GPB5, glycoprotein hormone-beta-5; 5HT, 5-hydroxytryptamine/serotonin; IT, insect oxytocin/vasopressin-like peptide; LGR, leucine-rich repeat-containing GPCR; mACh, muscarinic acetycholine; mgl, metabotropic glutamate; mth, methuselah; MS, myosuppressin; sNPF, short neuropeptide F; npr, neuropeptide receptor; oa, octopamine; op, opsin; orp, orphan; pct, proctolin; pdf, pigment-dispersing factor; pth, parathyroid hormone; pyn, pyrokinin; rxn, relaxin/insulin-like; RYa, RYamide; SK, sulfakinin; SIFa, SIFamide; smo, smoothened; stn, stan/starry night; TK, tachykinin; tyr, tyramine. The gene models corresponding to Dop3_1-4 (D_2-like dopamine receptor), GPRmgl6_1-2, GPRgbb2_1-2, and GPRgbb4_1-5 are believed to represent fragments of single genes split among different contigs. Similarly, Op1_1-4 are fragments of a single gene and confirmed by RT-PCR, and Op2_1 and Op2_2 represent overlapping portions of the same gene but assigned to different contigs, possibly due to an assembly error.

Footnotes:

† Entire cDNA cloned.

‡ N-terminus of CRF/DH-R2a includes gene model ISCW007615.

* Partial cDNA clone

NEW: not automatically annotated, but newly identified region.

Supplementary Table 27. Summary of neuropeptides and neuropeptide GPCRs in *Ixodes scapularis*.

<table>
<tr><th>Neuropeptide</th><th>Neuropeptide Gene ID</th><th>Neuropeptide GPCR</th><th colspan="7">Neuropeptide GPCR Gene ID and Transmembrane (TM) Domains</th></tr>
<tr><th></th><th></th><th></th><th>TM1</th><th>TM2</th><th>TM3</th><th>TM4</th><th>TM5</th><th>TM6</th><th>TM7</th></tr>
<tr><td>ACP</td><td>DS968442</td><td>ACP-R1†</td><td colspan="5">ISCW011612</td><td colspan="2">NEW</td></tr>
<tr><td></td><td></td><td>ACP-R2†</td><td colspan="4">ISCW001755</td><td>ISCW003272</td><td colspan="2">ISCW008018</td></tr>
<tr><td></td><td></td><td>ACP-R3†</td><td colspan="4">ISCW019339</td><td colspan="3">ISCW019342</td></tr>
<tr><td></td><td></td><td>ACP-R4†</td><td colspan="4">ISCW017422</td><td colspan="3">ISCW000658</td></tr>
<tr><td></td><td></td><td>ACP-R5</td><td></td><td></td><td></td><td></td><td>ISCW000195</td><td></td><td></td></tr>
<tr><td></td><td></td><td>ACP-R6</td><td></td><td></td><td></td><td></td><td></td><td colspan="2">ISCW013251</td></tr>
<tr><td>Allatotropin</td><td>ISCW017791</td><td>AT-R</td><td colspan="2">ISCW015323</td><td colspan="5">ISCW015322</td></tr>
<tr><td>Ast-A</td><td>ISCW022939</td><td>Ast-A-R1</td><td colspan="7">ISCW001334</td></tr>
<tr><td></td><td></td><td>Ast-A-R2</td><td colspan="7">ISCW016381</td></tr>
<tr><td></td><td></td><td>Ast-A-R3</td><td colspan="7">ISCW016382</td></tr>
<tr><td></td><td></td><td>Ast-A-R4</td><td>ISCW014938</td><td></td><td></td><td></td><td></td><td></td><td></td></tr>
</table>

Ast-B	ISCW017595	Ast-B-R1*	ISCW008779-A
		Ast-B-R2*	ISCW008779-B
Ast-C§	ISCW001803	Ast-C-R	ISCW007666
Ast-CC§	ISCW001408		
Bursicon a§	ISCW004617	Burs-R	ISCW015788
Bursicon b§	ISCW004618		
Capa		Capa-R1†	ISCW000633 / NEW / ISCW015219
		Capa-R2	ISCW012018
		Capa-R3	ISCW014181
CCAP	ISCW010619	CCAP-R1	ISCW000563
CCHamide-1	ISCW013057	CCHa1-R	ISCW015075
Corazonin	ISCW014429	CRZ-R1†	ISCW010571 / ISCW006212
		CRZ-R2	ISCW005601

CRF/DH	ISCW007845	CRF/DH-R1	ISCW007036
		CRF/DH-R2a[†‡]	ISCW007612
		CRF/DH-R2b	ISCW017942
		CRF/DH-R3	NEW; ISCW019068
		CRF/DH-R4	ISCW019312
		CRF/DH-R5	NEW
CT/DH	ISCW020490	CT/DH-R1	ISCW012970
	ISCW009341	CT/DH-R2	ISCW003092
		CT/DH-R3	ISCW018841
		CT/DH-R4	ISCW004902
		CT/DH-R5	ISCW017538
GPA2[§]	DS669550	LGR1-A	ISCW007539
GPB5[§]	ISCW010926	LGR1-B	ISCW001983
Inotocin	DS955355	IT-R1[†]	ISCW016651

		IT-R2†	ISCW008700		
		IT-R3†	ISCW007179		
Kinin	ISCW024200	Kin-R1†	ISCW022730		ISCW022728
		Kin-R2	ISCW022739		
		Kin-R3	ISCW022222		
		Kin-R4	ISCW015326		
Myosuppressin		MS-R	ISCW004636		
PDF		PDF-R1	ISCW017309		
		PDF-R2		ISCW017314	
Proctolin	ISCW005701	Proct-R	ISCW017865	ISCW017866	
Pyrokinin	ISCW019582	PK-R	ISCW022759		NEW
RYamide	ISCW005825	RYa-R1	ISCW020603		ISCW020601
		RYa-R2			ISCW020600

SIFamide	ISCW022950	SIFa-R	ISCW017837, ISCW017839
sNPF	ISCW007409	sNPF-R1	ISCW000923
		sNPF-R2	ISCW000924
Sulfakinin	DS674693	SK-R1	ISCW005570
		SK-R2	ISCW009627
		SK-R3	ISCW022781
		SK-R4	ISCW005948
		SK-R5	ISCW001892
		SK-R6	ISCW007293
		SK-R7	ISCW001201
Tachykinin	ISCW008383	TK-R1	ISCW010010, ISCW010011
		TK-R2	ISCW013545, ISCW013543
		TK-R3	ISCW007598
		TK-R4	ISCW013598

		TK-R5	ISCW015892
		TK-R6	ISCW006511
		TK-R7	ISCW005553
		TK-R8	ISCW000039
		TK-R9	ISCW006476
		TK-R10	ISCW001766
Trissin	DS706258	Trissin-R1	ISCW006418
		Trissin-R2	ISCW009718

† Entire cDNA cloned.
‡ N-terminus of CRF/DH-R2a includes gene model ISCW007615.
* Original annotation contained two fused genes which have now been corrected (A+B).
§ These ligands use the same receptor.
NEW: not automatically annotated, but newly identified region.

Supplementary Table 28. Selection of neuropeptide and G protein-coupled receptor (GPCR) genes that have been expanded in *Ixodes scapularis* compared to other sequences in arthropods.

	A. No. Neuropeptide Genes		B. No. Peptide Copies in the Propeptide			C. No. GPCR Genes	
**Neuropeptide**	***I. scapularis***	**Other Arthropods**	***I. scapularis***	**Other Arthropods**	**GPCRs**	***I. scapularis***	**Other Arthropods**
**ACP**	1	1	1	1	ACP-Rs	6	1
**Ast-A**	1	1	4	1-35	Ast-A-Rs	4	1-2
**Ast-B**	1	1	3	1-6	Ast-B-Rs	2	1
**Capa**	-	1	-	2-3	Capa-Rs	3	1
**Corazonin**	1	1	1	1	CRZ-Rs	2	1
**CRF/DH**	1	1	1	1	CRF/DH-Rs	5	1-2
**CT/DH**	2	1	1	1	CT/DH-Rs	5	1-2
**Inotocin**	1	1	1	1	IT-Rs	3	1
**Kinin**	1	1	19	1-8	Kin-Rs	4	1
**PDF**	1	1	1	1	PDF-Rs	3	1
**sNPF**	1	1	1	1-5	sNPF-Rs	2	1
**Sulfakinin**	1	1	2	2	SK-Rs	7	1-2
**Tachykinin**	1	1	4	4-11	TK-Rs	10	1-2
**Trissin**	1	1	1	1	Trissin-Rs	2	1

Genes expanded in *I. scapularis* relative to other sequenced arthropods are shaded in gray. The number of neuropeptide genes is not expanded in *I. scapularis* in comparison to other arthropods (Section A). The number of neuropeptides in the *I. scaplaris* kinin propeptide is expanded compared to other arthropods (Section B). Twelve neuropeptide GPCRs are expanded in number in *I. scapularis* in comparison to other arthropods (Section C).

Supplementary Table 29. Details of the *Ixodes scapularus* gustatory receptor (IsGr) family genes and proteins. Columns are: Gene – the gene and protein name assigned (suffixes are PSE – pseudogene, NTE – N-terminus missing, CTE – C-terminus missing, INT – internal exon missing, FIX – assembly was repaired, JOI – gene model spans scaffolds; multiple suffixes are abbreviated to single letters); OGS – the official gene number in the 20,486 proteins in OGSv1 (prefix is ISCW); Supercontig – the v1 genome assembly supercontig ID (prefix DS); Coordinates – the nucleotide range from the first position of the start codon to the last position of the stop codon in the scaffold; Strand – + is forward and - is reverse; Introns – number of introns in coding region; AAs – number of encoded amino acids in the protein; Comments – comments on the OGS gene model, repairs to the genome assembly, and pseudogene status (numbers in parentheses are the number of obvious pseudogenizing mutations).

Gene	OGS	Scaffold	Coordinates	Strand	Introns	AAs	Comments
Gr1FIX	018232	700356	130622-133945	+	3	403	Fix assembly gap
Gr2FIX	011821	855368	2651->5255	+	3	399	Fix assembly gap
Gr3FIX	-	855368	<16600-25223	+	3	389	Fix assembly gap
Gr4CTE	-	855368	29556->30788	+	3	363	Last exon missing
Gr5INT	-	855368	50700-60493	-	3	365	Third exon missing
Gr6CTE	011822	855368	78232->80092	+	3	367	Last exon missing
Gr7INT	-	855368	88901-90482	+	3	369	Third exon missing
Gr8PSE	-	855368	102468-103531	+	3	327	Pseudogene (10)
Gr9	800162	855368	122988-129732	+	3	404	New gene model
Gr10	800163	855368	160441-163747	+	3	394	New gene model
Gr11FIX	011826	855368	172171-176518	+	3	409	Fix assembly
Gr12FIX	-	681555	<144303-147585	-	3	408	Fix assembly gap
Gr13FIX	-	681555	122727-130437	-	3	394	Fix assembly
Gr14	800164	681555	110946-114435	-	3	406	New gene model
Gr15	800165	681555	97115-99209	-	3	409	New gene model
Gr16FIX	-	681555	85287-92568	-	3	413	Fix assembly gap
Gr17CTE	-	681555	<68420-69367	-	1	316	Last three exons missing
Gr18	800161	740051	6893-11542	+	3	394	New gene model
Gr19FJ	-	686724	82->1133	+	3	401	Join across two scaffolds
	-	797949	190->4652	-			Fix gap between scaffolds
Gr20JI	-	615125	<1-677	-	3	305	Join across two scaffolds
	-	686967	<1-12718	+			Part of exon one missing
Gr21FIX	-	848686	<7763-10335	+	3	401	Fix assembly gap
Gr22PSE	-	832595	17507-19366	+	3	393	Pseudogene (1)
Gr23PSE	-	832595	32755-34887	+	3	393	Pseudogene (5)
Gr24	800167	938791	41333-43178	+	3	393	New gene model
Gr25	800168	938791	80049-81932	+	3	393	New gene model
Gr26	800166	853698	97706-99752	-	3	393	New gene model
Gr27	800169	853698	89684-92137	-	3	393	New gene model
Gr28	800170	853698	111225-112868	-	3	393	New gene model
Gr29FIX	-	743784	<1-539	-	3	393	Fix assembly gap
Gr30FIX	-	857335	219->1239	-	3	393	Fix assembly gap
Gr31FIX	-	960372	<1-1000	+	3	393	Fix assembly gap
Gr32FIX	-	682467	236->1431	+	3	388	Fix assembly gap
Gr33	-	873761	45170-47981	+	2	387	Lost first intron
Gr34	800171	873761	51055-55819	-	3	380	New gene model
Gr35	800184	849364	15863-44702	+	3	379	New gene model
Gr36INT	000403	626740	7238-16841	+	3	374	Second exon missing
Gr37	018421	701157	1484-10355	+	6	372	Extra introns
Gr38CTE	013468	908024	<17508-18416	-	-	303	Last three exons missing
Gr39CTE	014940	953252	1139->2071	+	1	311	Last three exons missing

Gr40	800173	895205	28720-34708	+	3	397	New gene model
Gr41JI	-	976502	<1-1135	-	3	373	Join across two scaffolds
		671510	176901->189177	-			Second exon missing
Gr42CTE	008702	827442	6174->7127	+	1	318	Last three exons missing
Gr43CTE	008702	827442	8389->9369	+	1	327	Last three exons missing
Gr44CTE	008702	827442	11273->12205	+	1	311	Last three exons missing
Gr45CTE	-	660244	1213->1959	+	1	249	Last three exons missing
Gr46FC	-	828673	<1->519	+	-	200	Fix assembly gap
							Last three exons missing
Gr47	800174	884190	15927-17192	-	0	421	New gene model
Gr48	800175	663085	56946-58130	-	0	394	New gene model
Gr49	800176	663085	62527-63657	+	0	376	New gene model
Gr50	800177	966800	5785-7014	+	0	409	New gene model
Gr51	800178	637704	96604-97833	-	0	409	New gene model
Gr52	800179	931786	315480-316709	-	0	409	New gene model
Gr53	800180	931786	328322-329596	-	0	424	New gene model
Gr54	800181	940066	661113-662393	+	0	426	New gene model
Gr55	800182	688611	441077-442354	-	0	425	New gene model
Gr56	800183	775741	1373-2533	+	0	386	New gene model
Gr57PSE	-	907737	45413-46569	+	0	386	Pseudogene (8)
Gr58PSE	-	894387	688->1690	-	0	325	Pseudogene (9)
Gr59NC	012263	921071	<111821->112591	+	0	257	Both ends missing
Gr60NC	-	663085	<72725->73345	-	0	200	Both ends missing
Gr61	018260	714433	1067742-1076817	+	3	433	Fine as is
Gr62CTE	011649	878513	<4955-17506	-	1	307	C-terminus missing

Supplementary Table 30. ***Ixodes scapularis*** **ionotropic glutamate receptors and ionotropic receptors**

Gene Name	VectorBase Accession	Scaffold	Start	Stop	Length (bp)	Introns	Comments[1]	Notes
IscaAMPAR01	ISCW016542-PA	DS647564	326210	285753	725	11	-	No ATG
IscaAMPAR02	novel	DS852167	93345	41206	719	11	PSE	1 frameshift
IscaAMPAR03	ISCW017534-PA	DS674345	860787	894098	914	14	-	
IscaAMPAR04	ISCW017535-PA	DS674345	896771	928186	878	12	-	No ATG
IscaIR25a	ISCW008225-PA	DS776301	656	15191	627	5	PSE, CTE	4 frameshifts. No ATG. Added N-term and C-term
IscaIR270.1	ISCW014237-PA	DS941993	1246	48375	526	6	INT	
IscaIR270.2	novel	DS944343	445174	621659	770	8	INT	
IscaIR271	ISCW000549-PA	DS643501	48779	30656	487	9	-	
IscaIR272	ISCW022000-PA	DS917723	300652	307852	283	3	NTE	No ATG. Added C-term, removed residues at N-term
IscaIR273	ISCW006307-PA	DS743451	18121	16238	628	0	-	
IscaIR274	ISCW015704-PA	DS613622	176323	163863	398	5	NTE	
IscaIR275	ISCW020630-PA	DS844967	256726	273694	429	5	NTE	No ATG. Removed residues at N-term
IscaIR276	ISCW022877-PA	DS911299	93493	103589	454	6	-	
IscaIR277	ISCW001635-PA	DS618314	1505	19198	448	7	-	
IscaIR278	ISCW015703-PA	DS613622	146719	160224	423	7	-	
IscaIR279	ISCW019302-PA	DS809877	693067	708183	376	4	NTE	
IscaIR280	ISCW015107-PA	DS979414	264628	258457	354	4	NTE	No ATG
IscaIR281	ISCW010196-PA	DS829427	63163	77406	347	5	NTE	
IscaIR93a	ISCW007957-PA	DS778079	2881	36116	659	9	-	
IscaKA01	ISCW023268-PA	DS954809	79910	4181	736	13	CTE	No ATG
IscaKA02	novel	DS683624	147641	132726	381	3	PSE, NTE, CTE	1 internal stop codon. No ATG
IscaKA03	ISCW001842-PA	DS664102	467895	401477	761	10	PSE	1 frameshift. No ATG. Few edits
IscaKA04	ISCW023274-PA	DS954809	715472	675153	808	12	-	No ATG
IscaKA05	ISCW008266-PA	DS811111	136492	107642	870	12	-	
IscaKA06	ISCW012402-PA	DS907449	324300	367939	728	11	CTE	No ATG
IscaKA07	ISCW008263-PA	DS811111	21039	8427	264	3	NTE	No ATG

IscaNMDAR01	ISCW010976-PA	DS891848	1499	36711	582	9	-	Few edits. No ATG. Short?
IscaNMDAR02	ISCW005598-PA	DS758678	464306	409871	854	13	-	Few edits. No ATG
IscaNMDAR03	ISCW009282-PA	DS834911	299979	287443	1114	9	-	

[1]PSE = pseudogene; NTE = N-terminal end missing; CTE = C-terminal end missing; INT = internal gap.

Supplementary Table 31. Putative Cys-loop and ionotropic glutamate ligand-gated ion channels in the *Ixodes scapularis* genome.

Ion Channel	Acaricidal Compound	Subunits *Ixodes*	Subunits *Drosophila*
**Cys-loop ligand-gated ion channels**			
Nicotinic acetylcholine receptors	Spinosyn	12	10
GABA receptors	Fipronil	4	3
Glutamate-gated anion channels	Ivermectin	6	1
Histamine-gated anion channels	Ivermectin	1	2
pH-sensitive anion channels	Ivermectin	1	1
Other subunits		8	5
**Ionotropic glutamate receptors**			
AMPA		4	2
Kainate		7	10
NMDA		3	2
IRs		15†	66

†30 additional short sequence fragments encoding potential IRs were also identified.

Supplementary Table 32. Proteins identified by LC-MS/MS of ISE6-*Anaplasma* infected *Ixodes scapularis* ISE6 cells.

EARLY INFECTION		LATE INFECTION	
**Over-expressed in infected cells**	**N=13**	**Under-expressed in infected cells**	**N=50**
Cell growth	7.7%*,**	Cell growth	20.0%*,**
Protein metabolism	38.5%	Protein metabolism	30.0%
Nucleic acid metabolism	23.1%	Nucleic acid metabolism	14.0%
Transport	15.4%	Transport	6.0%
		Energy metabolism	16.0%
		Cell communication	6.0%
		Lipid metabolism	0.0%
Unknown	15.3%	Unknown	8.0%
**Up regulated in infected cells**	**N=8**	**Up regulated in infected cells**	**N=31**
Cell growth	12.5%*,**	Cell growth	3.2%*,**
Protein metabolism	37.5%	Protein metabolism	38.7%
Nucleic acid metabolism	25.0%	Nucleic acid metabolism	25.8%
Transport	0.0%*	Transport	0.0%*
		Energy metabolism	9.7%
		Cell communication	3.2%
		Lipid metabolism	3.2%
Unknown	25.0%	Unknown	16.2%

Biological process protein ontology of differentially represented proteins between infected and uninfected tick cells during early and late infections (* and ** indicate significant differences ($p<0.05$) between under- and over-representedproteins in both early and late infections and between early and late infections, respectively).

Supplementary Table 33. Protein differential representation between *Anaplasma phagocytophilum*-early infected and control uninfected *Ixodes scapularis* ISE6 cells.

FASTA Protein Description	UNIPROT	Protein Name	Fold Change[a]	FDR[b]	Biological Process[c]
**Under-expressed in infected cells, N=13**					
tr|B7P2Q4|B7P2Q4_IXOSC Lamin, putative OS=Ixodes scapularis GN=IscW_ISCW000339	B7P2Q4|	Laminin	-2.53	0.000	Cell growth and/or maintenance
tr|B7P7F7|B7P7F7_IXOSC Heat shock protein, putative OS=Ixodes scapularis GN=Isc	B7P7F7|	HSP	-2.31	0.004	Protein metabolism
tr|B7P9E4|B7P9E4_IXOSC Na+/K+ ATPase, alpha subunit, putative (Fragment) OS=Ixo	B7P9E4|	Na+/K+ ATPase, alpha subunit	-2.14	0.000	Transport
tr|B7P5B3|B7P5B3_IXOSC U5 snRNP-specific protein, putative (Fragment) OS=Ixode	B7P5B3|	U5 snRNP-specific protein	-2.04	0.004	Protein metabolism
tr|B7PMC3|B7PMC3_IXOSC Putative uncharacterized protein OS=Ixodes scapularis GN	B7PMC3	Unknown	-2.03	0.000	Unknown
tr|B7P2T4|B7P2T4_IXOSC Ribosomal protein S17, putative OS=Ixodes scapularis GN	B7P2T4|	ribosomal protein S17	-1.95	0.004	Protein metabolism
tr|B7PV22|B7PV22_IXOSC Poly [ADP-ribose] polymerase, putative OS=Ixodes scapul	B7PV22|	poly [ADP-ribose] polymerase	-1.94	0.013	Unknown
tr|B7QDV1|B7QDV1_IXOSC Histone, putative OS=Ixodes scapularis GN=IscW_ISCW01223	B7QDV1	histone	-1.93	0.000	Nucleic acid metabolism
tr|B7P595|B7P595_IXOSC Proline and glutamine-rich splicing factor (SFPQ), puta	B7P595|	proline and glutamine-rich splicing factor (SFPQ)	-1.89	0.003	Nucleic acid metabolism
tr|B7PR83|B7PR83_IXOSC Ubiquitin conjugating enzyme E1, putative OS=Ixodes scap	B7PR83|	ubiquitin conjugating enzyme E1	-1.82	0.000	Protein metabolism
tr|B7P0P1|B7P0P1_IXOSC DNA topoisomerase 2 OS=Ixodes scapularis GN=IscW_ISCW01	B7P0P1|	DNA topoisomerase II	-1.65	0.002	Nucleic acid metabolism
tr|B7QMV1|B7QMV1_IXOSC Elongation factor, putative OS=Ixodes scapularis GN=IscW	B7QMV1	elongation factor 2 (eEF2)	-1.50	0.000	Protein metabolism
tr|B7P5X8|B7P5X8_IXOSC Voltage dependent anion selective channel, putative OS=	B7P5X8|	voltage-dependent anion-selective channel (mt)	-1.33	0.028	Transport
**Over-expressed in Infected cells, N=8**					
tr|A6N9P0|A6N9P0_ORNPR 40S ribosomal protein S14 OS=Ornithodoros parkeri PE=2 S	A6N9P0|	ribosomal protein S14	+ 1.69	0.000	Protein metabolism
tr|B7Q0Q1|B7Q0Q1_IXOSC Putative uncharacterized protein (Fragment) OS=Ixodes sc	B7Q0Q1	Unknown	+ 1.78	0.002	Unknown
tr|B7PZ14|B7PZ14_IXOSC RNA binding protein, putative OS=Ixodes scapularis GN=I	B7PZ14|	RNA-binding protein	+ 1.83	0.011	Nucleic acid metabolism

tr|B7P3Q5|B7P3Q5_IXOSC Vasa intronic protein, putative OS=Ixodes scapularis GN	B7P3Q5|	vasa intronic protein	+ 2.01	0.000	Nucleic acid metabolism
tr|B7QD48|B7QD48_IXOSC Putative uncharacterized protein (Fragment) OS=Ixodes sc	B7QD48|	Unknown	+ 2.35	0.037	Unknown
tr|B7PSQ6|B7PSQ6_IXOSC 40S ribosomal protein S3A, putative OS=Ixodes scapularis	B7PSQ6	40S ribosomal protein S3A	+ 3.10	0.004	Protein metabolism
tr|B2YGD3|B2YGD3_9ARAC Actin (Fragment) OS=Galianora bryicola PE=4 SV=1	B2YGD3	actin	+ 3.27	0.027	Cell growth and/or maintenance
tr|B7PXR5|B7PXR5_IXOSC Chaperonin complex component, TCP-1 eta subunit, putativ	B7PXR5|	chaperonin complex component, TCP-1b eta subunit	+ 11.92	0.004	Protein metabolism

[a] + indicates a significant increase in protein levels and - indicates a significant decrease in protein levels in infected cells ($p < 0.05$).
[b]False discovery rate (FDR) associated to protein identification.
[c]Protein ontology for biological process determined using human protein databases at:
http://www.hprd.org / and http://www.ebi.ac.uk/interpro/

Supplementary Table 34. Protein differential representation between *Anaplasma phagocytophilum*-late infected and control uninfected *Ixodes scapularis* ISE6 cells.

FASTA Protein Description	UNIPROT	Protein Name	Fold Change[a]	FDR[b]	Biological Process[c]
**Under-expressed in infected cells, N=50**					
tr|B7P7F7|B7P7F7_IXOSC Heat shock protein, putative OS=Ixodes scapularis GN=Isc	B7P7F7|	HSP	-5.81	0.004	Protein metabolism
tr|B7P2Q4|B7P2Q4_IXOSC Lamin, putative OS=Ixodes scapularis GN=IscW_ISCW000339	B7P2Q4	Laminin B	-5.64	0.000	Cell growth and/or maintenance
tr|B7P595|B7P595_IXOSC Proline and glutamine-rich splicing factor (SFPQ), puta	B7P595|	proline and glutamine-rich splicing factor (SFPQ)	-2.73	0.003	Nucleic acid metabolism
tr|B7P0P1|B7P0P1_IXOSC DNA topoisomerase 2 OS=Ixodes scapularis GN=IscW_ISCW01	B7P0P1|	DNA topoisomerase II	-2.66	0.002	Nucleic acid metabolism
tr|B7P9E4|B7P9E4_IXOSC Na+/K+ ATPase, alpha subunit, putative (Fragment) OS=Ixo	B7P9E4|	Na+/K+ ATPase, alpha subunit	-2.38	0.000	Transport
tr|B7P3D3|B7P3D3_IXOSC FKBP-type peptidyl-prolyl cis-trans isomerase, putative	B7P3D3|	FKBP-type peptidyl-prolyl cis-trans isomerase	-2.27	0.037	Protein metabolism
tr|B7P1C8|B7P1C8_IXOSC Protein hu-li tai shao, putative OS=Ixodes scapularis GN	B7P1C8|	protein hu-li tai shao, Adducin	-2.17	0.000	Cell growth and/or maintenance
tr|B7Q1Y2|B7Q1Y2_IXOSC 6-phosphogluconate dehydrogenase, decarboxylating (Frag	B7Q1Y2	6-phosphogluconate dehydrogenase	-2.15	0.003	Energy metabolism
tr|B7P230|B7P230_IXOSC Translation initiation factor 2C, putative OS=Ixodes sc	B7P230|	translation initiation factor 2C	-2.08	0.015	Protein metabolism
tr|B7QDV1|B7QDV1_IXOSC Histone, putative OS=Ixodes scapularis GN=IscW_ISCW01223	B7QDV1	histone	-2.06	0.000	Nucleic acid metabolism
tr|B7P5B3|B7P5B3_IXOSC U5 snRNP-specific protein, putative (Fragment) OS=Ixode	B7P5B3|	U5 snRNP-specific protein	-2.05	0.004	Protein metabolism
tr|B7P8J4|B7P8J4_IXOSC ATP-dependent RNA helicase, putative (Fragment) OS=Ixode	B7P8J4|	ATP-dependent RNA helicase	-2.02	0.027	Nucleic acid metabolism
tr|B7PAS1|B7PAS1_IXOSC MCM2 protein, putative (Fragment) OS=Ixodes scapularis	B7PAS1	MCM2; Predicted ATPase involved in replication control	-1.99	0.010	Cell growth and/or maintenance
tr|B7PSE0|B7PSE0_IXOSC Ribosomal protein L4, putative OS=Ixodes scapularis GN=I	B7PSE0	ribosomal protein L4	-1.96	0.000	Protein metabolism
tr|B7Q5Y2|B7Q5Y2_IXOSC Prohibitin, putative OS=Ixodes scapularis GN=IscW_ISCW0	B7Q5Y2	prohibitin	-1.92	0.000	Cell communication; Signal transduction
tr|B7PKP8|B7PKP8_IXOSC Spermidine synthase, putative OS=Ixodes scapularis GN=I	B7PKP8	spermidine synthase	-1.83	0.000	Energy metabolism
tr|B7PKR5|B7PKR5_IXOSC Glutamyl-tRNA synthetase, cytoplasmic, putative OS=Ixode	B7PKR5	glutamyl-tRNA synthetase	-1.82	0.000	Protein metabolism

tr|B7PQP7|B7PQP7_IXOSC Hydroxyacyl-CoA dehydrogenase, putative (Fragment) OS=Ix	B7PQP7	hydroxyacyl-CoA dehydrogenase	-1.81	0.000	Energy metabolism
tr|B7PMC3|B7PMC3_IXOSC Putative uncharacterized protein OS=Ixodes scapularis GN	B7PMC3	Unknown	-1.81	0.000	Unknown
tr|B7QC74|B7QC74_IXOSC Transcription factor containing NAC and TS-N domains, pu	B7QC74	transcription factor containing NAC and TS-N domains	-1.73	0.020	Nucleic acid metabolism
tr|B7PKQ6|B7PKQ6_IXOSC Cell division protein, putative (Fragment) OS=Ixodes sca	B7PKQ6	cell division protein	-1.72	0.027	Cell growth and/or maintenance
tr|B7QFX7|B7QFX7_IXOSC RAB-9 and, putative OS=Ixodes scapularis GN=IscW_ISCW021	B7QFX7	RAB-9, small Rab GTPase that regulates vesicular traffic from early to late endosomal stages of the endocytic pathway	-1.71	0.000	Cell communication; Signal transduction
tr|B7PUR9|B7PUR9_IXOSC Failed axon connections, putative OS=Ixodes scapularis G	B7PUR9	failed axon connections	-1.69	0.000	Unknown
tr|B7PA04|B7PA04_IXOSC Putative uncharacterized protein OS=Ixodes scapularis G	B7PA04|	Unknown	-1.68	0.004	Unknown
tr|B7PV22|B7PV22_IXOSC Poly [ADP-ribose] polymerase, putative OS=Ixodes scapul	B7PV22|	poly [ADP-ribose] polymerase	-1.65	0.013	Unknown
tr|B7PRG2|B7PRG2_IXOSC 60S acidic ribosomal protein P0, putative OS=Ixodes sca	B7PRG2	60S acidic ribosomal protein P0	-1.62	0.044	Protein metabolism
tr|B7P573|B7P573_IXOSC Processing peptidase beta subunit, putative OS=Ixodes sc	B7P573|	processing peptidase beta subunit	-1.59	0.011	Protein metabolism
tr|B7PIZ1|B7PIZ1_IXOSC GDI-1 GDP dissociation inhibitor, putative (Fragment) O	B7PIZ1|	GDI-1 GDP dissociation inhibitor	-1.58	0.000	Cell communication; Signal transduction
tr|B7P289|B7P289_IXOSC Prolyl 4-hydroxylase alpha subunit, putative OS=Ixodes s	B7P289|	prolyl 4-hydroxylase alpha subunit	-1.58	0.003	Protein metabolism
tr|B7PVI7|B7PVI7_IXOSC RNA-binding protein musashi, putative OS=Ixodes scapula	B7PVI7|	RNA-binding protein musashi	-1.56	0.002	Nucleic acid metabolism
tr|B7QMV1|B7QMV1_IXOSC Elongation factor, putative OS=Ixodes scapularis GN=IscW	B7QMV1	elongation factor 2 (eEF2)	-1.55	0.000	Protein metabolism
tr|B7QM86|B7QM86_IXOSC Talin, putative OS=Ixodes scapularis GN=IscW_ISCW023338	B7QM86	Talin, cytoskeletal associated protein	-1.51	0.000	Cell growth and/or maintenance
tr|B7PCN1|B7PCN1_IXOSC Aldo-keto reductase, putative OS=Ixodes scapularis GN=Is	B7PCN1	aldo-keto reductase	-1.47	0.004	Energy metabolism
tr|B7Q3Z3|B7Q3Z3_IXOSC 26S proteasome regulatory subunit rpn1, putative OS=Ixo	B7Q3Z3|	26S proteasome regulatory subunit rpn1	-1.47	0.048	Protein metabolism
tr|A4UTU3|A4UTU3_DERVA Beta-actin OS=Dermacentor variabilis PE=2 SV=2	A4UTU3	Beta actin	-1.46	0.000	Cell growth and/or maintenance

tr|B2D2D4|B2D2D4_9ACAR Translation elongation factor EF-1 alpha/Tu (Fragment)	B2D2D4	Translation elongation factor EF-1 alpha/Tu	-1.45	0.000	Protein metabolism
tr|B7P1Z8|B7P1Z8_IXOSC Heat shock protein, putative OS=Ixodes scapularis GN=Is	B7P1Z8|	HSP	-1.45	0.016	Protein metabolism
tr|B7QMD6|B7QMD6_IXOSC Transaldolase, putative OS=Ixodes scapularis GN=IscW_ISC	B7QMD6	transaldolase	-1.43	0.000	Energy metabolism
tr|B7QIJ3|B7QIJ3_IXOSC Quinone oxidoreductase, putative (Fragment) OS=Ixodes s	B7QIJ3|	quinone oxidoreductase	-1.41	0.000	Energy metabolism
tr|B7P5X8|B7P5X8_IXOSC Voltage-dependent anion-selective channel, putative OS=	B7P5X8|	voltage-dependent anion-selective channel (mt)	-1.40	0.028	Transport
tr|Q6X4W3|Q6X4W3_HAELO Actin OS=Haemaphysalis longicornis GN=Act1 PE=2 SV=1	Q6X4W3	Actin	-1.40	0.000	Cell growth and/or maintenance
tr|B7P1U8|B7P1U8_IXOSC Spectrin alpha chain, putative OS=Ixodes scapularis GN=	B7P1U8|	spectrin alpha chain, cytoskeletal protein	-1.39	0.000	Cell growth and/or maintenance
tr|B7PGM6|B7PGM6_IXOSC G-3-P dehydrogenase, putative (Fragment) OS=Ixodes scapu	B7PGM6	Glyceraldehyde 3-phosphate dehydrogenase	-1.39	0.000	Energy metabolism
sp|Q8WQ47|TBA_LEPDS Tubulin alpha chain OS=Lepidoglyphus destructor PE=1 SV=2	|Q8WQ4	Alpha tubulin	-1.37	0.000	Cell growth and/or maintenance
tr|B7QMW0|B7QMW0_IXOSC Fatty acid-binding protein FABP, putative OS=Ixodes sca	B7QMW0	fatty acid-binding protein FABP	-1.34	0.000	Transport
tr|A8UY20|A8UY20_9ACAR Elongation factor 1-alpha (Fragment) OS=Hypochthonius l	A8UY20|	elongation factor -alpha (eEF1a)	-1.32	0.003	Protein metabolism
tr|B7PG97|B7PG97_IXOSC Transcription factor NFAT, subunit NF45, putative (Frag	B7PG97	transcription factor NFAT, subunit NF45	-1.31	0.011	Nucleic acid metabolism
tr|B7PD56|B7PD56_IXOSC cyclophilin B precursor OS=Ixodes scapularis	B7PD56|	cyclophilin B precursor	-1.31	0.003	Protein metabolism
tr|B7Q0D4|B7Q0D4_IXOSC Fumarylacetoacetase, putative OS=Ixodes scapularis GN=Is	B7Q0D4	fumarylacetoacetase	-1.27	0.015	Energy metabolism
tr|B7PA92|B7PA92_IXOSC Beta tubulin OS=Ixodes scapularis GN=IscW_ISCW017133 PE	B7PA92|	beta tubulin	-1.27	0.003	Cell growth and/or maintenance
**Over-expressed in Infected cells, N=31**					
tr|B7PEN4|B7PEN4_IXOSC Heat shock protein, putative OS=Ixodes scapularis GN=Is	B7PEN4	HSP70	+ 1.20	0.011	Protein metabolism
tr|B4YTT8|B4YTT8_9ACAR Heat shock protein 70-1 OS=Tetranychus cinnabarinus PE=2	B4YTT8|	HSP70-1	+ 1.30	0.002	Protein metabolism
tr|B7Q6Z1|B7Q6Z1_IXOSC Saposin, putative	B7Q6Z1|	saposin	+ 1.37	0.000	Lipid metabolism

OS=Ixodes scapularis GN=IscW_ISCW01159					
tr|B4YTT9|B4YTT9_9ACAR Heat shock protein 702 OS=Tetranychus cinnabarinus PE=	B4YTT9|	HSP70-2	+ 1.42	0.000	Protein metabolism
tr|IscW_ISCW008184|IscW_ISCW008184 Calreticulin (Fragment) OS=Ixodes scapularis	IscW_ISCW008184	calreticulin, chaperone activity	+ 1.46	0.000	Protein metabolism
tr|B7P591|B7P591_IXOSC Phosphoribosylamidoimidazole succinocarboxamide synthas	B7P591|	phosphoribosylamidoimidazole-succinocarboxamide synthase	+ 1.46	0.000	Nucleic acid metabolism
tr|B7PV15|B7PV15_IXOSC Glyoxylate/hydroxypyruvate reductase, putative OS=Ixodes	B7PV15|	glyoxylate/hydroxypyruvate reductase	+ 1.48	0.000	Energy metabolism
tr|B7PKH2|B7PKH2_IXOSC Mcm2/3, putative (Fragment) OS=Ixodes scapularis GN=Isc	B7PKH2	minichromosome maintenance protein Mcm2/3	+ 1.49	0.030	Nucleic acid metabolism
tr|B7PBW3|B7PBW3_IXOSC Protein disulfide isomerase 1, putative OS=Ixodes scapu	B7PBW3	protein disulfide isomerase 1	+ 1.53	0.050	Protein metabolism
tr|B7PEL0|B7PEL0_IXOSC Tetraspanin, putative OS=Ixodes scapularis GN=IscW_ISCW	B7PEL0|	tetraspanin	+ 1.57	0.000	Unknown
tr|B7PRN8|B7PRN8_IXOSC Brain acid soluble protein, putative OS=Ixodes scapular	B7PRN8	brain acid soluble protein	+ 1.58	0.018	Nucleic acid metabolism
tr|A6N9M1|A6N9M1_ORNPR 40S ribosomal protein S2/30S OS=Ornithodoros parkeri PE=	A6N9M1	40S ribosomal protein S2/30S	+ 1.68	0.017	Protein metabolism
tr|B7PH44|B7PH44_IXOSC Malate dehydrogenase, putative OS=Ixodes scapularis GN=I	B7PH44|	malate dehydrogenase	+ 1.72	0.000	Energy metabolism
sp|Q09JT4|RL38_ARGMO 60S ribosomal protein L38 OS=Argas monolakensis GN=RpL38	|Q09JT4	60S ribosomal protein L38	+ 1.90	0.003	Protein metabolism
tr|B7QF39|B7QF39_IXOSC Transcription factor Mbf1, putative OS=Ixodes scapulari	B7QF39|	Transcription factor Mbf1	+ 2.02	0.003	Nucleic acid metabolism
tr|B5M799|B5M799_9ACAR Histone H2B OS=Amblyomma americanum PE=2 SV=1	B5M799|	Histone H2B	+ 2.06	0.048	Nucleic acid metabolism
tr|B7QF45|B7QF45_IXOSC 3 ketoacyl CoA thiolase, putative OS=Ixodes scapularis	B7QF45|	3-keto-acyl-CoA thiolase	+ 2.13	0.032	Protein metabolism
tr|B7Q1Y8|B7Q1Y8_IXOSC Putative uncharacterized protein OS=Ixodes scapularis G	B7Q1Y8	Unknown	+ 2.18	0.039	Unknown
tr|B7PZ14|B7PZ14_IXOSC RNA binding protein, putative OS=Ixodes scapularis GN=I	B7PZ14|	RNA-binding protein	+ 2.20	0.011	Nucleic acid metabolism
tr|B7Q5H9|B7Q5H9_IXOSC Fructose bisphosphate aldolase OS=Ixodes scapularis	B7Q5H9	fructose 1,6-bisphosphate aldolase	+ 2.23	0.004	Energy metabolism

GN=I					
tr|B7PHT2|B7PHT2_IXOSC Histone H2A OS=Ixodes scapularis GN=IscW_ISCW004478 PE=	B7PHT2	Histone H2A	+ 2.72	0.000	Nucleic acid metabolism
tr|B7Q645|B7Q645_IXOSC Secreted salivary gland peptide, putative (Fragment) OS	B7Q645|	secreted salivary gland peptide	+ 2.81	0.000	Protein metabolism
tr|B7Q4T5|B7Q4T5_IXOSC Putative uncharacterized protein OS=Ixodes scapularis GN	B7Q4T5|	Unknown	+ 2.83	0.000	Unknown
tr|B7Q0Q1|B7Q0Q1_IXOSC Putative uncharacterized protein (Fragment) OS=Ixodes sc	B7Q0Q1	Unknown	+ 2.91	0.002	Unknown
tr|B7PB95|B7PB95_IXOSC Stathmin OS=Ixodes scapularis GN=IscW_ISCW003366 PE=3 S	B7PB95|	stathmin	+ 2.96	0.000	Cell communication; Signal transduction
tr|B7QD48|B7QD48_IXOSC Putative uncharacterized protein (Fragment) OS=Ixodes sc	B7QD48	Unknown	+ 3.14	0.037	Unknown
tr|A6N9P0|A6N9P0_ORNPR 40S ribosomal protein S14 OS=Ornithodoros parkeri PE=2 S	A6N9P0|	ribosomal protein S14	+ 3.22	0.000	Protein metabolism
tr|B7PKZ9|B7PKZ9_IXOSC BRI1 KD interacting protein, putative OS=Ixodes scapula	B7PKZ9|	BRI1-KD interacting protein	+ 3.98	0.014	Protein metabolism
tr|Q86G66|Q86G66_DERVA Putative beta thymosin OS=Dermacentor variabilis PE=2 SV	Q86G66	beta thymosin	+ 4.68	0.000	Cell growth and/or maintenance
tr|B7P3Q5|B7P3Q5_IXOSC Vasa intronic protein, putative OS=Ixodes scapularis GN	B7P3Q5	vasa intronic protein	+ 4.81	0.000	Nucleic acid metabolism
tr|B7PXR5|B7PXR5_IXOSC Chaperonin complex component, TCP1 eta subunit, putativ	B7PXR5	chaperonin complex component, TCP-1b eta subunit	+ 16.16	0.004	Protein metabolism

[a] + indicates a significant increase in protein levels and - indicates a significant decrease in protein levels in infected cells ($p < 0.05$).
[b]False discovery rate (FDR) associated to protein identification.
[c]Protein ontology for biological process determined using human protein databases at: http://www.hprd.org / and http://www.ebi.ac.uk/interpro/

Supplementary Table 35. Protein identification in *Ixodes scapularis* ISE6 cells infected with *Anaplasma phagocytophilum*.

FASTA protein Description	Species	No. Peptides[a]	FDR[b]
**Proteins identified with FDR <1%**			
tr|B7PEV0|B7PEV0_IXOSC Chaperonin subunit, putative OS=Ixodes scapularis GN=Isc	*Ixodes scapularis*	12	0.000
sp|Q8WQ47|TBA_LEPDS Tubulin alpha chain OS=Lepidoglyphus destructor PE=1 SV=2	*Lepidoglyphus destructor*	10	0.000
tr|B7PEN4|B7PEN4_IXOSC Heat shock protein, putative OS=Ixodes scapularis GN=Isc	*Ixodes scapularis*	10	0.000
tr|B7QI01|B7QI01_IXOSC Hsp90 protein, putative OS=Ixodes scapularis GN=IscW_IS	*Ixodes scapularis*	9	0.000
tr|B7P1U8|B7P1U8_IXOSC Spectrin alpha chain, putative OS=Ixodes scapularis GN=	*Ixodes scapularis*	8	0.000
tr|B7Q5X7|B7Q5X7_IXOSC Vinculin, putative OS=Ixodes scapularis GN=IscW_ISCW0214	*Ixodes scapularis*	8	0.000
tr|B7Q9F1|B7Q9F1_IXOSC Protein disulfide isomerase, putative OS=Ixodes scapular	*Ixodes scapularis*	8	0.000
tr|B7QIT3|B7QIT3_IXOSC Putative uncharacterized protein OS=Ixodes scapularis GN	*Ixodes scapularis*	7	0.000
tr|B7P8Q5|B7P8Q5_IXOSC Hsp70, putative (Fragment) OS=Ixodes scapularis GN=IscW	*Ixodes scapularis*	6	0.000
tr|B7Q0J9|B7Q0J9_IXOSC Peptidyl-prolyl cis-trans isomerase OS=Ixodes scapulari	*Ixodes scapularis*	6	0.000
tr|B7QAM1|B7QAM1_IXOSC Chaperonin complex component, TCP-1 theta subunit, putat	*Ixodes scapularis*	6	0.000
tr|B7QC85|B7QC85_IXOSC Tumor rejection antigen (Gp96), putative (Fragment) OS=I	*Ixodes scapularis*	6	0.000
tr|B7QM86|B7QM86_IXOSC Talin, putative OS=Ixodes scapularis GN=IscW_ISCW023338	*Ixodes scapularis*	6	0.000
tr|B7QMV1|B7QMV1_IXOSC Elongation factor, putative OS=Ixodes scapularis GN=IscW	*Ixodes scapularis*	6	0.000
tr|B7P3Z6|B7P3Z6_IXOSC Chaperonin complex component, TCP-1 gamma subunit, putat	*Ixodes scapularis*	5	0.000
tr|B7P4U1|B7P4U1_IXOSC Protein disulfide isomerase, putative OS=Ixodes scapular	*Ixodes scapularis*	5	0.000
tr|B7PA92|B7PA92_IXOSC Beta tubulin OS=Ixodes scapularis GN=IscW_ISCW017133 PE=	*Ixodes scapularis*	5	0.000
tr|B7PG97|B7PG97_IXOSC Transcription factor NFAT, subunit NF45, putative (Fragm	*Ixodes scapularis*	5	0.000
tr|B7PN34|B7PN34_IXOSC KH domain RNA binding protein, putative (Fragment) OS=Ix	*Ixodes scapularis*	5	0.000
tr|B7PUR9|B7PUR9_IXOSC Failed axon connections, putative OS=Ixodes scapularis G	*Ixodes scapularis*	5	0.000
tr|B7PX63|B7PX63_IXOSC Zinc finger protein, putative OS=Ixodes scapularis GN=Is	*Ixodes scapularis*	5	0.000
tr|B7Q0D4|B7Q0D4_IXOSC Fumarylacetoacetase, putative OS=Ixodes scapularis GN=Is	*Ixodes scapularis*	5	0.000
tr|B7QE46|B7QE46_IXOSC ATP synthase subunit beta OS=Ixodes scapularis GN=IscW_	*Ixodes scapularis*	5	0.000
tr|B5AHF4|B5AHF4_9ACAR Heat shock protein 90 OS=Tetranychus cinnabarinus PE=2 S	*Tetranychus cinnabarinus*	5	0.000
tr|A4UTU3|A4UTU3_DERVA Beta-actin OS=Dermacentor variabilis PE=2 SV=2	*Dermacentor variabilis*	5	0.000
tr|A0S0Q6|A0S0Q6_9ACAR Actin (Fragment) OS=Neoseiulus womersleyi PE=2 SV=1	*Neoseiulus womersleyi*	4	0.000
tr|B7P1Z8|B7P1Z8_IXOSC Heat shock protein, putative OS=Ixodes scapularis GN=Isc	*Ixodes scapularis*	4	0.000
tr|B7PAR6|B7PAR6_IXOSC Heat shock protein, putative OS=Ixodes scapularis GN=Isc	*Ixodes scapularis*	4	0.000

tr|B7PH44|B7PH44_IXOSC Malate dehydrogenase, putative OS=Ixodes scapularis GN=I	*Ixodes scapularis*	4	0.000
tr|B7PIM5|B7PIM5_IXOSC CNDP dipeptidase, putative (Fragment) OS=Ixodes scapular	*Ixodes scapularis*	4	0.000
tr|B7Q5G8|B7Q5G8_IXOSC Spectrin beta chain, putative OS=Ixodes scapularis GN=Is	*Ixodes scapularis*	4	0.000
tr|B7Q5Y2|B7Q5Y2_IXOSC Prohibitin, putative OS=Ixodes scapularis GN=IscW_ISCW0	*Ixodes scapularis*	4	0.000
tr|B7QCK2|B7QCK2_IXOSC ATP synthase subunit alpha OS=Ixodes scapularis GN=IscW_	*Ixodes scapularis*	4	0.000
tr|B7QJ21|B7QJ21_IXOSC Chaperonin complex component, TCP-1 eta subunit, putati	*Ixodes scapularis*	4	0.000
tr|B5M6E6|B5M6E6_HAPSC Beta tubulin OS=Haplopelma schmidti PE=2 SV=1	*Haplopelma schmidti*	4	0.000
tr|A1KXJ1|A1KXJ1_BLOTA Blo t Mag29 allergen OS=Blomia tropicalis PE=2 SV=1	*Blomia tropicalis*	3	0.000
tr|B7P0M7|B7P0M7_IXOSC Aldehyde dehydrogenase, putative (Fragment) OS=Ixodes s	*Ixodes scapularis*	3	0.000
tr|B7P5X8|B7P5X8_IXOSC Voltage-dependent anion-selective channel, putative OS=I	*Ixodes scapularis*	3	0.000
tr|B7PAB9|B7PAB9_IXOSC Methylmalonate semialdehyde dehydrogenase, putative OS=I	*Ixodes scapularis*	3	0.000
tr|B7PDF3|B7PDF3_IXOSC FKBP-type peptidyl-prolyl cis-trans isomerase, putative	*Ixodes scapularis*	3	0.000
tr|B7PHC3|B7PHC3_IXOSC Carbon-nitrogen hydrolase, putative OS=Ixodes scapularis	*Ixodes scapularis*	3	0.000
tr|B7PHJ5|B7PHJ5_IXOSC Cytochrome b5 domain-containing protein, putative (Fragm	*Ixodes scapularis*	3	0.000
tr|B7PKG2|B7PKG2_IXOSC Fasciclin domain-containing protein, putative OS=Ixodes	*Ixodes scapularis*	3	0.000
tr|B7PKR5|B7PKR5_IXOSC Glutamyl-tRNA synthetase, cytoplasmic, putative OS=Ixode	*Ixodes scapularis*	3	0.000
tr|B7PRN8|B7PRN8_IXOSC Brain acid soluble protein, putative OS=Ixodes scapular	*Ixodes scapularis*	3	0.000
tr|B7PSE0|B7PSE0_IXOSC Ribosomal protein L4, putative OS=Ixodes scapularis GN=I	*Ixodes scapularis*	3	0.000
tr|B7PV15|B7PV15_IXOSC Glyoxylate/hydroxypyruvate reductase, putative OS=Ixodes	*Ixodes scapularis*	3	0.000
tr|B7Q5I4|B7Q5I4_IXOSC Multifunctional chaperone, putative OS=Ixodes scapulari	*Ixodes scapularis*	3	0.000
tr|B7Q5L2|B7Q5L2_IXOSC Calponin, putative OS=Ixodes scapularis GN=IscW_ISCW021	*Ixodes scapularis*	3	0.000
tr|B7QEE0|B7QEE0_IXOSC Hypoxia up-regulated protein, putative OS=Ixodes scapula	*Ixodes scapularis*	3	0.000
tr|B7QGH2|B7QGH2_IXOSC Glutathione S-transferase, putative OS=Ixodes scapularis	*Ixodes scapularis*	3	0.000
tr|B7QMW0|B7QMW0_IXOSC Fatty acid-binding protein FABP, putative OS=Ixodes sca	*Ixodes scapularis*	3	0.000
tr|B4YTT9|B4YTT9_9ACAR Heat shock protein 70-2 OS=Tetranychus cinnabarinus PE=	*Tetranychus cinnabarinus*	3	0.000
tr|A6N9Z0|A6N9Z0_ORNPR Ubiquitin/40S ribosomal protein S27a OS=Ornithodoros par	*Ornithodoros parkeri*	3	0.000
tr|A6NA14|A6NA14_ORNPR Truncated peroxiredoxin (Fragment) OS=Ornithodoros parke	*Ornithodoros parkeri*	3	0.000
tr|B7P3B9|B7P3B9_IXOSC Lumican, putative OS=Ixodes scapularis GN=IscW_ISCW00102	*Ixodes scapularis*	2	0.000
tr|B7P3M8|B7P3M8_IXOSC D-3-phosphoglycerate dehydrogenase, putative (Fragment)	*Ixodes scapularis*	2	0.000
tr|B7P427|B7P427_IXOSC Transmembrane protein Tmp21, putative OS=Ixodes scapular	*Ixodes scapularis*	2	0.000
tr|B7P526|B7P526_IXOSC Reductase, putative OS=Ixodes scapularis GN=IscW_ISCW00	*Ixodes scapularis*	2	0.000
tr|B7P591|B7P591_IXOSC Phosphoribosylamidoimidazole-succinocarboxamide synthas	*Ixodes scapularis*	2	0.000

tr|B7P5U7|B7P5U7_IXOSC Lon protease homolog (Fragment) OS=Ixodes scapularis GN=	*Ixodes scapularis*	2	0.000
tr|B7PA04|B7PA04_IXOSC Putative uncharacterized protein OS=Ixodes scapularis GN	*Ixodes scapularis*	2	0.000
tr|B7PA24|B7PA24_IXOSC Protein phosphatase 2A regulatory subunit A, putative OS	*Ixodes scapularis*	2	0.000
tr|B7PBW3|B7PBW3_IXOSC Protein disulfide isomerase 1, putative OS=Ixodes scapul	*Ixodes scapularis*	2	0.000
tr|B7PCL8|B7PCL8_IXOSC Hydroxysteroid (17-beta) dehydrogenase, putative OS=Ixo	*Ixodes scapularis*	2	0.000
tr|B7PEU9|B7PEU9_IXOSC Heat shock protein OS=Ixodes scapularis GN=IscW_ISCW0178	*Ixodes scapularis*	2	0.000
tr|B7PEY5|B7PEY5_IXOSC Alanyl-tRNA synthetase, putative OS=Ixodes scapularis G	*Ixodes scapularis*	2	0.000
tr|B7PGM6|B7PGM6_IXOSC G-3-P dehydrogenase, putative (Fragment) OS=Ixodes scapu	*Ixodes scapularis*	2	0.000
tr|B7PIZ1|B7PIZ1_IXOSC GDI-1 GDP dissociation inhibitor, putative (Fragment) OS	*Ixodes scapularis*	2	0.000
tr|B7PMY6|B7PMY6_IXOSC Actin depolymerizing factor, putative OS=Ixodes scapula	*Ixodes scapularis*	2	0.000
tr|B7PTR3|B7PTR3_IXOSC Limbic system-associated membrane protein, putative OS=I	*Ixodes scapularis*	2	0.000
tr|B7PUK8|B7PUK8_IXOSC Clathrin heavy chain, putative (Fragment) OS=Ixodes scap	*Ixodes scapularis*	2	0.000
tr|B7PYE7|B7PYE7_IXOSC B-cell receptor-associated protein, putative OS=Ixodes s	*Ixodes scapularis*	2	0.000
tr|B7Q0D5|B7Q0D5_IXOSC Pyruvate kinase OS=Ixodes scapularis GN=IscW_ISCW020197	*Ixodes scapularis*	2	0.000
tr|B7Q4P0|B7Q4P0_IXOSC Putative uncharacterized protein OS=Ixodes scapularis GN	*Ixodes scapularis*	2	0.000
tr|B7Q4T5|B7Q4T5_IXOSC Putative uncharacterized protein OS=Ixodes scapularis GN	*Ixodes scapularis*	2	0.000
tr|B7Q6Y2|B7Q6Y2_IXOSC Chaperonin subunit, putative OS=Ixodes scapularis GN=Is	*Ixodes scapularis*	2	0.000
tr|B7Q8W6|B7Q8W6_IXOSC Alkyl hydroperoxide reductase, thiol specific antioxida	*Ixodes scapularis*	2	0.000
tr|B7QAW3|B7QAW3_IXOSC Electron transfer flavoprotein, beta subunit, putative O	*Ixodes scapularis*	2	0.000
tr|B7QBM8|B7QBM8_IXOSC Enoyl-CoA hydratase, putative OS=Ixodes scapularis GN=Is	*Ixodes scapularis*	2	0.000
tr|B7QC74|B7QC74_IXOSC Transcription factor containing NAC and TS-N domains, pu	*Ixodes scapularis*	2	0.000
tr|B7QFN6|B7QFN6_IXOSC Proliferating cell nuclear antigen OS=Ixodes scapularis	*Ixodes scapularis*	2	0.000
tr|B7QGQ3|B7QGQ3_IXOSC Putative uncharacterized protein OS=Ixodes scapularis GN	*Ixodes scapularis*	2	0.000
tr|B7QHT2|B7QHT2_IXOSC Profilin (Fragment) OS=Ixodes scapularis GN=IscW_ISCW023	*Ixodes scapularis*	2	0.000
tr|B7QIJ3|B7QIJ3_IXOSC Quinone oxidoreductase, putative (Fragment) OS=Ixodes s	*Ixodes scapularis*	2	0.000
tr|B7QL57|B7QL57_IXOSC Adenylyl cyclase-associated protein OS=Ixodes scapulari	*Ixodes scapularis*	2	0.000
tr|B7QLY6|B7QLY6_IXOSC Nucleoside diphosphate kinase OS=Ixodes scapularis GN=Is	*Ixodes scapularis*	2	0.000
tr|B7QMD6|B7QMD6_IXOSC Transaldolase, putative OS=Ixodes scapularis GN=IscW_ISC	*Ixodes scapularis*	2	0.000
tr|Q64K73|Q64K73_9ACAR Calreticulin (Fragment) OS=Ixodes woodi PE=3 SV=1	*Ixodes woodi*	2	0.000
tr|A5LHV9|A5LHV9_HAELO Protein disulfide isomerase-2 OS=Haemaphysalis longicorn	*Haemaphysalis longicornis*	2	0.000
tr|A6N9S1|A6N9S1_ORNPR Thioredoxin peroxidase OS=Ornithodoros parkeri PE=2 SV=	*Ornithodoros parkeri*	2	0.000
tr|A9Y1V1|A9Y1V1_HAELO Ribosomal protein P0 OS=Haemaphysalis longicornis PE=2 S	*Haemaphysalis*	2	0.000

	longicornis		
tr|A9XYV8|A9XYV8_MASGI Putative uncharacterized protein (Fragment) OS=Mastigopr	*Mastigoproctus giganteus*	1	0.000
tr|B4YTU0|B4YTU0_9ACAR Heat shock protein 70-3 OS=Tetranychus cinnabarinus PE=	*Tetranychus cinnabarinus*	1	0.000
sp|Q4PLZ3|TCTP_IXOSC Translationally-controlled tumor protein homolog OS=Ixodes	*Ixodes scapularis*	1	0.000
tr|B7P1C8|B7P1C8_IXOSC Protein hu-li tai shao, putative OS=Ixodes scapularis GN	*Ixodes scapularis*	1	0.000
tr|B7P1U0|B7P1U0_IXOSC GTP-specific succinyl-CoA synthetase, beta subunit, put	*Ixodes scapularis*	1	0.000
tr|B7P201|B7P201_IXOSC Ran GTPase-activating protein, putative OS=Ixodes scapul	*Ixodes scapularis*	1	0.000
tr|B7P2P8|B7P2P8_IXOSC ATP synthase alpha subunit vacuolar, putative (Fragment)	*Ixodes scapularis*	1	0.000
tr|B7P2Q4|B7P2Q4_IXOSC Lamin, putative OS=Ixodes scapularis GN=IscW_ISCW000339	*Ixodes scapularis*	1	0.000
tr|B7P328|B7P328_IXOSC Superoxide dismutase (Fragment) OS=Ixodes scapularis GN	*Ixodes scapularis*	1	0.000
tr|B7P361|B7P361_IXOSC 26S protease regulatory subunit 6B, putative OS=Ixodes s	*Ixodes scapularis*	1	0.000
tr|B7P363|B7P363_IXOSC Ufm1-conjugating enzyme, putative OS=Ixodes scapularis	*Ixodes scapularis*	1	0.000
tr|B7P3A9|B7P3A9_IXOSC Coatomer delta subunit, putative OS=Ixodes scapularis GN	*Ixodes scapularis*	1	0.000
tr|B7P3G6|B7P3G6_IXOSC Medium-chain acyl-CoA dehydrogenase, putative OS=Ixodes	*Ixodes scapularis*	1	0.000
tr|B7P3N4|B7P3N4_IXOSC Cytochrome P450, putative OS=Ixodes scapularis GN=IscW_I	*Ixodes scapularis*	1	0.000
tr|B7P462|B7P462_IXOSC Putative uncharacterized protein (Fragment) OS=Ixodes sc	*Ixodes scapularis*	1	0.000
tr|B7P4M6|B7P4M6_IXOSC Tyrosyl-tRNA synthetase, putative OS=Ixodes scapularis	*Ixodes scapularis*	1	0.000
tr|B7P557|B7P557_IXOSC Mapmodulin, putative OS=Ixodes scapularis GN=IscW_ISCW00	*Ixodes scapularis*	1	0.000
tr|B7P5C4|B7P5C4_IXOSC Translation initiation factor 4F, helicase subunit, puta	*Ixodes scapularis*	1	0.000
tr|B7P6A9|B7P6A9_IXOSC ATP synthase subunit beta OS=Ixodes scapularis GN=IscW_I	*Ixodes scapularis*	1	0.000
tr|B7P6P0|B7P6P0_IXOSC Glycoprotein 25l, putative OS=Ixodes scapularis GN=IscW_	*Ixodes scapularis*	1	0.000
tr|B7P7P7|B7P7P7_IXOSC Apoptosis inhibitor, putative OS=Ixodes scapularis GN=I	*Ixodes scapularis*	1	0.000
tr|B7P7U3|B7P7U3_IXOSC Chloride channel, putative OS=Ixodes scapularis GN=IscW	*Ixodes scapularis*	1	0.000
tr|B7P839|B7P839_IXOSC DEK domain-containing protein, putative OS=Ixodes scapu	*Ixodes scapularis*	1	0.000
tr|B7P9E4|B7P9E4_IXOSC Na+/K+ ATPase, alpha subunit, putative (Fragment) OS=Ixo	*Ixodes scapularis*	1	0.000
tr|B7PB95|B7PB95_IXOSC Stathmin OS=Ixodes scapularis GN=IscW_ISCW003366 PE=3 S	*Ixodes scapularis*	1	0.000
tr|B7PBJ3|B7PBJ3_IXOSC Putative uncharacterized protein (Fragment) OS=Ixodes sc	*Ixodes scapularis*	1	0.000
tr|B7PDF5|B7PDF5_IXOSC Prolyl endopeptidase, putative OS=Ixodes scapularis GN=I	*Ixodes scapularis*	1	0.000
tr|B7PEA9|B7PEA9_IXOSC 40S ribosomal protein, putative OS=Ixodes scapularis GN=	*Ixodes scapularis*	1	0.000
tr|B7PEL0|B7PEL0_IXOSC Tetraspanin, putative OS=Ixodes scapularis GN=IscW_ISCW0	*Ixodes scapularis*	1	0.000
tr|B7PH43|B7PH43_IXOSC Alpha tubulin OS=Ixodes scapularis GN=IscW_ISCW003527 PE	*Ixodes scapularis*	1	0.000
tr|B7PHG9|B7PHG9_IXOSC ATPase, putative OS=Ixodes scapularis GN=IscW_ISCW01829	*Ixodes scapularis*	1	0.000

tr|B7PHT2|B7PHT2_IXOSC Histone H2A OS=Ixodes scapularis GN=IscW_ISCW004478 PE=	*Ixodes scapularis*	1	0.000
tr|B7PIN1|B7PIN1_IXOSC Heat shock protein 20.6, putative OS=Ixodes scapularis G	*Ixodes scapularis*	1	0.000
tr|B7PJ70|B7PJ70_IXOSC Reticulon/nogo, putative OS=Ixodes scapularis GN=IscW_IS	*Ixodes scapularis*	1	0.000
tr|B7PKH2|B7PKH2_IXOSC Mcm2/3, putative (Fragment) OS=Ixodes scapularis GN=Isc	*Ixodes scapularis*	1	0.000
tr|B7PKL1|B7PKL1_IXOSC Neurofilament medium polypeptide, putative (Fragment) OS	*Ixodes scapularis*	1	0.000
tr|B7PKP8|B7PKP8_IXOSC Spermidine synthase, putative OS=Ixodes scapularis GN=I	*Ixodes scapularis*	1	0.000
tr|B7PL04|B7PL04_IXOSC Pyruvate decarboxylase (E-1) alpha subunit, putative (Fr	*Ixodes scapularis*	1	0.000
tr|B7PL25|B7PL25_IXOSC Double-stranded RNA-specific editase B2, putative OS=Ix	*Ixodes scapularis*	1	0.000
tr|B7PMC3|B7PMC3_IXOSC Putative uncharacterized protein OS=Ixodes scapularis GN	*Ixodes scapularis*	1	0.000
tr|B7PNG4|B7PNG4_IXOSC Alpha tubulin, putative OS=Ixodes scapularis GN=IscW_ISC	*Ixodes scapularis*	1	0.000
tr|B7PNN1|B7PNN1_IXOSC Proteasome subunit alpha type OS=Ixodes scapularis GN=Is	*Ixodes scapularis*	1	0.000
tr|B7PPI3|B7PPI3_IXOSC Secreted protein, putative OS=Ixodes scapularis GN=IscW_	*Ixodes scapularis*	1	0.000
tr|B7PPL3|B7PPL3_IXOSC Microtubule-binding protein, putative OS=Ixodes scapular	*Ixodes scapularis*	1	0.000
tr|B7PQP7|B7PQP7_IXOSC Hydroxyacyl-CoA dehydrogenase, putative (Fragment) OS=Ix	*Ixodes scapularis*	1	0.000
tr|B7PR83|B7PR83_IXOSC Ubiquitin conjugating enzyme E1, putative OS=Ixodes scap	*Ixodes scapularis*	1	0.000
tr|B7PT52|B7PT52_IXOSC Embryonic protein DC-8, putative OS=Ixodes scapularis GN	*Ixodes scapularis*	1	0.000
tr|B7PVG5|B7PVG5_IXOSC GTP-binding protein, putative OS=Ixodes scapularis GN=Is	*Ixodes scapularis*	1	0.000
tr|B7PVL8|B7PVL8_IXOSC Guanine nucleotide-binding protein G, putative (Fragment	*Ixodes scapularis*	1	0.000
tr|B7PWM5|B7PWM5_IXOSC Alternative splicing factor SRp20/9G8, putative OS=Ixode	*Ixodes scapularis*	1	0.000
tr|B7PWY6|B7PWY6_IXOSC Ubiquitin carboxyl-terminal hydrolase OS=Ixodes scapular	*Ixodes scapularis*	1	0.000
tr|B7PZ24|B7PZ24_IXOSC Chaperonin complex component, TCP-1 delta subunit, putat	*Ixodes scapularis*	1	0.000
tr|B7PZR4|B7PZR4_IXOSC Surfeit 4 protein, putative OS=Ixodes scapularis GN=IscW	*Ixodes scapularis*	1	0.000
tr|B7Q0D6|B7Q0D6_IXOSC Phosphoserine aminotransferase, putative OS=Ixodes scapu	*Ixodes scapularis*	1	0.000
tr|B7Q121|B7Q121_IXOSC Putative uncharacterized protein OS=Ixodes scapularis GN	*Ixodes scapularis*	1	0.000
tr|B7Q1V4|B7Q1V4_IXOSC Galectin, putative OS=Ixodes scapularis GN=IscW_ISCW008	*Ixodes scapularis*	1	0.000
tr|B7Q2W2|B7Q2W2_IXOSC UTP-glucose-1-phosphate uridylyltransferase, putative (F	*Ixodes scapularis*	1	0.000
tr|B7Q3I2|B7Q3I2_IXOSC Citrate synthase (Fragment) OS=Ixodes scapularis GN=IscW	*Ixodes scapularis*	1	0.000
tr|B7Q5F6|B7Q5F6_IXOSC Proteasome subunit alpha type OS=Ixodes scapularis GN=Is	*Ixodes scapularis*	1	0.000
tr|B7Q645|B7Q645_IXOSC Secreted salivary gland peptide, putative (Fragment) OS	*Ixodes scapularis*	1	0.000
tr|B7Q6Z1|B7Q6Z1_IXOSC Saposin, putative OS=Ixodes scapularis GN=IscW_ISCW01159	*Ixodes scapularis*	1	0.000
tr|B7Q8U6|B7Q8U6_IXOSC Adenosine kinase, putative OS=Ixodes scapularis GN=IscW_	*Ixodes scapularis*	1	0.000
tr|B7QAP3|B7QAP3_IXOSC Dihydropteridine reductase, putative OS=Ixodes scapulari	*Ixodes scapularis*	1	0.000

tr|B7QDB1|B7QDB1_IXOSC Ubiquitin carboxyl-terminal hydrolase (Fragment) OS=Ixo	*Ixodes scapularis*	1	0.000
tr|B7QDV1|B7QDV1_IXOSC Histone, putative OS=Ixodes scapularis GN=IscW_ISCW01223	*Ixodes scapularis*	1	0.000
tr|B7QE67|B7QE67_IXOSC Proteasome subunit alpha type OS=Ixodes scapularis GN=Is	*Ixodes scapularis*	1	0.000
tr|B7QF40|B7QF40_IXOSC Proteasome, subunit beta, putative OS=Ixodes scapularis	*Ixodes scapularis*	1	0.000
tr|B7QFX7|B7QFX7_IXOSC RAB-9 and, putative OS=Ixodes scapularis GN=IscW_ISCW021	*Ixodes scapularis*	1	0.000
tr|B7QHA1|B7QHA1_IXOSC Ubiquitin carboxyl-terminal hydrolase isozyme L3, putat	*Ixodes scapularis*	1	0.000
tr|B7QJ52|B7QJ52_IXOSC Transcriptional regulator DJ-1, putative OS=Ixodes scap	*Ixodes scapularis*	1	0.000
tr|B7QJH6|B7QJH6_IXOSC Alpha-actinin, putative OS=Ixodes scapularis GN=IscW_ISC	*Ixodes scapularis*	1	0.000
tr|B7QLE3|B7QLE3_IXOSC Protein kinase C substrate, 80 KD protein, heavy chain,	*Ixodes scapularis*	1	0.000
tr|B7QNN4|B7QNN4_IXOSC Protein arginine N-methyltransferase PRMT1, putative OS=	*Ixodes scapularis*	1	0.000
tr|Q4PM51|Q4PM51_IXOSC Translation initiation factor 5A (Fragment) OS=Ixodes sc	*Ixodes scapularis*	1	0.000
tr|Q4VRW1|Q4VRW1_IXOSC Nucleotidase 4F8 OS=Ixodes scapularis PE=2 SV=1	*Ixodes scapularis*	1	0.000
tr|A6N9P0|A6N9P0_ORNPR 40S ribosomal protein S14 OS=Ornithodoros parkeri PE=2 S	*Ornithodoros parkeri*	1	0.000
tr|B0LAI9|B0LAI9_9ACAR Glutathione S-transferase mu class OS=Rhipicephalus annu	*Rhipicephalus annulatus*	1	0.000
tr|B2D2D4|B2D2D4_9ACAR Translation elongation factor EF-1 alpha/Tu (Fragment)	*Ornithodoros coriaceus*	1	0.000
tr|B5M792|B5M792_9ACAR Heterogeneous nuclear ribonucleoprotein (Fragment) OS=Am	*Amblyomma americanum*	1	0.000
tr|Q6X4W3|Q6X4W3_HAELO Actin OS=Haemaphysalis longicornis GN=Act1 PE=2 SV=1	*Haemaphysalis longicornis*	1	0.000
tr|Q86G66|Q86G66_DERVA Putative beta thymosin OS=Dermacentor variabilis PE=2 SV	*Dermacentor variabilis*	1	0.000
tr|B7PC41|B7PC41_IXOSC Scavenger receptor class B type I, putative OS=Ixodes s	*Ixodes scapularis*	1	0.002
tr|B7Q9Z3|B7Q9Z3_IXOSC Proteasome subunit alpha type (Fragment) OS=Ixodes scapu	*Ixodes scapularis*	1	0.002
tr|B7Q634|B7Q634_IXOSC Cap binding protein, putative OS=Ixodes scapularis GN=I	*Ixodes scapularis*	1	0.002
tr|B7PVI7|B7PVI7_IXOSC RNA-binding protein musashi, putative OS=Ixodes scapula	*Ixodes scapularis*	3	0.002
tr|B7P625|B7P625_IXOSC Prohibitin, putative OS=Ixodes scapularis GN=IscW_ISCW00	*Ixodes scapularis*	2	0.002
tr|B7P950|B7P950_IXOSC DNA-binding protein, putative OS=Ixodes scapularis GN=I	*Ixodes scapularis*	2	0.002
tr|B7QMM1|B7QMM1_IXOSC Glycine C-acetyltransferase/2-amino-3-ketobutyrate-CoA l	*Ixodes scapularis*	1	0.002
tr|B7QIG3|B7QIG3_IXOSC Electron transfer flavoprotein, alpha subunit, putative	*Ixodes scapularis*	4	0.002
tr|B4YTT8|B4YTT8_9ACAR Heat shock protein 70-1 OS=Tetranychus cinnabarinus PE=2	*Tetranychus cinnabarinus*	3	0.002
tr|Q4PM16|Q4PM16_IXOSC 60S ribosomal protein L23 OS=Ixodes scapularis PE=2 SV=	*Ixodes scapularis*	1	0.002
tr|B7Q505|B7Q505_IXOSC Elongation factor Tu OS=Ixodes scapularis GN=IscW_ISCW0	*Ixodes scapularis*	3	0.002
tr|A6NA07|A6NA07_ORNPR 60S ribosomal protein L9 OS=Ornithodoros parkeri PE=2 S	*Ornithodoros parkeri*	1	0.002
tr|B7QH63|B7QH63_IXOSC Putative uncharacterized protein OS=Ixodes scapularis G	*Ixodes scapularis*	1	0.002
tr|B7QIL1|B7QIL1_IXOSC FERM, RhoGEF and pleckstrin domain-containing protein,	*Ixodes scapularis*	1	0.002

tr|B7Q0K8|B7Q0K8_IXOSC Ribosome biogenesis protein-Nop58p/Nop5p, putative OS=I	*Ixodes scapularis*	2	0.002
tr|B7PJP9|B7PJP9_IXOSC Enolase OS=Ixodes scapularis GN=IscW_ISCW017666 PE=3 SV=	*Ixodes scapularis*	1	0.002
tr|A7BFI9|A7BFI9_HAELO Valosin containing protein OS=Haemaphysalis longicornis	*Haemaphysalis longicornis*	3	0.002
tr|B7P0P1|B7P0P1_IXOSC DNA topoisomerase 2 OS=Ixodes scapularis GN=IscW_ISCW01	*Ixodes scapularis*	2	0.002
tr|B7QGH7|B7QGH7_IXOSC Ataxin-10, putative OS=Ixodes scapularis GN=IscW_ISCW022	*Ixodes scapularis*	1	0.002
tr|B7Q0R0|B7Q0R0_IXOSC Phosphoglycerate mutase, putative OS=Ixodes scapularis G	*Ixodes scapularis*	1	0.002
tr|B7PZP2|B7PZP2_IXOSC Putative uncharacterized protein (Fragment) OS=Ixodes s	*Ixodes scapularis*	2	0.002
tr|B7PXG2|B7PXG2_IXOSC Glycoprotein gC1qBP, putative OS=Ixodes scapularis GN=I	*Ixodes scapularis*	1	0.002
tr|B7QF39|B7QF39_IXOSC Transcription factor Mbf1, putative OS=Ixodes scapulari	*Ixodes scapularis*	2	0.002
tr|B7Q5K4|B7Q5K4_IXOSC Radixin, moesin, putative OS=Ixodes scapularis GN=IscW_	*Ixodes scapularis*	1	0.002
tr|B7P7A5|B7P7A5_IXOSC Ribophorin, putative (Fragment) OS=Ixodes scapularis GN=	*Ixodes scapularis*	3	0.002
tr|A1DZP1|A1DZP1_9ACAR Elongation factor 1alpha (Fragment) OS=Rhysotritia dupli	*Rhysotritia duplicata*	1	0.002
tr|B7Q6G7|B7Q6G7_IXOSC Flavonol reductase/cinnamoyl-CoA reductase, putative (F	*Ixodes scapularis*	3	0.002
tr|B7PSK1|B7PSK1_IXOSC Vacuolar sorting protein VPS28, putative OS=Ixodes scapu	*Ixodes scapularis*	1	0.002
tr|B7PDE1|B7PDE1_IXOSC 26S proteasome non-ATPase regulatory subunit, putative	*Ixodes scapularis*	1	0.002
tr|A0SHR2|A0SHR2_AMBVA Protein disulfide isomerase OS=Amblyomma variegatum PE=2	*Amblyomma variegatum*	3	0.002
tr|B7PJY6|B7PJY6_IXOSC Flavonol reductase/cinnamoyl-CoA reductase, putative OS=	*Ixodes scapularis*	2	0.003
tr|B7Q6N4|B7Q6N4_IXOSC Proteasome subunit alpha type, putative OS=Ixodes scapul	*Ixodes scapularis*	2	0.003
tr|B7Q2P8|B7Q2P8_IXOSC 16 kDa thioredoxion, putative OS=Ixodes scapularis GN=I	*Ixodes scapularis*	3	0.003
tr|B7Q0Q1|B7Q0Q1_IXOSC Putative uncharacterized protein (Fragment) OS=Ixodes sc	*Ixodes scapularis*	1	0.003
tr|Q4PLZ7|Q4PLZ7_IXOSC Signal peptidase, putative OS=Ixodes scapularis GN=IscW	*Ixodes scapularis*	1	0.003
tr|B7PCD2|B7PCD2_IXOSC NADP-dependent isocitrate dehydrogenase, putative OS=Ixo	*Ixodes scapularis*	1	0.003
tr|B7P585|B7P585_IXOSC Phosphoglycerate kinase OS=Ixodes scapularis GN=IscW_ISC	*Ixodes scapularis*	2	0.003
tr|B7P595|B7P595_IXOSC Proline and glutamine-rich splicing factor (SFPQ), puta	*Ixodes scapularis*	2	0.003
tr|B7PE36|B7PE36_IXOSC Nucleosome assembly protein NAP-1, putative (Fragment)	*Ixodes scapularis*	1	0.003
tr|B7QN17|B7QN17_IXOSC Thioredoxin-dependent peroxide reductase OS=Ixodes scapu	*Ixodes scapularis*	2	0.003
tr|B7Q1W5|B7Q1W5_IXOSC Elongation factor 1 gamma, putative OS=Ixodes scapulari	*Ixodes scapularis*	1	0.003
tr|A6N9Z4|A6N9Z4_ORNPR 40S ribosomal protein S3 OS=Ornithodoros parkeri PE=2 SV	*Ornithodoros parkeri*	2	0.003
tr|A8UY20|A8UY20_9ACAR Elongation factor 1-alpha (Fragment) OS=Hypochthonius l	*Hypochthonius luteus*	2	0.003
tr|B7QAW9|B7QAW9_IXOSC ATP synthase B chain, putative OS=Ixodes scapularis GN=	*Ixodes scapularis*	1	0.003
tr|B7PUS2|B7PUS2_IXOSC Ribosome recycling factor, putative OS=Ixodes scapulari	*Ixodes scapularis*	1	0.003
tr|A9QQC2|A9QQC2_LYCSI Cofilin OS=Lycosa singoriensis PE=2 SV=1	*Lycosa singoriensis*	1	0.003

tr|B7PEL3|B7PEL3_IXOSC Protein tyrosine phosphatase, putative OS=Ixodes scapula	*Ixodes scapularis*	1	0.003
tr|B7P2S4|B7P2S4_IXOSC Acetyl-CoA acetyltransferase, putative (Fragment) OS=Ix	*Ixodes scapularis*	1	0.003
tr|B7PR84|B7PR84_IXOSC Ubiquitin-activating enzyme E1, putative (Fragment) OS=	*Ixodes scapularis*	4	0.003
tr|B7QIX6|B7QIX6_IXOSC Kinesin, putative OS=Ixodes scapularis GN=IscW_ISCW01433	*Ixodes scapularis*	1	0.003
tr|B7PC82|B7PC82_IXOSC Thimet oligopeptidase, putative OS=Ixodes scapularis GN	*Ixodes scapularis*	1	0.003
tr|B7QI53|B7QI53_IXOSC Apoptosis-promoting RNA-binding protein TIA-1/TIAR, put	*Ixodes scapularis*	3	0.003
tr|B2ZWT4|B2ZWT4_HAELO Peptidyl-prolyl cis-trans isomerase OS=Haemaphysalis lo	*Haemaphysalis longicornis*	1	0.003
sp|Q09JT4|RL38_ARGMO 60S ribosomal protein L38 OS=Argas monolakensis GN=RpL38	*Argas monolakensis*	1	0.003
tr|B7QDB3|B7QDB3_IXOSC Ribosomal protein S27 OS=Ixodes scapularis GN=IscW_ISCW	*Ixodes scapularis*	1	0.003
tr|B7PS62|B7PS62_IXOSC 26S proteasome regulatory complex, subunit RPN10/PSMD4,	*Ixodes scapularis*	1	0.003
tr|B7P2W2|B7P2W2_IXOSC 60S ribosomal protein L14, putative OS=Ixodes scapularis	*Ixodes scapularis*	1	0.003
tr|B7QIP4|B7QIP4_IXOSC 4SNc-Tudor domain protein, putative OS=Ixodes scapularis	*Ixodes scapularis*	3	0.003
tr|Q4PLY7|Q4PLY7_IXOSC Nucleoside diphosphate kinase (Fragment) OS=Ixodes scapu	*Ixodes scapularis*	2	0.003
tr|B7P289|B7P289_IXOSC Prolyl 4-hydroxylase alpha subunit, putative OS=Ixodes s	*Ixodes scapularis*	1	0.003
sp|A6NA00|RSSA_ORNPR 40S ribosomal protein SA OS=Ornithodoros parkeri PE=2 SV=	*Ornithodoros parkeri*	2	0.003
tr|B7PQS1|B7PQS1_IXOSC Phenylalanyl-tRNA synthetase beta subunit, putative OS=	*Ixodes scapularis*	1	0.003
tr|B7PGC4|B7PGC4_IXOSC Uridine 5'-monophosphate synthase, putative OS=Ixodes sc	*Ixodes scapularis*	1	0.003
tr|B7PEK1|B7PEK1_IXOSC Polypyrimidine tract binding protein, putative (Fragmen	*Ixodes scapularis*	1	0.003
tr|B7Q1Y2|B7Q1Y2_IXOSC 6-phosphogluconate dehydrogenase, decarboxylating (Frag	*Ixodes scapularis*	2	0.003
tr|Q64K74|Q64K74_IXOSC Calreticulin OS=Ixodes scapularis PE=3 SV=1	*Ixodes scapularis*	1	0.003
tr|B7PA03|B7PA03_IXOSC ATP-dependent helicase (DEAD box), putative OS=Ixodes s	*Ixodes scapularis*	1	0.003
tr|B7PD56|B7PD56_IXOSC Peptidyl-prolyl cis-trans isomerase OS=Ixodes scapularis	*Ixodes scapularis*	3	0.003
tr|B7PZG8|B7PZG8_IXOSC Aldehyde dehydrogenase, putative OS=Ixodes scapularis GN	*Ixodes scapularis*	2	0.003
tr|A6N9N9|A6N9N9_ORNPR Ribosomal protein S7 OS=Ornithodoros parkeri PE=2 SV=1	*Ornithodoros parkeri*	1	0.003
tr|B7PGX4|B7PGX4_IXOSC Synaptic vesicle-associated integral membrane protein,	*Ixodes scapularis*	1	0.004
tr|B7Q331|B7Q331_IXOSC Glucose-6-phosphate 1-dehydrogenase (Fragment) OS=Ixode	*Ixodes scapularis*	1	0.004
tr|B7P5W3|B7P5W3_IXOSC Acyl-CoA synthetase, putative OS=Ixodes scapularis GN=I	*Ixodes scapularis*	1	0.004
tr|B7PQP6|B7PQP6_IXOSC Acetyl-CoA acetyltransferase, putative (Fragment) OS=Ix	*Ixodes scapularis*	1	0.004
tr|B7PTQ4|B7PTQ4_IXOSC ADP ribosylation factor 79F, putative OS=Ixodes scapular	*Ixodes scapularis*	1	0.004
tr|B7Q5H9|B7Q5H9_IXOSC Fructose-bisphosphate aldolase OS=Ixodes scapularis GN=I	*Ixodes scapularis*	2	0.004
tr|B5M728|B5M728_9ACAR Translocon-associated protein subunit alpha OS=Amblyomma	*Amblyomma americanum*	1	0.004
tr|B7PSQ6|B7PSQ6_IXOSC 40S ribosomal protein S3A, putative OS=Ixodes scapularis	*Ixodes scapularis*	1	0.004

tr|B7Q396|B7Q396_IXOSC Secreted protein, putative OS=Ixodes scapularis GN=IscW	*Ixodes scapularis*	1	0.004
tr|B7Q4L8|B7Q4L8_IXOSC Ribosomal protein, putative (Fragment) OS=Ixodes scapul	*Ixodes scapularis*	1	0.004
tr|B7QCB3|B7QCB3_IXOSC Cytochrome B5, putative OS=Ixodes scapularis GN=IscW_IS	*Ixodes scapularis*	1	0.004
tr|B7Q2P2|B7Q2P2_IXOSC Zinc finger protein, putative (Fragment) OS=Ixodes scapu	*Ixodes scapularis*	1	0.004
tr|B7P2T4|B7P2T4_IXOSC Ribosomal protein S17, putative OS=Ixodes scapularis GN	*Ixodes scapularis*	1	0.004
tr|B7Q9E5|B7Q9E5_IXOSC Alpha-2-macroglobulin receptor-associated protein, puta	*Ixodes scapularis*	1	0.004
tr|B7P5B3|B7P5B3_IXOSC U5 snRNP-specific protein, putative (Fragment) OS=Ixode	*Ixodes scapularis*	1	0.004
tr|B7PJP4|B7PJP4_IXOSC Dolichyl-di-phosphooligosaccharide protein glycotransfe	*Ixodes scapularis*	1	0.004
tr|B7P7F7|B7P7F7_IXOSC Heat shock protein, putative OS=Ixodes scapularis GN=Isc	*Ixodes scapularis*	1	0.004
tr|B7Q0B3|B7Q0B3_IXOSC Heat shock protein 70 (HSP70)-interacting protein, puta	*Ixodes scapularis*	1	0.004
tr|B7PNL5|B7PNL5_IXOSC Syntenin, putative OS=Ixodes scapularis GN=IscW_ISCW0057	*Ixodes scapularis*	1	0.004
tr|B7P5Y0|B7P5Y0_IXOSC Seryl-tRNA synthetase, putative OS=Ixodes scapularis GN	*Ixodes scapularis*	1	0.004
tr|B7PXR5|B7PXR5_IXOSC Chaperonin complex component, TCP-1 eta subunit, putativ	*Ixodes scapularis*	1	0.004
tr|B7PPR5|B7PPR5_IXOSC Putative uncharacterized protein (Fragment) OS=Ixodes sc	*Ixodes scapularis*	1	0.004
tr|B7PCN1|B7PCN1_IXOSC Aldo-keto reductase, putative OS=Ixodes scapularis GN=Is	*Ixodes scapularis*	2	0.004
tr|B6V3B5|B6V3B5_IXORI Glutathione peroxidase OS=Ixodes ricinus PE=2 SV=1	*Ixodes ricinus*	2	0.004
tr|B7QNR8|B7QNR8_IXOSC Importin beta, nuclear transport factor, putative OS=Ix	*Ixodes scapularis*	2	0.004
tr|B7P573|B7P573_IXOSC Processing peptidase beta subunit, putative OS=Ixodes sc	*Ixodes scapularis*	3	0.006
tr|B7P7M2|B7P7M2_IXOSC Signal recognition particle protein, putative OS=Ixodes	*Ixodes scapularis*	2	0.006
tr|B7PNN7|B7PNN7_IXOSC Attractin and platelet-activating factor acetylhydrolase	*Ixodes scapularis*	1	0.006
tr|B7Q4F2|B7Q4F2_IXOSC Cop9 complex subunit 7A, putative OS=Ixodes scapularis	*Ixodes scapularis*	1	0.008
tr|B7PAS1|B7PAS1_IXOSC MCM2 protein, putative (Fragment) OS=Ixodes scapularis	*Ixodes scapularis*	1	0.008
tr|B7PCK4|B7PCK4_IXOSC Splicing factor u2af large subunit, putative OS=Ixodes	*Ixodes scapularis*	1	0.008
tr|B7QEF1|B7QEF1_IXOSC VAMP-associated protein involved in inositol metabolism	*Ixodes scapularis*	1	0.008
tr|B7Q5L0|B7Q5L0_IXOSC ATP synthase OS=Ixodes scapularis GN=IscW_ISCW021200 PE	*Ixodes scapularis*	1	0.008
tr|B7PQA7|B7PQA7_IXOSC Secreted protein, putative OS=Ixodes scapularis GN=IscW	*Ixodes scapularis*	1	0.008
tr|A8UYT9|A8UYT9_9ACAR Elongation factor 1-alpha (Fragment) OS=Schoutedenocopt	*Schoutedenocoptes aquilae*	1	0.008
tr|A8UY35|A8UY35_9ACAR Elongation factor 1-alpha (Fragment) OS=Hormosianoetus m	*Hormosianoetus mallotae*	1	0.008
tr|B7PN29|B7PN29_IXOSC Steroid membrane receptor Hpr6.6/25-Dx, putative OS=Ixo	*Ixodes scapularis*	2	0.008
tr|B7QAM9|B7QAM9_IXOSC Putative uncharacterized protein (Fragment) OS=Ixodes s	*Ixodes scapularis*	1	0.008
tr|B7PQ08|B7PQ08_IXOSC U1 small nuclear ribonucleoprotein A, putative OS=Ixode	*Ixodes scapularis*	1	0.008
tr|A9QQ29|A9QQ29_LYCSI Translation elongation factor 2 (Fragment) OS=Lycosa sin	*Lycosa singoriensis*	1	0.008

tr|B7PYP5|B7PYP5_IXOSC Heat shock protein, putative OS=Ixodes scapularis GN=Is	*Ixodes scapularis*	1	0.008
tr|B7Q3T6|B7Q3T6_IXOSC THO complex subunit, putative OS=Ixodes scapularis GN=Is	*Ixodes scapularis*	1	0.009
tr|B7QC21|B7QC21_IXOSC Annexin V, putative (Fragment) OS=Ixodes scapularis GN=	*Ixodes scapularis*	2	0.009
tr|B7PSB7|B7PSB7_IXOSC Activator of 90 kDa heat shock protein ATPase, putative	*Ixodes scapularis*	1	0.010
tr|B7QLI8|B7QLI8_IXOSC Tyrosine aminotransferase, putative (Fragment) OS=Ixodes	*Ixodes scapularis*	1	0.010
tr|Q4PM83|Q4PM83_IXOSC Ribosomal protein L27A, putative OS=Ixodes scapularis G	*Ixodes scapularis*	1	0.010
tr|B7PV22|B7PV22_IXOSC Poly [ADP-ribose] polymerase, putative OS=Ixodes scapul	*Ixodes scapularis*	1	0.010
tr|B7PHM9|B7PHM9_IXOSC Isocitrate dehydrogenase, putative (Fragment) OS=Ixodes	*Ixodes scapularis*	1	0.010
tr|A9QQ53|A9QQ53_LYCSI 60S ribosomal protein L13 (Fragment) OS=Lycosa singorie	*Lycosa singoriensis*	1	0.010
**Proteins identified with 1% < FDR < 5%**			
tr|B7QCA7|B7QCA7_IXOSC Glucosidase II, putative (Fragment) OS=Ixodes scapulari	*Ixodes scapularis*	1	0.010
tr|B7PR58|B7PR58_IXOSC GTP binding protein Rab-1A OS=Ixodes scapularis GN=IscW	*Ixodes scapularis*	2	0.010
tr|B7QCB8|B7QCB8_IXOSC 26S proteasome regulatory subunit 7, psd7, putative (Fr	*Ixodes scapularis*	1	0.010
tr|A9QQ67|A9QQ67_LYCSI 40S ribosomal protein S3a OS=Lycosa singoriensis PE=2 S	*Lycosa singoriensis*	2	0.010
tr|B7Q760|B7Q760_IXOSC Nucleotide excision repair factor NEF2, RAD23 component	*Ixodes scapularis*	2	0.010
tr|B7PRH5|B7PRH5_IXOSC T-complex protein 1, delta subunit OS=Ixodes scapularis	*Ixodes scapularis*	1	0.010
tr|B7P971|B7P971_IXOSC Calponin, putative OS=Ixodes scapularis GN=IscW_ISCW0030	*Ixodes scapularis*	1	0.010
tr|B7PTK1|B7PTK1_IXOSC Multiple ankyrin repeats single kh domain protein, puta	*Ixodes scapularis*	1	0.010
tr|B7PIP9|B7PIP9_IXOSC Ankyrin 2,3/unc44, putative OS=Ixodes scapularis GN=IscW	*Ixodes scapularis*	2	0.010
tr|B7PKZ9|B7PKZ9_IXOSC BRI1-KD interacting protein, putative OS=Ixodes scapula	*Ixodes scapularis*	1	0.010
tr|B7Q362|B7Q362_IXOSC Eukaryotic translation initiation factor 4 gamma, putat	*Ixodes scapularis*	1	0.011
tr|B7P6L7|B7P6L7_IXOSC 26S proteasome regulatory complex, subunit PSMD5, putat	*Ixodes scapularis*	1	0.011
tr|B7PU84|B7PU84_IXOSC Putative uncharacterized protein (Fragment) OS=Ixodes s	*Ixodes scapularis*	1	0.011
tr|B7PD93|B7PD93_IXOSC Ran-binding protein, putative OS=Ixodes scapularis GN=I	*Ixodes scapularis*	1	0.011
tr|B7QMM9|B7QMM9_IXOSC Polyadenylate-binding protein-interacting protein, puta	*Ixodes scapularis*	1	0.011
tr|B7P9A9|B7P9A9_IXOSC HyFMR1 protein, putative (Fragment) OS=Ixodes scapulari	*Ixodes scapularis*	1	0.011
tr|B7PAK1|B7PAK1_IXOSC Integrin beta (Fragment) OS=Ixodes scapularis GN=IscW_I	*Ixodes scapularis*	1	0.011
tr|B7PAI0|B7PAI0_IXOSC Ribosomal protein L28, putative OS=Ixodes scapularis GN=	*Ixodes scapularis*	1	0.011
tr|B7P230|B7P230_IXOSC Translation initiation factor 2C, putative OS=Ixodes sc	*Ixodes scapularis*	1	0.011
tr|A8E4J9|A8E4J9_9ACAR Calreticulin OS=Haemaphysalis qinghaiensis PE=2 SV=1	*Haemaphysalis qinghaiensis*	1	0.011
tr|B7PM02|B7PM02_IXOSC Proteasome beta2 subunit, putative OS=Ixodes scapularis	*Ixodes scapularis*	1	0.012
tr|A6N9M1|A6N9M1_ORNPR 40S ribosomal protein S2/30S OS=Ornithodoros parkeri PE=	*Ornithodoros parkeri*	2	0.013

tr|Q4PM69|Q4PM69_IXOSC Histone H4 OS=Ixodes scapularis GN=IscW_ISCW019498 PE=3	*Ixodes scapularis*	2	0.013
tr|B7Q3Z3|B7Q3Z3_IXOSC 26S proteasome regulatory subunit rpn1, putative OS=Ixo	*Ixodes scapularis*	2	0.014
tr|B7Q7H2|B7Q7H2_IXOSC Kinesin light chain, putative OS=Ixodes scapularis GN=I	*Ixodes scapularis*	1	0.014
tr|B7PXJ6|B7PXJ6_IXOSC Glyoxylate/hydroxypyruvate reductase, putative (Fragmen	*Ixodes scapularis*	2	0.014
tr|B7PUB0|B7PUB0_IXOSC Secreted protein, putative OS=Ixodes scapularis GN=IscW	*Ixodes scapularis*	1	0.016
tr|B7PJZ9|B7PJZ9_IXOSC Dynein light chain OS=Ixodes scapularis GN=IscW_ISCW003	*Ixodes scapularis*	1	0.017
tr|B7P377|B7P377_IXOSC Lim and sh3 domain protein 1, lasp-1, putative OS=Ixode	*Ixodes scapularis*	1	0.017
tr|B7Q310|B7Q310_IXOSC Putative uncharacterized protein OS=Ixodes scapularis G	*Ixodes scapularis*	1	0.017
tr|B7P8J4|B7P8J4_IXOSC ATP-dependent RNA helicase, putative (Fragment) OS=Ixode	*Ixodes scapularis*	1	0.017
tr|B7PAG0|B7PAG0_IXOSC THO complex subunit, putative OS=Ixodes scapularis GN=I	*Ixodes scapularis*	1	0.017
tr|B7PXG9|B7PXG9_IXOSC Glutathione S-transferase, putative OS=Ixodes scapulari	*Ixodes scapularis*	1	0.017
tr|B7PKQ6|B7PKQ6_IXOSC Cell division protein, putative (Fragment) OS=Ixodes sca	*Ixodes scapularis*	1	0.017
tr|B7PVP6|B7PVP6_IXOSC Rho/RAC guanine nucleotide exchange factor, putative OS=	*Ixodes scapularis*	1	0.017
tr|B0LUH3|B0LUH3_IXORI Thioredoxin peroxidase OS=Ixodes ricinus PE=2 SV=1	*Ixodes ricinus*	1	0.017
tr|B2YGD3|B2YGD3_9ARAC Actin (Fragment) OS=Galianora bryicola PE=4 SV=1	*Galianora bryicola*	1	0.017
tr|B7QFT9|B7QFT9_IXOSC Lectin, putative OS=Ixodes scapularis GN=IscW_ISCW01262	*Ixodes scapularis*	1	0.017
tr|B7PR90|B7PR90_IXOSC Ribosomal protein L13A, putative OS=Ixodes scapularis GN	*Ixodes scapularis*	1	0.017
tr|B7QL56|B7QL56_IXOSC DNA replication licensing factor, putative (Fragment) O	*Ixodes scapularis*	1	0.017
tr|B7Q4R6|B7Q4R6_IXOSC Ku P70 DNA helicase, putative (Fragment) OS=Ixodes scap	*Ixodes scapularis*	1	0.017
tr|B7PKK7|B7PKK7_IXOSC Ubiquitin carrier protein OS=Ixodes scapularis GN=IscW_	*Ixodes scapularis*	1	0.018
tr|B7PCH9|B7PCH9_IXOSC Histidine triad nucleotide binding protein, putative (F	*Ixodes scapularis*	1	0.018
tr|B7PT80|B7PT80_IXOSC Spindle pole body protein, putative OS=Ixodes scapulari	*Ixodes scapularis*	1	0.019
tr|B7QF74|B7QF74_IXOSC Microsomal glutathione S-transferase, putative OS=Ixode	*Ixodes scapularis*	1	0.019
tr|B7QMF1|B7QMF1_IXOSC Reductase, putative (Fragment) OS=Ixodes scapularis GN=I	*Ixodes scapularis*	1	0.019
tr|B7P555|B7P555_IXOSC Coronin, putative (Fragment) OS=Ixodes scapularis GN=Is	*Ixodes scapularis*	1	0.019
tr|B7P1Y7|B7P1Y7_IXOSC Transcription factor S-II, putative OS=Ixodes scapulari	*Ixodes scapularis*	1	0.019
tr|B7QF45|B7QF45_IXOSC 3-keto-acyl-CoA thiolase, putative OS=Ixodes scapularis	*Ixodes scapularis*	1	0.019
tr|B7PGP8|B7PGP8_IXOSC Replication factor C, subunit RFC3, putative OS=Ixodes s	*Ixodes scapularis*	1	0.019
tr|B7PWX1|B7PWX1_IXOSC Phospholipase A-2-activating protein, putative (Fragment	*Ixodes scapularis*	1	0.020
tr|B7Q0N5|B7Q0N5_IXOSC Heat shock protein 70 (HSP70)-interacting protein, puta	*Ixodes scapularis*	1	0.020
tr|B7Q0E8|B7Q0E8_IXOSC Serpin 7, putative OS=Ixodes scapularis GN=IscW_ISCW009	*Ixodes scapularis*	1	0.020
tr|B7QDS2|B7QDS2_IXOSC Matricellular protein osteonectin/SPARC/BM-40, putative	*Ixodes scapularis*	2	0.021

tr|B7P367|B7P367_IXOSC Putative uncharacterized protein OS=Ixodes scapularis GN	*Ixodes scapularis*	1	0.021
tr|B7P3D3|B7P3D3_IXOSC FKBP-type peptidyl-prolyl cis-trans isomerase, putative	*Ixodes scapularis*	1	0.021
tr|B7QD48|B7QD48_IXOSC Putative uncharacterized protein (Fragment) OS=Ixodes sc	*Ixodes scapularis*	1	0.021
tr|B7PIZ2|B7PIZ2_IXOSC ADP-ribosylation factor, putative (Fragment) OS=Ixodes	*Ixodes scapularis*	1	0.022
tr|B7PCU5|B7PCU5_IXOSC 2-oxoglutarate dehydrogenase, putative OS=Ixodes scapul	*Ixodes scapularis*	1	0.023
tr|A6N9M5|A6N9M5_ORNPR 40S ribosomal protein S20 OS=Ornithodoros parkeri PE=2	*Ornithodoros parkeri*	1	0.023
tr|B7PVQ8|B7PVQ8_IXOSC Putative uncharacterized protein OS=Ixodes scapularis G	*Ixodes scapularis*	1	0.023
tr|B7Q347|B7Q347_IXOSC Putative uncharacterized protein OS=Ixodes scapularis G	*Ixodes scapularis*	1	0.024
tr|B7PSJ2|B7PSJ2_IXOSC Proteasome subunit alpha type OS=Ixodes scapularis GN=I	*Ixodes scapularis*	1	0.024
tr|A5HLD6|A5HLD6_9ARAC Heat shock protein 70kDa (Fragment) OS=Diguetia mojavea	*Diguetia mojavea*	1	0.024
tr|B5M794|B5M794_9ACAR Damaged-DNA binding protein DDB p127 subunit (Fragment)	*Amblyomma americanum*	1	0.025
tr|B7PRG2|B7PRG2_IXOSC 60S acidic ribosomal protein P0, putative OS=Ixodes sca	*Ixodes scapularis*	1	0.025
tr|B5M799|B5M799_9ACAR Histone H2B OS=Amblyomma americanum PE=2 SV=1	*Amblyomma americanum*	1	0.025
tr|B7PFQ0|B7PFQ0_IXOSC 60S ribosomal protein L27, putative OS=Ixodes scapulari	*Ixodes scapularis*	1	0.025
tr|B7PHX7|B7PHX7_IXOSC Adenylate kinase, putative OS=Ixodes scapularis GN=IscW	*Ixodes scapularis*	1	0.026
tr|A9P773|A9P773_BOOMI Glycogen synthase kinase OS=Boophilus microplus GN=GSK-3	*Boophilus microplus*	1	0.026
tr|B7PU34|B7PU34_IXOSC P2X purinoceptor,putative OS=Ixodes scapularis GN=IscW_	*Ixodes scapularis*	1	0.026
tr|B7PVV2|B7PVV2_IXOSC Procollagen-lysine, 2-oxoglutarate 5-dioxygenase, putat	*Ixodes scapularis*	1	0.026
sp|Q4PMB3|RS4_IXOSC 40S ribosomal protein S4 OS=Ixodes scapularis GN=RpS4 PE=2	*Ixodes scapularis*	1	0.027
tr|B7PIV8|B7PIV8_IXOSC Putative uncharacterized protein (Fragment) OS=Ixodes s	*Ixodes scapularis*	1	0.027
tr|B7PZ79|B7PZ79_IXOSC Proteasome subunit alpha type, putative OS=Ixodes scapu	*Ixodes scapularis*	1	0.027
tr|B7Q650|B7Q650_IXOSC Reductase, putative (Fragment) OS=Ixodes scapularis GN=	*Ixodes scapularis*	1	0.027
tr|B7QCU0|B7QCU0_IXOSC CDK inhibitor P21 binding protein, putative OS=Ixodes s	*Ixodes scapularis*	1	0.031
tr|B7QLS5|B7QLS5_IXOSC Numb-associated kinase, putative OS=Ixodes scapularis G	*Ixodes scapularis*	1	0.033
tr|B7P9Y8|B7P9Y8_IXOSC Protocadherin-16, putative OS=Ixodes scapularis GN=IscW	*Ixodes scapularis*	1	0.033
tr|B7Q2Z7|B7Q2Z7_IXOSC Ribosomal protein S28, putative (Fragment) OS=Ixodes sc	*Ixodes scapularis*	1	0.035
tr|B7PAM5|B7PAM5_IXOSC Peptidyl-prolyl cis-trans isomerase, putative OS=Ixodes	*Ixodes scapularis*	1	0.036
tr|B7PKB9|B7PKB9_IXOSC Lumican, putative (Fragment) OS=Ixodes scapularis GN=Is	*Ixodes scapularis*	1	0.036
tr|B7P9W6|B7P9W6_IXOSC 26S proteasome regulatory complex, subunit RPN5/PSMD12,	*Ixodes scapularis*	1	0.040
tr|B7QF31|B7QF31_IXOSC Caspase, apoptotic cysteine protease, putative (Fragment	*Ixodes scapularis*	1	0.041
tr|Q4PMB6|Q4PMB6_IXOSC 60S ribosomal protein L7a OS=Ixodes scapularis PE=2 SV=	*Ixodes scapularis*	1	0.042
tr|B7Q1G1|B7Q1G1_IXOSC Methylmalonyl coenzyme A mutase, putative OS=Ixodes sca	*Ixodes scapularis*	1	0.045
tr|B7QL45|B7QL45_IXOSC La/SS-B, putative (Fragment) OS=Ixodes scapularis GN=Is	*Ixodes scapularis*	2	0.046

tr|B7Q0T6|B7Q0T6_IXOSC Acetylcholinesterase, putative OS=Ixodes scapularis GN=	*Ixodes scapularis*	1	0.047
tr|B7PXM3|B7PXM3_IXOSC Putative uncharacterized protein OS=Ixodes scapularis G	*Ixodes scapularis*	1	0.047
tr|B7PXA7|B7PXA7_IXOSC Golgi reassembly stacking protein, putative OS=Ixodes s	*Ixodes scapularis*	1	0.048
tr|B7QIF6|B7QIF6_IXOSC Golgi protein, putative (Fragment) OS=Ixodes scapularis	*Ixodes scapularis*	1	0.049
**Proteins identified with 5% < FDR < 10%**			
tr|B7QDQ3|B7QDQ3_IXOSC Molecular chaperone, putative OS=Ixodes scapularis GN=I	*Ixodes scapularis*	1	0.050
tr|B7QAM6|B7QAM6_IXOSC Protein disulfide isomerase, putative (Fragment) OS=Ixo	*Ixodes scapularis*	1	0.051
tr|B7QJ34|B7QJ34_IXOSC OTU domain, ubiquitin aldehyde binding protein, putativ	*Ixodes scapularis*	1	0.051
tr|B7Q1W9|B7Q1W9_IXOSC Dihydrolipoamide acetyltransferase, putative (Fragment)	*Ixodes scapularis*	1	0.052
tr|B7P2P0|B7P2P0_IXOSC Membrane protein, putative OS=Ixodes scapularis GN=IscW	*Ixodes scapularis*	1	0.052
tr|B7Q792|B7Q792_IXOSC Putative uncharacterized protein (Fragment) OS=Ixodes s	*Ixodes scapularis*	1	0.054
tr|B7PV46|B7PV46_IXOSC Putative uncharacterized protein OS=Ixodes scapularis G	*Ixodes scapularis*	1	0.054
tr|Q6W976|Q6W976_9ARAC Sodium/potassium ATPase alpha subunit (Fragment) OS=Opi	*Opiliones sp.*	1	0.054
tr|B7PSW5|B7PSW5_IXOSC Programmed cell death 6-interacting protein, putative O	*Ixodes scapularis*	1	0.056
tr|B7Q6Y7|B7Q6Y7_IXOSC RNA-binding protein musashi, putative OS=Ixodes scapula	*Ixodes scapularis*	1	0.056
tr|B7PGQ2|B7PGQ2_IXOSC Calnexin, putative OS=Ixodes scapularis GN=IscW_ISCW003	*Ixodes scapularis*	1	0.056
tr|B7P924|B7P924_IXOSC Rap1 GTPase-GDP dissociation stimulator, putative OS=Ix	*Ixodes scapularis*	1	0.059
tr|B7Q5F9|B7Q5F9_IXOSC Glyoxalase, putative OS=Ixodes scapularis GN=IscW_ISCW0	*Ixodes scapularis*	1	0.059
tr|B7PAD5|B7PAD5_IXOSC Microtubule-binding protein, putative (Fragment) OS=Ixo	*Ixodes scapularis*	1	0.059
tr|B7PYD1|B7PYD1_IXOSC ATP-dependent RNA helicase, putative (Fragment) OS=Ixod	*Ixodes scapularis*	1	0.059
tr|B7Q3D3|B7Q3D3_IXOSC ATP-citrate synthase, putative OS=Ixodes scapularis GN=I	*Ixodes scapularis*	1	0.059
tr|B7P163|B7P163_IXOSC Eukaryotic translation initiation factor 3 subunit C, p	*Ixodes scapularis*	1	0.060
tr|B7PXW1|B7PXW1_IXOSC Ribosomal protein S25, putative (Fragment) OS=Ixodes sc	*Ixodes scapularis*	1	0.063
tr|B7PT39|B7PT39_IXOSC Putative uncharacterized protein (Fragment) OS=Ixodes s	*Ixodes scapularis*	1	0.063
tr|B7PYR8|B7PYR8_IXOSC Putative uncharacterized protein OS=Ixodes scapularis G	*Ixodes scapularis*	1	0.063
tr|B7QMC8|B7QMC8_IXOSC Alpha-macroglobulin, putative (Fragment) OS=Ixodes scap	*Ixodes scapularis*	1	0.063
tr|B5TMF7|B5TMF7_DERVA Glyceraldehyde 3-phosphate dehydrogenase OS=Dermacentor	*Dermacentor variabilis*	1	0.063
tr|B7QLA4|B7QLA4_IXOSC Phosphatidylethanolamine-binding protein, putative OS=I	*Ixodes scapularis*	1	0.066
tr|Q4PLY0|Q4PLY0_IXOSC F1F0-type ATP synthase subunit g OS=Ixodes scapularis P	*Ixodes scapularis*	1	0.068
tr|B7PMA8|B7PMA8_IXOSC Putative uncharacterized protein OS=Ixodes scapularis G	*Ixodes scapularis*	1	0.080
tr|B7PF38|B7PF38_IXOSC (S)-2-hydroxy-acid oxidase, putative OS=Ixodes scapular	*Ixodes scapularis*	1	0.080
tr|B7PQ21|B7PQ21_IXOSC DEAD box ATP-dependent RNA helicase, putative (Fragment	*Ixodes scapularis*	1	0.081

tr|B7QIE9|B7QIE9_IXOSC Nudix hydrolase, putative OS=Ixodes scapularis GN=IscW_	*Ixodes scapularis*	1	0.082
tr|B7P4E1|B7P4E1_IXOSC Glutamate dehydrogenase, putative OS=Ixodes scapularis	*Ixodes scapularis*	1	0.082
tr|B7QH59|B7QH59_IXOSC Nuclear distribution protein NUDC, putative (Fragment)	*Ixodes scapularis*	2	0.083
tr|B7PLL8|B7PLL8_IXOSC Estradiol 17-beta-dehydrogenase, putative OS=Ixodes sca	*Ixodes scapularis*	1	0.084
tr|B7PPR8|B7PPR8_IXOSC FK506 binding protein (FKBP), putative OS=Ixodes scapul	*Ixodes scapularis*	1	0.084
tr|B7PKP9|B7PKP9_IXOSC Glyceraldehyde 3-phosphate dehydrogenase OS=Ixodes scap	*Ixodes scapularis*	1	0.087
tr|Q26229|Q26229_RHIAP Autoantigen OS=Rhipicephalus appendiculatus PE=2 SV=1	*Rhipicephalus appendiculatus*	1	0.088
tr|B7PL00|B7PL00_IXOSC Antiviral helicase Slh1, putative OS=Ixodes scapularis G	*Ixodes scapularis*	1	0.088
tr|B7PYA7|B7PYA7_IXOSC Putative uncharacterized protein OS=Ixodes scapularis G	*Ixodes scapularis*	1	0.090
tr|B7PZS8|B7PZS8_IXOSC Protein transport protein sec23, putative OS=Ixodes sca	*Ixodes scapularis*	1	0.090
tr|B7PKV8|B7PKV8_IXOSC RNA-binding protein, putative OS=Ixodes scapularis GN=I	*Ixodes scapularis*	1	0.095
tr|B7PNU5|B7PNU5_IXOSC Glutamine synthetase, putative OS=Ixodes scapularis GN=	*Ixodes scapularis*	1	0.095
tr|B7P7A4|B7P7A4_IXOSC Importin, putative OS=Ixodes scapularis GN=IscW_ISCW016	*Ixodes scapularis*	1	0.097
tr|Q17248|Q17248_BOOMI Angiotensin-converting enzyme-like protein OS=Boophilus	*Boophilus microplus*	1	0.098

[a] Number of peptides by which proteins were identified.
[b]False discovery rate (FDR) is used as a measure of statistical significance of peptide identification and is calculated using the refined method proposed by[174].

Supplementary Table 36. Summary of F statistics for filtered RAD loci. Heterozygosity within a subpopulation of *I. scapularis* collected from different geographic regions and the Wikel laboratory colony.

Sample	n	T	% pol	SNP	Private	P	H_o	H_E	F_{IS}	π
**Mid West**										
Wisconsin	12	3,365,898	7.08	589,587	51,068	0.989	0.013	0.016	0.012	0.017
Indiana	10	3,368,281	4.76	581,843	22,650	0.990	0.013	0.014	0.006	0.015
**North East**										
Maine	10	3,654,874	6.31	622,432	35,573	0.989	0.013	0.016	0.011	0.017
New Hampshire	10	3,433,477	6.40	594,562	34,352	0.989	0.013	0.016	0.011	0.017
Massachusetts	7	3,362,822	5.52	584,514	26,532	0.989	0.014	0.015	0.007	0.017
**South East**										
Virginia	10	3,180,531	5.22	555,145	44,662	0.989	0.016	0.016	0.005	0.020
North Carolina	5	3,709,763	5.56	636,280	66,486	0.988	0.015	0.017	0.011	0.019
Florida	5	2,741,357	7.48	500,178	80,789	0.988	0.016	0.018	0.010	0.019
**Reference**										
Wikel	5	3,786,899	3.80	628,131	22,051	0.990	0.014	0.013	0.003	0.015

n - number of analyzed individuals from each population; T - the number of RAD loci; % pol - percentage of polymorphic loci; SNP - total number of SNPs; private - the number of private SNPs; P - average frequency of the more common allele; H_O, H_E – observed and expected heterozygosity at polymorphic sites; F_{IS} - fixation index across polymorphic sites; π – average nucleotide diversity (calculated across polymorphic and non-variant sites)

Supplementary Table 37. Genetic variation among populations of *I. scapularis* collected from different geographic regions of the U.S. and the Wikel laboratory colony. F_{ST} values are shown as a measure of differentiation. F_{ST} = <0.05, low genetic variation (light tan shading); F_{ST} = 0.05-0.15, moderate genetic variation (tan shading); F_{ST} = 0.15-0.25, high genetic variation (orange shading); F_{ST} = >0.25, very high genetic variation[178].

Location	Sample	IN	ME	NH	MA	VA	NC	FL	Wikel
Mid West	WI	0.045	0.037	0.040	0.042	0.100	0.119	0.102	0.072
	IN		0.055	0.057	0.064	0.132	0.153	0.124	0.106
North East	ME			0.038	0.043	0.106	0.127	0.106	0.078
	NH				0.046	0.105	0.125	0.104	0.079
	MA					0.119	0.139	0.113	0.092
South East	VA						0.091	0.079	0.142
	NC							0.072	0.161
	FL								0.123

Abbreviations: Indiana, IN; Maine, ME; New Hampshire, NH; Massachusets, MA; Virginia, VA; North Carolina, NC; Florida, FL; Wisconsin, WI.

Supplementary Table 38. Proposed tick and mite genomes, clinical significance and sequencing priority.

Acari Classification	Species/Geographic Region	Diseases Transmitted[a]	Sequencing Priority
**Superorder Acariformes**	*Leptotrombidium deliense* Asia	Scrub typhus	**Tier 1**
**Superorder Parasitiformes**	*Ixodes scapularis* Nth. America	LD, HGA, babesiosis, POW	
Family Ixodidae (hard ticks)	*Ixodes pacificus* Nth. America	LD, HGA	**Tier 2**
Lineage Prostriata	*Ixodes ricinus* Africa/Eurasia	LD, TBE, babesiosis, HGA	
	Ixodes persulcatus Eurasia	LD, TBE	
	Dermacentor variabilis Nth. & Central America	RMSF, tularemia, anaplasmosis, tick-induced paralysis	
Lineage Metastriata	*Amblyomma americanum* Nth., Central & Sth. America	HME, STARI, tularemia	
Family Argasidae (soft ticks)	*Ornithodorus turicata* Nth. America	TBRF	**Tier 3**

[a]From[186,187]; human babesiosis (*Babesia microti*); HGA, human granulocytic anaplasmosis (*Anaplasma phagocytophilum*); HME, human monocytic erhlichiosis (*E. chaffeensis*); LD, Lyme disease (*Borrelia burgdorferi)*; POW, Powassan virus; RMSF, Rocky Mountain spotted fever (*Rickettsia rickettsii*); scrub typhus (*Orientia tsutsugamushi*), STARI, southern tick-associated rash illness (*Borrelia lonestari)*; TBRF*,* tick-borne relapsing fever (*Borrelia turicatae.*); ND, not determined.

Supplementary Note 1

Predicted Protein Sequence of *Ixodes scapularis* Gustatory Receptors (GRs)

```
>IsGr1FIX
MLRGFQLQSKFCRVSGCLFLPGLLTNPLETVSVTWKSWYSFYSALCFLFFVGYESNLITRYVLKIDGSDHLFSQSLI
VLMHVVVVLKSVVNYISMITGSRSILDFLRESALFEEAIDFPSCKCCIPKEYFRADVKRILLFVVFFLVYCVGTHFQ
LNDVFGSEKPWSAQYVMYRVCGMLTGILFFTYDSLHFVSVKVCSKVLGEYIKTQLKVIETCVSHSPGGSLEQAAKDV
EAVRMRLCIIRNLKTTLNDVWNRSIVTSCACQILVLCIAIFTVCTGGLARQDLWMALAYSLYTVYETVDLANVSQSM
ANNVQNVKEACKRAATFDGPEFFIQQIQYLHNSINPQDFTVVGGDFFSIDMPLLVSITGSVITYSVILVQTSQEFDT
NTNVDGANGTRPGSVPGS

>IsGr2FIX
MLRSFQLQARFCRVCGCLFLPGLLTNPLDTVKVTWQSWYTFYSAACFIFFVWYEFNLITRYVLMIDGSDHLFTQSLH
VLMHIVVVLKSLVNYVSMISGSRSILDFFREAESFEGTIDIPSCKCCVFKTFMWADVRRMLLFVAYLAIYLAGTHFQ
LIDVLGGQELGSEQYVLYRVGAVFAGILFFTYDSLHFVSLKVCSLVLEEYVKTQCKVIEVCVSLRPTGSMDQTAKEV
ETVRVRLCVIGNLKTTLNDVWNRSIVTSCACQILVVCIAIFTICTGGLARQELWLALIYSLYTVYETVDLASVSQSL
SNSMKKIKNACKGAPTFEGTEAYNKQIQHLHNSINPQDINVVGGDLFRIDMPLLVSITGSVITYSVILVQTSQEFDT
NTNVEGANGTRPGY

>IsGr3FIX
MLQRCVPFAIACRLFGCFFIHNFPGKSLDQAKVSWKSLYTLYSFTCFIAYLVSEIAYVIRYVDELGKISRSFSRSLL
LLVHVVITARIATNVAAMLMGPEKLLAFFRQSESFEKAIDFTTRQRSLRTSAFERWAALRAFLSLSGMAFCYAAGVN
FLMGQLEESLGSRWVIPTRIVGFFMITAVLLYDSLLYLFLRSSAKVFGEYMHTLLGAFKKCKRYRSIRSRSGVSCHI
EFIRSNMNEVKRLKEALSDIWTWPLMVASASLVIMNSFVFSAVIQDGLKKELWWAVTYSLYSTLSFIDLAYVSQALV
NEARKLKDAILVVPTYDATDDFSQQLRYLHETIDPDGMCFGGGGFFALKNSLLVSMTGAILVYTVILVQTSDTMDHK
MDAT

>IsGr4CTE
MISFMHQRCVPYAILGRLYGCFFVHNFWRKSLGDAHVTWKSLYTVYSFGFFVIYLLGEIMFATSFARDVKDVSDAFS
RHLLILVHGVVTTRVLANSVAMLTKPNKLLAFFRKSEAFEKDTAFSLRTYSLCSSVAHRWNAMRAFAAFLGLTLSYS
VAIQFLAMEHGEQILSQMAVPVKLVGFIMTTGFFVYDSMLYLFLRSCINVLVEYTQFQLVVFREQNLLFRPGEPSKI
EAMRLSLNKMRKLKELLNDIWAGPLIVACASTVITDCVILDAMFYDGMKQELWIIAAYALSASLSFIDLACTGQTLI
DEARKLKSAMLMVRAYGEPDRYLKQLRYLYEGFDPEGMCLDGGGFFVLRKSLLLP

>IsGr5INT
MISLMQRQFLPYALLCRLGGCFFIPRFWKPLEDAKVTWRSLYTAYSAFAVASWFSVELTFIVKRCHIYSNLSYHDFP
SLVLLILRATVSLKALLNFVTMATGSSGLVKFFRKASVFEKTTGFLPSSRCPKGVWKDRWSFLRRFFVVQGIVSSYV
FSTLLSSVSLTADLPADFGFLGKLGAVLTGMYYLLYDVFPYIVLSSCSSVLVAYLQAQVKMFERCCRFEAVHNNMQL
SQQLEVIRHNLGGIRDLKHSLNAIWEAPLVAMSVGVLLDVCVVFYAIFHDGFFRSHVRLAMSYCLYSSFAFMDMACI
SQALTDEAQKLKDATKAAYTFAATNGYVQQMAGTMITYTVILSQTSDGLANKAVPRN

>IsGr6CTE
MSSYMQRQFVPYAILCRLGGCFFIQNFWKPLENAKVTWKSLYTAYSVFFIALNFSLDIVLIVQESYVFRDLSQAFSP
SLILVLRMVVTSKILLSAGTMATGSLGLLEFFKKSSLYEKITGFSPARRDFRAFWKHRWSLFRRILVLIGFICTYII
SMLPFMYSLGELLPASFSFLGQISAVLGAWCYLLYDALPYMVLRSCSAVLVEYLHVQLKTVQRCCKVKPSRNERKSL
```

EQLEVIRHNMAKITDLKDCLNAIAQVPLATMSAGVLIFDCVVCYAMFNDGFFATDVPLALSYCVYSSFAFLDLAFAS
QALTDEAQKLSNATKVAPTFGASDEYVQELRYLHKTIDPDGMCLSAGGFFRLNKSLLLT

>IsGr7INT
MTSFMQRQFVPYAIPFRVGGCFFIENFWKPLEHARITWSNLYVAYSASLVGVSFGVEMWDIVESSDILNNLSHALYP
CLLLILRAITNFKLLLNVVTMATGSIKFLEFFKKASIFEKATRFSPVRRGFWFFLTNHWSFMRQLVLIISLTSNCVI
SMVAFAVTVTNLLPNSFRFIGGLIAALICTCYLIYDVLPYIVLRSCSAVLVDYLQAQLRLFESCCNAKAVRAEGHLS
RQLEAIRHNFGMIRDLKESLNAIWQLPLAVMSVTVLLLVCVDCYGMFHDTFQGLGILLAVSYCLYAAFALVDLACVS
QFLTDEAQKFKNATKMALTFEVTGRHVQQMAGTFITYTVIIAQTGEELRNKATSGNSTIPN

>IsGr8PSE
MQWQFVSYAIIIWIGACFCIQNFZKSPDNAKATWMSLYIACSACLVVVFFCFEITPILKIFIAFNDLSHVFSSSLVF
ILRCLVCFKVLVNRASMATGTNRLLEFFKKSSIFEKKKTEFSPCSRGTRDILRPRWSFZRRSLVVLVTVSTYAILTZ
NLMSSLKQVYPLMZTFWASVLLSYLGZPTSSTILPHSWSZGTTLQSWWSIFKLNZNFWNVAVNDSLFELRSCLNNLV
IHHNIGNMZYLKDSLNVMWQVPLIVMSAGIILLVCVACHPMFFRLXFAPKFPLTASSSVYPSLAFIDMVFSSQSLPG
EAENFKIASKKAFAFEAVDGIRHQ

>IsGr9
MKSLMLHRFYAYGLLCRIGGCFFIQNFNRHSLDKARIAWKSLYTLYSALCVLFSFGFFIWFDVAFIIREASTAYGLS
GLFSETLSLTLHAVVSSRILINLSLMIAGSGKLLDFFRRAVIFEQTTGFEPAKCCAPLSRKPGWSLLRRTLVVVTLA
TSYVLLVNFYIVHYTGAISPEWALTSKVVGSIAAVFLFLYDSLCYVVLRCCSGVLLEYVSAQLRAFQDCSKPKDILP
QMQASRQLETIRLNVCSIRELTQILNSIWKASLAGKCAGIILANCVVLYSMFHDGVFKRQIWVTLSYCAYSSLAFLE
LVFISQALIDETQELKNATKKVRTSDATDNYAQELQYLHQSIDPKGMCLSGGGFFRLSKSLLVTMAGSIITYTVILV
QTSDELTSKMESVGAPPGS

>IsGr10
MRSFMLQRFAGYGKLCRIGGCLFIQNFHKESLASARVTWKCPYTLYSILCVCFVFSFEVAFLALRMRVLSLFSSRFT
QSLLFILHITIIFKIFINFWAMATGSGKLLDFFRKAVIFEKSTGFSCVKGRFRWPIPRRCLVLAALVANYVIGVRLF
IGEVVNALPRQWILAATICGYVAGFGFVLYDSLPFVVLRCSTEALVEYTHSQMLAFKGCDRTKGACTDMNASRRIET
IRLNLCNIRELNRLLNDMWKCPLTAMCANVILMSCIVLYSLFENGIYMREVWVVLLYTLYSALCFFELTLISQALSD
EVQRLKDATRAVITTDATEDYLHQLRVLHDTIEPLGMCLSGGGFFSLKKPLLVSMTAAIITYTVILVQTSDDITEKT
DVYSAFPRR

>IsGr11FIX
MSSYMLRRFARYGRLCRVGGCFFIKNFNEKSLEKATVTWKSLYTVYSTLCFCFFFWFEAAFIVQKAYVITFFSRSFA
HSLLFILHTVVSCKIFVNFSAMVVGSAKLLDFFRKSDTFEKSTGFAQPQKRRSPMVRRSLVIVALVISYVIGIHLFV
GDITNELPRQWVIAAKVSGYIAGVGFFLYDSLPFVVLMCCNEVLVEYTHAQLVHFKVCDRSKAACSDLDASRHMETI
RINLCQIRKLKDTLNTVWKWPLAAMSASILLILCIVLYAVFDGGLFLRDIWIILAYSVYSTLCFVEMTFVSQALMSE
AQRLKDATKAVLTTDATDPYGKELRYLHDVIDPVDMCLTGGGFFRLKKSLLVSMAGAIITYAVILVQTSDALAERIG
GDFSTTLKNWFNVTSSRNTTGESG

>IsGr12FIX
MNSFMLKRFAAYGMLCRLGGCFFIKDLRRNTLEKARVSWKSPYLLYSASCLTSIIAFQVTYIMKRVEVFNNISQTFS
RLLLIIILQTIITLKIGINFASMTTGSAKLLEFFRKSATFEKSTGFPVCKGSWTTSSTSPWSLLRRLCFAVALINSYV
ITMHFFVGGLANNLPPQWILAGKIVGCIAGLFFFLYDSLPYVVLRCCSSVLVEYIRAQLITFERCNESNVFRLESQT
TLQLEAIRCNLGFVKELKDSLNAAWKCPLAAMSTSIIFLVCVVFYSMFQDGVYKEQIWIALSYCVYSSLSFVEMAYV

SQALMDEAQKLKDATKRVHTSHATDDYARQLRYLHDSIEPKGMCLSGGGFFRLNKSLLVSMAGAMITYTVILVQTND
GLSNKIDSSNASMVGGIVVREPL

>IsGr13FIX
MSSFMQRQFMPYAVLCRLGGCFFIRNFRKPLENSNVVCKSVYTAYSAFIILLCFSFQVILFIRKAHVFKNFSHDFSP
FLLHIVRTIMILKALLNAVIMATGSATLLEFFRKSSAFEKTTGFSPSTQGVRGIWRRRWSFFRQSLVVIGAVITYFI
SAIPFITSLTEMLPTDLHFLRKLGVVIITAYYLLYDALPYMVLRSCSTVLIAYLQFQRKMFERCCELKSSYNKTELS
GQLEVIRHHLGHIRDLKDFLNTIWQVPLAAMSAAILLCACIVCYTMFHDGFSAEDIPLAVSFCVYSSLAFVDMALVS
QTLHDEAQKLKNATKTAFTFEAADVCVQQLRYLHETIDPKGMYLCGGGFLRINKALLVSMAGTMITYTVIISQTSDG
LANKAAPTD

>IsGr14
MQSVMLERFSLYGQLCRYGGCFFIQQLKSLENAKVVWKDLYTLYSATCVIFSFSFFLLLEVLFVLETNNFSTSIQSD
KFSDILIQTQHVVVSSKVLVNFLSMATGSGDLLNYFKKAAAFEKRSGFVPSKRCVRTLGEERWSLFRRVLVLVALAT
SYILFMHFYVAHVADTVARVWAIACKIIGPIAGFLFFLYDTLCYGVLRCCSGVLLEYIRAQLREFEDCTRSNGALSG
TEACRRLERIRLNMCSIRELSQNLNSTWNASLAATVAGIILANCVVSYSIFIDGIFEREVWIALAYCVYTSLVVLEL
VYMSQALMDETQKLKNATKNIRPFDLSRDCSQELRYLHDSIDPKDMCLTGGGFFRLNKPLLVSITGSIITYTVILVQ
TSNKLTSSTDFVVAPPAPYHK

>IsGr15
MSSYMLQRFAGYGMLCRFGGCFFIQNFSKKSLEKATVTWKSPYTVYSILCFCFFFWFEAAFIVQKAYVLTIFSRSFA
RSLLFILHTVVTYKIFVNFSAMVMGTTKLLDFFRMSGAFEHSTGFRIPEKHRWPMARCCLVVAVLVISYAIGIHFFV
GEVTNGLPRQWVIAAKVCGYIAGAGFFLYDSLPFVVLRCCTEVLVEYIHAQSLSFRDCDRSKVARTDQDASREIENI
RINLSQIRKLKDTLNDVWKLPLAAMSASILLILCVVLYSVFDNGLYLRDIWIILTYSAYSTLCFLEMTCVSQALMDE
AQRLKDAVRAVPTTDATEAYVQQLRYLHDVIDPVDMCLTGGGFFCIKKSLLVSMAGAIITYTVILVQTSDELAQKID
DALPTTSLKNWFNFSSTNAISQDG

>IsGr16FIX
MSSVMLRNFLPYGRFCRFSGCLFIQNFRKRPESMRVQWMSWYTIYSAFCFAVFAIVQASYIFERVILFLTNIRLFTK
SLFIVMQFAIVTKIVVNLSSNILGAASMVRFFRECAVFETSTGFSPPKPARRLKFCHCIRLAMLTAFLVCSVLSTTF
LIRRLLSPASGVLDVFVKIASVFSNYLFFVYDTHHFLILRPCSEVLILYIKAQADILSSALRVPDCWKRAATVDAVE
RVRLNNCKIRNLKTNLNGVWKASIVTSSVVILLMVCVAVYSAFDAGVPRSHLVLSMAYGVYSTLDFVDMATLSQTLV
NEAQKIKDSLKKVLTCQASESYVNQVHYLHNSLNPSDMALSGGGFFRLDMALLVSITGSIITYTVILVQTSEGAEHS
MARNITRYYVRVSNRTNFRTLRLTHSPP

>IsGr17CTE
MSSYMLQKFATYAMLCRLGGCFFIQNFRKDSLTNARVSWKSPYTLYTASCLAVIAIYQVTYMVKRVDILEDISRNFS
LSLILILQATITLKIAINFVSMVAGSARLLEFLQNSAAFEKSTCFLLCKGHGPSSSRRPWSLLRRLCIICALINSYV
LAMHFFVGGLLTKALPAEWILAGKIMGSVTGLFFYLYDSLPYVVLRCCTAVLVEYVRAQLIIFERCNRSNVFTLGSQ
ASQLLQVIRCNLVTIKELKQSLNAAWQCALAASSTGILFVVCIVVYSLFHEGLYKYHILTALSYCVYSSLSFMEIAY
VSQALADE

>IsGr18
MSSFMQRQFVPYAILCRLGGCFYIQNFRKPLENAEVTWKTLYTVYSALIVSFFFAFEMSSIIKISFVFRDLSRAFTA
SLMLLLRCMLCLKILVNTATMATGSSRLLEFFEKSSTYETISGFSPASRGVRGLWRHRWSFFCRSLVVLGVISTYVM
LTMYFTVSLMKKLLPANLRFLGIPSGVLFGVNYILYDALPYMVLRSCSAVLVDYLQAQLKSFESCCKSRSARCDRQLP
RQLEVIRYNLGVIRDLKDSFNAIWHVPLAAMSAGLILLVCVVWYAIFYEGLFAPQITLSASYCLYSSFAFIDMACVS

QALTDEAQKLKNVTKIAFTFEVTDGYTQQLRYLHETIDPDDMCLSGGGFFRLNKSLLVSMAGTMITYTVIISQTSDG
LTNNATPTN

>IsGr19FJ
MQPKGPLSPVMLRRFAAFGMLCRLGGCFFIQTFSSKSMENAKVSWKNFYTIYSASCFVSIASFQVAYVIHRAEILSD
ITHSFSRSILLILSSTVSLKMIINFVSIMAGSSRLLEFFRNSARFEASTGFLSARPFASVATNHLWSKFHRVLVAVA
LAISYAVGFHFFVSGLTELLPPQWILTGNILGVFVCALFHLYNSIPYMVLRCCSSVLVEYMRAQFVQFEGCKGLQGD
SSDAHASQAIEVVRLNLGVIKQLKDSLNSTWHWSLGATCSGIIFMTCVVLFTMFQDGVHHREIWVSVSFLVYSWLSF
LELVYVSQALVDEAQKLKDATKVAPMLHAAEGYIQQLRYLHDTIDPKGMCLSGGGFFRLNKSLLVSMTGSIITYTVI
LSQNSDDLSQKIDLYS

>IsGr20JI
MQPKGPLSPVMLRRFAAFGMLCRLGGCFFIQTFSSKSMENAKVSWKNFYTIYSASCFVSIASFQVAYVIQRAEILSD
ITHSFSRSIILIVGSTIALNMIINFVSIMAGSSRLLEFFRNSARFEALTGFLSARPFAIIATNHLWSKFHRVLVAVV
LAISYAVGFHFFVSGLTELLPPQWILTGNILGVFVCALFHLYNSIPYMVLRCCSSVLVEYMQAQFVQFEGCAQKLKD
ATKVAPMLHATEGYIQQLRYLHDTIDPKGMCISGGGFFRLNKSLLVSMTGSIITFTVILSQNSEDLAHKIDLYS

>IsGr21FIX
MQPKAPLSPVMLRNFAGFGMLCRLAGCFFIQSFSSKSVENAKVNWKNFYTIYSVTCLLSIVSFQVAYVIHRAEMISN
ITHSFSRSILLIVSSTVSLNMIINFVSIVVGSYRLLQFLRNSARFEASTGFLSARPFASVATNHLWSKFHRVLVVVA
LLNSYAVGFHFLVSGLTVLLPPQWILTGNIFGAFVCALFHLYNSIPYMVLRCCSSALVEYMRAQFVIFELCKGFQGA
RSDAHASQVIETVRLNLGVIRELKESLNSIWHWSLGATCSGIIFMTCVVLFTLFQDGVHHREVWVSVSFLVYSWLSF
VDMIYVSQALVDGAQKLKNATKVAPMLHAMECYIQQLRYLHDTIDPKGMCLSGGGFFRLKKSLLVLMTGTIITYTVI
LSQNSDDLAHKIDLYS

>IsGr22PSE
MSSTMVQRSALHAIFSRLHGCFFIQNFHGKSLKNAKVTWKTPYTFYSFSWFAVYIFIEILFSIRFAYVIQNISDALS
RSLLLVVLSVAVVKLTTNLAVMFTKPDKLLAFFRKSEAFETNTGFSPRSYSLLHSAADRWNAVRALAAFMGLVMYFS
LAEWFIMVELIQTVPTQWSVPVGNIGFLHGNRFLSFYDSLSYFFLRNCTNVLIEYIQVTVEGFQEANKWKHFHFQPD
APLQIEAMRLRINKFGSSRDTEZHLGRTLIVACAGTVIIDCVVVDAVFHDGIKKELWLGAGYFVYSSLCFIDLAYTG
QALVNEVRKLKSAILMVPAFGAPDTYLQQLRYLHESVDPEGMSFGGGSFFVLKKSLVLSMIGSVIIFGVILVQTSNS
VAFKINTT

>IsGr23PSE
MAGHSLATGQRTIAIHWPMTGQICDAWGCSFIHDFKRKSLNNAQVDWKTPYTFYSFSCFVIYLFLTTLFATRFAYVI
KGISDALSRTLLLVIYSVIVVKITTILAVMFTKWNKLLACVRKSEAFKSNTSFFVXPHSAWHSAAZIWSVGRVLAVF
VGLALYFAAAEWILMVELTSSMPPEWSDLVRLFDFFMGIGSMVYDPVLYLFLTTCTZVLEEYIHVQMKPFQEAXRED
FNIHPQFLLQIEAMRLRIFKVRQLKESLNIIWADTIIVACAITXADCVVLDAVFHDGTRKELWIAVSCELYASLCFN
DLAYTGQTLIDEXLPPTVVSRADYPYNQKVVYLHESVDAEKICLGGGGSFFLKKSLLPSMTGAIIIFGVILVQTSNF
QKLNIKAA

>IsGr24
MVSVMVQRCVPYAILGRLQGCFFIHNFRGKSLRNAKVTWKTPYTFYSISCYIFYILLETLFATHFARVIRNISDALS
RSLLLVVFGVVVVKVIANLSVMLTKPDELLVFFRKSEAFETTTGFSSCTRRSQDSAAVRWKVLRKCGVYMGQVLYFT
LTERFIMVDIAQSMPPEWSVPTKIFAFFLGIGFLCYESLSYFFVRSCTEVLVEYIQIQVELFQKAGELSHVGFQPPF
SSQVDAMRLRIDSIRKLKESLNNIWAGPLIVSCANTIIVDCVVVDAVFHDGIRTELWLVAGYSVYASLCFVDLAYTG

QAFIDEVRKLKSAILMVPTYGASDSYLRQLRYLHESVDPDEMCLGGGSFFVLKRSLLLSMTGSVIIFGVILVQTSNT
MSLRINAA

>IsGr25
MVSIMVKRSLPFAIVARLQGCFFIPNFGGNSLRNVKVTWKTPYTIFSISCFAFYMFLEFLFAKQFSHVVANISDTLS
RSLLLVVFGVCVVKVLVNLSVMLTKSKKLLAFYRKSEAFETSTGFSLHTHSLRHSSAHRWNAVRACGVYMALALCFT
NVERFILVDMAQSVPTEWSVLMKIFGVSLGFGFIFYESLSYFFLRSCIQVLGEYIQVQVELFQKDVQCSNVHLQPQF
SSQVQAVRLHMSKIKELKELLNDIWAEALIVTCANAIILDCVVLDAVFHDGIRKELWLAAFYSLYAPLCIVDLAFTG
QGLINEARKLQGVILMVPAFGAPESYLQQLRYLHESVDPDGMCLGGGGFFLLKRSLLLSMTGSIIIFGVILVQTSNT
VTLKINAG

>IsGr26
MTSMMVQRSTPYAIFCRLCGCFFIHNFRGKSLRNAKVALKSRYTFYSFSWFLLYMFLEALFSKRFGYVIRNISDPLS
RALMLVVLGVGLVKLITNLAVMILKPDKLLAFFRESEAFEMTTEFLPQAHSLRNSAAYGWHAVRAFSAVVGLGLFFI
EAERFIIVELSQSLSPQWSVPLRVIGFVAGTGYVAYDSLSYFFLRNCTKVLVKYIQVQVELFQKVGKLNNFYFLAQS
PHQVEAMRLRINKIKKLKESLNAIWAEPLIVACAGTIIIDCVVVDALVHDGIKKELWLAAGYSVYSTLCFIDLAHTG
QTLIDEVRKLKSAILMVPAFGAPESCLQQLRYLHESVQPEGMGLSGGSFFVLKRSLLLSMTGSIIIFGVILIQTSNT
MTLKVNAA

>IsGr27
MSSTMVQRVALYSLLCRLYGCFFIQNFREKTLANAKATWKALYTLYSISCFVLWFVIEMLCFTNYTDVVRSISDTLS
KTLLLVAYGVLVVKLIVNLAVMFTKPDKMLTFFRKSEAFENNTGFTPRTNSLLRSATDRWNMVRALAVFMGFALYLT
GALWYVRTELLKSIPPLWFVPVIILGTYMFIGFLLYDSVSHLFLRSCTNVLVQYIRAQAEVIKEAGKLTNFHLQSQS
PLQMEAVRLRINKIRKLKESLNEIWAGPLIVHCASTLVVDCVILDAVFHDGIRKELYIILICSLYTSIGFIDLAYIG
QTLIDEARSLKNTILMLPAFGAPDSYIQQLRYLHESVDPEGMCLGGKGFFALKRSLLVAMTGSVIIFGVILVQTSKS
MALKINAA

>IsGr28
MRSLMLQRAAPYAILCRLHGCFFIHNFRGNSLRNAKVNWKTPYTIYSLSFFGLYLILEEMYATRFTYVIRNISDTLS
KYLLLVIYGVVMVKIIANLTVMLAKPDKLLAFFLKSEVFETNTGFSPRTYSLQHSTFHRWNAVRAIWVFMAFVLFFT
EAERFMIAELTRSMPPQKSVPLTIFGFIMGSGFMVYDSLSYLFLRCCTKVLVEYIHVEVQGFQEAGKLQNIPFHLHS
PREIEATRLRMNNIRKLKESFNEIWEGPLILACASTIMVNCVVLDAMFHDGMRKELWLAVAYSLYSSLCFIDLAYTG
QSLIDEVRKLKSAILMVPAFEAPDCYLTQLRYLHEVVDPEGMCLGGGGFFVLKRSLLVPMTGSIIIFGVILVQTSNT
LALKNNAT

>IsGr29FIX
MSSTMVQRVALFSLLCRLYGCFFIQNFRGKSLADAKATLKSPYTLYSFSCFGLYFLLEAMFSTQYEGSVETISATLS
KTLLLVAYGVVVVKLIVNLAVMFTKPDKMLTFFRKSDAFERSTSFTPRTYSWRRSAKQRSSRVRARVVFMVYALYLT
VAEWYIMAEVLQSIPPRWSVPVIILGIIMGIGFFVYDSVSHVFLRSCTHVLVQYIRVQAEFIKEAGKLTNFPLHPKS
SLQMEAVRLRINKIRKLKDLLNDIWAGPLIVHCASTLLVDCVTLDAVFHDGIRKELWIIVICSLYTSVGFIDLAYTG
QTLIDEAHRLKNTILMLPAFGAPDSYLQQLRYLYESVDPKEMCLGGGGFLALRRSLLVAMTGSVITFGVVLVQTSKS
MARLVNAA

>IsGr30FIX
MSSTMVQRSALYAMLGRLHGCFFIHNFHGKSLKNAKVTWKTRYTIYSFSWFAVYIFIETLFSVRFARVIQSISDALS
RSLLLLVLCVAAVKLMTNLAVMFTKPDKLLAFFRNSEAFETNTGFSPRSYSLLHSAADRWNAVRALAAFMGLVMYFS
LAEWFIMVELIQTVPTQWSVPVVIFGFFTGTGFILYDSLSYFFLKNCTNVLIDYIQVQVEFFQNAGKWKNFQLQPQS

PLQIEAMRLRINKIRKLKETLNNIWAGTLIVACAGTVIIDCVVVDAVFHDGIKKELWIGAGYSVYSSLCFIDLAYTG
QALVDEVRKLKSAILMVPTFGAPDTYLQQLRYLHESVDPEGMCFEGGGFFVLKKSLVLSMIGSVIIFGVILVQTSNS
LTLKINST

>IsGr31FIX
MSSTMVQRCALYAILGRLHGCFFINNFHGKSLKNAKVTWKTPYTIYSFSWFAVYIFIEILFSIRFAYVIQNISDALS
RSLLLVVLSVAVVKLTTNLAVMFTKPDKLLAFFRKAEAFETNTGFSPRSYSLLHSAADRWNAVRALAAFMGLVMYFS
LAEWFVMVELMQTVPTQWSVPVGIFGFFTGTGFILYDSMAYYFLKNCTNVLIEYIQVQVELFQKAGRWKNFQFQPQS
PLQIEAMRLRINKIRKLKETLNNIWAGTLIVACAGTVIIDCVIVDAVFHDGIKKELWIGAGYSVYSTLCFIDLAYTG
QALVDEVRKLKSAILMVPTFGAPDTYLQQLRHLHESVDPEGMFFDGGGFFVLKKSVLLSMIGSVITFGVILVQTSNS
LTLKINST

>IsGr32FIX
MVERSTIPAIVFRVFGCFFVPNFSGESLAQAKVTWKSFYTCYSLACFVVYIFAETAFVIRSLDVLRDVSHSFSRSLM
LTVHVIVTARITGNLVAMLTGQEKLLEFFWNSESFEKNIGFLPHARSKRGKRSTRRWATMRMFLVVFGMVLCYAAGV
YYRIGQSAQSIGASWVLPVKIIGVCMAAGLVVYDSLSYLLLRNSATVLAEYIRAQLEAFKECRRSSSINLQNKVSGQ
IESIRLNMSKVKKLKESLNNIWNWPLMVASASLVIMNCIVFNGIFHDGFKQEIWLSITYALHASLCFIDLAFASQAL
VDEARELKNATLVVPTFETMEDLLHQLRFLHETIDPDAMCFSGGGFFSINNSLLVSITGSIIVFTVILVQTSDTIDA
DAA

>IsGr33
MSSYVEREFKFVARSCRLSGCLFVSNSWSGRFAEFRPNFRSWYALYFGFLGVVTCGFEITLLHRRISYIYMREKDFS
ELLFMIIHIVIGLNIATNTLVFILGTERLIDILRSTKRLEGAMGFEPARSSRVDDARKLFKMFLFAIFQAAFVLSRL
ASSKEIFQEPSTALTVIVTICFSLSCVGYAIHGTVVLNANMFFYSVLSEYLKPQVAIVETLSSQILARNPRYTAKIL
ERTRLHFVSIRNIVRSVDRLFEWGLVVSFLTCAFTLCFTLYSLFDASTSWSKMYIYIIYSVNSSANISELTHAAFRM
KQQALHIKHVLEKTPLVNLPRRLVLQVEFFAENIEAEQLCVTGSGFFTVDKPVLTSPKEHRAAISVLLIGRSAVDEI
AA

>IsGr34
MTVIETRFRRSTRILRWAGAWFIEDATNPARQPLKTMLTRPYTWYCIFCFSILFCIELSLIFWTLLFSFGERKMFLN
TLFVVLHITVVTKTLLSVVSLALSASKFKKLVNKARHFEVSRNFKPLPQHKKRITKASLRIWGQAILIVVFVVVRNT
DMMLMVEISNIFLAIVLNVVMGAASVLLVIYDGMYSTVLKGLVEIYVAYLKKEVDILKKARTATGPQASSILEDCRL
DVNSVQTLIRYTNRIMKYAIVIAYGGNLIMLCGIAYCLVDPTSKWSLRIFCFCYGVLISLDMVDIGFLVESLKMQAS
KMKWVLQSMNFLGLPDSFSKQVRFLHDCLESGQMDFSACGFFKVNLTLLISMGGAIITYTVILVQTSQGLSM

>IsGr35
MTFAYSQFRYSTRLLRWGGVWIVAEATNPGKQSFKTTLKRPYFWYCVLCLSTLVGTEFGNIIWALLFSFKHRKVFVS
GVYTATQITVLVKTMLSSLMVALAAGRLKKLVARANQFEIIRNIKIAPRSKKVTWRDIRIWGRVLFMVLFVSIRNMD
NLSILDVENIFGLGALVVVMTASSMLLVIYDCLYSTVFKSLVEIFIEYLRYEIRVLKKMKMELNSGPSMKMVEDCRI
EFNTIQGFVKSTNQVMRYAFVMAYAGNLIMLCNIVYLLVDTAATPWALRIFSSTYGILMWIDMIDNGVVAEGIKASK
MKWLLQSMPFQGLPDSFAKQVRFLHDIVDDSAMYFTGAGFFRINLPQLVSMGSTIITYTVILIQTSQGLQA

>IsGr36INT
MSNLAEQFDAVAKFGHATGSLFITRTSDGTSPKYRTMFRSLYSLYAMFIVGGCVIYEIFLLHFKVSGNSSLTTFSNT
VFNTLLVIIAIRIAANVSIILSLSGKLADVLNHAEDFKASLPVKSGLQRKNRSFIDLIRRFLMFLSFAVFTLSRYLF
FGELTSERPPSTATMATSAFVIVSTVVLTSACNFVHAIATLVYDLFTDDLGDLVAVAKVRLSPGSMLWGPRTARVLE

DTRLKYLAMRKIIQELNDVLQYSTFVTVTCTLLTLCTCAYLISETESSWGKLVFTASYAVASSLELVHITVSMSQLK
EQVQLFKESLDIQLFCASGLGFFTVDKPLLVSIFFSRLAYSLKVMQLCFDLLAIIIKISALRRMFI

>IsGr37
MLLMKQQQSSLRTYECRPDSFFAGIAVGAYVATIEPRRRMKDTDRLNTTIYILFLTSVNVEAVINNFLMFVKAPKFV
ELLHLCAKIEMNIGTPPYVQHDTISFTWKIMAFQAVLSCCNFVLNIISDFGTALVLSAEGQVSVDVMVIGILYSILG
VVYVSSLCLVTRLWMTYFSKAFTLYLSCIYRNLDQCLRSRSTPESRKVSLVDHTRVQLTLLKNCADLASSLLGPSLL
YAYAYSVALLCAAAYYTIIPELSNKIRLFFLCFGVLHWISILLPTVSAHRIKGAVIELRSIVQGVSMADFSDDLLAQ
LRMMLNSIRHDDLKFTGCGFFVVDLSTFADIMGAVITYTVVLVQTNDSYLKGSLEHCLENSTII

>IsGr38CTE
MYFARARFAIDAGLLAVAGCSFPPLNDSLKGSFTTWREAYAVACICVAVALEAFAYVGKFTSNPALSSLFNNTLFFV
IRIVNLVKVVALRFFLRAEARRVTELITQAEAYEESRNIRVRYRAPLFKTAYRCVSFVAVMSFFAARWHVYVKRLFS
NSPLPLKAFLDFLTVLSASCMTVWDGIHTILVRYFADVFLEYLKAENVALTALTQRKVVGFGRAMSTALRGIESNYE
EILRMVATARSVLRSLVFFGFTCNAVIVCAVLYSYTDGTSTISLLLSGTLYAAYTIAETLDITFAAETLATE

>IsGr39CTE
MGNNKMPAIYAGYRQFFVFCRIAGCCFVDGAFIRHGCSDLKIKIWSWYILYSLAGLWFYFWAMAVLIGSESNRPIFD
TPNMIFYGYNVLINIQAAISMLSLLRHSGTYLEIIKTCGDLEVAIGLPREQAQRKLEKISRRCLIFMILDSARGLAI
NKRVLPLSLRFMWSLHDWVKMGLLACFEVGVYLVGIWASLSFWLVVYNASVLKEYFACVNARMVQALTDPTGPAESL
QRVRLNHAALRGMVLKINNAFDLQVTLYYGISIYFLCASLYGVLLFPLTYADRAIRAIFVVCLATSVYVSARAAHNM
TSE

>IsGr40
MPAVREAYSAIYSGYRPFFLFCKLTGCCSIQGLWTKTLFDELKVKMTIWSALYSFLLLTCYVWTLVLFVEILVKQHF
QHPSSPISTVKGLFYGYYVLLYLQSTVNAFTLVRHAGALLAIIRDCSSLETQIGLEKDRVRRRLIIVSRGCLGFMVL
DCIKSLTLAYRVVPAAWLHLSWMHDWVKIVCVAFFLIGVMLVGLWFSMSFWMIVYNAYVLRHYFARVNELLVEGLSM
GGDCGRALQRVRWYQAEIRDIVSRFNSVLGLQSTFYYGGSVYFMCATVFGAFLSNISVLVRIVRSVFVITMAIGLLV
SARAGQKMTSERHLKKGEVPRCLFAFLRVKKVTWFLHLLLVTSEAGEKAFTGCGLFKVNLSMLVAISGAVITYTVVL
VQTDEEAVRQCV

>IsGr41JI
MDGRVPAAYPGYRFFLAVARISGCCFIDGVLFKTGPWMLRPNFRLLSLVHFAFCVFLSLWPPASFVMVRAQSRKTLS
QIHSITGYGFYAAIYGQALVNILNMAFKRSDLVDVVRMASQLERRLQVPKKAVERRLRQVSLMCFAFVLFDGFKYML
GLRTVMLLAFSLLDESHVVFRAVFIPGFLLGCVLVTVWYNLSFWMIVYFSEMVRQYFAALNDSLELALSTSKESFEA
AERIRTNLVALRKLLKKINSIIGVQALSYYAGSVFFLCATLYRILISEGALTDRVSRLTYLATMSAGIVISTRASHL
MSQELHMLVLAAEDAQGCLTGCGMFVINLPLIVVVVGAVITYTIVLVQTSDSAMNIKCLHGGITP

>IsGr42CTE
MIKRRRNSSIYNEVIRFPSFKDGFQTLSTFHRCLGYSFFTWQERQGITQVIVSVWRPYLLYALCSWTFFVFVMLQDT
YHVLFLAAEDNGDALKVIDKCILIFYFVRCIGIQIANSITVLLRSGRLREVVVALDTLETSFNRDTHLRSVAKIILS
LNVLFSVTALVSILDEISGFDGYMEPLHMKITYSVFSLLFAETVCMLCYTWAMFFGKVFEAFIRCINEDIESLATLK
QVRQLELDVLHNRFCDLSNAFGECNAILNTSLAVSVPLNILNASPWGYFILSTDGDAFHVFTDVLGFGTMCAELLVL
CVYGSAAQTQ

>IsGr43CTE

MRVLRPRVLAVSPPSSAMLASPFKIQPSYPSGKSLLSGFSVIAYFHRLLGFCFISKDANGRPVSKIIGPYMIYAFIS
WALYLFVIGSDIVRVSILLQDIRNRAIDKAIQILACVRCIGIEIATIVLLVTKSSQLVELLVTLEELEERLNRATSL
RATAIRVVILNVIFSVTSVLSISAEIYGFDEYSAEAYMKILYGVFSLVFAENVCMISFSWLMFFCRVFGVYLSHVNE
DIDCMSNELVVSIPELAELHRLFVNVGWAFARLEQLLGVAILVSFPLNIVSAAPWGYYMLKADKGTTMFMLDLIGFF
TICAEMLATGVYARATNRE

>IsGr44CTE
MFTESIMSPTSSTKTFHAAFHQVNRLHRTFGYSFISRSFSPSGEHITCNRLGPYTVYFVLSWSMTVGVFVYDAIEAL
AVYEDDEVLDKATTLLYSVRTISIQLCTMVAAVITAPKIRKVAAELGELEARLQRPTSLTRVSRNVLAANAIYSVVS
FVALMPLMFQFRELSKNQLYWNIVYIGVNLFYGQTSVMITYSWSMFFSKVFAELIRSINQELREMCSPSYSRESRDV
GDVHALFYGVIEAFEQCNSTFGISLVVLFSLNMLMAAPWGYWLIRNVGKPEVVSVNFLGFMVLCAQMAFVAIYSFYP
STE

>IsGr45CTE
MLSAPSKTRYGEHRPSIASVLIREEWNKAPEAHVIIERFLKMTRLLGCGFIEGLFTDNASTLRPQRASWYLVYTLTC
IGFIFACAVHGVRTNISRGTMDGDIYLAVCVFYLLQALATFLTMFMYAPQLVEIVTMCIEFEVRRPLALDQRRCLNH
FFMAVVVWLTLDFVVKNFLRMALVALSPSVYEFFLNATIVSGVLLMLSWSTIPQVGVVVMSRWLTVFLCETQNMLVR
CGELTGHFPLTVVTNYSR

>IsGr46FC
MLRRFLRMTRLAGCCFVEGLFASTEAGPKLTARRAVAVPPVLFVWPGVSWYHSFKSVLRNSSKATLDGDIYVALSAS
FFLATNATAISMVLHAPKLVELIHMCDAFELKRPLRQRRKLNRLCTWIVLLLCAFTLHQNAFRLQRLVTTATALHFV
RRLFTLLGVLFQLAWTHISPAGVFLMSRVLNAYAEEAHAALELIGE

>IsGr47
MLDQWHLAVPKNEGVEPTLFRVPDLGLDSSRSRNPRKVFLQDTRPSVWRARELMIPGVLMCAVAMPGPFFQFMNPRF
KLIMRMYQGLLYVGFTAYEAYRLIEFVEEFMKDEKTSLICFFHSLINTFLFPFVYIYVARKSESLGPLLSRWEDGHG
HLKFIPERNVLKPKFLVNVYMALTVFLIVLFHSVNAARSCYEIAWNYSNRTFPVKAVIFMGKTAHHYVLQTMYSGLE
SLAFSLMFLLWLLYNDFSCEVKTAPTLTIKTITAIREKYRSLCIVTEATANFLNSLLFLFFFRTSFDFMSSVIYYSM
QDGRAMKLWILVYEGILTFINVFHNTALAEMSSLLSLQAKDTLYEVSKVPAEPQAYKDLLLFLEVYRKRPEAMAGCG
VLQVDRALVLKLCGSTVTIVLILFQLDPNLSHKVSL

>IsGr48
MQRNIIVTSKTPVMPFNAKAWAFKPESEHDTKKKTDDDRGTTWDYIKMTLMYIYATHIVSNAIAGSIRTGSMMYLIE
TMTYTVRSVVSTVTTTYCFVKRGEINEIAQELQTFEGPEPTELLQKASRKRKYLRFSILCYSCLLIAMTSLFFVLVP
AQKYFDKCFYGINLEKAGIPNAPAIMIGLIEWNSYNIIVTGSPLLMPWYMYLCDHLRAQMLYFRVSQRGILDSGPLN
LAKFKRIQFMCAKMIDVERRLDNLFAPVLFLWIVDLLVNIVLPIRTLVNGIASFTLANVMSFFIEVIYSVSFFMILS
FSLAQVDKEYRDLDEEMYRVRNSVPGEDWQLCQQVVHMETGIKSSRFTLTGWGLFEVDRSFILTIVGAVATYTVVLI
QLTPGEETY

>IsGr49
MITSEKQIQQKAQQKMFRRNLYMQVLETGSKGLLDKIGILKWPLLLVAYAYTVHTTINVFLTFMRIHNMMKVLDIAG
YAARSFFACLNLRQAFQISTPSNRLLQRLSFGENQRRCFEVSTFLKVFVLVYFVVEVSLSIDFVLNGDIGEYVTSFL
YGTNISTTNMTQEVIKAATFFNLTLFDILSIVPGLLMADYIAACLRLRRLLASFRITVMDGRVKKTVTCTEVKRYQD
LSYDAWRELKRIDDIYTTVVFLWYLDIIINLVLSMRNLSKGISSRQFALDSAYYIVIFVTLSLSASSVDTEAKDLMQ
EVKQLRSNIDEDDWQTGGQILLLETGLQSSRIVLNSGHFCVIDRPFILGVVGAIATYTILVVQLTPPG

>IsGr50
MKVSSSFFGQSSARSRWAVKRLVWTVERSRNAKNQDVAIDVQVSQLANVGPFRFALKKALLLPTLWLLLCYGLHLAA
TIGSAGSTLTSFAYLLAVGNLIRAFTSIVSIVHIITFRNDILNILTSIENIFHDSLSEFVSRTRRFSLNLCAFCFGS
CLFHGTLICVSSLSGPWRDFYQARFYGVNCSRLPSAVRVIPILLDAPLLSITSSVTAMMACLFITVCYMLSLVTLHF
SHTMNLMLSLSASGKLTPGRVKDALLRLFLTGDAVCKLNMTYGPIMFWWYVDLLRSFLFSIPALLVAVTTSKEFFHY
SFVVVDLTRDVIVFLLMSLVASDMARHIEESVVHSLKVADSMDDVRSDVRLAVNVEMLVNAVQETKVQLSGREFFHV
DRSLINRVLSIVATFAIIVFQFLS

>IsGr51
MNSAQRSQTKKGPPQIDRSDVLRMFRGLFAMMKLVGLLPRDLPEVIEAEVDARSIARRMRRAGVLLFIVFGYLIHFS
AATVYNVTHDGGFFGFFANCGYVLRNIFAALSLVHFLVFQRVLLRIVVDGFRIFEHPPLSIERKVRRATVLAACFVV
STFVALQNTTVWVGFVDVQKYFNYYLYKGDVTQGTIPRQLGYLFSFIDATTYAIMESTLNCIITFHACVSLYLGCLC
ENFVRIIREVSQQTSVSGGQVKALRRLMTRLSDVMVRFDRVGSPVVFCWYANIVGSLILSTPGILLGMRRAAPSDYA
YMLTDLLTMLVILVALTFALADPTSLLRSSYVHALKISTKVDIDDEEVNHSAHVLMDSIISTKVAVTGCKCFQVTRD
MVLSILTMTSTYIIVVYQYIEHAM

>IsGr52
MNWAQYNLIKSGSSEIGRNDVLHMFRAMLVMMKLLGVLPRDLEGDIGHEIDARSIAKSMRRAGVLLFIVFGYLIHFS
AATIYNVTHDGGFFGFFANCGYVLRNIFSALCLIHFLVFQRVLLRIVVDGFHIFEEPPRGIERRIRRATVLAVCFDV
VSYLAIVNASSYCGYKDVQKYFNFYLYKGDVSNGTIHKQLGYVLSTLDATAYATIMSVIYWFISFHACVSLYLGYLC
ENFAEIVRKVSRQSSVSGGQVKSLRRLTTRLSDILVRFDRVGHPVVFCWYLNIVSTLILSAPGILLGMRKASPYDYG
YMLTDLITMLIVFVGVNFALADPARVFRSSFVHALKISTKVDINNEEVNHSAHVLIVSINSAKVAATGCKCFQVTRG
MVLAILSMVSTYIIVVYQFIENAL

>IsGr53
MEKSTPDRFRRVIRVSAAASRAFESNREHLQEDKNWLEMLFKELLVALKIHGIVIFKPDPNSVAPRKGNAKSLLRNV
RPSVILLVVFTSYGIHYAASSFTSLGRNPNGLLSLFSDVAYLFRIATALFTAFYMTSVSTSVSSLLSDSTTIFQKTL
PQNAIKSIRGYVIGMSVFAFANLLAFLGVKVTELYQSGFDGYYNYNLYDLAPKSKSVIYYAVPALDIAFCTIIITMP
KWIMGFHVSVCKYLGCQAVSLSGTFASERVVVLKRAREFREYHSALCELIFRFDEIFNLVLFFWYADIIISFVLSVP
YIIIRTDNSTPWTYAFVMVDVACNVALLVVFNMAASDPGRLARDAQLVVLKMSSRADPDDVRLNHELLLLANAVKVA
KVEMSGWNCFDVQRGLTITVLSMLSTYVVIVYQMMHHTL

>IsGr54
MPSSPVVSPETASKSVTMLEKCTRAAEEGTSITVPLKDVLKALRVFGIGPLTPRKLESRIKQGPSQDLTTHQIRYNQ
LAWVIMHIWIVRLLLRLGQVFLGKGKVGEAAVELLRGLASSVSLNRIILYRRRVCHFFWSVDHVTDRSLSYGKLSSF
TSVYTIGIWLYILVRLILDVANLFTTVGFKGYMEQWLLVDTIPGSLTFAVYIIAVPDTVIRRLILSGPTIFMISFYS
QLMWVIVSQFNSFHCTLKRKCTGSRNFGSDRLRQLRIRHADLCLLVQDVDDIMSPLAFLWHAMMVLGACAEATHLLQ
LKISEDAWAIVHISLDLAYMLVFFGIVSFSSAAVCQAYNATLNYVNLMSARMSQVDDAEFARQALLLMSQMQSISVS
VTAWKFYDMNRAALITTLGAVVTYVVVVFQMAPKLLQGPSE

>IsGr55
MKVGVRSFSTMIIFAPPRSKLLSQLEMLFKPLIYSFGSFSGPPGVSVSLRSRLTAYTTTLVVLTTLTCHLALLIMSL
SRGFEHASIRRGCTCVRLACSLIIVVLLIRRSHEVIAFKSRLLSLYSHLPVLDPSSVRLGKAKLVVWCTAAFVYLQI
SYATFQGFLPDTNEESMAAYFKELWFGLDNKHFPPLLTRCLWNLESFVFHVTTEAVVRVPVLFYVAACFLLKARLRD
FRLMLGRQRWKQSMTTLTTLTVKELRDLQRLHGILTEAAQQLEDVFSPIIFCSYTTFVVHIIASLYNIFDRNLLYFS
GAPGKIHLIRQVEVYLEFGLTVWFFLLLTVAAAFVNDEPSRLLPVVEKMILDVDELSVSSSFRAAFLLARFSRPFAQ
LTAWRVCVISRGLVLTVMGAFLTYGVIFFQFVHLGNSAAK

>IsGr56
MKKKPISKVFVPRSRNSESDAIFKIPMAALKFTGLFWNTTCRPARLLSFLLKISIVTTQAKLLSDAFTYETVDMVLY
GSRILTANVSFIIFALQERNLRNAIKDLSDKASFLLPLQRQRKIRTLSCSLACVSAIIIAVFLSGPAYVLFFTDKRL
QTDLLSRFVAYLNEVCFAVVIWYPLCFMPILFVNVSQTFAELLSQYNEMIPKLFCTENHNIYSLNCKFRHSREQRHE
MRRLLSVCGKIFAPCLFIWYGPTFLGCCAELSNFMRQSDAWVHRYYKAVTSAHGWAMFWGVSLAAHHVYATGRASWD
VLQDCTLRLPLDVGVHMELVMLKEDCRKIAMAFTIGGFYKLTLRTAFSVFSCMLTYAFVWYQIGPGSQPNVASHTNS
D

>IsGr57PSE
MKKKPIPKLFVPRSRNKESNAIFKIPLVALKFTSFFWNTTSRSARLLSFVLKISIDVTQTKLLSDEFAZQTVNLVLY
GSRILTANVSFIIFALQERSLRKVLKGLSDKAGCLLPLQKQZNIQMLNCTLACLSAIIIGVFLSGPAYIIFXTEKZL
QKDLLSHFVAYLNEVCFALVIZYPLRFMTTLFVTVSQTFAELLSQYNEMIPKRLCTEDYYIYSLDXKFTNSRKQRHR
MCHLLDVRVKIFASCLFIWYGPTLLGCCAELGTFMRQSDSWVQRYYKAVTSARGWAIFWXVSLVANPVYATRQVSWG
ILQDCTLTLSVDVVVHMELVMLKEDSREILKAFTIGGFYMLTLIPAFSVFSCMLTZVFVWYQIGPGSQRSAAIYTSS
G

>IsGr58PSE
SFLLKVSIDVTQAKLLSDAFTZQTVNLVLYGXRILTANVLFIISAXQXRSLRKVLKDLSDKAGCLLPLQKQZEIQTL
NCTLAGLSRIIIGAFLSGPAYILFFTDKLLQTDLLSHFVAYFNEVCFALVIWYTLCFMPIMFVTVSZTSARLLSQYS
EMILKRFXTEDYDICSLDCKFTDTTKQRHKMCRLLDDCCKIFAPCFFVWYGPTFLGCCAELSTFMRQSDSWMQRYYK
AVTSAHGXLAMLANPVYATRRVSWGILQECTLRLSLDVVVHMELVILKEDSRDIVMAFTIGRFYELTLKTAFSVLSC
MLTZAFVWYQIGPRSQRSAAIYTNSG

>IsGr59NC
SVFFTEANPKHSDRTLLFFVALIWYLNTLFVYGTLVFVPILFISFCLVLARAFKVHNVVIRNVHKSGLSLEADSLAQ
VRIFYERICTLVTDLNEIFGPVIFSWYIMIVLSVCIDMTQLFSDTNLLKNTKEDEGFLFSLRGIYSLLSFLGTCLAA
SRVSEEALAPLPHLHELTLRSWRLDMDTKMEAHFFLSRLSSSPVSMTGWNFFTINRSFILSVCAALTTYVVIIIQMN
PKAMKTINKLVTTALNNTGNGTASSE

>IsGr60NC
AGSSTELTKFIMVRQTVPKHTHKTIIDVTYVQVKHFRSLVRVCAKNSDVQAGPVKRLHSIYTSLWNAVQKLDSQLSL
AVFLWYVDLVLNIIISVRMVQHTLSQFNPYSAAGAYVQALYLGLMFLLMSYAAANLIVEVRHVDHDVCQLVCALTAV
DGQTCNQVMLLQEEVANCRMAFTGWNCFNIDRSFILTVIGAIITYAVVLQQLT

>IsGr61
MYTSRRTTKLFVIRHVDEKVDPENNRTVFKQLRPILWSLKLLGFYSDLYEDAERPVRPWYHDVSTWTCVTLGLLHAY
ALASLTACIEGDFWATAYNFFRAGSAVVSSQAVISKGPQACALIHRLGTFSSRPGHNLKKTCTVMVLVVLGYVILRI
AVHSMVLLDYNAHDLSKHAKIAWFGIESQLPASVLLPLCLVDVVLNSILVTGSLLLSVALYLALTVALRHRYEAFND
AINRHVRDNVVRQEETQDQSWTTTRENRGLDVHELRELRQIQTDLGVAVLEMETHFSPTAFAWVSFFVLGVCAEVSR
FLGHHESGLSDEHRILVIGLNLGSVTLLFVLLAVMSSRLSETSNASMMPLHRALGLSSDRPQEYHEGLLLISQLRAP
AVVLTGWGFFYFSRGFVLTLAGALLSYVIIILQLNSDLDKEKEIGKDG

>IsGr62CTE
MSSRIFTVEPRSCDDDPDTTDPFRIILTSLNLLGVFSLGPSSGGTIFRRCVIAYRSVATFYIHYVAFAHLYSLCSGV
RGWTDIITSFIACSSVFTLDSLSLRRERMECLLLSMRTClaEGSVKARWAVLRKSRVCTVCIWTYLAAFIVNSICSV

TLSNPQSSWDSYLLGANASRVSERKAKAVALVATTLRLYLVDGPWFFVMALFVLSCWVLRASFRDLEEKVTEDMSAE
DVSRLKERYCQLTAVVNELDDNLSPSLFVWYVAVLVALCSRFSAIVTRTSHEVQIFWWLTHLLGLLWTLAILFGVS

Supplementary Methods

Selection of *Ixodes scapularis* Wikel strain for genome sequencing

The *Ixodes scapularis* Wikel strain, established by Dr. S. Wikel (Quinnipiac University, Hamden, CT) was selected for genome sequencing. This colony was established in 1996 using approximately 30 pairs of field collected adult male and female ticks from New York, Oklahoma and a Lyme disease endemic area of Connecticut. At time of sequencing, the Wikel strain had been continuously in-bred from brother-sister crosses for twelve generations. Ticks derived from this colony have been found competent for transmission of *Borrelia burgdorferi* (strains B31 and 297) and *Babesia microti* isolates.

Genome Size

Prior to sequencing, flow cytometry was performed on propidium iodide-stained nuclei prepared from synganglia cells and used to estimate the haploid nuclear genome size of *I. scapularis* Wikel strain ticks as approximately 2.31 Gbp[1].

Construction of Genomic Libraries

Construction of Small, Medium and Large Insert Genomic Libraries

Total DNA was extracted from a single batch of *I. scapularis* Wikel strain embryos using Qiagen Genomic Tips GS-100 (Qiagen, Piscataway, NJ) according to manufacturer instructions. Embryos were surface sterilized in 10% bleach solution for 10 minutes prior to DNA extraction. Genomic DNA was used to construct small (~ 4 kb) and medium (10-12 kb) insert genomic, and large (40 kb) insert fosmid libraries at the J. Craig Venter Institute (JCVI) and The Broad Institute of Harvard/MIT.

Construction of a Bacterial Artificial Chromosome (BAC) clone library

An *I. scapularis* 10X BAC clone library with an average insert size of ~120 Mbp was produced by the Clemson University Genomics Institute (CUGI). The library comprised 184,320 independent clones which were arrayed to nylon filters. ^{32}P-labeled *I. scapularis* genomic DNA was hybridized to the filters and used to identify clones with a high repeat content using published procedures[2]. Forty-five clones that failed to demonstrate a strong hybridization signal were selected for complete BAC sequencing and assembly (Supplementary Table 4).

Genome Sequencing and Assembly

Ixodes scapularis Nuclear Genome

The genome of *I. scapularis* Wikel strain was sequenced in a joint effort by the Broad Institute and the JCVI and funded by the National Institute of Allergy and Infectious Diseases, National Institutes of Health (NIAID/NIH). Sequence data were generated by Sanger shotgun sequencing of the genomic libraries described above. Sequence reads were assembled with the Celera Assembler (CA) software, which is available as open-source (http://wgs-assembler.sf.net). The original version of the CA software[3] had been modified to assemble at low sequence identity[4], to report high quality SNPs and longer variants[5,6], and to trim reads based on partial overlaps to other

reads[7]. Running on Sanger data only, the *I. scapularis* assemblies did not use the CABOG unitig module developed for 454 pyrosequencing data[7]. An initial assembly was generated with CA version 3.1 before the completion of sequencing. Subsequent assemblies used CA version 4.0. The final assembly incorporated parameter settings and process modifications chosen to increase assembly contiguity on this data. The final assembly, labeled Assembly D in Supplementary Table 1, was deposited in GenBank as JCVI_ISG_i3_1.0 and has the VectorBase designation IscaW1.

Analysis of reads. K-mer analysis indicated high polymorphism in these data, where K-mer is defined as K consecutive basecalls in a read. For a read of length N, M=N-K+1 is the precise number of K-mer instances and an upper bound on the number of distinct K-mer sequences. Each distinct K-mer sequence has some frequency F across all the reads. Distinct K-mers with F=1 are single-copy. Single-copy K-mers may be induced by sequencing error, low coverage across polymorphic loci, or low coverage in general. Single-copy K-mers are useless as alignment seeds. Celera Assembler uses K-mer matches to seed sequence alignments and thus to detect pair-wise read overlaps. At K=22, CA's default, 50% of *Ixodes'* distinct K-mers are single-copy and single-copy K-mers cover 12% of the data. Smaller values of K were required for sensitive overlap detection, especially in polymorphic regions of the genome. At K=16, the single-copy K-mers make up 25% of distinct K-mers and cover 2.6% of the data.

K-mer analysis also indicated high repetitiveness in these data. An F value of 50 was considered high frequency for a K-mer in these data. At K=16, just 1.8% of the distinct K-mers displayed high frequency in the reads, but these K-mers covered 56% of the data. This indicated that larger values of K would be required for specific overlap detection, especially in repetitive regions of the genome. Obviously, there were compelling and competing demands on the assembly parameter, K.

Trimming of reads. Reads were trimmed using CA's overlap-based trimming (OBT). The initial assembly used CA defaults. The trimming was based on each read's partial overlaps (local alignments) to other reads, where overlaps were discovered with the K-mer seed size K=22 using K-mers whose frequency in reads was greater than 1 and less than the frequency of the top 1% of most frequent K-mers. The software trimmed reads that (a) had a span confirmed by overlaps and (b) had some position at which overlaps consistently broke off. Analysis of the initial assembly uncovered anecdotal evidence of insufficient trimming. In an effort to improve trimming of this data, the later assemblies incorporated pipeline modifications designed to uncover additional partial overlap evidence. Assembly B was run with parameter changes that specified small seeds (K=16) and a low frequency threshold for seeds (freq<=50) at the default minimum overlap (length>=40). These parameters were chosen for high sensitivity among non-repetitive or polymorphic sequence. Assemblies C and D also incorporated large seeds (K=28) and high frequency threshold (frequency<=8000) with a large minimum overlap (length>300). These parameters were chosen for high specificity among repetitive sequences. Thus the OBT stage of assemblies C and D used the union of overlaps computed under two regimes. Celera Assembler's chimer detection option was disabled during assemblies C and D because the ratio of partial overlaps per read seemed to induce over-calling of chimera.

Overlaps and unitigs. Celera Assembler computed full overlaps between reads that shared a K-mer subsequence. Without changing the reads, the CA optionally corrected the observed error rate per overlap for all reads whose overlap collection

indicated a correctable basecall error. It then filtered the overlaps by error rate and used the surviving overlaps to construct unitigs, or high-confidence contigs.

Assemblies A and B used default settings, including 22-mer seeds, an alignment error threshold of 6% before correction, and 3% threshold after correction. Assemblies C and D used more permissive parameters: small K-mer seeds (K=14), a high frequency threshold for K-mers to use as seeds (frequency<=8000), and high tolerance for alignment error (mismatch<=20%). In assemblies C and D, 99.98% of the distinct K-mers were used as seeds, and the seeds covered 90% of the sequence data. The resulting overlap collection included 55 billion overlaps. In assemblies C and D, the correction option was disabled to avoid using high-error overlaps for correction. The unitig module was tested with several values of the overlap error rate filter and finally run with a permissive value (mismatch<=13%).

Contigs and scaffolds. Celera Assembler built contigs and scaffolds from unitigs and the mate pairs that unitigs incorporated. Unitigs were evaluated by the A-stat statistic that compares observed to expected coverage[3]. Unitigs with high coverage, presumed collapsed repeats, were precluded from nucleating contigs but reserved for possible incorporation into multiple scaffolds later. For assembly D, the genome size was set explicitly (size = 1 Gbp) to a smaller-than-expected value, effectively increasing the expected coverage. The goal was to incorporate more unitigs early in the scaffold building process. Assemblies A, B, and C had used the default behavior in which genome size is estimated from the unitigs at run time.

Assemblies A and B used default settings that allowed up to 6% error when merging unitigs into a contig, and up to 6% when recovering trimmed sequence from reads at contig ends to close a gap. Sequence analysis of contig ends in initial scaffolds indicated that polymorphism was preventing well-supported merges. In assemblies C and D, the error tolerance was increased to 20%. The CA consensus module failed on seven contigs, possibly due to accumulation of pair-wise error in the multiple sequence alignment. These seven alignments were inspected visually and adjusted slightly so as to permit continuation of the CA computation.

Supplementary Table 1 captures the effects of our assembly interventions. Adding 1/3rd more reads after assembly A increased the sizes of the maximal scaffold and contig, but had little effect on total span or N_{50} values (compare columns A and B). Adjusting K and other overlap parameters greatly increased maximum, mean, and N_{50} values (compare columns B and C). The drop in total span of scaffolds and contigs was partly due to combinations of previously separate contigs. The result of adjusting the genome size parameter was to increase the mean and N_{50} for values for scaffolds while slightly decreasing them for contigs (compare columns C and D). Each successive assembly incorporated more reads into contigs. Assembly D incorporated 44% of input reads in contigs. Assembly D left 2.2M reads (13% of input) in unincorporated unitigs called “degenerates” and 7 M unassembled reads (42% of input) called “singletons.” Of the 15.6 M reads that had a mate constraint after trimming, assembly D scaffolds satisfied the constraint for 3.8 M reads (24%).

The size and distribution of DNA on the IscaW1 scaffolds is shown in Supplementary Table 2. The longest scaffolds range from 1-4 Mb and comprise approximately 3.6% of the genome. Approximately 48.9% of the genome is represented by scaffolds ranging from 10-100 Kb and scaffolds of 10 Kb or less comprise approximately 23.6% of the genome.

Sequencing, Assembly and Analysis of *Ixodes scapularis* BAC Clones

Forty-five BAC clones selected from the *I. scapularis* 10X BAC library were shotgun sequenced and assembled (Supplementary Table 4). BAC sequence accession ranges are: AC192414-AC192429, AC192742- AC192744, AC200531, and AC205630-AC205654. More than 185,000 BAC clones were end-sequenced and trace reads are available at VectorBase (https://www.vectorbase.org/).

The assembled BACs were aligned to the *I. scapularis* IscaW1 annotated scaffolds using Mummer (Supplementary Table 4; Supplementary Fig. 4). Of the 45 BACs, only 12 align to a single IscaW1 scaffold, six align with between two to four IscaW1 scaffolds, and the remaining BACs align to ten to more scaffolds. Analyses of BACs with multiple hits to IscaW1 scaffolds failed to identify any potential coding sequence. Repeat-rich regions were identified in assembled BACs utilizing an in-house repeat library built using RepeatScout. Of the 45 sequenced BACs, 21 are composed of low complexity regions and do not contain gene structure suitable for annotation (data not shown). Pfam genome alignments show that repeat associated domains are common and include extensin like, formin, reverse transcriptase, integrase, endo-exonuclease phosphatase, Pao, and PF00075, an RNase H domain for an enzyme involved in retroviral replication, that is often found in association with reverse transcriptase domains (data not shown). The most prevalent retroelement had the following arrangement of domains: PF03372 (Endo-Exonuclease phosphatase)-PF00078 (Reverse transcriptase)-PF00665 (integrase). Some element regions were found that lacked the PF03372 Endo-Exonuclease phosphatase domain, and less often the Integrase domain. To determine gene content in the BACs, homology searches were performed using protein databases (NR Genbank non-redundant database, Pfam domains, and annotated *I. scapularis* IscaW1 peptides), and *I. scapularis* EST data (Supplementary Table 5). The remaining 24 BACs contain various amounts of coding sequence.

***Ixodes scapularis* Mitochondrial Genome**

The mitochondrial (mt) genome of *I. scapularis* was assembled from trace sequence. The genome assembly and manual annotations are available at VectorBase (https://www.vectorbase.org/). Phylogenetic analyses were performed to compare the *I. scapularis* mt genome to that of published mt genomes from other species of Ixodida and other arthropods. Supplementary Fig. 10 shows the organization of the mitochondrial genome of *I. scapularis* and comparison of mitochondrial gene arrangement between *I. scapularis* and other ticks and arthropods.

Rickettsia Endosymbiont of *Ixodes scapularis*

Analysis of *I. scapularis* trace reads revealed a substantial amount of reads comprised of bacterial DNA. Extraction of 16S rDNA sequences and subsequent comparative analysis with other bacterial species suggested that one organism with close affinity to members of the genus *Rickettsia* (Alphaproteobacteria: Rickettsiales) was simultaneously sequenced with *I. scapularis*. This organism was named Rickettsia Endosymbiont of *Ixodes scapularis* (REIS). The genome of REIS was assembled and annotated as a separate effort[8]. Briefly, ten previously sequenced *Rickettsia* genomes were used to recruit REIS reads from the *I. scapularis* read set, with subsequent

scaffold recruitment and assembly yielding 109 contigs linked into one chromosome spanning 1.82 Mb. In addition, four rickettsial plasmids (pREIS1-4) were obtained. The annotated genome is available at GenBank (ACLC01000000) and PATRIC[8]. A rickettsial isolate cultured from *I. scapularis* ovaries was recently named as *Rickettsia buchneri* sp. and may be identical to REIS[9].

Among sequenced *Rickettsia* genomes, REIS is the largest to date (>2Mb) and contains 2,309 genes across the chromosome and four plasmids[10]. The 109 gaps in the assembly reflect the extremely high repeat nature caused by an extraordinary proliferation of mobile genetic elements (MGEs), which are dominated by >650 transposases (TNPs). TNP-mediated recombination events have resulted in dozens of pseudogenes, and also contribute to limited synteny with other *Rickettsia* genomes. An integrative conjugative element named RAGE (Rickettsiales amplified genetic element) is present on both the REIS chromosome and plasmids, encoding F-like type IV secretion system genes and many genes characteristic of the intracellular mobilome. The abundance of TNPs relative to genome size, together with the RAGEs and other MGEs that encompass ~35% of the genome, place REIS among the most repetitive bacterial genomes sequenced to date. Despite the proliferation of MGEs in the REIS genome, a typical core rickettsial genome was obtained, characteristic of reductive genome evolution as a consequence of an obligate intracellular lifestyle dependent on the utilization of host metabolites. Robust phylogeny estimation places REIS ancestral to the spotted fever group rickettsiae, containing the agent of Rocky Mountain Spotted Fever, among other pathogens.

***Ixodes scapularis* Genome Annotation**

The annotation of the *I. scapularis* genome was performed via a joint effort between the JCVI and VectorBase. The genome annotation release IscaW1.2 is available at VectorBase and GenBank (accession ID: ABJB010000000). A total of 18,385 scaffolds (17,365 >10kbp and 1,020 <10kbp; ~5% of the assembled scaffolds) were annotated, containing 20,486 protein-coding genes, and 4,439 non-coding RNA genes. Supplementary Fig. 5 shows that the majority of *I. scapularis* expressed sequence tags (ESTs) map to scaffolds of 10 Kb or greater in length, thus providing justification for this approach. *Ixodes scapularis* gene, intron, and exon statistics are shown in Supplementary Figs. 1-3 and Supplementary Table 3 in comparison to those for multiple sequenced invertebrates.

The JCVI and VectorBase annotation pipelines utilize complementary approaches; the former focuses on *ab initio* gene predictions, while the latter utilizes primarily similarity-based methods. Both pipelines were run independently and the resulting outputs were merged by JCVI into a single consensus gene set. Several iterations of merging and manual review were performed. Updates to the gene set are performed on a regular basis at VectorBase.

Repeat Identification

The *I. scapularis* genome sequence was masked for repeat sequence prior to annotation. Publicly available repeat sequences were obtained from GenBank and *de novo* repeat identification was performed by JCVI using RepeatScout[11] and by VectorBase using RECON[12]. Repeat sequences were merged into a single library that serves as input to RepeatMasker[13] to mask the genome (data not shown).

J. Craig Venter Institute Gene Prediction Pipeline

An initial set of *I. scapularis* protein predictions were generated using dipteran protein sequences obtained from GenBank and aligned to the *I. scapularis* genome sequence using the programs AAT[14] and GeneWise[15]. The *I. scapularis* EST set comprising 193,151 EST and cDNA sequences was aligned to the genome sequence (Supplementary Fig. 5) and high quality alignments were used to produce automated annotations based on gene structure using the software package PASA[16]. ESTs were also used to evaluate and capture potential genes in small contigs that were not initially included in the annotated scaffolds. EST hits to small contigs that are not part of the annotated scaffolds typically represent transcripts derived from transposable elements such as non-LTR type elements and do not contain an open reading frame. Finally, the *ab initio* gene prediction programs Augustus[17] and GeneZilla[18] (formerly known as TIGRscan) were used to generate gene models. VectorBase homology-based gene predictions were then incorporated into JCVI database and the gene sets were subsequently combined using EVidenceModeler[19].

VectorBase Gene Prediction Pipeline

The Ensembl pipeline[20] was used to predict non-coding and protein coding genes based on mRNA, EST/cDNA and protein evidence. The supercontigs were masked with the repeat libraries described above. UniProt protein sequences[21] were mapped to the *I. scapularis* supercontigs using the Genewise program[15]. Two gene sets were produced based on the taxonomic origin of the proteins: (1) a “targeted” gene set from *I. scapularis* proteins only, with strict criteria, and (2) a “similarity” gene set from the remaining proteins. In the “similarity” gene set, gene predictions were prioritized according to protein origin: genes based on phylogenetically close species were placed first on the genome, then non-overlapping models based on more phylogenetically distant species were added, and finally eukaryota- and metazoa-based gene models were used to fill in gaps. Independently, the *I. scapularis* EST and mRNA sequences were mapped to the supercontig sequences using the Exonerate program[22], generating a third gene set. Finally, a fourth *ab initio* gene set was produced using the SNAP program[23] and supercontig sequences, and retaining only those predictions containing a Pfam domain. The four gene sets were merged into a single gene set that was then subsequently combined with the JCVI gene predictions.

Supplementary Figs. 1-3 show a comparison of haploid nuclear genome size (in Mb) to features associated with the coding fraction of the genome (gene/exon/intron number and length) for 12 sequenced arthropod genomes based on EnsemblGenomes release 12. While *I. scapularis* has the largest haploid genome of any sequenced arthropod, the gene number and length, exon number and length, and intron number and length statistics for *I. scapularis* are similar to those for other sequenced arthropods. Together, these analyses suggest that the genome size of *I. scapularis* reflects the accumulation of significant amounts of non-coding sequence.

Sequencing of the *Ixodes scapularis* Transcriptome

As part of this project, 183,834 *I. scapularis* EST sequences were generated by Sanger sequencing of a pooled *I. scapularis* stage and tissue library and are available at GenBank and VectorBase (ESTs accession range: EW781064-EW964897). The cDNA library was constructed from total RNA extracted from the following stages: *I.*

scapularis embryos, blood fed larvae, nymphs blood fed for 1-3 days, fully engorged nymphs, unfed males, unfed females, and adult females blood fed for two, four and seven days. The majority of ESTs align to IscaW1 scaffolds ranging in size from 10-500 Kb (Supplementary Fig. 5).

Gene Ontology Analysis of *Ixodes scapularis* Expressed Sequence Tags (ESTs)

***Methodology**.* The predicted protein sequence of the 24,925 *I. scapularis* gene models (protein coding and non-coding RNA genes) was downloaded from VectorBase (https://www.vectorbase.org/) in March 2012 and the program Blast2GO[24] (http://blast2go.de) was used to predict functional classification for each sequence. The Blast2GO program performs a homology search against the NCBI non-redundant (NR) database and assigns sequence to one of three gene ontology categories (biological process, cellular component and molecular process). Statistical analyses were performed using default settings and pie charts showing assignment to predicted functional category (Supplementary Fig. 6) were generated using a cut-off minimum of 1,000 sequences.

Blast2GO annotations were obtained for approximately 50% of the 24,925 *I. scapularis* predicted protein sequences. The majority of annotations were inferred based on similarity to sequences for *I. scapularis* and the tropical bont tick, *Amblyomma maculatum*, followed by sequences for *Homo sapiens*, *Mus musculus* and *Pediculus humanus.* The majority of GO classifications were inferred based on electronic annotation only. Blast2GO classified the *I. scapularis* sequences into thirteen “Biological Process” functional groups (Supplementary Fig. 6a). For the “Cellular Component” category, the program classified sequences into six functional categories, namely “cytoplasmic part”, “intracellular organelle”, “nucleus”, “intracellular non-membrane-bounded organelle”, “integral to membrane” and “protein complex” (Supplementary Fig. 6b). For the “Molecular Function” category, more than 50% of the sequences were classified as either “hydrolase activity”, “protein binding” and “transferase activity”, while the remaining sequences were classified as “zinc ion binding”, “nucleic acid binding”, “transposase activity”, “oxidoreductase activity” or “purine ribonucleoside triphosphate binding” (Supplementary Fig. 6c).

***Ixodes scapularis* Gene and Genome Evolution**

Comparative Evolutionary Analysis of the *Ixodes scapularis* Gene Repertoire

Molecular Species Phylogeny. To estimate the average rate of amino acid substitutions in the conserved cores of orthologs shared across multiple invertebrate and vertebrate species and to reconstruct the arthropod phylogenetic tree, single-copy orthologs from www.OrthoDB.org[25] were selected from *I. scapularis* and 11 additional species, including the Crustacean water flea, *Daphnia pulex*, five insects: *Pediculus humanus*, body louse; *Nasonia vitripennis*, jewel wasp; *Tribolium castaneum*, flour beetle; *Anopheles gambiae*, malaria mosquito and *Drosophila melanogaster*, fruit fly; and five outgroup species: human, mouse, chicken, zebrafish and *Nematostella vectensis* (sea anemone), resulting in 524 Strict Single-Copy (SSC) Orthologous Groups (OGs), with one gene from each species. Multiple protein sequence alignments were performed with MUSCLE[26] for each OG, and conserved well-aligned cores were extracted using GBlocks[27] (>66% conservation, 100% flanking, maximum of 8 non-

conserved positions, minimum block size of 4) resulting in 90,763 aligned amino acids, of which 67% showed variation. The phylogenetic tree was computed with PhyML[28] employing the JTT substitution model, estimated proportion of invariable sites, four substitution rate categories, estimated gamma distribution parameter, empirical amino acid equilibrium frequencies, optimized tree topology search, branch lengths, and substitution model parameters, with 100 bootstrap replicates (Fig. 3a).

Intron Evolution. The identification of introns in well-aligned sequence regions of single-copy orthologs across representative arthropod and non-arthropod species was performed in a manner similar to that employed in other studies[29]. 524 Strict Single-Copy (SSC) orthologous groups (OGs) were selected from www.OrthoDB.org[25] with one gene in each of the 12 selected species (*NVECT*, *Nematostella vectensis*; *HSAPI*, *Homo sapiens*; *MMUSC*, *Mus musculus*; *GGALL*, *Gallus gallus*; *DRERI*, *Danio rerio*; *ISCAP*, *Ixodes scapularis*; *DPULE*, *Daphnia pulex*; *PHUMA*, *Pediculus humanus*; *NVITR*, *Nasonia vitripennis*; *TCAST*, *Tribolium castaneum*; *AGAMB*, *Anopheles gambiae*; *DMELA*, *Drosophila melanogaster*). A second, larger set of OGs was selected allowing no more than three paralogs in three species and selecting the longest protein per species, resulting in 1,529 Relaxed Single-Copy (RSC) OGs. The introns were mapped on to the protein sequence alignments, allowing for small splice site changes (one amino acid difference) [as observed in other studies[30]], and conserved regions with an intron in at least one species were identified by requiring >30% amino acid identity in the aligned blocks of five columns before and after the intron position, and no species with any missing sequence in the region, resulting in sets of informative intron positions in each species (Supplementary Fig. 11). From a total of 44,222 SSC and 135,216 RSC introns, between 32% and 52% of introns in each species are located in well-aligned core regions of the ortholog alignments and may therefore be compared across the 12 species. Using strict or relaxed orthologous groups (SSC or RSC) does not affect the proportions of informative introns. The non-arthropod species have the most introns, the Dipterans have the least, and *ISCAP* has the greatest number of introns and informative introns among the arthropods. Informative intron positions from the five outgroup species (*NVECT*, *HSAPI*, *MMUSC*, *GGALL*, and *DRERI*), and the five insects (*PHUMA*, *NVITR*, *TCAST*, *AGAMB*, and *DMELA*) were compared to *ISCAP* and *DPULE* to quantify shared and unique intron positions across all 12 species in the strict (SSC) and relaxed (RSC) sets of orthologous groups (Fig. 3b; Supplementary Table 7). Comparing the 18,987 SSC and 53,322 RSC informative introns identified 4,621 and 13,459 intron positions, respectively. Only 42 SSC and 113 RSC intron positions are conserved across all 12 species. Examining pairwise conservation of intron positions between *ISCAP* and each of the other eleven species shows the greatest sharing with the non-arthropods (*NVECT*, *HSAPI*, *MMUSC*, *GGALL*, and *DRERI*), about 3 times more than with *AGAMB* and *DMELA*, and about 1.5-1.8 times more than with *DPULE*, *PHUMA, NVITR, and TCAST* (Supplementary Table 8).

To reconstruct the 12-species phylogeny based on conservation of intron positions, presence/absence matrices for the 4,621 SSC and 13,459 RSC intron positions across the 12 species were used to compute Euclidean distance matrices with 1000 bootstrap samples in R (Development Core Team 2011). These matrices were used to compute Unweighted Pair Group Method with Arithmetic Mean (UPGMA) and Neighbor Joining (NJ) trees using the neighbor program from PHYLIP[31]. The resulting

trees were ordered and compared using the Newick Utilities[32] to identify bootstrap support values for the consensus trees. Employing the intron presence/absence data as a phylogenetic signal successfully reconstructs the species tree from both the strict and relaxed sets of orthologs using both UPGMA and NJ algorithms (Supplementary Fig. 12). *ISCAP* consistently shows greater similarities to the outgroup species - vertebrates and the sea anemone - than to the pancrustaceans.

To compute intron gain/loss estimates across the phylogeny, the presence/absence matrices for the 4,621 SSC and 13,459 RSC intron positions across the 12 species were analyzed using the MALIN suite for maximum likelihood analysis of intron evolution in eukaryotes[33]. Intron gain/loss rates were first optimized, and then presence/gain/loss estimates were computed with the Dollo Parsimony (DP) and Posterior Probability (PP) algorithms (Supplementary Fig. 13; Supplementary Table 9). The greatest numbers of losses are estimated to have occurred on the Pancrustacea branch, from 1.6-1.7 (DP) to 3.4-3.5 (PP) times more losses than on the Arthropoda branch. *DPULE* stands out as having a large number of intron gains, in agreement with results from the analysis of the *D. pulex* genome[34].

To compare lengths of introns among the 12-species, the base-pair lengths of all identified pairwise orthologous introns for the strict and relaxed sets between *ISCAP* and each of the other eleven species were collected from their corresponding General Feature Format files. Wilcoxon tests were performed in R (Development Core Team 2011) to evaluate statistical differences in length distributions between species (Supplementary Fig. 14; Supplementary Table 10). Examining the distributions of orthologous intron lengths shows that *ISCAP* introns are most similar to those of *MMUSC* and the other vertebrates, but more than an order of magnitude longer than introns shared with pancrustaceans.

Orthology. Examining groups of orthologs delineated across 33 arthropod species from www.OrthoDB.org[25] identified about a quarter of *I. scapularis* genes with recognizable orthologs in each of the representative species selected from six different arthropod lineages: Crustacea, *DPULE*, *Daphnia pulex*; Phthiraptera, *PHUMA*, *Pediculus humanus*; Hymenoptera, *NVITR*, *Nasonia vitripennis*; Coleoptera, *TCAST*, *Tribolium castaneum*; Lepidoptera, *BMORI*, *Bombyx mori*; and Diptera, *DMELA*, *Drosophila melanogaster* (Supplementary Fig. 9). A further quarter of *I. scapularis* orthologs are less broadly conserved across Arthropoda, with gene losses in other species resulting in more patchy phyletic distributions. Of the remaining genes with no identifiable orthology, about half exhibit homology (BLAST *e*-value $<1e^{-05}$) to genes in the other six representative species or to other *I. scapularis* genes.

Gene Duplications in *Ixodes scapularis*

Protein clustering of arthropod genes was performed for *I. scapularis* and ten other arthropods, using reciprocal BLASTP and OrthoMCL clustering methods. Proteome sources for *I. scapularis* and two additional chelicerate species, three Crustacea, five Insecta and two vertebrate outgroup species, as available in 2011, used for these analyses are listed in Supplementary Table 11. To address a deficit of non-insect arthropod gene sets, two transcriptome datasets were included in the analyses, one for the dog tick, *Dermacentor variabilis*, and a second for the shrimp, *Pandalus latirostris*. Similar genes, measured with reciprocal best BLASTP were clustered using standard methods outlined for OrthoMCL[35]. OrthoMCL has practical advantages over related techniques in identifying orthology, and compares favorably in detecting true

orthology[36]. In the present study, significance criteria were applied as per recommended options. Specifically, these criteria were a similarity *p*-value ≤ 1e^{-05}, protein percent identity ≥ 40%, and MCL inflation of 1.5 (this affects granularity of clustering). Reciprocal best similarity pairs between species, and reciprocal better similarity pairs within species (*i.e.,* recently arisen paralogs, or "in-paralogs") were added to a similarity matrix. The matrix was normalized by species and subjected to Markov clustering (MCL) to generate orthology groups, including recent in-paralogs. One aspect of the OrthoMCL method that is important to the results is the fact that the program eliminates partial genes from clusters. Thus, short protein sequences that otherwise represent a family, were excluded.

Computational analyses were performed to evaluate the contribution of gene duplications to the complement of *I. scapularis* genes and to explore the possibility of one or more whole-genome duplication events in the evolution of this species. Putative duplicated sequences (paralog pairs) were identified in the *I. scapularis* transcriptome using a method based on that of[37]. Briefly, 20,901 tentative consensus (TC) sequences, produced by alignment of 192,461 *I. scapularis* ESTs, were downloaded from the Dana Faber Cancer Institute – The Gene Index Project (compbio.dfci.harvard.edu/tgi) on February 19, 2008. The program *getorf*[38] was used to identify all possible open reading frames (ORFs) for each TC sequence. The longest ORF for each sequence was selected using *longorf* and Vmatch (http://www.vmatch.de) was used to perform an "all-against-all" nucleotide sequence comparison of each ORF translated in six reading frames. Sequence pairs with at least 75% nucleotide similarity within a predicted open reading frame were identified as candidate paralog pairs.

Predicted protein sequences for *I. scapularis* and other arthropods, as identified by OrthoMCL, are summarized in Supplementary Table 11. This table shows groups of genes clustered based on orthology groups (singletons or duplicates) and unique groups of paralogs. The number of orthology groups found in *I. scapularis* approaches that for insects, while the other two Chelicerate species, *Tetranychus urticae* and *D. variabilis*, have considerably fewer groups. The tabulation of missed orthology groups (OrMis1) is somewhat higher for the Chelicerata, with *I. scapularis* missing the fewest number of groups. This result may be either partly or entirely explained by shorter, partial genes that predominate in the datasets available for species of this clade. By comparing species protein sizes to the median size for each gene family, we found that *I. scapularis* has a -123 amino acid (aa) average difference, and 24% short outliers (2 standard deviations shorter), *T. urticae* has -25 aa, 10% short outliers, and *D. variabilis* has 75% short outliers (note that analyses were based on an artifactually incomplete transcriptome for this species). The Crustacea range from -80 aa to +10 aa average difference from the median, while the Insecta average above the median gene family size. While these results suggest that *I. scapularis* may be missing common gene families, the more likely interpretation is that the tick has fragmented, artifactually short genes, and the same may also be true for *T. urticae.*

Analyses of the *I. scapularis* transcriptome revealed no signatures of large-scale gene duplication or entire genome duplication events. Nucleotide sequence comparison of the longest ORFs corresponding to each of the 20,901 unique *I. scapularis* TCs identified 4,786 putative paralog pairs, suggesting that approximately 22% of *I. scapularis* transcripts are derived from tandemly duplicated genes. This percentage is consistent with estimates of paralog content in the genomes of other organisms. For

reference, paralogs are estimated to comprise approximately 10%, 15% and 20% of the total gene content of the yeast, *Sachharomyses cerevisiae*, *H. sapiens* and the roundworm, *C. elegans*, respectively[39].

An improved *I. scapularis* gene set assembled from RNAseq data is publicly available here: http://arthropods.eugenes.org/EvidentialGene/arthropods/deertick/
A summary document summarizing this improved *I. scapularis* gene set and other arthropod gene sets is available here:
http://arthropods.eugenes.org/EvidentialGene/arthropods/Arthropod_Orthology_Completeness/

Analysis of Repetitive Sequences in the *Ixodes scapularis* Genome

Identification of Tandem Repeats (TRs) in a Small Insert *Ixodes scapularis* Genomic Library

The Tandem Repeats Finder software[40] was used to analyze DNA sequences obtained from end-sequencing of a small-insert *I. scapularis* gDNA library described previously[41] (Supplementary Table 12). Only end-sequences with a sum total of TRs $\geq$100 bp were included. TRs from both the 5' and 3' end sequences for each corresponding clone were summarized together.

Identification and Analysis of Repetitive DNA in the IscaW1 Assembly

Repeat sequences were identified with RECON[12] and RepeatScout[11], and collated into a library that was then used to mask the genome with RepeatMasker[13]. *Ixodes scapularis* Class I and II TEs were identified based on structural features and sequence similarities to other reported TEs (Supplementary Table 13), and are available for download from the TEfam database at: http://tefam.biochem.vt.edu.

Miniature Inverted Terminal Repeats (MITEs). The repeat library IxRepeatlib022908fsa was used to run FINDMITE[42] (no requirement of direct repeat; terminal inverted repeat at 12 bp with no mismatch, and MITE length was set at 100-700 bp). The resulting candidates were used as query to run TEalign, which is a pipeline that runs BLAST against the *I. scapularis* genome, retrieves matching copies plus flanking sequences, and performs clustal alignments. TEalign results were used to manually assess whether each element is a MITE and to classify them, on the basis of clear boundaries shared by multiple copies, terminal inverted repeats, and target site duplications. After obtaining the initial list of MITEs using methods described above, multiple rounds of self-BLAST were performed to remove redundancy using a cut-off of overall 80% identity. The non-redundant MITEs are used as a library to perform RepeatMasker (-div 20). Run RepeatMasker output was used to count MITE copy number and % genome occupancy (Supplementary Table 13). RepeatMakser may overestimate the copy number of elements as one copy may be broken into multiple pieces. Relatively stringent FINDMITE parameters were used for these analyses and it is likely there are additional MITEs await annotation.

LTR Retrotransposons. LTR retrotransposons were identified in the genome assembly and 45 BAC clones using both structure and homology-based approaches (Supplementary Figs. 7-8; Supplementary Table 13). LTR_STRUCT (Version 1.1)[43] allowed the identification at the structural level. For the homology-based approach, the strategy defined by[44] was employed with refinements[45]. Briefly, the canonical

sequences of LTR retrotransposons from several insect genomes were recruited from Repbase[46] and Tefam. TBLASTN[47] was used to search for sequence homologous to the *pol* region of representative LTR retrotransposons in the *I. scapularis* genome. Those hits showing at least 30% amino acid identity over at least 80% of the length of the query sequence were subjected to further analyses to identify both LTRs of each element by means of BLAST2 sequences[48]. This first part of the strategy allowed the identification of canonical sequences representing complete copies that are putatively active and/or consensus sequences corresponding to those constructed after alignment of at least three complete copies of each LTR retrotransposon element in the tick genome. BLASTN searches[47] were then performed using as query each one of the consensus/canonical sequences for each LTR retrotransposon element and providing a list of coordinates of putative each element in the genome. The final criterion used to define two copies as belong to the same LTR retrotransposon element was an identity of 80% or greater at the nucleotide level.

Non-LTR Retrotransposons. Non-LTR transposable elements were identified using a homology-based approach, named TESeeker[49]. To classify the putative TEs obtained from TESeeker, BLASTN searches were performed with each putative TE and the top hit was identified. Next, the longest intact ORF was identified and analyzed using a "classifier." The classifier operates as follows: a library of reverse transcriptase conserved domains (CD)[50, 51] for insect non-LTR retrotransposons was used to classify the ORFs, and, in turn, the original hits. First, the longest ORF of the putative TEs was aligned using MUSCLE[26] to the available CDs for the clade used to generate it. Next, the ORF was trimmed according to the average length of the CD for that particular clade. Only sequences that were at least 95% of the average length of the CD were trimmed and further analyzed. Next, the resulting putative non-LTR was aligned to the entire set of Class I CDs, again using MUSCLE, and an element was inferred from the maximum likelihood tree built from the previous multiple sequence alignment using PhyML[52]. A putative element was considered part of a clade if the branch length for that clade was less than 3.0 and the clade was the closest.

To obtain the representation within the genome, TBLASTN searches were performed using the putative TEs as queries, each of which represented an element within the clade. Hits were counted if they were at least 80% identical to the query and were at least 40% of the query length (shown as "Copy Number" in Supplementary Table 13). Next, to estimate the total genome percent and total base pairs, an assumption was made for each element having intact conserved domains, that the reverse transcriptase was full-length. Knowing the average length of an element for each clade enabled extrapolation of the amount of base pairs for a full-length element, and it is recognized that this may produce an overestimate.

Transposable Element Coding Sequences. A search of the *I. scapularis* genomic DNA for transposable element coding sequences was devised by (1) performing PSI-BLAST of the coding regions of representatives of the diverse families of transposable elements against the non-redundant database from NCBI; (2) constructing matrices from the alignments to be used by the tool RPS-BLAST; (3) retrieving genomic matches by RPS-BLAST against this database that were larger than 500 nucelotides (nt) and with an *e* value < $1e^{-15}$, with an additional 500 nt of flanking regions; (4) identifying terminal repeats (direct and inverted) and trimming the sequences accordingly (sequences without repeats were trimmed on their coding

sequences); (5) clustering the data set of 7,461 elements having 90% identity over 90% of the sequence length to obtain 5,522 clusters of elements, then (6) comparing the consensus sequences to several databases by BLAST, and finally (7) running a program to classify these elements. The data were displayed on a hyperlinked excel spreadsheet from which any element, as well as the corresponding database matches, can be retrieved. The results are summarized in Supplementary Table 14.

Several mariner and piggyBac elements were found containing a full length transposase without stop codon or frame shifts and having inverted repeats. The database is freely available from http://exon.niaid.nih.gov/transcriptome/I_scap_te/Is-te-web.xls and the FASTA file from http://exon.niaid.nih.gov/transcriptome/I_scap_te/is-te-JoseRibeiro-fasta.zip.

Repetitive elements comprise a dynamic component of the coding and non-coding regions of eukaryotic genomes[53,54] (Supplementary Tables 13-14). In addition to the 38 well-represented LTR retrotransposon elements identified in the *Ixodes* genome by means of a homology-based approach, we identified an extra set of 83 lower quality LTR retrotransposon elements in the *I. scapularis* genome assembly and 45 BAC clones by means of LTR_STRUC software, most of which probably correspond to remants of ancient mobilizations. Only 20 out of these 83 elements had intact or well-conserved ORFs that permitted further classification (Supplementary Table 13). The *I. scapularis* genome has a moderate amount of non-LTR retrotransposons (Supplementary Table 13). Most of these non-LTRs are non-functional, and have frame-shift mutations and indels. For those with a complete reverse transcriptase (RT) ORF, necessary for accurate classification, the CR1 clade contributed the most copies to the genome. The fact that a high number of distinct TE families were observed in the relatively young and evolutionarily close CR1 and L2 clades[51,55] may be explained by the lack of a controlling mechanism within the *I. scapularis* genome, which allowed propagation and maintainance within the genome. Unlike other arthropods, the *I. scapularis* genome seems to lack a number of non-LTR clades such as R2, RTE, and LOA that are present in mosquitoes and *Drosophila*[56,57,58,59]. It is possible that these elements may have been present in the *I. scapularis* genome but may have been controlled and degraded, thus preventing their identification. A large number of non-LTR retrotransposons could not be classified to clade due to a low level of conservation and degradation of their RT ORF. For the purpose of this analysis, these elements were grouped into the “unclassified non-LTRs” category.

Arrangement of DNA on the *I. scapularis* Chromosomes

Physical Mapping Using Fluorescence *in situ* hybridization (FISH)

Mitotic chromosomes were obtained from passage 31 of *I. scapularis* cell line ISE18[60,61]. Demecolcine (0.1 µg/ml) was added to the culture for 6-8 h to stop mitosis in metaphase and increase yield of chromosomes spreads for FISH. ISE18 chromosome preparations were held at -20˚C in fixative until use. C_0t -1 DNA was prepared for *I. scapularis* according to previous protocols[62] and used for FISH. Forty-five clones, corresponding to those fully sequenced and assembled herein, were selected from the 10X BAC library and grown in overnight cultures prior to BAC DNA isolation, according to[2]. FISH probes were prepared by labeling BAC DNA with either a biotin- or digoxigenin nick translation mix (Roche Molecular Biochemicals, Indianapolis, IN).

Unincorporated nucleotides were removed from the samples with the QIAquick Nucleotide Removal Kit (Qiagen, Valencia, CA). A small insert (approximately 4 kb) gDNA clone library was prepared from sheared *I. scapularis* egg DNA (Wikel strain) using the TOPO PCR 4.0 cloning vector (Invitrogen, Carlsbad, CA)[41]. End-sequencing of a 384-well plate from this library was conducted at the Purdue University Genomics Core Facility, and the sequences are available at GenBank (Accession numbers GU318418–GU319109). Clones with end sequences comprised of at least 100 bp of tandemly-repetitive DNA, as identified with Tandem Repeats Finder software[40], were selected for FISH experiments (Supplementary Table 12). Clones were grown in 5 ml of LB medium + antibiotic, and plasmid DNA was extracted using the QIAprep spin miniprep kit (Qiagen, Valencia, CA). Plasmid DNA was labeled and used for FISH according to published methods[41]. Probes based on the (TTAGG)n motif used to localize the telomeres were also constructed and the protocol for FISH and imaging processes was carried out as described previously[2,41].

FISH using *I. scapularis* C_0t-1 DNA showed strong hybridization signals to the termini of nearly all chromosomes prepared from ISE18 cells (Fig. 2a). This pattern mirrored that observed with FISH probes for the ISR-2 tandem repeat family (95-99 bp repeat units) and high molecular weight *Hpa*II-insensitive gDNA of *I. scapularis*, also believed to contain these same tandem repeats[41]. FISH using clones containing tandem repeats other than the ISR1-3 tandem repeat families were tested and these experiments showed several examples of tandem repeats that had prominent hybridization patterns dispersed among the presumed euchromatic regions of the chromosomes (Fig. 2c; Supplementary Table 12).

A total of 45 clones from the 10X BAC library, representing those that were completely sequenced and assembled, were hybridized to ISE18 chromosomes (Supplementary Table 15). Fig. 2d-f depicts a representative example of these experiments, where a non-specific hybridization pattern was observed that is thought to reflect repeats dispersed among euchromatic regions of the chromosomes. Note that the terminal regions at one end of nearly all chromosomes are devoid of a hybridization signal to the representative BAC clone shown; this is the area to which the C_0t-1 DNA-fractionated DNA hybridized (as well as the ISR-2 repeats and high molecular weight *Hpa*II-insensitive gDNA of *I. scapularis*) and is thought to represent the centromere. Only three BAC clone hybridizations resulted in specific hybridization signals; these patterns matched that of hybridizations with markers for either the NORs or the ISR-3 tandem repeat family[41].

Analysis of the *I. scapularis* genome for signature telomeric sequences resulted in the discovery of a mixture of (TTAGG)n and (TTAGGG)n motifs in short stretches interrupted by other DNA sequences. This information agreed with previous findings[63], where the (TTAGG)n telomeric motif was characterized by stretches <3 kb in the related tick, *Ixodes ricinus*. This feature of *Ixodes* species is in contrast with that reported in other arthropods typically having (TTAGG)n motifs in stretches of ~20 kb[63]. FISH hybridization of a (TTAGG)n probe to *I. scapularis* chromosomes showed a “two-spot” hybridization pattern at the termini of all sister chromatids of mitotic chromosomes (Fig. 2b)[41]. The position of the telomeric repeats relative to the nearly adjacent centromeric heterochromatin supports a telocentric (or acrocentric) chromosome structure, consistent with the original description of ISE18 chromosomes[61].

An ideogram (Fig. 2g) of *I. scapularis* chromosomes (2N=28 with an XX, XY sex determination system) was constructed based on the relative hybridization patterns of several tandem repeats to mitotic chromosomes prepared from cell line ISE18[41,61]. These repeats include a telomeric (TTAGG)n motif, the nucleolar organizing regions (NORs), and major repeat families ISR-1, ISR-2a, ISR-2b, and ISR-3[41]. Physical mapping of these markers provided a basis to distinguish individual as well as several different groups of chromosomes. Those that can be readily distinguished include the sex chromosomes X (the largest) and Y (the smallest), as well as three pairs of chromosomes that hybridize to only ISR-1, ISR-2a, and ISR-2a + ISR-3, respectively. Also, an additional pair of chromosomes can be identified based on hybridization to ISR-2a over approximately half the entire chromosome. The other chromosomes in the karyotype were grouped according to their hybridization signals to these markers, but could not be reliably paired or distinguished from similar chromosomes. These groups include those that show signals for ISR-2a + NOR (4 chromosomes), ISR-1+ISR2a (4 chromosomes), and the remaining chromosomes that hybridize only to ISR-2a (10 chromosomes), respectively. This ideogram representing the current *I. scapularis* physical map serves as an anchor to position additional FISH markers as they are further developed.

***Ixodes scapularis* Genes and Gene Families**

The *Ixodes scapularis* Sialome

The saliva of blood sucking arthropods consists of a complex mixture of peptidic and non-peptidic compounds that disarm their hosts' hemostasis, inflammation and immunity, thus helping blood feeding. Antimicrobial compounds are also commonly found, and these may protect the ingested meal from bacterial overgrowth, as well as protecting the feeding lesion in the case of hard ticks. While hematophagous insects have near one hundred salivary polypeptides identified from transcriptome analysis, saliva of hard ticks may contain several hundred polypeptides. Comparative transcriptome analysis of related arthropods indicates that salivary gland gene products are evolving at a fast pace, perhaps due to the immune pressure imposed by their hosts. Indeed, while salivary peptides can belong to ubiquitous protein families, unique salivary protein families are found at a genus and even subgenus level. These unique families probably derive from a gene common to the family or order ancestor but rendered unrecognizable by divergent evolution[64,65]. Gene duplications are commonly associated with salivary genes, even within insects having relatively compact genomes, such as the mosquito *An. gambiae* (~278 Mb, three pairs of chromosomes)[54], where the uniquely Nematoceran D7 family consists of eight genes, and the uniquely anopheline G1 protein family has six genes[66]. In insects with larger genomes, or perhaps more importantly, larger number of chromosomes, such as the kissing bug *Rhodnius prolixus* (~600 Mb, 11 pairs of chromosomes)[67], dozens of gene products coding for salivary lipocalins have been described, and are possibly derived from both gene duplication and genome duplication events[68,69]. In *I. scapularis* (~2.1 Gb, 14 chromosome pairs) [1,61], a large expansion of the lipocalin family (associated with anti-complement and anti-inflammatory activities), as well as proteins containing Kunitz domains (associated with serine protease inhibitory activity as well as channel blockers, functioning as anti-clotting and possibly as anesthetics or vasodilators)[70,71,72,73] were identified, in addition

to other gene expansions for numerous unique protein families[74,75]. Sialotranscriptome analysis based on ~8,000 ESTs from nymphs and adults at different stages of feeding led to the identification of 26 different groups of proteins (not including housekeeping proteins)[74]. Of these 26 families, 16 are either unique to ticks, or found only in the genus *Ixodes*, based on available sequence data[64]. When the deducted protein sequences were compared within a family, and a smaller than 90% sequence identity was used as a threshold level, 197 sequences were identified as possibly derived from individual genes (Supplementary Table 16); more closely related sequences are possible alleles or may derive from conserved gene duplication events. The large amounts of gene duplicates may provide a mechanism for antigenic variation, by differential expression of genes during the feeding process, as observed for *I. scapularis* cystatins[76], while polymorphism may be maintained by frequency dependent selection of antigenic epitopes[74].

The availability of the draft genome of *I. scapularis* allows for verification of these salivary gene expansions and provides a platform for determining temporal and tissue specificity of these genes. In particular, it provides evidence for the large expansion of proteins with Kunitz domains, as well as for the apparent lack of genomic evidence for the expansion of unique protein families, such as the WC-10 family, or the anti-complement ISAC family.

Kunitz-domain family. Seventy-four of the 20,452 annotated tick proteins possess one or more Kunitz domains (Supplementary Table 16), making the tick genome the richest source of proteins with this domain. Only 25 of the 46,704 human proteins, or 33 of the 26,255 bovine proteins have this signature as revealed by the KU Smart signature[77] (Ensembl Proteome sets obtained at 7/31/2008). For comparison with insect proteomes the mosquitoes *Aedes aegypti, Culex quinquefasciatus* and *An. gambiae* have five, eight and four proteins respectively, with Kunitz domains (mosquito proteomes obtained from VectorBase in Dec/2009). Interestingly, no Kunitz domain-containing proteins were found in sialotranscriptomes of these three mosquitoes, but they occur in the sialomes of *Culicoides*[78,79] and *Simulium*[80], indicating a case of convergent evolution in the salivary recruitment of genes to assist blood feeding. Two *I. scapularis* proteins, Ixolaris and Penthalaris, containing two and five Kunitz domains, respectively, have been functionally characterized as potent inhibitors of the extrinsic pathway of blood clotting[81,82]. It is possible that this large family contain also channel blockers with toxic or vasodilatory properties, as recently identified for a Kunitz protein from a metastriate tick[73].

WC-10 and Isac families. The WC-10 protein family codes for mature proteins with masses near 10 kDa and a tryptophan-cysteine dipeptide motif at their carboxyterminus. Their function is unknown. Twenty-one members of the WC-10 family were identified in previous sialotranscriptome studies, but only four such proteins are found in the deducted tick proteome (Supplementary Table 16). Inspection of shotgun sequences indicates that some additional members of this family may be found, but not all. Similarly, four members of the Isac family of anticomplement proteins have been described, but only one protein of this family is found in the deducted proteome, coding for a protein that is only 65% identical to previously reported anticomplement proteins. Shotgun sequences, however, are found that code for three of the Isac proteins, indicating these may not have assembled into the genomic scaffolds. On the other hand, tick salivary proteins may be under strong evolutionary pressure imposed by their

host's immunity and thus may differ among geographical strains, which differed between the salivary EST and genome sequencing sets.

Ixodes scapularis Innate Immunity/Tick-Pathogen Interactions

Computational analysis to identify putative immune-related genes within the *I. scapularis* genome was performed using information available in GenBank[83], VectorBase[84,85], Ensembl[86], and OrthoDB[25]. An extensive BLAST search (default parameters) was performed to identify sequences sharing homology with previously identified members from *D. melanogaster*, *An. gambiae* and *Ae. aegypti*. When multiple similar sequences were available for BLAST search, the longest isoform was used as a query. Sequences were then analyzed within Ensembl, OrthoDB and VectorBase to address gene prediction as orthologues and/or paralogues. Proteins sequences were also retrieved based on lists of significant BLASTp hits, and analyzed using Pfam[87] and PROSITE[88] for conserved domain identification. The results illustrated here (Supplementary Figs. 22-23) correspond to sequences obtained as orthologues for *I. scapularis* following subsequent manual curation. Retrieved *I. scapularis* sequences were further analyzed using PROSITE and the Conserved Domain Database for JAK-STAT domain identification[89].

Toll pathway. Our *in silico* approach identified four protein sequences annotated as peptidoglycan recognition receptors (PGRPs) (Supplementary Table 17; Supplementary Fig. 22a). However, our group did not assign a function to these genes, as PGRP isoforms may be categorized either in the Toll or the IMD pathways. We did not identify any Gram-negative binding protein (GNBPs). All bioinformatics comparisons using *Drosophila* GNBP1 or 3 as a query against the *I. scapularis* genome yield high *e*-values and no apparent functional correlation. Spaetzle processing enzyme (SPE) is a CLIP domain-containing serine protease. Multiple sequences could be found carrying CLIP and trypsin-like serine protease domains in the *I. scapularis* genome. However, their precise role is unclear. Modular serine protease (ModSP) and Grass leads to SPE cleavage. ModSP carries four low-density lipoprotein-receptor class A domains and a complement control protein (CCP) module. We did not identify any sequences carrying both domains. Grass, which shows a trypsin-like serine protease characteristic domain, shares similarity with several secreted salivary gland peptides (*e*-values < $1e^{-45}$). However, further studies are needed to properly identify a precise Grass and persephone counterpart in *I. scapularis*. We identified ten Toll sequences in the *I. scapularis* genome. Five of these sequences encode for either the characteristic Toll/Interleukin-1 receptor (TIR) or Leucine Rich Repeats (LRR) domains, but not both. An *I. scapularis* homologue of the adaptor molecule myd88 was uncovered, as well as homologues containing Death domains (DD) characteristic of the Pelle-Tube complex. We have also identified an embryonic polarity Dorsal homologue and a Cactus-like inhibitor of IκB carrying ankyrin repeats. Similar to what has been described in mosquitoes[90,91] we did not observe any homologue of the NF-κB factor dorsal-related immunity factor (DIF).

IMD pathway. Our *in silico* approach failed to identify a significant number of molecules involved in the IMD pathway (Supplementary Table 17; Supplementary Fig. 22b). Diaminopimelic (DAP)-type peptidocglycan (PGN) recognition leads to intracellular signaling through the adaptor molecule IMD, a DD-containing adaptor molecule that interacts with the PGRP receptors and triggers association of Fas-associated protein

with DD (FADD). We did not observe any IMD or FADD homologues in the *I. scapularis* genome. These results can be explained by either a high degree of gene dissimilarity between species (*i.e.*, IMD was also not identified in the louse[92] and pea aphid genome[93]), or these sequences were not represented during *I. scapularis* genome assembly). Furthermore, the large evolutionary distance between ticks and dipteran insects made it challenging to uncover genes using homology-based methods. By searching the *I. scapularis* genome for DREDD-like caspases, we uncovered six caspases, four of which are annotated as “caspases” in VectorBase and Genbank. Two other sequences were also identified but are annotated as caspase-2 and 3. The cleavage of IMD exposes an inhibitor of apoptosis binding motif to allow recruitment of inhibitor of apoptosis proteins 2 (IAP2). We uncovered an IAP2 homolog in *I. scapularis*. In *Drosophila*, DIAP2 interacts with IMD and leads to IMD K63-ubiquitination. This ubiquitination involves Uev1a, Ubc13 (also known as Bendless) and Ubc5, or Effete. Our analysis indicated that these enzymes are highly conversed in the *I. scapularis* genome. Polyubiquitination of IMD seems to be essential for recruitment activation of the downstream Transforming growth factor β–activated kinase 1 (TAK1) and the IκB kinase (IKK) complex, as well as for binding of TAB2 (TAK1-binding protein 2). We identified *I. scapularis* homologues of TAK1, TAB2 and the IKK complex. Once IKK complex is activated by TAK1, it phosphorylates relish, a bipartite NF-κB protein that has both a Rel homology domain and IκB-like ankyrin repeats. A relish orthologue was successfully uncovered in the *I. scapularis* genome. Similarly, the negative regulators Plenty of SH3 domains (POSH), Caspar and Caudal were also observed in the *I. scapularis*. Recently, akirins have emerged as another nuclear factor regulating immune responses in parallel with NF-κB in mice and in the context of the IMD pathway in *Drosophila*[94]. We have also identified an akirin homologue in *I. scapularis* – subolesin[95].

JAK/STAT. Candidate orthologues for all three core members of the JAK-STAT pathway (*e.g.*, receptor, JAK kinase, and STAT activator) were identified along with putative orthologues for the following regulators: suppressor of cytokine signaling (SOCS) and protein inhibitor of activated STAT (PIAS) (Supplementary Table 17; Supplementary Fig. 23).

RNAi pathway. The RNAi pathway is found in many eukaryotes[96,97]. Generally, the RNAi pathway can be categorized in two main signaling cascades: the siRNA (short-interfering) and the miRNA (micro) networks. The siRNA pathway is activated in response to endogenous or exogenous dsRNAs (double stranded) and has been associated with defense against viruses and transposable elements. Conversely, the miRNA cascade is only activated in response to endogenous dsRNA and differences in target mRNA complementarity may affect the final post-transcriptional gene silencing (*i.e.*, mRNA cleavage or translation arrest)[98,99,100,101]. In the *Drosophila* siRNA pathway, the RNaseIII-like Dicer-2 enzyme cleaves a long dsRNA into a small 20-25bp dsRNA molecule. R2D2, a RNA-binding protein, interacts with Dicer-2 to promote loading of a now single-stranded siRNA into a RNA-inducible complex (RISC). A major component of RISC is the RNase-H enzyme Argonaute, which degrades the target mRNA, complementary to the sequence encoded by the antisense siRNA, and promotes gene silencing. We have identified two Dicer homologues in the *I. scapularis* genome (Supplementary Fig. 23b). We did not identify a homologue for R2D2. However, five sequences sharing homology with Argonaute were discovered in the genome. Recent studies have indicated the RNAi antiviral response is extremely complex in

invertebrates, and an increasing number of molecules have been implicated in this pathway, controlling production of a range of virus-derived small RNAs. A list of other *I. scapularis* homologues is provided in Supplementary Table 17.

Other immune-related genes. We identified homologues of several immune-related gene in the *I. scapularis* genome (Supplementary Table 17) but the precise pathway controlling their expression cannot be predicted solely by comparative genomics. Differential expression of antimicrobial peptides (AMPs) after infection, particularly, corresponds to a key component of immunity in *Drosophila* and mosquitoes. While *Drosophila* has seven AMP families, each one having several members, we identified only defensins and defensin-like molecules in *I. scapularis*. In mosquitoes, families of defensins and cecropins are the most predominant AMPs and they are represented by multiple members[102]. In a more extreme case, extensive searches in the pea aphid genome failed to identify any AMPs[93]. Our bioinformatics analysis confirmed the presence of genes previously annotated as AMPs: defensin, scapularisin, microplusin and two unnamed AMPs. Based on a more robust computational approach, a recent publication has suggested an expansion of the defensin family in *I. scapularis* genome[103]. We were unable to find in the *I. scapularis* genome any gene sequences sharing similarity with attacin, diptericin, drosocin, drosomycin or cecropin. Other important homologues uncovered include the enzymes Dual and NADPH oxidases, which control production of reactive oxygen species, and lysozymes, fibrinogen-related and thio-ester containing proteins, all of which contribute to the immunological process upon microbial infection.

Ixodes scapularis Mevalonate-Farnesal Pathway Genes

A BLASTX and BLASTN search of the *I. scapularis* genome for the insect enzymes involved in the synthesis of juvenile hormone (JH) III revealed the presence of all but two of the enzymes involved in the farnesyl-PP pathway (Supplementary Fig. 18; Supplementary Table 18). The genes found were acetoacetyl-CoA thiolase, hydroxymethylglutaryl-CoA synthase, hydroxymethylglutaryl-CoA reductase, mevalonate kinase, phosphomevalonate kinase, diphosphomevalonate decarboxylase and farnesyl diphosphate synthase. Shown are the *I. scapularis* supercontig numbers and gene accession numbers. The top insect BLAST results from these *I. scapularis* messages had *e*-values ranging from $1e^{-44}$ to 0.0. Isopentenyl diphosphate isomerase and geranyl diphosphate synthase were not found. Transcripts for all but two of the enzymes involved in this pathway have been found in the adult synganglion transcriptomes of the hard ticks, *I. scapularis* and the American dog tick, *D. variabilis*, and only one missing from the soft tick, *Ornithodoros turicata*. In the insect JH III branch (Supplementary Fig. 18), only two enzymes were found in the *I. scapularis* genome, farnesol oxidase and methyl transferase (MT), the former also found in the *I. scapularis* and *D. variabilis* synganglion transcriptomes and MT in all three synganlion transcriptomes. The farnesol oxidase transcript has the classic SDR family motif and shares 60% identity with the pollinating wasp, *Ceratosolen solmsi marchali* (e-value, $1e^{-99}$). MT with a top BLAST hit for JH MT from the insect, *Schistocerca gregaria* (*e*-value, $4e^{-18}$) was found (Supplementary Table 18). Whether this enzyme functions as a JH MT in ticks is not known. There has been a large expansion of the MT gene family (Supplementary Fig. 19). It appears the MTs in *I. scapularis* examined so far do not have a JH binding domain. Farnesyl diphosphate pyrophosphatase, farnesal

dehydrogenase and JH epoxidase were not found. JH epoxidase in insects in the P450 family CYP15A1 is responsible for the addition of the C10-11 epoxide to methyl farnesoate to produce JH III; this family of P450s was not identified in the *I. scapularis* genome.

Biochemical studies of tissue extracts further support the hypothesis that ticks lack JH. In published work[104], radio HPLC was unable to detect methyl farnesoate, JH I, JH II, JH III, or JH III bisepoxide in different tissues, including the synganglion of the soft tick (*Ornithodoros parkeri*) and the hard tick (*D. variabilis*) at different stages of development; the lower detection limit for JH and methyl farnesoate in these studies in the synganglion was 1.3 fmol for 10-tick equivalents in a 3 hour incubation. In the same study, no JH I, JH II, JH III, JH III bisepoxide, or methyl farnesoate was detected in adult hemolymph at the time of egg development in the same ticks as determined by EI GC-MS; the MS sensitivity was 1.6 pg in the scan mode from 40 to 300 AMU and 750 fg in the SIM mode for fragments at m/z 76 and 225. The same study failed to identify any lipid soluble material from whole body extracts of eggs, larvae, nymphs and adults of *D. variabilis* that would result in the retention of larval characters in the *Galleria* moth bioassay. The lower detection limits for eggs, larvae and nymphs were 28 pg for JH I and JH II and 980 pg JH III per g of tick tissue. For adults, the detection limits were 116 pg for JH I and JH II and 4069 pg JH III per g tissue. To date, JH has only been found in insects and only methyl farnesoate in the sister group to insects, the Crustacea. Finally, published work[105] does not support the hypothesis that ticks regulate egg development via JH[106] in *D. variabilis*; ecdysteroids initiated the synthesis of vitellogenin in *D. variabilis* but not JH III. Most evidence to date suggests that JH is not produced in ticks and that JH is not involved in tick metamorphosis and reproduction. The discovery of most of the farnesyl-PP (mevalonate) pathway and two enzymes, farnesol oxidase and methyl transferase, in the farnesal (insect JH) branch in both the *I. scapularis* geneome and adult syngnalion transcriptomes studied suggest these pathways are involved in reproduction at least and warrants future research in the potential role of these enzymes in the endocrinology and regulation of tick development.

Ixodes scapularis **Heme Synthesis and Storage Protein Genes**

To identify genes coding for enzymes in the heme pathway, heme biosynthesis genes from a range of animals, fungi and prokaryotes, including multiple *Rickettsia* species were used in TBLASTX similarity searches of the *I. scapularis* assembly (ABJB010000000) and trace files and the REIS assembly[10]. Genes were manually annotated using Artemis software (v.11, Sanger Wellcome Trust) (Supplementary Table 20). To provide further support for functional predictions additional curation of each gene model was facilitated based on E.C. number. Putative hemelipoglyco-carrier protein (CP) genes were identified via TBLASTN search of the *I. scapularis* ISCW1 assembly at VectorBase using sequences from the tick, *D. variabilis*[107] and other invertebrates (Supplementary Table 22). Gene models were manually annotated using Artemis software v.8[108] and corresponding accession numbers were identified, where possible.

Adaptation to hematophagy has developed multiple times within the Arthropoda and even within a particular group such as the Diptera[109,110]. Despite the abundance of heme from the host hemoglobin, triatomine bugs (Order Hemiptera: Family Triatominae) apparently have the ability to synthesize heme as evident by the functional expression

of delta-aminolevulinic acid dehydratase, the rate limiting enzyme in the heme biosynthetic pathway[111]. However, investigators were unable to demonstrate heme biosynthesis in the southern cattle tick, *Rhipicephalus microplus*[111]. Several steps in the heme biosynthesis pathway were found in the *I. scapularis* genome (Supplementary Fig. 15; Supplementary Table 20). In the light of these findings, the question of the role of heme biosynthesis enzymes in the processing of host blood versus *de novo* heme synthesis should be re-examined. In addition, the importance of these processes compared to heme sequestration by unique heme-binding proteins in ticks as described below, requires further evaluation.

An important adaptation that co-evolved with blood feeding is heme sequestration by heme-binding proteins along with heme excretion, both of which prevent oxidative stress and tissue damage. Free heme results in reactive oxygen that leads to lipid peroxidation and cytotoxicity[112]. Heme is also important as a prosthetic group for respiration, enzymatic detoxification and oxygen transport[113]. In *Rhodnius prolixus*, host hemoglobin is digested to free heme which is then absorbed into the hemolymph and sequestered by a 15-kDa heme-binding protein (RHBP), reducing lipid peroxidation[114,115]. Other heme-binding proteins present in *R. prolixus* include nitrophorins for nitric oxide transport[116] and which have been implicated in host vasodilation during blood feeding. This suggests multiple uses for heme and heme binding proteins in blood feeding insects and possibly in other organisms like ticks.

Two storage proteins are found in tick hemolymph, a heme lipocarrier protein (CP) and the yolk protein (Vg), which share a common evolutionary origin[107,117] . These proteins have similar structural motifs that include the LPD_N, the C-terminus vWD, the unknown function DUF1943 domain, cleavage sites (RXXR) and the GL/ICG domain (Supplementary Fig. 16). CP in hard ticks is found in both sexes and in all developmental stages and tissues studied. All CPs studied are composed of two subunits, 92 and ~100 kDa. Research suggests that the main source of CP mRNA in *D. variabilis* is the fat body and the salivary gland[107]. They also showed that host attachment and blood feeding initiated CP expression in virgin females while mating and feeding to repletion reduced the level of CP protein. Potentially, 10 CPs were found in the genome of *I. scapularis* (Supplementary Table 22), although all but one are incomplete gene models. This is by the far the greatest number of CPs found from a single tick species. It is not clear whether these genes are expressed, and if so, the importance of their protein products in tick physiology.

The regulation of full-length yolk protein messages was studied in the hard tick, *D. variabilis*. Studies showed that DvVg1 and DvVg2 are exclusively expressed in females after mating and feeding to repletion and are up-regulated by ecdysteroids not JH III. Both Vgs are not expressed in males (fed and unfed) or females before mating and feeding to repletion. The main source for DvVg1 and DvVg2 is the fat body and the gut cells. In the soft tick, *O. moubata*, studies have shown that the source of OmVg is the fat body and the gut and is regulated by ecdysteroids similar to the case in *D. variabilis*[118]. The same study observed a major difference between *D. variabilis* and *O. moubata,* where in the latter, Vg expression was initiated by engorgement in both virgin and mated females but increased further in mated females.

Multiple incomplete CP gene models and two Vg genes were identified in the genome of *I. scapularis* (Supplementary Table 22). The alignment of these sequences with homologous sequences from *D. variabilis* is shown in Supplementary Fig. 16. The

conceptual CP proteins are similar in amino acid length and have the characteristic domains (LPD_N, DUF1943, vWD, RXXR and GLCG). The N-terminus sequence for the small subunit is FEVGKEYVY which is 100% identical to that determined for the *R. microplus* CP[119]. This sequence is directly downstream from the secretion signal and marks the start of the LPD_N domain. The N-terminus of the larger subunit is DASAKERKEIED which has high sequence similarity to the *R. microplus* CP[119] and exists directly downstream from the only predicted cleavage site. The tick Vg genes contain three domains (LPD_N, DUF1943 and vWD). Additionally, the RXXR cleavage site may be absent, as is the case for the *I. scapularis* Vgs, or variable in number and locations as observed for Vgs from other tick species. In ticks, Vg proteins typically consist of several subunits with variable N-terminus sequences while CPs consist of two subunits produced by only one RXXR cleavage site. We also found that all tick Vgs have an amino acid spacer (10-20 amino acids) between the secretion signal and the LPD_N which does not exist in CPs. The high level of sequence similarity observed between tick CPs and Vgs complicates the characterization of these molecules.

Ixodes scapularis Blood Digestion Genes

Unlike most other blood feeding arthropods, ticks digest the protein contents of a blood meal intracellularly in the epithelial cells of the midgut. Hemoglobin liberated from hemolyzed erythrocytes binds to clathrin-coated pits on the luminal sides of the midgut epithelial cells and is internalized by pinocytosis into large (3-12 µm) endosomes (Fig 1D). Once inside the epithelial cells, the endosomes fuse with lysosomes to form specialized digestive vesicles. All hemoglobin digestion occurs intracellularly in these digestive vesicles and is carried out by a cascade of proteolytic enzymes, most functioning at acidic pH (3.5-4.5 pH, the pH optimum of the digestive vesicles). These enzymes selectively target different sites on the globin moieties, ending in dipeptides and free amino acids. The enzymatic steps previously described for *Ixodes ricinus*[120] are believed to be the same or similar in *I. scapularis,* since the same enzymes occur in the *I. scapularis* genome (Supplementary Table 21). Similar hemoglobinolytic enzymes have been found in other tick species[121,122], indicating that this novel mode of hemoglobin digestion is widespread throughout the Ixodida.

Digestion of the globin moieties is initiated by the aspartic protease cathepsin D (the major hemoglobinase), assisted by the cysteine class endopeptidases cathepsin L and legumain. The action of these enzymes liberates heme and large (approximately 8 – 11 kDa) peptides peptide fragments. In the next stage of the process, the large peptides are digested further by the cysteine amino cathepsin B and the cysteine carboxypeptidase cathepsin L, cleaving them further into smaller fragments, ~5-7 kDa. The third stage in the digestive process is carried by cathepsin C, assisted by Cathepsin B, resulting in small (approximately 3-5 kDa) peptides. The final stage in the process is completed by serine carboxpeptidases (SCP) and leucine aminopeptidases (LAP) resulting in dipeptides and free amino acids. The latter are transcytosed from the digestive cells into hemolymph. Heme liberated from the digestion of the parent molecule is transported from the digestive vesicles by heme-binding proteins to hemosomes, unique storage vesicles where the heme is detoxified by forming unique hematin-like aggregates[123].

Hemoglobinolysis in ticks shows greater similarity to the enzymatic pathway in endoparasitic flatworms and nematodes than to blood feeding insects, although ticks are unique in carrying it out intracellularly within digestive vesicles of the midgut epithelium[120,124,125].

Ixodes scapularis **Metabolic Detoxification Genes**

Ixodes scapularis cytochrome P450 (CYP450) annotations (Supplementary Table 23) were produced from the JCVI version 0.5 (133 sequence pieces) and VectorBase version 0.5 (195 sequence pieces) gene model predictions. BLAST comparison of these two gene model sets was used to produce a set of 223 unique CYP450 sequences. DNA sequence for each P450 was recovered from the WGS section of NCBI and each gene was assembled manually based on comparison to the closest matches from other tick, mite and insect CYP450 sequences. EST searches were also used to confirm intron-exon boundaries and to extend partial gene models. Phylogenetic trees were constructed with the most closely related sequences to assign CYP names based on established CYP nomenclature. Comparison of Ixodes P450s to *Tetranychus urticae* showed only *Halloween* gene families CYP302, CYP307, CYP314, CYP315 and the 26-hydroxylase that degrades ecdysteroids CYP18 are conserved (Supplementary Fig. 17). CYP306 is missing in both species. Putative carboxylesterase (EC 3.1) and acetylcholinesterase (AChE)-like (EC. 3.1.1.7/3.1.1.8) genes were identified in the *I. scapularis* genome seqeunce by TBLASTN search of scaffolds at NCBI (Supplementary Table 24). Gene models were manually annotated using Artemis v.8[108] and the putative function of conceptual protein sequences was predicted based on protein sequence homology to invertebrate and vertebrate protein sequences. To identify divergent members of the carboxylesterase gene family, reciprocal TBLASTN searches were conducted against the ISCW1.1 assembly using the predicted *I. scapularis* carboylesterase and AChE-like protein sequences.

Two hundred and six CYP genes and six pseudogenes were identified in the *I.scapularis* genome (Supplementary Table 23). Ninety-one additional fragments were also identified that were too short to name; some of these fragments may represent pseudogenes. This finding represents the largest number of CYP genes identified in any animal to date. The *I. scapularis* CYP18, CYP302, CYP307, CYP314 and CYP315 gene products may be involved in ecdysteroid metabolism, based on the function of orthologous genes in other invertebrates. The function of the remaining *I. scapularis* P450s is unclear. By comparison, the body louse, *P. humanus*, which like *I. scapularis* is also exclusively hematophagous, has only 36 CYP genes. It is unlikely that the large number of *I. scapularis* P450 genes reflects a need to detoxify blood components such as heme. One possible explanation for the expanded number of CYP450s in *I. scapularis* is exposure to plant toxins secreted as oils by plant trichomes. *Ixodes scapularis* spends much of its life cycle off host and may be exposed to a wide variety of plant chemicals, especially as it exploits vegetation in order to locate and transfer to its animal hosts.

A total of 75 putative carboxylesterase/AChE-like genes, 11 putative pyrethroid metabolizing carboxylesterases with sequence similarity to the *R. microplus* CzEST9 gene which is associated with pyrethroid resistance in the cattle tick[126], and two putative juvenile hormone esterases were identified in the *I. scapularis* assembly (Supplementary Table 24). Analyses suggest that the majority of these gene models

represent complete or near complete CDS. However, some sequences listed in Supplementary Table 24 likely represent one or more exons of incomplete gene models. Further annotation, coupled with wet lab analyses will ultimately resolve the final number of carboxylesterase-like genes in the tick. Of note, many members of the carboxylesterase-like gene family are located on the same scaffold, with two extreme cases being scaffolds DS818569 and DS921995, both of which contain ten putative carboxylesterase gene models. This finding suggests significant tandem duplications, a phenomenon commonly associated with this gene family.

Ixodes scapularis **Neuropeptide Genes**

Identification of the neuropeptide genes was based on Blast searches utilizing gene sequences available in VectorBase. Where possible, additional evidence for some of these neuropeptides derived from transcriptomes; immunohistochemistry data for other ixodid tick species was also included, further supporting their functional assignment.

A search of the *I. scapularis* genome for neuropeptides and neuropeptide receptors of the classical invertebrate neuroendocrine system revealed the presence of at least 39 canonical neuropeptide genes (Supplementary Tables 25-28). Twelve additional novel putative neuropeptide genes were identified from their tandem repeats with conserved C-terminal sequences including the canonical sequences for amidation and dibasic (or monobasic) cleavage signals (Supplementary Table 25). Canonical predicted neuropeptides include multiple allatostatins, myoinhibitory peptides, allatotropin, bursicon α, bursicon β, crustacean cardioactive peptide, CCH, corazonin, diuretic hormone, FMRFamides, eclosion hormone, glycoprotein hormone α/β, insulin-like peptide, neuroparsin (insulin-like growth factor binding protein or IGFBP), ion-transport peptide, orcokinin, sulfakinin, prothoracicotropic hormone (PTTH)-like hormone, proctolin, pyrokinins, periviscerokinin, SIFamide and tachykinin.

Ticks are chelicerates, a subphylum that evolved more than 500 million years ago[127], and are evolutionarily distinct from the insects and crustacea. Ixodid ticks are unique among blood feeding arthropods in their ability to feed for long periods, create additional cuticle to accommodate enormous blood meals, and remove excess blood meal water via their salivary glands. Blood feeding also stimulates development and reproductive functions. Here we review genes for neuropeptides believed essential to these processes. Among the most abundant of these neurohormones is allatostatin (Type A). The gene ISCW022937, a likely ortholog of the cockroach allatostatin precursor (AAC72892), was found in the tick genome database, but its function has not been determined. Three copies of the gene for an allatostatin receptor were also identified. Allatotropin and allatostatins regulate production of juvenile hormone (JH) in insects and may have additional functions as well; however, there is no conclusive evidence of JH in ticks[104]. Consequently, the function of these peptide hormones and/or their receptors in ticks is enigmatic. Evidence of allatostatin mRNA was found in the synganglion of the dog tick, *D. variabilis*[128] and *I. scapularis*[129], suggesting that this hormone and its receptor may be conserved throughout the Ixodida. The gene for allatotropin was found in the *I. scapularis* genome and evidence of a transcript predicting its occurrence in the synganglion of adult *I. scapularis* was reported[129] and also demonstrated by immunohistochemistry in *Rhipicephalus appendiculatus*[130]. These peptides may also have other regulatory functions. In insects, allatotropin was shown to

stimulate the foregut muscles, whereas allatostatin was found to inhibit contractions of the foregut, and, as a result, suppressed feeding activity[131]. Consequently, the role of these genes in *I. scapularis* awaits further biochemical and molecular studies.

Genes associated with the ecdysial process were found, including corazonin, eclosion hormone, CCAP, and bursicon (α and β). In addition to the complete gene model of corazonin in the *I. scapularis* genome, ESTs matching corazonin and the corazonin receptor were identified in an unpublished synganglion cDNA library from adult female *D. variabilis*[128]; and this neuropeptide was also detected in unfed adult female *R. appendiculatus* by immunohistochemistry[130]. Similarly, a match for eclosion hormone (ISCW001941) to a conserved hypothetical *I. scapularis* protein (NCBI XM_002399230) was found. Expression of these hormones and/or hormone receptors was reported in adult female *D. variabilis* by 454 pyrosequencing[128]. Genes for both bursicon α and bursicon β were identified. Transcripts for both bursicon subunits were also found in the synganglion of feeding adult female *D. variabilis*[128]. Bursicon is an approximately 30 kDa, highly conserved molecule in insects where it functions in wing expansion (in *Drosophila*) and as a cuticle-hardening (tanning hormone) regulator[132]. Although adult female ixodid ticks do not molt again after nymphal eclosion, they do secrete new cuticle during feeding and it is likely that these genes contribute to hormonal regulation of cuticle hardening and tanning.

Insulin-like peptide (ILP), a member of the insulin superfamily, is a highly conserved gene that is widespread among multiple taxa. Following transcription, it is translated as a preprohormone. In insects, following cleavage of the signal peptide, the mature proteins containing the characteristic A, B, and C-chain peptides are stored in secretory granules. Subsequently, the C peptide is removed by convertase. Genes for preproconvertase (ISCW020499) and IGFBP (ISCW003285) were found in the *I. scapularis* genome, suggesting the existence of an insulin signaling pathway. Insulin-like signaling activity is believed to regulate development, longevity, metabolism, and female reproduction[133] as well as ecdysteroidogenesis[134]. Silencing IGFBP (by RNA interference) prevented blood-feeding females from feeding to repletion, indicating the role of this protein in regulating feeding in ticks[135]. ILP mRNA was found in the transcriptome of the female *D. variabilis* synganglion and ILP immunoreactivity has been identified in other tick species[130,136]. ILP is believed to be secreted from neurosecretory sites in the periganglionic sheath into the periganglionic sinus and thereupon into general circulation.

Orcokinins and sulfakinins are believed to be important in regulating contractions of the digestive tract in insects and are likely to play a similar role in *I. scapularis*. Orcokinins increase gut contractions, presumably enhancing feeding activity, whereas sulfakinins inhibit feeding activity. At least one orcokinin gene and two sulfakinin isoforms were identified in the genome. Transcripts of four orcokinins, a preprosulfakinin and a sulfakinin receptor were found in the transcriptome of the female *D. variabilis* synganglion[128]. Sulfakinins show homology to cholecystokinins, which are believed to function as satiety inducing peptides[137]. We hypothesize that the sequential up or down regulation of these genes following mating induces rapid blood feeding to repletion.

Several genes were found that are important in regulating salivary gland function. In addition to dopamine, long known as a secretory agonist[138], myoinhibitory peptide (allatostatin B) and SIFamide peptide were identified in the *I. scapularis* genome. These peptides were also identified in neurosecretory cells and their axonal projections leading

to the salivary glands by immunohistochemistry indicating their importance in regulating the function of these glands[139].

Several other neuropeptides have been identified in *I. scapularis*, *e.g.,* allatostatin-C, proctolin, pyrokinin-2, pyrokinin-3, pyrokinin-4, and periviscerokinin[140,141]. In addition, periviscerokinin was identified in *I. ricinus* and *R. microplus* by MALDI-TOF/TOF mass spectrometry[142].

Ixodes scapularis G-protein Coupled Receptor (GPCR) Genes

Putative *I. scapularis* GPCRs were identified by TBLASTN searches of the tick genome assembly at VectorBase (https://www.vectorbase.org/). The primary source of query sequences included GPCRs from the mosquitoes *An. gambiae*[143] and *Ae. aegypti*[144] and the fruitfly *D. melanogaster* (FlyBase; http://flybase.org/), while additional invertebrate and vertebrate GPCR sequences were used when appropriate. Identified GPCRs were used to iteratively search the *I. scapularis* genome for additional GPCR sequences. Alignments of conceptual GPCR amino acid sequences were conducted with ClustalW or MultAlin software (http://bioinfo.genotoul.fr/multalin/multalin.html). GPCRs were categorized according to class and family based on sequence similarity to invertebrate and mammalian GPCRs and named according to nomenclature guidelines developed for invertebrate vectors as detailed at VectorBase (Supplementary Table 26). GPCR annotations described in this publication will be made available as third party annotations through VectorBase. Full length cDNAs of the following putative receptors were cloned and NCBI accession numbers were obtained as follows: Family A: 1. Kinin receptor (HM807526), 2. Periviscerokinin/CAPA receptor (JQ771528), 3. Orphan neuropeptide receptor (HM771426); Family B: Corticotropin-releasing hormone-like (CRF-like) receptor 2a (JF837597).

Ixodes scapularis Chemosensory Ligand-Binding Protein Gene Families

The search for putative homologs of the odorant-binding protein (OBP), chemosensory protein (CSP) and chemosensory protein family B (CheBs) genes was conducting as previously described[145], and included several rounds of exhaustive searches using information from known protein sequences as queries[146,147,148,149,150,151,152]. First, we searched the preliminary predicted gene set using BLASTP (BLOSUM45 matrix with an *e* value threshold of 10^{-5}), HMMER (http://hmmer.wustl.edu/) (*e* value domain threshold of 10^{-5}), and HHsearch[153] (*e*-value threshold of 10^{-5}). The HMMER and HHsearch searches used Pfam[154], PBP/GOBP (for OBP; PF01395), and OS-D (for CSP; PF03392), lipocalin (for vertebrate OBP; PF00061) HMM profiles. Furthermore, because some chemosensory family members are highly divergent, we also built extra custom HMM profiles (used in all HMMER and HHsearch searches). In the case of CheBs we used the members of the family recently identified and characterized by the J. Rozas group in the 12 *Drosophila* genomes. We built these profiles after clustering known protein sequences representative of all relevant phylogentic groups with BlastClust (ftp://ftp.ncbi.nih.gov/genomes) (*e* value threshold of 10^{-5}, length coverage "-L" of 0.5 and score density "-S" of 0.6). We selected the four clusters with the highest numbers of sequences, aligned the clusters separately with MAFFT[155] (E-INS-i with BLOSUM30 matrix, 10,000 maxiterate, and offset "0") and, for each cluster, built an HMM profile using HMMER. Second, we searched the raw DNA sequence data using TBlastN (BLOSUM45 with *e* value threshold of 10^{-3}),

EXONERATE[22] (50% of the maximum store threshold), and HMMER (*e* value domain threshold of 10^{-10}). For the latter analysis, we searched against all 6-frames using Pfam's and our custom HMM profiles as queries. All searches were performed exhaustively until no new hit was found, adding always all newly identified members to the queries. Finally, all results were manually curated, and the putative gene structure was checked for known OBP/CSP/CheB characteristics (signal peptide, typical secondary structures, presence of start and stop codons, etc).

***Ixodes scapularis* Gustatory Receptor (GR) Genes**

The GR family was manual annotated using methods employed for insect and *Daphnia* genomes[156]. Briefly, TBLASTN searches were performed using major lineages of insect and *Daphnia* GRs as queries, and gene models were manually assembled in TEXTWRANGLER. Iterative searches were also conducted with each new tick protein as query until no new genes were identified in each major subfamily or lineage. Many of the genes identified are missing one or more short C-terminal exons, and while some of these were identified from raw reads, leading to fixed gene models, many were not. A final check for possible divergent genes/proteins was performed by HMMER at VectorBase using the automated annotations, and revealed nine existing models and just two additional highly divergent genes/proteins, Gr47 and 62. All of the IsGr genes and encoded proteins are detailed in Supplementary Table 29. All IsGr proteins are provided below in FASTA format.

The IsGr gene set consists of 62 models, comparable in size with that of many insects and *Daphnia*. There were only five obvious pseudogenes, although some of the currently incomplete gene models might in fact be pseudogenes, and there are many gene fragments remaining in the genome. Gene models were present in the automated annotations for just 11 of these genes, and only one was precisely correct. For the genes that are intact within existing supercontigs, 23 new models have been added to the annotation, indicated with numbers starting with 800 in Supplementary Table 29. Although there are no ESTs for these Grs in the limited available transcriptome data, the basic gene structure for the entire IsGr set is a long first exon, followed by three short C-terminal exons separated by three phase 0 introns. The locations of these introns and their phases are the same as predicted by[157] to be ancestral to the entire insect chemoreceptor superfamily, and are also shared with Gr genes in other animals (Robertson, *unpublished*). The only major exception is the Gr47-60 lineage, which are intronless in the coding region, presumably resulting from an ancient gene conversion with a reverse-transcribed mRNA.

Phylogenetic Analysis of the* Ixodes scapularis *GRs. GR protein sequences of *D. melanogaster, An. gambiae, D. pulex* and *I. scapularis* were aligned with MAFFT using standard parameters (gap opening penalty = 1.530 and offset = 0.123) and 1000 iterations. Phylogenetic analysis was performed with the RAXML 7.0.4[158] software using the PROTGAMMAWAG model. Tree figure (Supplementary Fig. 20) was edited with FigTree 1.3.1 (http://tree.bio.ed.ac.uk/software/figtree).

***Ixodes scapularis* Cys-loop and iGluR Ligand-gated Ion Channel Genes**

iGluR and IR genes were identified and annotated using previously described methods[159] (Supplementary Fig. 21; Supplementary Tables 30-31).

MicroRNAs (miRNAs) in *Ixodes scapularis*

Three different sets of microRNA (miRNA) gene predictions were consolidated from miRBase[160], miROrtho[161], and VectorBase[162] resulting in the identification of a conservative set of 45 predicted miRNA genes (Supplementary Table 6). These include likely orthologs of recognized miRNAs such as *bantam* and *iab-4*. Although this set of miRNAs is unlikely to be complete, it is comparable in number to predictions from other arthropod genomes: *e.g.,* 52 in the genome of the spider mite, *T. urticae*[163], 50 in the water flea, *D. pulex*[34], and 57 in the body louse, *P. humanus*[164].

***Ixodes scapularis* Proteomics**

Ixodes scapularis ISE6 cells (provided by Timothy J. Kurtti, University of Minnesota) were grown at 34°C in the absence of CO_2 with L15B-300 complete media[165]. Cells were harvested followed by lipid removal ($CHCl_3$: MeOH), acetone protein precipitation and denaturation. The protein samples were digested with trypsin and the resulting peptides were analyzed by high-pressure liquid chromatography (HPLC) and ESI-MS/MS with a hybrid ion trap mass spectrometer LTQ-Orbitrap LX (Thermo Scientific) at the Purdue Proteomics Facility, Bindley Bioscience Center. Mass spectrometry (MS) data were processed using with the Omics Discovery Pipeline[166,167] and MS/MS peptide identification was performed using the Agilent Technologies Spectrum Mill MS Proteomics Workbench. The *I. scapularis* Wikel strain IscaW1.2 predicted protein set (https://www.vectorbase.org/) was used to perform the MS/MS protein database search and reverse scores were calculated to account for decoy database searching. Significant LC-MS peaks ($p \leq 0.05$) discovered by the Omics Discovery Pipeline were matched to corresponding m/z values and retention times of a MS/MS peptide library (identified from Spectrum Mill). These identified peptides were subject to filtering by removing non-confident peptides and false positives[168,169]. This stringent analysis produced a final data set comprising approximately 486 proteins. This data set was queried to provide support for *I. scapularis* heme biosynthesis gene model predictions (Section S8).

***Ixodes* Proteins Associated With *Anaplasma* Infection**

Cell Culture and Protein Extraction*.* The tick cell line ISE6, derived from *I. scapularis* embryos (provided by U.G. Munderloh, University of Minnesota, USA), was cultured in L15B medium as described previously[170], but the osmotic pressure was lowered by the addition of one fourth sterile water by volume. The ISE6 cells were inoculated with *A. phagocytophilum* (NY18 isolate)-infected HL-60 cells as described previously[170,171]. Uninfected and infected cultures (N=5 independent cultures each) were sampled at 3 days post-infection (dpi) (early infection; percent infected cells 11-17% (Avg±SD, 13±2)) and 10 dpi (late infection; percent infected cells 56-61% (Avg±SD, 58±2)), the cells were centrifuged at 10,000 g for 3 min, and cell pellets were frozen in liquid nitrogen until used for protein extraction. Approximately 10^7 cells were pooled from each condition and lysed in 350 µl lysis buffer (PBS, 1% Triton X-100, 1 mM sodium vanadate, 1 mM sodium fluoride, 1 mM PMSF, 1µg/ml leupeptin, 1µg/ml pepstatin) for 30 min at 4ºC. Total cell extracts were centrifuged at 200 g for 5 min to remove cell debris. The supernatants were collected and protein concentration was

determined using the Bradford Protein Assay (Bio-Rad, Hercules, CA, USA) with BSA as standard.

Proteomics analysis of infected and uninfected* Ixodes scapularis *ISE6 tick cells. Proteomics analysis of *I. scapularis* ISE6 tick cells in response to *A. phagocytophilum* infection was performed using protein one-step in-gel digestion, peptide iTRAQ labeling, IEF fractionation, LC-MS/MS analysis and peptide identification. Protein extracts from the four experimental conditions, control uninfected early (CE), infected early (IE), control uninfected late (CL) and infected late (IL) (100 µg each) were resuspended in up to 300 µl of sample buffer and applied using a 5-well comb on a conventional SDS-PAGE gel (1.5 mm-thick, 4% stacking, 10% resolving). The run was stopped as soon as the front entered 3 mm into the resolving gel so that the whole proteome became concentrated in the stacking/resolving gel interface. The unseparated protein bands were visualized by Coomassie Brilliant Blue R-250 staining, excised, cut into cubes (2 mm^2) and digested overnight at 37ºC with 60 ng/µl trypsin (Promega, Madison, WI, USA) at 5:1 protein:trypsin (w/w) ratio in 50 mM ammonium bicarbonate, pH 8.8 containing 10% (v/v) acetonitrile (ACN) and 0.01% (w/v) 5-cyclohexyl-1-pentyl-ß-D-maltoside (CYMAL-5)[172]. The resulting tryptic peptides from each proteome were extracted by 1 hr incubation in 12 mM ammonium bicarbonate, pH 8.8. trifluoroacetic acid (TFA) was added to a final concentration of 1% and the peptides were finally desalted onto C18 OASIS HLB Extraction cartridges (Waters, Milford, Massachusetts, USA) to remove the amine-containing buffers and dried-down.

Dried peptides were taken up in 30 µl of iTRAQ dissolution buffer provided with the kit (Applied Biosystems, Madrid, Spain) and labeled by adding 70 µl of the corresponding iTRAQ reagent in ethanol and incubating for 1 hr at room temperature in 70% ethanol, 180 mM triethylammoniumbicarbonate (TEAB), pH 8.53. CE was labeled with 114, IE was labeled with 115, CL was labeled with 116 and IL labeled with 117 iTRAQ tags. After quenching the reaction with 100 µl 0.1% formic acid for 30 min, samples were brought to dryness to completely stop the labeling reaction. This quenching process was repeated once more to promote TEAB volatilization. The four labeled samples were resuspended in 100 µl 0.1% formic acid and combined into one tube. The mixture was dried down, redissolved in 3.3 ml 5 mM ammonium formiate, pH 3, cleaned up with SCX Oasis cartridges (Waters) using as elution solution 1 M ammonium formiate pH 3, containing 25% ACN, and dried down. The peptide pools were resuspended in 0.5 ml 0.1% TFA, desalted onto C18 Oasis cartridges using as elution solution 50% ACN in 5 mM ammonium formiate, pH 3 and dried down. The sample was taken up in focusing buffer (5% glycerol and 2% IPG buffer pH 3-10 (GE Healthcare, Madrid, Spain) loaded onto 24-wells over a 24 cm-long Immobiline DryStrip, pH3-10 (GE Healthcare) and separated by IEF on a 3100 OFFgel fractionator (Agilent, Santa Clara, CA, USA), using the standard method for peptides recommended by the manufacturer. The recovered fractions were acidified with 20 µl of 1 M ammonium formiate, pH 3, and the peptides were desalted using OMIX C18 tips (Varian, Palo Alto, CA, USA). After elution with 50% ACN in 5 mM ammonium formiate, pH 3, the peptides were dried-down prior to RP-HPLC-LIT analysis. All samples were analyzed by LC-MS/MS using a Surveyor LC system coupled to a linear ion trap mass spectrometer model LTQ (Thermo-Finnigan, San Jose, CA, USA) as described previously[173]. The LTQ was programmed to perform a data-dependent MS/MS scan on the 15 most intense precursors detected in a full scan from 400 to 1600 amu (3 µscans, 200 ms

injection time, 10,000 ions target). Singly charged ions were excluded from the MS/MS analysis. Dynamic exclusion was enabled using the following parameters: 2 repeat counts, 90 s repeat duration, 500 exclusion size list, 120 s exclusion duration and 2.1 amu exclusion mass width. PQD parameters were set at 100 ms injection time, 8 microscans per scan, 2 amu isolation width, 28% normalized collision energy, 0.6 activation Q, 0.3 ms activation time. For PQD spectra generation 10,000 ions were accumulated as target and automatic gain control was used to prevent over-filling of the ion trap. Protein identification was carried out as described previously[173] using SEQUEST algorithm (Bioworks 3.2 package, Thermo Finnigan), allowing optional (Methionine oxidation) and fixed modifications (Cysteine carboxamidomethylation, Lysine and N-terminal modification of +144.1020 Da). The MS/MS raw files were searched against the alphaproteobacteria combined with the arachnida Swissprot database (Uniprot release 15.5, 7 July, 2009) supplemented with porcine trypsin and human keratins. This joint database contains 638,408 protein sequences. To calculate false discovery rate, the same collections of MS/MS spectra were also searched against inverted databases constructed from the same target databases. The alphaproteobacteria Swissprot database was used to identify and discard *Anaplasma* and possible symbiotic bacterial sequences from further analyses.

A total of 1447 MS/MS spectra were assigned to 903 unique peptides[174] (false discovery rate, FDR=10%). After identifying and discarding *Anaplasma* and other bacterial symbiotic peptide sequences, the 735 remaining peptides belonged to 424 different proteins (Supplementary Tables 32-35). Of these, 88% had similarity to *Ixodes* sequences while 95% had similarity to sequences from other tick species (Supplementary Table 35). Proteomics data showed a strong correlation with conceptual coding sequences predicted from the *I. scapularis* genome. For some of the identified proteins, the discrepancy between peptide data and predicted protein sequence may reflect polymorphisms between ISE6 cells and the Wikel tick strain and the need to improve *I. scapularis* gene models.

Population Structure of *Ixodes scapularis* Across North America

Sample collection

Ixodes scapularis adult females were collected from eight geographical locations in the USA: Florida, Indiana, Maine, Massachusetts, New Hampshire, North Carolina, Virginia, and Wisconsin by our research group or kindly provided by collaborators. In addition, samples were obtained for the reference Wikel strain from the University of Texas Medical Branch, Galveston, TX. The colony has been maintained in continuous culture since establishment. The GPS location was recorded for each field collected sample. Samples were stored in 80% ethanol at 4°C, in ALT buffer (SIGMA) or at -70°C until processing. Genomic DNA was separately extracted from individual females using a phenol:chloroform:isopropyl alcohol (SIGMA) method and treated with RNAse A (Ambion).

RAD library preparation

RAD-seq libraries were produced from 77 individual female *I. scapularis*. One µg genomic DNA from each individual was digested in a 50 µL reaction with 100 units of *Sbf*I-HF restriction enzyme (New England Biolabs, Beverly MA, USA) for 1.5 hrs at

37°C, followed by incubation at 65°C for 20 minutes to inactivate the enzyme. An aliquot (1 µl) was analyzed on 1% agarose gel to check the digestion efficiency and the remaining product was ligated to the unique P1 RAD adapter primers (50 nM per reaction) with 1000 units of T4 ligase in 1× NEB buffer 2 (New England Biolabs) and 100 mM rATP (Fermentas). Samples were incubated for one hr at 20°C, followed by enzyme inactivation at 65°C for 20 minutes. Adapter ligated DNA fragments from individual samples were pooled and sonicated using Qsonica sonicator for six minutes at maximum power. Samples were cleaned with MinElute PCR purification kit (Qiagen). Fragments of 400-600 bp were selected using 1% agarose gel and DNA was recovered with the MinElute gel extraction kit (Qiagen). Blunt ends were repaired using blunting enzyme mix (New England Biolabs) in 1X blunting buffer and 1mM dNTP mix. Samples were incubated for one hr at 20°C and purified with MinElute PCR purification kit (Qiagen). A-overhangs (10mM dATP; Fermentas) were then added using Klenow fragment (3'-5' exo) (New England Biolabs) in 1x NEB Buffer 2. Samples were incubated for one hr at 20°C and purified with MinElute PCR purification kit (Qiagen). The P2 RAD adapter (10 µM) was ligated using 1000 units of T4 DNA ligase (New England Biolabs) in 1× NEB buffer 2 (New England Biolabs) and 100mM rATP (Fermentas). Samples were incubated for one hr at 20°C followed by purification with MinElute PCR purification kit (Qiagen).

Finally, 10 µL of the P1 and P2 adapter ligated DNA was used as a template in a 100 µL PCR reaction with 50 µL of the Phusion High Fidelity 2× Master mix (New England Biolabs) and 2 µL each of 10 µM P1 and P2 primers. PCR conditions were: 98°C for 30 s, 14 cycles of 98°C for 10 s, 65°C for 30 s, 72°C for 30 s, and a final elongation step at 72°C for 5 min. Samples were sequenced on the Illumina HiSeq2500 platform in the Rapid run mode to obtain 150 bp single-end reads.

Sequence processing and SNP calling

Illumina reads were processed by the Bioinformatics Core at Purdue University. Reads were corrected for barcodes and restriction site, low quality bases (Phred score less than 10) were trimmed and all reads were trimmed to 140 bp and then de-multiplexed (sorted by barcode) using the "process_radtags.pl" script of STACKS[175,176]. Quality trimmed reads were separately aligned to the *I. scapularis* Wikel genome assembly, IscaW1 (Ixodes-scapularis-Wikel_SCAFFOLDS_IscaW1.fa downloaded from VectorBase) using the end-to-end mode and default parameters of Bowtie2 v 2.2.3[177]. Three individual samples with the percent of mapped reads less than 50% were removed from analysis. Polymorphic loci (catalogue loci) were identified for SNP discovery using the ref_map.pl pipeline in STACKS version (v1.19). First, sequences aligned to the same genomic location were stacked together and merged to form loci. Only loci with a sequencing depth of ten or more reads per individual were retained. SNPs at each locus were called by STACKS implementing a multinomial-based likelihood model regardless of the reference sequence itself. Lastly, a catalogue of all possible loci and alleles was generated and each individual was matched against the catalogue. In total, 745,760 SNPs across 35,460 loci were identified using the 'population' program within the STACKS package based on the criteria: (1) minimum 60% individuals within a population, (2) minimum two populations to report a locus, and (3) minimum stack depth of 10 per locus.

F-statistics and Population structure

The *population* program within STACKS (v1.19) was used[176] in combination with the system of Wright[178] to assess fixation index and genetic variation within and among populations. The F statistic was used to measure fixation index (F_{IS}) and genetic variability (F_{ST}). Using 745,760 SNPs, genome-wide measures of diversity, such as observed heterozygosity (H_O), expected heterozygosity (H_E), nucleotide diversity (π) across individuals (intra-population) and genetic differentiation were calculated to assess genetic distance or differentiation as evidence of selection. We enabled kernel smooth function in *population* with a default window size of 150kb such that a kernel smooth function (weights function) was applied to all SNP locations within a sliding window covering a 3x150 Kb region at either side of a center polymorphic locus. This function uses the distance between each SNP within the sliding window and the center SNP, and the defined window size, so that F_{IS} have stable values within each sliding window and across the whole genome. The same process was conducted for π. At each SNP locus, π was calculated from the count of a specific allele in the population, and the sample size of all alleles in the population[179,180]. At each SNP location, $F_{IS} = 1 - Ho/\pi$. The reported F_{IS} (Supplementary Table 36) is the population-level mean value across all the polymorphic sites within each sub-population.

F_{ST} is an indication of variation among populations. At each SNP position, F_{ST} was calculated by the following formula[176,180,181]:

$$F_{ST} = 1 - \frac{\sum_j \binom{n_j}{2} \pi_j}{\pi_{all} \sum_j \binom{n_j}{2}}$$

where, n_j is the sample size of alleles in population *j*, and π_j is π in population *j*, while π_{all} is π calculated over the pair of populations (pairwise comparison between two sub-populations) (Supplementary Table 37).

In addition, we used *fastStructure* (beta release)[182] to assess population structure using a genome wide set of 745,760 SNPs across 35,460 catalogue loci (~21 SNPs per loci) and a subset of 34,693 SNPs, the first SNP per catalogue locus to resolve genetic structure at a broad spatial scale. *fastStructure* delineates clusters of individuals on the basis of genotypes at multiple loci using a Bayesian approach. Models were fitted with a defining number of clusters (K) from 1 to 20. Next, the most suitable K (K=6) was selected for the full set of 745,760 SNPs using a python script chooseK.py from *fastStructure*. Briefly, marginal likelihood values for K=1 to 20 were manually vetted. Marginal likelihood increased from -0.4730 to -0.3882 when K increased from 1 to 6, and then decreased by 0.01 at K=7 (range -0.3953 to -0.3825). The same method was used to select the most suitable K (K=5) for the subset of 34,693 SNPs. Marginal likelihood increased from -0.4975 to -0.4004 when K increased from 1 to 5, then decreased to -0.4045 at K=6 reaching a plateau afterwards. Using the output from *fastStructure* and *DISTRUCT* (v1.1)[183], a bar plot was created where each individual of the sample is represented by a vertical line divided into *K* colored segments with the length of each segment being proportional to the estimated membership in each of the inferred *K* groups.

Expression of *Ixodes scapularis* ligand-gated ion channels in *Xenopus laevis* oocytes

Functional expression of an *Ixodes scapularis* ligand-gated ion channel subunit (known as IscaGluCl1) in *Xenopus laevis* oocytes (Fig. 7; Supplementary Fig. 25) was achieved by cRNA injection and two-electrode voltage clamp electrophysiology. A brief description of the methods is provided in the main paper and a more comprehensive account of the technique is available[184].

Supplementary References

1. Geraci, N. S., Johnston, J. S., Robinson, J. P., Wikel, S. K. & Hill, C. A. Variation in genome size of argasid and ixodid ticks. *Insect Biochem. Mol. Biol.* **37**, 399-408 (2007).

2. Hill, C. A. *et al.* The position of repetitive DNA sequence in the southern cattle tick genome permits chromosome identification. *Chromosome Res*. **17**, 77-89 (2009).

3. Myers, E. W. *et al.* A whole-genome assembly of *Drosophila*. *Science* **287**, 2196-2204 (2000).

4. Rusch, D. B. *et al.* The Sorcerer II Global Ocean Sampling Expedition: Northwest Atlantic through Eastern Tropical Pacific. *PLoS Biol* **5**, e77 (2007). doi:10.1371/journal.pbio.0050077.

5. Levy, S. *et al.* The diploid genome sequence of an individual human. *PLoS Biol*. **5**, e254 (2007). doi:10.1371/journal.pbio.0050254.

6. Denisov, G. *et al.* Consensus Generation and Variant Detection by Celera Assembler. *Bioinformatics* **24**, 1035-1040 (2008).

7. Miller, J. R. *et al.* Aggressive Assembly of Pyrosequencing Reads with Mates. *Bioinformatics* **24**, 2818-2824 (2008).

8. Gillespie, J. J. *et al.* PATRIC: The Comprehensive Bacterial Bioinformatics Resource with a Focus on Human Pathogenic Species. *Infect. Immun.* **79**, 4286-4298 (2011).

9. Kurtti, T. J. *et al. Rickettsia buchneri* sp. nov., a rickettsial endosymbiont of the blacklegged tick *Ixodes scapularis*. *Internat. J. System. Evol. Microbiol*. **65**, 965–970 (2015).

10. Gillespie, J. J. *et al.* A Rickettsia genome overrun by mobile genetic elements provides insight into the acquisition of genes characteristic of an obligate intracellular lifestyle. *J. Bacteriol.* **194**, 376-394 (2012).

11. Price, A. L., Jones, N. C. & Pevzner, P. A. *De novo* identification of repeat families in large genomes. *Bioinformatics* **21**, i351-8 (2005). doi:10.1093/bioinformatics/bti1018.

12. Bao, Z. & Eddy, S. R., Automated *de novo* identification of repeat sequence families in sequenced genomes. *Genome Res*. **12**, 1269-1276 (2002).

13. Smit, A. F. A., Hubley, R. & Green, P. *RepeatMasker Open-3.0*. 1996-2010. <http://www.repeatmasker.org>.

14. Huang, X., Adams, M. D., Zhou, H. & Kerlavage, A. R. A tool for analyzing and annotating genomic sequences. *Genomics* **46**, 37-45 (1997).

15. Birney, E., Clamp, M. & Durbin, R., GeneWise and Genomewise. *Genome Res.* **14**, 988-995 (2004).

16. Haas, B. J. *et al.* Improving the *Arabidopsis* genome annotation using maximal transcript alignment assemblies. *Nucl. Acids Res.* **31**, 5654-5666 (2003).

17. Stanke, M. & Morgenstern, B. AUGUSTUS: a web server for gene prediction in eukaryotes that allows user-defined constraints. *Nucl. Acids Res.* **33**: W465-W467 (2005). doi:10.1093/nar/gki458.

18. Majoros, W., Pertea, M., Delcher, A. L. & Salzberg, S. L. Efficient decoding algorithms for generalized hidden Markov model gene finders. *BMC Bioinformatics* **6**, 16 (2005). doi:10.1186/1471-2105-6-16.

19. Haas, B. J. *et al.* Automated eukaryotic gene structure annotation using EVidenceModelerand the Program to assemble spliced alignments. *Genome Biol.* 9, R7 (2008). doi:10.1186/gb-2008-9-1-r7.

20. Flicek, P. *et al.* Ensembl's 10th year. *Nucl. Acids Res.* **38**, D557-D562 (2010). doi:10.1093/nar/gkp972.

21. The UniProt Consortium. Ongoing and future developments at the Universal Protein Resource. *Nucl. Acids Res.* **39**, D214-D219 (2011). doi:10.1093/nar/gkq1020.

22. Slater, G. S. & Birney, E. Automated generation of heuristics for biological sequence comparison. *BMC Bioinformatics* **6**, 31 (2005).

23. Korf, I. Gene finding in novel Genomes. *BMC Bioinformatics* **5**, 59 (2004). doi:10.1186/1471-2105-5-59.

24. Conesa, A. *et al.* Blast2GO: a universal tool for annotation, visualization and analysis in functional genomics research. *Bioinformatics* **21**, 3674-3676 (2005).

25. Waterhouse, R. M., Zdobnov, E. M., Tegenfeldt, F., Li, J. & Kriventseva, E. V. OrthoDB: the hierarchical catalog of eukaryotic orthologs in 2011. *Nucleic Acids Res.* **39**, D283-D288 (2011).

26. Edgar, R. MUSCLE: multiple sequence alignment with high accuracy and high throughput. *Nucl. Acids Res*. **32**, 1792-1797 (2004).

27. Castresana, J. Selection of conserved blocks from multiple alignments for their use in phylogenetic analysis. *Mol. Biol. Evol.* **17**, 540-552 (2000).

28. Guindon, S. *et al.* New algorithms and methods to estimate maximum-likelihood phylogenies: assessing the performance of PhyML 3.0. *Syst. Biol.* **59**, 307-321 (2010).

29. Rogozin, I. B., Wolf, Y. I., Sorokin, A. V., Mirkin, B. G. & Koonin, E. V., Remarkable interkingdom conservation of intron positions and massive, lineage-specific intron loss and gain in eukaryotic evolution. *Curr. Biol.* **13**, 1512-1517 (2003).

30. Rogozin, I. B., Lyons-Weiler, J. & Koonin, E. V. Intron sliding in conserved gene families. *Trends Genet*. **16**, 430-432 (2000).

31. Felsenstein, J. Coalescents in full bloom. *Evolution* **63**, 3275–3276 (2009).doi:10.1111/j.1558-5646.2009.00811.x.

32. Junier, T. & Zdobnov, E. M. The Newick utilities: high-throughput phylogenetic tree processing in the Unix shell. *Bioinformatics* **26**, 1669-1670 (2010).

33. Csurös, M. *et al.* Malin: maximum likelihood analysis of intron evolution in eukaryotes. *Bioinformatics* **24**, 1538-1539 (2008).

34. Colbourne, J. K. *et al.* The ecoresponsive genome of *Daphnia pulex*. *Science* **331**, 555-561 (2011).

35. Li, L., Stoeckert, C. J. Jr. & Roos, D. S. OrthoMCL: identification of ortholog groups for eukaryotic genomes. *Genome Res.* **13**, 2178-2189 (2003).

36. Chen, F., Mackey, A. J., Vermunt, J. K. & Roos, D. S. Assessing performance of orthology detection strategies applied to eukaryotic genomes. *PLoS ONE* **2**(4): e383, (2007). doi:10.1371/journal.pone.0000383.

37. Schlueter, J. *et al.* Mining EST databases to resolve evolutionary events in major crop species. *Genome*. **47**, 868-876 (2004).

38. Rice, P., Longden, I. & Bleasby, A. EMBOSS: the European molecular biology open software suite. *Trends Genet.* **16**, 276–277 (2000).

39. Lynch, M. & Conery, J. The evolutionary fate and consequences of duplicate genes. *Science* **290**, 1151-1155 (2000).

40. Benson, G. Tandem repeats finder: a program to analyze DNA sequences. *Nucl. Acids Res.* **27**, 573–580 (1999).

41. Meyer, J. M., Kurtti, T. J., Van Zee, J. P. & Hill, C. A. Genome organization of major tandem repeats in the hard tick, *Ixodes scapularis. Chrom. Res.* **18**, 357-370 (2010).

42. Tu, Z. Eight novel families of miniature inverted repeat transposable elements in the African malaria mosquito, *Anopheles gambiae*. *Proc. Natl. Acad. Sci U.S.A*. **98**, 1699-1704 (2001).

43. McCarthy, E. M. & McDonald, J. F. LTR_STRUC: a novel search and identification program for LTR retrotransposons. *Bioinformatics* **19**, 362-367 (2003).

44. Tubio, J. M., Naveira, H. & Costas, J. Structural and evolutionary analyses of the Ty3/gypsy group of LTR retrotransposons in the genome of *Anopheles gambiae*. *Mol. Biol. Evol.* **22**, 29-39 (2005).

45. Tubio, J. M. *et al.* Evolutionary dynamics of the Ty3/gypsy LTR retrotransposons in the genome of *Anopheles gambiae*. *PloS ONE* **6**, e16328, (2011). doi:10.1371/journal.pone.0016328.

46. Jurka, J. *et al.* Repbase update, a database of eukaryotic repetitive elements. *Cytogenet Genome Res*. **110**, 462-467 (2005).

47. Altschul, S. F. *et al.* Gapped BLAST and PSI-BLAST: a new generation of protein database search programs. *Nucl. Acids Res.* **25**, 3389-3402 (1997).

48. Tatusova, T. A. & Madden, T. L. Blast 2 sequences-a new tool for comparing protein and nucleotide sequences. *FEMS Microbiol. Lett*. **174**, 247-250 (1999).

49. Kennedy, R. C., Unger, M. F., Christley, S., Collins, F. H. & Madey, G. R. An automated homology-based approach for identifying transposable elements. *BMC Bioinformatics* **12**, 130, (2011). doi:10.1186/1471-2105-12-130.

50. Xiong, Y. & Eickbush, T. H. Origin and evolution of retroelements based upon their reverse transcriptase sequences. *EMBO J.* **9**, 3353-3362 (1990).

51. Malik, H. S., Burke, W. D. & Eickbush, T. H. The age and evolution of non-LTR retrotransposable elements. *Mol. Biol. Evol*. **16**, 793–805 (1999).

52. Guindon, S. & Gascuel, O. A Simple, Fast, and Accurate Algorithm to Estimate Large Phylogenies by Maximum Likelihood. *Syst. Biol.* **5**, 696-704 (2003).

53. Lander, E. S. *et al.* Initial sequencing and analysis of the human genome. *Nature* **409**, 860-921 (2001).

54. Holt, R. A. *et al.* The genome sequence of the malaria mosquito *Anopheles gambiae*. *Science* **298**, 129-149 (2002).

55. Lovsin, N., Gubensek, F. & Kordi, D. Evolutionary dynamics in a novel L2 clade of non-LTR retrotransposons in Deuterostomia. *Mol. Biol. Evol.* **18**, 2213–2224 (2001).

56. Biedler, J. & Tu, Z. Non-LTR retrotransposons in the African malaria mosquito, *Anopheles gambiae:* unprecedented diversity and evidence of recent activity. *Mol. Biol. Evol*. **20**, 1811–1825 (2003).

57. Arensburger, P. *et al.* Sequencing of *Culex quinquefasciatus* establishes a platform for mosquito comparative genomics. *Science* **330**, 86-88 (2010).

58. Jakubczak, J. L., Burke, W. D. & Eickbush, T. H. Retrotransposable elements R1 and R2 interrupt the rRNA genes of most insects. *Proc. Natl. Acad. Sci. U.S.A*. **88**, 3295–3299 (1991).

59. Felger, I. & Hunt, J. A. A non-LTR retrotransposon from the Hawaiian Drosophila: the LOA element. *Genetica* **85**, 119–130 (1992).

60. Munderloh, U. G., Liu, Y., Wang, M., Chen, C. & Kurtti, T. J. Establishment, maintenance and description of cell lines from the tick *Ixodes scapularis. J. Parasitol*. **80**, 533–543 (1994).

61. Chen, C., Munderloh, U. G. & Kurtti, T. J. Cytogenetic characteristics of cell lines from *Ixodes scapularis* (Acari: Ixodidae). *J. Med. Entomol*. **31**, 425-434 (1994).

62. Zwick, M. S. *et al.* A rapid procedure for the isolation of C_0t-1 DNA from plants. *Genome* **40**, 138-142 (1997).

63. Vítková, M., Král, J., Traut, W., Zrzavy, J. & Marec, F. The evolutionary origin of insect telomeric repeats, (TTAGG)n. *Chromosome Res*. **13**, 145-156 (2005).

64. Francischetti, I. M. B., Sá-Nunes, A., Mans, B. J., Santos, I. M. & Ribeiro, J. M. C. The role of saliva in tick feeding. *Front. Biosci.* **14**, 2051-2088 (2009).

65. Ribeiro, J. M. & Arca, B. From sialomes to the sialoverse: An insight into the salivary potion of blood feeding insects. *Adv. Insect Physiol.* **37**, 59-118 (2009).

66. Arca, B. *et al.* An updated catalogue of salivary gland transcripts in the adult female mosquito, *Anopheles gambiae*. *J. Exp. Biol.* **208**, 3971-3986 (2005).

67. Panzera, F. *et al.* Genome size determination in chagas disease transmitting bugs (hemiptera-triatominae) by flow cytometry. *Am. J. Trop. Med. Hyg.* **76**, 516-521 (2007).

68. Kondrashov, F. A., Rogozin, I. B., Wolf, Y. I. & Koonin, E. V. Selection in the evolution of gene duplications. *Genome Biol.* **3**, research0008, doi:10.1186/gb-2002-3-2-research0008 (2002).

69. Sankoff, D. Gene and genome duplication. *Curr. Opin. Genet. Dev.* **11**, 681-684 (2001).

70. Ascenzi, P. *et al.* The bovine basic pancreatic trypsin inhibitor (Kunitz inhibitor): a milestone protein. *Curr. Protein Pept. Sci.* **4**, 231-251 (2003).

71. Castaneda, O. & Harvey, A. L. Discovery and characterization of cnidarian peptide toxins that affect neuronal potassium ion channels. *Toxicon*. **54**, 1119-11124 (2009).

72. Harvey, A. L. Recent studies on dendrotoxins and potassium ion channels. *Gen. Pharmacol*. **28**, 7-12 (1997).

73. Paesen, G. C. *et al.* An ion-channel modulator from the saliva of the brown ear tick has a highly modified Kunitz/BPTI structure. *J. Mol. Biol.* **389**, 734-47 (2009).

74. Ribeiro, J. M. *et al.* An annotated catalog of salivary gland transcripts from *Ixodes scapularis* ticks. *Insect Biochem. Mol. Biol.* **36**, 111-129 (2006).

75. Valenzuela, J. G. *et al.* Exploring the sialome of the tick, *Ixodes scapularis*. *J. Exp. Biol.* **205**, 2843-2864 (2002).

76. Kotsyfakis, M., Karim, S., Andersen, J. F., Mather, T. N. & Ribeiro, J. M. Selective cysteine protease inhibition contributes to blood-feeding success of the tick *Ixodes scapularis*. *J. Biol. Chem.* **282**, 29256-29263 (2007).

77. Schultz, J., Copley, R. R., Doerks, T., Ponting, C. P. & Bork, P. SMART: a web-based tool for the study of genetically mobile domains. *Nucleic Acids Res.* **28**, 231-234 (2000).

78. Campbell, C. L. *et al.* Midgut and salivary gland transcriptomes of the arbovirus vector *Culicoides sonorensis* (Diptera: Ceratopogonidae). *Insect Mol. Biol.* **14**, 121-36 (2005).

79. Wilson, A. D., Heesom, K. J., Mawby, W. J., Mellor, P. S. & Russell, C. L. Identification of abundant proteins and potential allergens in *Culicoides nubeculosus* salivary glands. *Vet. Immunol. Immunopathol.* **122**, 94-103 (2008).

80. Andersen, J. F., Pham, V. M., Meng, Z., Champagne, D. E. & Ribeiro, J. M. Insight into the Sialome of the Black Fly, *Simulium vittatum*. *J. Proteome Res*. **8**, 1474-1488 (2009).

81. Francischetti, I. M., Mather, T. N. & Ribeiro, J. M. Penthalaris, a novel recombinant five-Kunitz tissue factor pathway inhibitor (TFPI) from the salivary gland of

the tick vector of Lyme disease, *Ixodes scapularis*. *Thromb. Haemost.* **91**, 886-898 (2004).

82. Francischetti, I. M., Valenzuela, J. G., Andersen, J. F., Mather, T. N. & Ribeiro, J. M. Ixolaris, a novel recombinant tissue factor pathway inhibitor (TFPI) from the salivary gland of the tick, *Ixodes scapularis*: identification of factor X and factor Xa as scaffolds for the inhibition of factor VIIa/tissue factor complex. *Blood* **99**, 3602-3612 (2002).

83. Benson, D. A., Karsch-Mizrachi, I., Lipman, D. J., Ostell, J. & Sayers, E. W. GenBank. *Nucl. Acids Res.* **40**, D32-D53 (2011). doi:10.1093/nar/gkr1202.

84. Lawson, D. *et al.* VectorBase: a home for invertebrate vectors of human pathogens. *Nucleic Acids Res.*, **35**, D503–D505 (2007).

85. Lawson, D. *et al.* VectorBase: a data resource for invertebrate vector genomics. *Nucleic Acids Res.* **37**, D583–D587 (2009).

86. Kersey, P. J. *et al.* Ensembl Genomes: an integrative resource for genome-scale data from non-vertebrate species. *Nucl. Acids Res.* **40**, D91-D97, (2011).doi:10.1093/nar/gkr895.

87. Bateman, A. *et al.* The Pfam protein families database. *Nucl. Acids Res.* **32**, D138-D141 (2004). doi:10.1093/nar/gkh121.

88. Hulo, N. *et al.* The PROSITE database. *Nucl. Acids Res.* **34**, D227-D230 (2006). doi:10.1093/nar/gkj063.

89. Marchler-Bauer, A. *et al.* CDD: a Conserved Domain Database for the functional annotation of proteins. *Nuc. Acids Res.* **39**, D225-D229 (2011). doi:10.1093/nar/gkq1189.

90. Valanne, S., Wang, J. H. & Ramet, M. The *Drosophila* Toll signaling pathway. *J. Immunol*. **186**, 649-656 (2011).

91. Shin, S. W. *et al.* REL1, a homologue of *Drosophila* dorsal, regulates toll antifungal immune pathway in the female mosquito *Aedes aegypti*. *J. Biol. Chem.* **280**, 16499-16507 (2005).

92. Kim, J. H. *et al.* Comparison of the humoral and cellular immune responses between body and head lice following bacterial challenge. *Insect Biochem. Mol. Biol*. **41**, 332-339 (2011).

93. Gerardo, N. M. *et al.* Immunity and other defenses in pea aphids, *Acyrthosiphon pisum*. *Genome Biol*. **11**, R21 (2010). doi:10.1186/gb-2010-11-2-r21.

94. Goto, A. *et al.* Akirins are highly conserved nuclear proteins required for NF-κB-dependent gene expression in *Drosophila* and mice. *Nature Immunol.* **9**, 97-104 (2008).

95. de la Fuente, J. *et al.* The tick protective antigen, 4D8, is a conserved protein involved in modulation of tick blood ingestion and reproduction. *Vaccine* **24**, 4082-4095 (2006).

96. de la Fuente, J. *et al.* Functional genomic studies of tick cells in response to infection with the cattle pathogen, *Anaplasma marginale*. *Genomics* **90**, 712–722 (2007).

97. Kurscheid, S. *et al.* Evidence of a tick RNAi pathway by comparative genomics and reverse genetics screen of targets with known loss-of-function phenotypes in *Drosophila*. *BMC Mol. Biol*. **10**, 26 (2009). doi:10.1186/1471-2199-10-26.

98. Khvorova, A., Reynolds, A., Jayasena, S. D. Functional siRNAs and miRNAs exhibit strand bias. *Cell*. **115**, 209-216 (2003).

99. Schwarz, D. S., Hutvagner, G., Haley, B., Zamore, P. D. Evidence that siRNAs function as guides, not primers, in the *Drosophila* and human RNAi pathways. *Mol. Cell*. **10**, 537-548 (2002).

100. Hammond, S. M., Bernstein, E., Beach, D. & Hannon, G. J. An RNA-directed nuclease mediates post-transcriptional gene silencing in *Drosophila* cells. *Nature* **404**, 293-296 (2000).

101. Ding, S. W. RNA-based antiviral immunity. *Nat. Rev. Immunol*. **10**, 632-644 (2010).

102. Waterhouse, R. M. *et al.* Evolutionary dynamics of immune-related genes and pathways in disease-vector mosquitoes. *Science* **316**, 1738-1743 (2007).

103. Wang, Y. & Zhu, S. The defensin gene family expansion in the tick *Ixodes scapularis*. *Dev. Comp. Immunol.* **35**, 1128-1134 (2011).

104. Neese, P. A., Sonenshine, D. E., Kallapur, V. L., Apperson, C. S. & Roe, R. M. Absence of insect juvenile hormones in the American dog tick, *Dermacentor variabilis* (Say) (Acari: Ixodidae) and *Ornithodoros parkeri* Cooley (Acari: Argasidae). *J Insect Physiol*. **46**, 477-490 (2000).

105. Thompson, D. M. *et al. In vivo* role of 20-hydroxyecdysone in the regulation of the vitellogenin mRNA and egg development in the American dog tick, *Dermacentor variabilis* (Say). *J. Insect Physiol*. **51**, 1105-1116 (2005).

106. Pound, J. M., Oliver, J. H. Jr. Juvenile hormone: evidence of its role in the reproduction of ticks. *Science*. **206**, 355-357 (1979).

107. Donohue, K. V., Khalil, M. S., Mitchell, R. D., Sonenshine, D. E. & Roe, R. M. Molecular characterization of the major hemelipoglycoprotein in ixodid ticks. *Insect Mol. Biol*. **17**, 197-208 (2008).

108. Rutherford, K. *et al.* Artemis: sequence visualization and annotation. *Bioinformatics* **16**, 944-945 (2000).

109. Law, J. H., Ribeiro, J. M. C. & Wells, M. A. Biochemical insights derived from insect diversity. *Ann. Rev. Biochem*. **64**, 87-111 (1992).

110. Mans, J. B. & Neitz, A. W. H. Adaptation of ticks to a blood-feeding environment: evolution from a functional prospective. *Insect Biochem. Mol. Biol*. **34**, 1-17 (2004).

111. Braz, G. R. C., Coelho, H. S. L., Masuda, H. & Oliveira, P. L. A missing metabolic pathway in the cattle tick *Boophilus microplu*s. *Curr. Biol*. **9**, 703-706 (1999).

112. Hamza, I., Chauhan, S., Hassett, R. & O'Brian, M. R. The bacterial irr protein is required for coordination of heme biosynthesis with iron availability. *J. Biol. Chem.* **273**, 21669-21674 (1998).

113. Furuyama, K., Kaneko, K. & Vargas, P. D. Heme as a magnificent molecule with multiple missions: heme determines its own fate and governs cellular homeostasis. *Tohoku J. Exp. Med.* **213**, 1-16 (2007).

114. Machado, E. A., Oliveira, P. L., Moreira, M. F., de Souza, W. & Masuda, H. Uptake of *Rhodnius* heme-binding protein (RHBP) by the ovary of *Rhodnius prolixus*. *Arch. Insect Biochem. Physiol.* **39**, 133-143 (1998).

115. Oliveira, P. L. *et al.* A heme-binding protein from hemolymph and oocytes of the blood-sucking insect, *Rhodnius prolixus*. Isolation and characterization. *J. Biol. Chem*. **270**, 10897-10901 (1995).

116. Weichsel, A., Andersen, J. F., Champagne, D. E., Walker, F. A. & Montfort, W. R. Crystal structures of a nitric oxide transport protein from a blood-sucking insect. *Nat. Struct. Biol*. **5**, 304-309 (1998).

117. Donohue, K. V., Khalil, S. M. S., Sonenshine, D. E. & Roe, R. M. Heme-binding storage proteins in the Chelicerata. *J. Insect Physiol*. **55**, 287-296 (2009).

118. Horigane, M., Shinoda, T., Honda, H. & Taylor, D. Characterization of a vitellogenin gene reveals two phase regulation of vitellogenesis by engorgement and mating in the soft tick *Ornithodoros moubata* (Acari: Argasidae). *Insect Mol. Biol.* **19**, 501-515 (2010).

119. Maya-Monteiro, C. M. *et al.* HeLp, a heme lipoprotein from the hemolymph of the cattle tick, *Boophilus microplus*. *J. Biol. Chem*. **275**, 36584-36589 (2000).

120. Horn, M., *et al.* Hemoglobin digestion in blood-feeding ticks: mapping a multipeptidase pathway by functional proteomics. *Chem Biol.* **16**,1053-1063 (2009).

121. Yamaji, K. *et al.* Hemoglobinase activity of a cysteine protease from the ixodid tick *Haemaphysalis longicornis. Parasitol Int.* **58**, 232-237 (2009).

122. Estrela A. B., Seixas, A., Teixeira Vde, O., Pinto, A. F. & Termignoni, C. Vitellin- and hemoglobin-digesting enzymes in *Rhipicephalus (Boophilus) microplus* larvae and females. *Comp. Biochem. Physiol. B Biochem. Mol. Biol.* **157**, 326-335 (2010).

123. Lara, F. A. *et al.* A new intracellular pathway of haem detoxification in the midgut of the cattle tick *Boophilus microplus*: aggregation inside a specialized organelle, the hemosome. *J. Exp. Biol.* **206**,1707-1715 (2003).

124. Caffrey, C.R. & Ruppel, A. Cathepsin B-like activity predominates over cathepsin L-like activity in adult *Schistosoma mansoni* and *S. japonicum*. *Parasitol. Res.* **83**, 632–635 (1997).

125. Delcroix, M. *et al.* A multienzyme network functions in intestinal protein digestion by a platyhelminth parasite. *J. Biol. Chem.* **281**, 39316–39329 (2006).

126. Guerrero, F. D. *et al.* BmiGI: a database of cDNAs expressed in *Boophilus microplus*, the tropical/southern cattle tick. *Insect Biochem. Mol. Biol.* **35**, 585-595 (2005).

127. Weygoldt, P. Evolution and systematics of the Chelicerata. *Exp. Appl. Acarol.* **22**, 63-79 (1998).

128. Donohue, K. V. *et al.* Neuropeptide signaling sequences identified by pyrosequencing of the American dog tick synganglion transcriptome during blood feeding and reproduction. *Insect Biochem. Mol. Biol.* **40**, 79-90 (2010).

129. Egekwu, N., Sonenshine, D.E., Bissinger, B.W. & Roe, R.M. Transcriptome of the female synganglion of the black-legged tick *Ixodes scapularis* (Acari: Ixodidae) with comparison between Illumina and 454 systems. *PLoS One* **9**:e102667 (2014).

130. Simo, L., Slovak, M., Park, Y. & Źitňan, D. Identification of a complex peptidergic neuroendocrine network in the hard tick *Rhipicephalus appendiculatus. Cell Tissue Res.* **335**, 639–655 (2009).

131. Audsley, N. & Weaver, R. J. Neuropeptides associated with the regulation of feeding in insects. *Gen. Comp. Endocrinol.* **162**, 93-104 (2009).

132. Luo, C. W. *et al.* Bursicon, the insect cuticle-hardening hormone, is a heterodimeric cysteine knot protein that activates G protein-coupled receptor LGR2. *PNAS.* **102**, 2820-2825 (2005).

133. Wu, Q. & Brown, M. R. Signaling and function of insulin-like peptides in insects. *Annu. Rev. Entomol.* **51**, 1–24 (2006).

134. Gu, S.H., Lin, J.L., Lin, P.L. & Chen, C.H. Insulin stimulates ecdysteroidogenesis by prothoracic glands in the silkworm, *Bombyx mori*. *Insect Biochem. Mol. Biol.*, **39**, 171-179 (2009).

135. Mulenga, A. & Khumthong, R. Silencing of three *Amblyomma americanum* (L.) insulin-like growth factor binding protein-related proteins prevents ticks from feeding to repletion. *J. Exp. Biol.* **231**, 1153-1161 (2010).

136. Zhu, X. X. & Oliver, J. H. Jr. Cockroach allatostatin-like immunoreactivity in the synganglion of the American dog tick *Dermacentor variabilis* (Acari: Ixodidae). *Exp. Appl. Acarol.* **25**, 1005-1013 (2001).

137. Lee, M. C., Schiffman, S. S. & Pappas, T. N. Role of neuropeptides in the regulation of feeding behavior: a review of cholecystokinin, bombesin, neuropeptide Y and galanin. *Neurosci. Biobehav. Rev.***18**, 313-323 (1994).

138. Sauer, J. R., Essenberg, R. C. & Bowman, A. S. Salivary glands in ixodid ticks: control and mechanism of secretion. *J. Insect Physiol.* **46**, 1069-1078 (2000).

139. Simo, L. M., Źitňan, D. & Park, Y. Two novel neuropeptides in innervations of the salivary glands of the black-legged tick, *Ixodes scapularis*: Myoinhibitory peptide and SIFamide. *J. Comp. Neurol.* **517**, 551-563 (2009).

140. Yang, Y., Bajracharya, P., Castillo, P., Nachman, R. J. & Pietrantonio, P. V. Molecular and functional characterization of the first tick CAP_{2b} (periviscerokinin) receptor from *Rhipicephalus* (*Boophilus*) *microplus* (Acari: Ixodidae). *Gen. Comp. Endo*. **194**, 142–151 (2013). doi:10.1016/j.ygcen.2013.09.001

141. Yang, Y., Nachman, R.J. & Pietrantonio, P.V. Molecular and pharmacological characterization of the Chelicerata pyrokinin receptor from the southern cattle tick, *Rhipicephalus* (*Boophilus*) *microplus. Insect Biochem. Mol. Biol.* **60**, 13–23 (2015). doi:10.1016/j.ibmb.2015.02.010

142. Neupert, S. *et al.* The neuropeptidomics of *Ixodes scapularis* synganglion, *J. Proteomics*. **72**, 1040-1045 (2009).

143. Hill, C. A. *et al.* G protein-coupled receptors in *Anopheles gambiae. Science* **298**, 176-178 (2002).

144. Nene, V. *et al.* Genome sequence of *Aedes* aegypti, a major arbovirus vector. *Science* **316**, 1718-1723 (2007).

145. Vieira, F. G. & Rozas, J. Comparative genomics of the odorant-binding and chemosensory protein gene families across the Arthropoda: origin and evolutionary history of the chemosensory system. *Genome Biol. Evol.* **3**, 476-490 (2011).

146. Starostina, E., Xu, A., Lin, H. & Pikielny, C. W. A *Drosophila* protein family implicated in pheromone perception is related to Tay-Sachs GM2-activator protein. *J. Biol. Chem*. **284**, 585-594 (2009).

147. Foret, S. & Maleszka, R. Function and evolution of a gene family encoding odorant binding-like proteins in a social insect, the honey bee (*Apis mellifera*). *Genome Res.* **16**, 1404-1413 (2006).

148. Foret, S., Wanner, K. W. & Maleszka, R. Chemosensory proteins in the honey bee: Insights from the annotated genome, comparative analyses and expressional profiling. *Insect Biochem. Mol. Biol*. **37**, 19-28 (2007).

149. Gong, D. P. *et al.* Identification and expression pattern of the chemosensory protein gene family in the silkworm, *Bombyx mori*. *Insect Biochem. Mol. Biol.* **37**, 266-277 (2007).

150. Gong, D. P., Zhang, H. J., Zhao, P., Xia, Q. Y. & Xiang, Z. H. The odorant binding protein gene family from the genome of silkworm, *Bombyx mori. BMC Genomics* **10**, 332 (2009). doi:10.1186/1471-2164-10-332.

151. Vieira, F. G., Sánchez-Gracia, A. & Rozas, J. Comparative genomic analysis of the odorant-binding protein family in 12 *Drosophila* genomes: purifying selection and birth-and-death evolution. *Genome Biol.* **8**, R235 (2007). doi:10.1186/gb-2007-8-11-r235.

152. Flicek, P. *et al.* Ensembl 2008. *Nucl. Acids Res* **36**, D707 (2008). doi:10.1093/nar/gkm988.

153. Soding, J. Protein homology detection by HMM-HMM comparison. *Bioinformatics* **21**, 951-960 (2005).

154. Finn, R. D. *et al.* Pfam: clans, web tools and services. *Nucl. Acids Res.* **34**, D247-D251 (2006). doi:10.1093/nar/gkj149.

155. Katoh, K., Kuma, K., Toh, H. & Miyata, T. MAFFT version 5: improvement in accuracy of multiple sequence alignment. *Nucl. Acids Res.* **33**, 511-518 (2005).

156. Peñalva-Arana, D. C., Lynch, M. & Robertson, H. M. The chemoreceptor genes of the waterflea, *Daphnia pulex*: many Grs but no Ors. *BMC Evol. Biol*. **9**, 79 (2009). doi:10.1186/1471-2148-9-79.

157. Robertson, J. A., Howard, L. A., Zinner, C. L. & Stemke, G. W. Comparison of 16S rRNA genes within the T960 and parvo biovars of ureaplasmas isolated from humans. *Int. J. Syst. Bacteriol.* **44**, 836-838 (1994).

158. Stamatakis, A., RAxML-VI-HPC: maximum likelihood-based phylogenetic analyses with thousands of taxa and mixed models. *Bioinformatics* **22**, 2688-2690 (2006).

159. Croset, V. *et al.* Ancient protostome origin of chemosensory ionotropic glutamate receptors and the evolution of insect taste and olfaction. *PLoS Genetics* **6**, e1001064, (2010). doi:10.1371/journal.pgen.1001064.

160. Kozomara, A. & Griffiths-Jones, S. miRBase: integrating microRNA annotation and deep-sequencing data. *Nucl. Acids Res.* **39**, D152-D157 (2011). doi:10.1093/nar/gkq1027.

161. Gerlach, D., Kriventseva, E. V., Rahman, N., Vejnar, C.E. & Zdobnov, E.M. miROrtho: Computational survey of microRNA genes. *Nucleic Acids Res.* **37**:D111–D117, (2009). doi:10.1093/nar/gkn707.

162. Giraldo-Calderón, G. I. *et al.* VectorBase: an updated bioinformatics resource for invertebrate vectors and other organisms related with human diseases. *Nuc. Acids Res.* **43**(Database issue), D707-13 (2015).

163. Grbić, M. *et al.* The genome of *Tetranychus urticae* reveals herbivorous pest adaptations. *Nature* **479**, 487-492 (2011).

164. Kirkness, E. F. *et al.* Genome sequences of the human body louse and its primary endosymbiont provide insights into the permanent parasitic lifestyle. *Proc. Natl. Acad. Sci. U.S.A.* **107**, 12168-12173 (2010).

165. Munderloh, U. & Kurtti, T. Formulation of medium for tick cell culture. *Exp. Appl. Acarol.* **7**, 219-229 (1989).

166. Riley, C. P. *et al.* The Proteome Discovery Pipeline – A data analysis pipeline for mass spectrometry-based differential proteomics discovery. *The Open Proteomics J.* **3**, 8-19 (2010).

167. Zhang, X., Asara, J. M., Adamec, J., Ouzzani, M. & Elmagarmid, A. K. Data preprocessing in liquid chromatography-mass spectrometry-based proteomics. *Bioinformatics* **21**, 4054-4059 (2005).

168. Riley, C. P. *et al.* A large, consistent plasma proteomics data set from prospectively collected breast cancer patient and healthy volunteer samples. *J. Transl. Med*. **9**, 80 (2011).

169. Kapp, E. A. *et al.* An evaluation, comparison, and accurate benchmarking of several publicly available MS/MS search algorithms: Sensitivity and specificity analysis. *Proteomics* **5**, 3475-3490 (2005).

170. Munderloh, U. G. *et al.* Invasion and intracellular development of the human granulocytic ehrlichiosis agent in tick cell culture. *J. Clin. Microbiol.* **37**, 2518–2524 (1999).

171. Villar, M. *et al.* Identification and characterization of *Anaplasma phagocytophilum* proteins involved in infection of the tick vector, *Ixodes scapularis*. *PLOS ONE* **10**, e0137237 (2015). doi: 10.1371/journal.pone.0137237.

172. Bonzon-Kulichenko, E. *et al.* A robust method for quantitative high-throughput analysis of proteomes by ^{18}O labeling. *Mol. Cell Proteomics* **10**:M110 003335 (2011). doi:10.1074/mcp.M110.003335.

173. López-Ferrer, D. *et al.* Statistical model for large-scale peptide identification in databases from tandem mass spectra using SEQUEST. *Anal Chem.* **76**, 6853-6860 (2004).

174. Navarro, P. & Vazquez, J. A. refined method to calculate false discovery rates for peptide identification using decoy databases. *J. Proteome Res.* **8**, 1792-1796 (2009).

175. Catchen, J. M., Amores, A., Hohenlohe, P., Cresko, W. & Postlethwait, J. H. Stacks: building and genotyping Loci de novo from short-read sequences. *G3 (Bethesda)* **1**,171-82 (2011). doi: 10.1534/g3.111.000240.

176. Catchen, J., Hohenlohe, P. A., Bassham, S., Amores, A. & Cresko, W. A. Stacks: an analysis tool set for population genomics. *Mol Ecol.* **22**, 3124-3140 (2013). doi: 10.1111/mec.12354

177. Langmead, B. & Salzberg, S.L. Fast gapped-read alignment with Bowtie 2. *Nat. Methods* **9**, 357-359 (2012).

178. Wright, S. Evolution and the Genetics of Population, Variability Within and Among Natural Populations. The University of Chicago Press, Chicago (1978).

179. Hohenlohe, P. A. *et al.* Population Genomics of Parallel Adaptation in Threespine Stickleback using Sequenced RAD Tags. *PLoS Genet.* **6**: e1000862. doi:10.1371/journal.pgen.1000862 (2010).

180. Nielsen, R. *et al*. Darwinian and demographic forces affecting human protein coding genes Darwinian and demographic forces affecting human protein coding genes. *Genome Research* **19**, 838–849 (2009). doi:10.1101/gr.088336.108

181. Weir, B. & Cockerham, C. C. Estimating F-Statistics for the Analysis of Population Structure. *Evolution* **38**(6), 1358–1370 (1984).

182. Raj, A., Stephens, M. & Pritchard, J. K. fastSTRUCTURE: variational inference of population structure in large SNP data sets. *Genetics* **197**:573-89. doi: 10.1534/genetics.114.164350 (2014).

183. Rosenberg, N. A., DISTRUCT: a program for the graphical display of population structure. *Mol. Ecol. Notes* **4,** 137–138 (2004).

184. Buckingham, S. D., Pym, L. & Sattelle, D. B. Oocytes as an expression system for studying receptor / channel targets of drugs and pesticides. *Methods in Molecular Biology* **322**, 331-345. (ed X. J. Liu) Humana Press Inc., Totowa, NJ, USA. (2006).

185. Guittard, E. *et al.* CYP18A1, a key enzyme of *Drosophila* steroid hormone inactivation, is essential for metamorphosis. *Dev. Biol.* **349,** 35-45 (2011). doi: 10.1016/j.ydbio.2010.09.023.

186. Jongejan, F. & Uilenberg, G. The global importance of ticks. *Parasitology* **129**(Suppl):S3-S14 (2004). doi: http://dx.doi.org/10.1017/S0031182004005967

187. Van Zee, P. *et al.* Tick genomics: the *Ixodes* genome project and beyond. *Intl. J. Parasitol.* **37** (12) 1297-1305 (2007).
